# Macroevolution of hyperdiverse flightless beetles reflects the complex geological history of the Sunda Arc

**DOI:** 10.1038/srep18793

**Published:** 2016-01-08

**Authors:** Rene Tänzler, Matthew H. Van Dam, Emmanuel F. A. Toussaint, Yayuk R. Suhardjono, Michael Balke, Alexander Riedel

**Affiliations:** 1 SNSB-Zoological State Collection (ZSM), Münchhausenstr. 21, D-81247 Munich, Germany; 2 Division of Zoology, Cibinong Science Center – LIPI (MZB), Jl. Raya Jakarta- Bogor, Indonesia; 3 Department of Ecology & Evolutionary Biology & Division of Entomology, Biodiversity Institute, University of Kansas, Lawrence, KS 66045, USA; 4 GeoBioCenter, Ludwig-Maximilians-University, Munich, Germany; 5 Museum of Natural History Karlsruhe (SMNK), Erbprinzenstr. 13, D-76133 Karlsruhe, Germany

## Abstract

The Sunda Arc forms an almost continuous chain of islands and thus a potential dispersal corridor between mainland Southeast Asia and Melanesia. However, the Sunda Islands have rather different geological histories, which might have had an important impact on actual dispersal routes and community assembly. Here, we reveal the biogeographical history of hyperdiverse and flightless *Trigonopterus* weevils. Different approaches to ancestral area reconstruction suggest a complex east to west range expansion. Out of New Guinea, *Trigonopterus* repeatedly reached the Moluccas and Sulawesi transgressing Lydekker′s Line. Sulawesi repeatedly acted as colonization hub for different segments of the Sunda Arc. West Java, East Java and Bali are recognized as distinct biogeographic areas. The timing and diversification of species largely coincides with the geological chronology of island emergence. Colonization was not inhibited by traditional biogeographical boundaries such as Wallace’s Line. Rather, colonization patterns support distance dependent dispersal and island age limiting dispersal.

The Indo Australian Archipelago (IAA) is the geologically complex transition zone between Oriental and Australian biota and arguably the most biodiverse region on the planet^1^. Biogeographers ever since Wallace have sought to understand the mechanisms behind the high diversity of the IAA and its evolution in space and time. Recently, several comprehensively sampled molecular phylogenies have helped to reveal underlying historical processes e.g. across Sundaland^2^, around Sulawesi^3^, the Philippines^4^,^5^ and Melanesia^6^. However, the Sunda Arc along the southern border of the IAA^7^ remains poorly studied, especially so the Lesser Sunda Islands comprising Bali, Lombok, Sumbawa and Flores. The pioneering work of Rensch^8^ reflects a great interest at the time but became outdated with the advent of plate tectonic theory and phylogenetics^9^,^10^.

Yet, the Sunda Arc provides a compelling setting to study biotic evolution along a complex island chain. Its islands are arranged like pearls on a string, slightly south of the equator. The waterways between islands are usually narrow, e.g. Bali Strait between Java and Bali is currently less than 3 kilometres wide, or Alas Strait between Lombok and Sumbawa <15 km. Today, and for approximately the last ten million years the islands of the Sunda Arc were aligned along the same trench, but their age and geological history differ greatly^11^,^12^. While Java is geologically of continental origin, the volcanic influence markedly increases towards its East. Bali, Lombok and Sumbawa are entirely of volcanic origin, emergent since ~11 million years ago (Ma) while Flores to the east presumably emerged later, c. 9 Ma^12^. Sumba is a remarkable exception, being a continental splinter from the Sunda Shelf pushed to the southeast^13^.

During the Pleistocene glacial maxima those islands located on the Sunda Shelf were connected with each other and the Asian mainland^14^,^15^. The resulting faunal exchange, e.g. between Java and Bali resulted in faunal similarities which are in stark contrast to the ones of islands further east. The deepwater Lombok Strait between Bali and Lombok, however, was never dry^14^,^16^. The resulting differences particularly in vertebrate faunas to the east and west of the Lombok Strait led Wallace^17^ to suggest a sharp division of biogeographical regions, known as Wallace’s Line^16^,^18^.

Habitats in the exposed Java Sea connecting the Sunda Shelf islands might have included savannah, heath forests or peat swamps^19^. These acted as an ecological filter favouring taxa of higher mobility, particularly mammals^20^,^21^,^22^,^23^. Some species, e.g. the tiger (*Panthera tigris* Linnaeus) had reached Sumatra, Java and Bali but were always absent from Borneo^24^. This Pleistocene faunal exchange occurred only in the relatively recent past of a much older fauna. The story of Wallace’s Line thus appears relevant only for some taxa and for just a fraction of the time-span during the biotic evolution of the IAA. The climatic processes producing the high visibility and prominence of Wallace’s Line may in fact distract from older geological patterns of faunal assembly in the IAA^25^.

Arthropods constitute the vast majority of species diversity across the IAA and the Sunda Arc, but their biogeographic origins remained mostly unknown until recently^21^,^26^,^27^,^28^,^29^,^30^,^31^,^32^,^33^,^34^. In some cases they show unexpected colonization histories including long-distance dispersal^35^,^36^. Some of the taxa with broad distributions across the IAA have a natural history that appears to be counterintuitive to the observed patterns, i.e. they are flightless and/or have little tolerance to seawater. Such taxa include the fanged frogs that originated in SE Asia and then spread across the IAA, with frequent exchange between Borneo, Philippines and Sulawesi, crossing both Wallace’s and Huxley’s lines^37^. Flightless *Trigonopterus* weevils crossed Wallace’s Line three times to reach Bali from the east^38^, mite harvestmen reached New Guinea twice from Sundaland^26^ and land snails reached the Lesser Sunda Islands from Australia^39^.

Here we study hyperdiverse and flightless *Trigonopterus* weevils that might have more than 1,000 species across the region^40^,^41^. This extraordinary diversity, limited dispersal ability and high degree of local endemism allows for fine scale analyses of patterns and processes in the faunal evolution of the IAA and the Sunda Arc. The genus *Trigonopterus* has a wide range, from east Sumatra across Melanesia to Samoa. New Guinea is a centre of diversity (>300 species recorded,^42^). Sulawesi is another hotspot with >100 species (Riedel, in prep.). Diversity decreases to the west, but is still substantial compared to other organisms; there are at least 100 species in Sundaland and the Lesser Sunda Islands^43^. All except two species are microendemics. The high level of species endemism provides many replicated natural experiments to understand the processes generating tropical diversity. Here we explore their biogeographical history and colonization patterns of the IAA in general and the Sunda Arc in particular.

*Trigonopterus* of Sundaland and the Lesser Sunda Islands are exclusively edaphic, inhabiting the leaf-litter, while in Sulawesi and New Guinea many species browse on foliage. In the relatively dry islands of Sumbawa and Flores, *Trigonopterus* are confined to montane areas of wet primary forests while they are absent from seasonal monsoon forests. Extensive areas of East Java and the Lesser Sunda Islands at low elevations and/or in rain shadows do not support habitats suitable for *Trigonopterus*, perhaps similar to the exposed areas of the Sunda Shelf during the Pleistocene glacial maxima.

Based on comprehensive taxon sampling we reconstruct the molecular phylogeny for the Sunda Arc *Trigonopterus*, infer divergence time estimates using multiple calibration strategies and use these data to investigate the biogeographical history of this lineage. Our findings suggest that lineage accumulation over time largely coincides with relative island age except for Sumatra and Borneo both of which were presumably outside of the initial area of range expansion. We find multiple east-west range expansion events out of New Guinea. Sulawesi acted as a colonization hub for multiple independent colonization events across different segments of the Sunda Arc. The Sunda Arc’s fauna is the result of a complex over water colonization history, with no back colonization to source areas.

## Results

### Molecular phylogenetics

**Figure 1:**
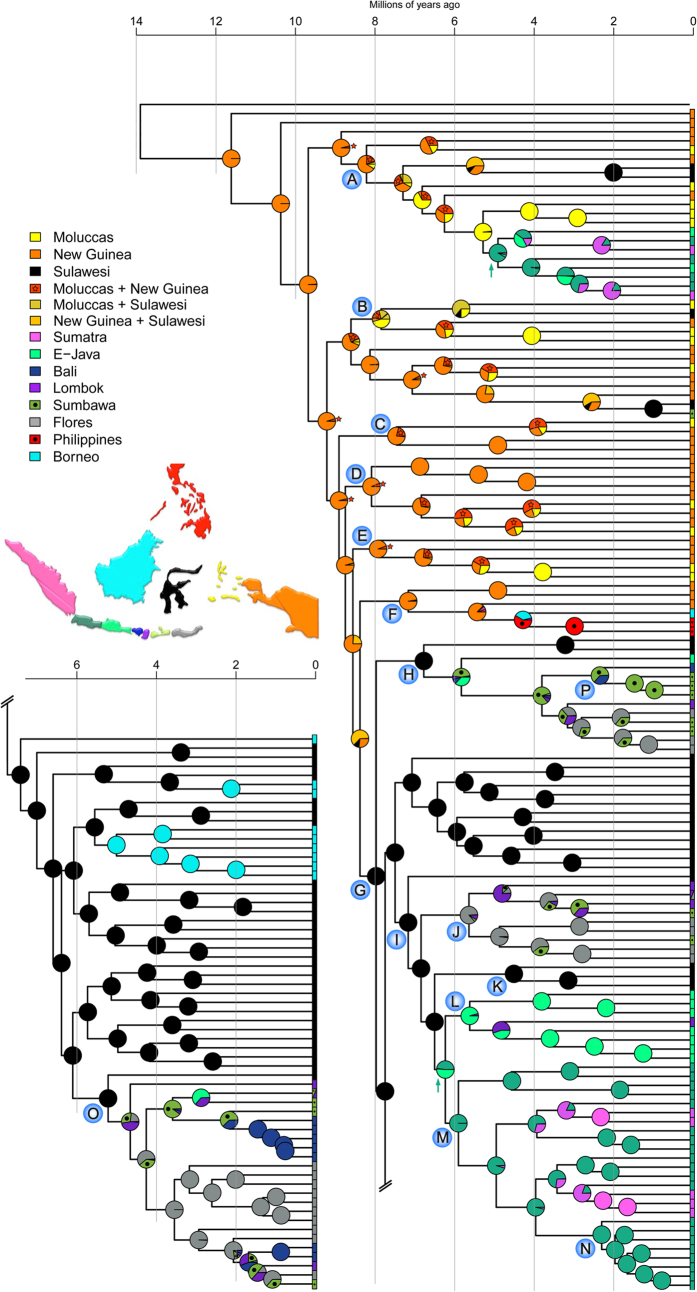
Historical biogeography of *Trigonopterus* weevils inferred from model-based analysis. The Bayesian phylogeny was dated using the re-emergence of Java at 10 Ma (analysis 2). The chronogram is presenting the median divergence time estimates resulting from the BEAST analysis. Ancestral areas were inferred using a time-stratified DEC + J + x model. The distribution of each taxon is given in a geographical matrix on the right side of the chronogram with colours as coded in the inset. Coloured pie-charts indicate the likelihood of ancestral areas as recovered at each node. Nodes referred to in the text are marked by letters A to O. The map inset was created in GeoMapApp v3.4.1 http://www.geomapapp.org and modified in Adobe Photoshop CS2.

The best-fit partitioning strategy recovered by PartitionFinder is shown in Suppl.-Table 11. The Bayesian inference (BI) analyses converged well with ESS values >200 for all parameters. The Bayesian inference, maximum parsimony, and maximum likelihood reconstructions recovered highly congruent topologies for the species of the Sunda Arc (Fig. 1, S1, S3). Node support was high (≥0.95 posterior probability in BI) for nodes critical to our inference (Fig. 1, S1). Some clades of the New Guinea/Moluccas have lower nodal support (e.g. clades D, E), but they are nested among other clades of the same areas and therefore do not affect our conclusions.

### Divergence time estimation

Using the emergence and formation of a larger New Guinea landmass beginning at 30 Ma placed the root of *Trigonopterus* at 29.9 Ma with a 95% height posterior distribution (HPD) of 27.8–31.7 Ma (Analysis 1, Fig. S2). This suggests a CO1 substitution rate of 0.0315 substitutions per site per million year (Myr) per lineage (subs/s/Myr/l) (0.0342 subs/s/Ma/l for the 3′ fragment). Analysis 2 was based on the re-emergence of Java at 10 Ma following a complete inundation of shallow sea^11^,^44^. This recovered the root of *Trigonopterus* at 11.6 Ma (Fig. 1) (95% HPD: 9.1–14.2 Ma) resulting in CO1 substitution rates of 0.0793 subs/s/Myr/l (0.0865 subs/s/Myr/l for the 3′ fragment).

### Biogeographical model testing

The results from the Maximum Likelihood (ML) estimation of biogeographical models are given in S-Table 8, which also provides the results for the model parameters d (rate for range expansion), e (rate for range contraction/extinction), J (allowing for jump dispersal) and x (a dispersal rate multiplier)^45^,^46^. The parameters J and x are fixed when reported as 0 values. The results of the Likelihood Ratio Tests (LRT) between models with and without the +J parameter (S-Table 9) are all in favour of the Dispersal-Extinction-Cladogenesis (DEC)-type models that included the +J parameter. Comparison of the different models with Akaike Information Criterion corrected for sample size (AICc) (Table 1), revealed support for the DEC + J + x model with dispersal constrained to adjacent areas except for the Lesser Sunda Islands chain. A constrained model receiving a higher log-likelihood (LnL) may seem counterintuitive. However, this is possible by giving a higher probability to just a few “correct events” (i.e. giving a better ML estimate with fewer events) to explain the tip data while low probability events are ruled out, thus optimizing d and e. The two constraint models received less support when distance was not included. The constrained model where dispersal was allowed between any of the Lesser Sunda Islands always received more support over the “adjacency only constrained model” where dispersal was only allowed between adjacent islands in the Lesser Sunda Islands chain. The unconstrained model received less support than the constrained model where dispersal was allowed between any of the Lesser Sunda Islands (Table 1).

### Parsimony area reconstruction

The results from the parsimony area reconstruction (Fig. S1) were broadly congruent with the preferred BioGeoBEARS analyses using the DEC + J + x model (Fig. 1, S2). This demonstrates the robust signature of the following biogeographical history.

### Sunda Islands biogeography in space

**Figure 2:**
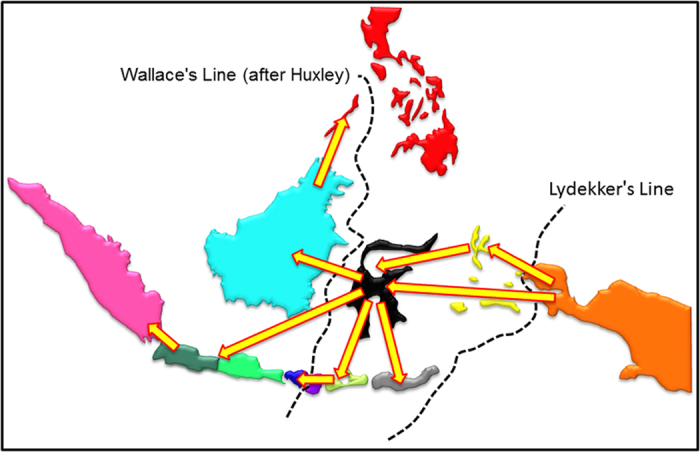
Synopsis of major dispersal events of *Trigonopterus*. From New Guinea, *Trigonopterus* repeatedly reached the Moluccas and Sulawesi transgressing Lydekker′s Line. Sulawesi became a secondary centre of diversification and colonization hub for different segments of the Sunda Arc with independent lineages. The map was created in GeoMapApp v3.4.1 http://www.geomapapp.org and modified in Adobe Photoshop CS2.

*Trigonopterus* of the Wallacea, the Philippines, and Sundaland (see^1^ for a definition of these areas) originated in New Guinea from where they expanded their range westward repeatedly transgressing Lydekker’s and Wallace’s Line (Figs 1 and 2; S1). The Moluccas and Sulawesi were stepping stones to the Sunda Arc; Sulawesi also evolved numerous endemic species. There were at least 12 westward colonization events out of New Guinea (Fig. 1, S1 clades A–F; Fig. 2). The vast majority of species west of New Guinea belongs to clade G, which is also the oldest westward range expansion (Fig. 1).

The early lineages of clade A are from New Guinea and the Moluccas from where Sulawesi and West Java were colonized independently; East Java and Sumatra were repeatedly colonized from West Java. Clade B originated in New Guinea from where it reached the Moluccas and Sulawesi and in an independent subclade Sulawesi and Sumbawa. Clades C, D and E reached the Moluccas from New Guinea. In clade F, we find a westward range expansion from New Guinea to Borneo and the Philippines. Clade G contains almost the entire fauna of Sulawesi, Borneo and the Sunda Arc, with four independent colonization events from Sulawesi towards the Sunda Arc followed by subsequent diversification: 1) clade H to East Java/Sumbawa and later to Bali, Lombok, and Flores; 2) clade J to Flores and from there to the other Lesser Sunda Islands; 3) the common ancestor of clades L and M to Java; 4) clade O to Lombok/Sumbawa and from there to Flores, followed by two independent colonization events to Bali^38^.

In Java, we find an early dichotomy between West Java (clade M) and East Java (clade L). There is little exchange between the two areas in clade A. West Java acts as a launching pad to Sumatra in clades A and M. East Java is the stepping stone to the Lesser Sunda Islands for clade L. There is no exchange between West and East Java in the relatively diverse clades L and M.

### Sunda Islands biogeography in time

*Trigonopterus* reached Sulawesi or parts thereof relatively early during their evolution, according to our tentative dating either 22.0 Ma using the emergence of New Guinea as calibration, or 8.0 Ma using the emergence of Java as a calibration (in the following, dates of this second calibration in parentheses) (Fig. 1, S2). The colonization of the Sunda Islands started with the ancestor of clades L and M in Java at 16.8 Ma (6.2 Ma) and continued with clade J in the Lesser Sunda Islands 15.6 Ma (5.6 Ma). With slight delay followed clade A in Java at 14 Ma (4.9 Ma), respectively clade O in the Lesser Sunda Islands at 13.6 Ma (4.7 Ma). Colonization of Sumatra started much later with lineages of clade M at 5.6 Ma (3.2 Ma). Colonization of Bali by lineages of clade O commenced at 7.0 Ma (1.5 Ma). The relatively recent radiation of clade N comprising eight species of mid to upper montane forests of West Java begins 5.0 MA (2.2 MA).

## Discussion

Substitutions rates for *Trigonopterus* (Analysis 1, 2) were here inferred from large scale paleogeographical events. These rates were up to 7.5 times higher (0.0115 vs 0.0865) than other published arthropod rates ranging from 0.0115 to 0.0195 subs/s/Myr/l (e.g.^47^,^48^,^49^,^50^). This however is in line with recent evidence linking flight loss to accelerated molecular evolution (Mitterboeck & Adamowicz, 2013), which was suspected for flightless *Trigonopterus* as well^38^. Thus, the timing of events remains tentative in the absence of specific ingroup fossils. Yet, our ancestral area estimations were largely congruent among different analyses (undated parsimony *versus* differently dated model based). This may in part be due to each species being found in only one area and *Trigonopterus* of the IAA being strongly structured geographically. While the parsimony analysis is free of temporal assumptions, the sequence of events in the analysis reveals a relative chronological order, which agrees with the dated, model based ancestral area inferences.

Our results reveal a New Guinean origin of Wallacean and Sundaland *Trigonopterus*. Species diversity of the Sunda Arc Islands has been derived through at least six colonization events followed by *in situ* diversification on different segments of the Sunda Arc (Fig. 2, S1). The highly diverse clade G arrived and diversified in Sulawesi and then expanded west or south with four colonization events to the Sunda Arc each followed by rapid diversification. It remains west of the Moluccas, with no back colonization to New Guinea or the Moluccas. Thus, the overall directionality of the range expansion is in the east to west direction. Colonization of the Sunda Islands equally appears as a one-way street, at least when following the parsimony, and the second model-based analysis respectively. The first model-based analysis interprets clade K as a back-colonization of Sulawesi from Java, but this scenario does not appear compelling, adding more steps to the colonization process.

Here, we show that the Sunda Arc is biogeographically broken into different segments, not all conforming to passages between islands or biogeographic lines such as Wallace’s. The Bali Strait is the most striking barrier, separating the continental island of Java from the volcanic Lesser Sunda Islands. Only two transgressions have occurred here between East Java and Lombok/Sumbawa (Fig. 1, S1, clade H, clade L), which is remarkable considering the rich faunas on either side not transgressing this barrier. In comparison, Wallace’s Line between Bali and Lombok and the Sunda Strait between Sumatra and Java were distinctly more porous with three and four transgressions respectively^38^. Apparently, the geological boundary between continental and volcanic islands is more significant than Wallace’s Line, largely inferred from climatic effects of Pleistocene sea level minima. A simple relationship between the age of terranes and the number of transgressions can be ruled out because most of these transgressions took place at a time when all islands were already available to colonization according to the Java calibrated reconstruction. However island age cannot be entirely ruled out as a significant factor contributing to the colonization process, as there is uncertainty in the age of the phylogeny.

This study also reveals an intra-island division between West and East Java with deep divergence of clades L and M (Fig. 1, S1). This pattern appears to confirm the separation of West and East Java as separate islands that re-emerged from the Java Sea between 10 to 5 Ma, and coalescing to form Java in its current shape only quite recently^11^,^44^. The Javanese species of clade A (Fig. 1, S1) show two transgressions of Central Java, but these occurred at ca. 3.2 Ma and 4.3 Ma based on model-based analysis 2, at a time when West and East Java were possibly already connected. Dispersal among the Lesser Sunda Islands was relatively frequent, also reflected in the model-based analyses by the adjacency constraints receiving a lower likelihood than the unconstrained results.

**Figure 3:**
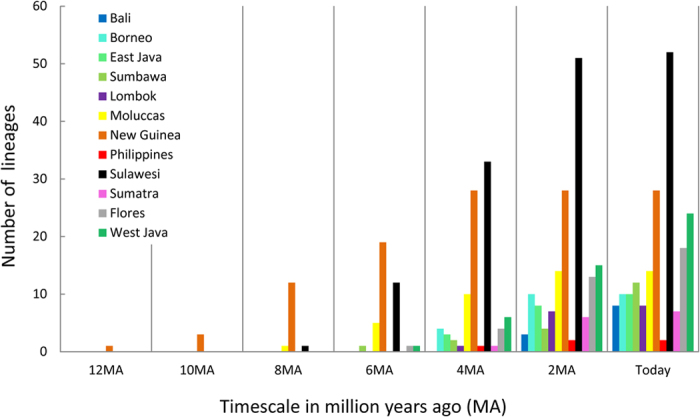
Lineage accumulation per area over time, revealing range expansion as new resources become available or in reach. Numbers of lineages are counted in time-slices of 2 MA-intervals in analysis 2 (see Fig. 1). For New Guinea, only a subset of the available species diversity was used resulting in negative bias for recent lineages.

Sulawesi’s early colonization entailing prompt diversification (Fig. 3) and its central position in the heart of the IAA made it a launching pad for the colonization of the more recent Sunda Islands. From Sulawesi there are five colonisation events to the rest of the Sunda Arc, plus three to Borneo. This leaves only two long distance colonization events from New Guinea/Moluccas directly to the Sunda Islands (Fig. 1, S1, clades A, F), which might however be artefacts caused by missing species from interspersed areas.

Sulawesi was colonized at different time slices: clade G arrived ca. 22.0 Ma (8.0 Ma), clade B arrived 12.2 Ma (5.8 mya), respectively 2.0 Ma (1.0 Ma), and clade A 6.5 Ma (2.0 mya) (Analysis 1/2, respectively). This step-by-step accumulation of lineages over time (Fig. 3) rather than simultaneous arrival suggests a prominent role of over water dispersal rather than major vicariance events in the formation of the Sulawesi fauna. The earliest inferred arrival at 22.0 Ma coincides with the collision of the Sula spur, a large promontory of the Australian continental margin, with the volcanic arms of north/northeast Sulawesi^12^,^51^ and although this may not have transposed an entire fauna to proto-Sulawesi, it was probably a time promoting dispersal by the close contact of the respective terranes.

Thus, concerning the colonization of Sulawesi the time-frame of analysis 1 is consistent with current geological opinion. The almost instant diversification appears somewhat earlier than in other taxa, generally starting not before 20 Ma^51^ and attributed to the small area of proto-Sulawesi at its early days. However, it is more problematic to see colonization and subsequent radiation on Java at 18 Ma and at Flores at 15 Ma. Hall^52^ suggests that “most of Sumatra and Java were elevated above sea level and emerged to their present size only since 5 Ma, and much of East Java continued to be the site of marine deposition until late in the Pliocene or even Pleistocene.” Flores appeared as the last of the Lesser Sunda Islands at 9 Ma^12^. Therefore, analysis 2 was performed which resulted in a staggering evolutionary rate of 0.0793 subs/s/Ma/l in CO1 but at the same time in an overall scenario more consistent with current geological opinion^11^,^12^,^44^,^52^. Here, Java would have been colonized by the first lineage at 6.2 Ma and Flores at 5.8 Ma. In the reconstructions of Spakman & Hall^12^ the islands of Bali, Lombok and Sumbawa appear at 11 Ma, and Flores slightly later at 9 Ma although the latter was the first of the Lesser Sundas with a radiation of *Trigonopterus*. According to Barberi *et al.*^53^ Sumbawa may be largely of Pleistocene age with Mt. Sangenges dated as 1.71 Ma. This agrees well with the age of the *T. sumbawensis*-clade P found at this mountain and neighbouring Mt. Batu Pasak dated 1.5 Ma according to analysis 2. Similarly, according to analysis 2 clade N radiated largely during the Pleistocene concurring with Quarternary volcanism building mountains such as Mt. Gede^7^. Diversification in New Guinea would have commenced at 11.6 Ma, which would be contemporaneous with the second phase of orogeny in New Guinea, the uplift of the Central Range^54^. Opening of the Sunda Strait started before 2 Ma^55^, which is consistent with analysis 2 where all four dispersal events from Java to Sumatra took place shortly before this time. Since then the Sunda Strait has acted as an effective barrier for dispersal between West Java and Sumatra.

The colonization of Lombok (Fig. 1, S1, clades J, L, O) appears relatively early in time, compared to the colonization of Bali (Fig. 1, S1, clade O), a geologically similar island; however, recent volcanic deposits may have coved earlier rocks, making it very difficult to determine island age by stratigraphy. Possibly, our results could indicate a longer above-water-history of Lombok compared to Bali. Biogeographical data of other organisms should be examined for congruence. Unfortunately only a few published phylogenies include representatives of single island endemics for the Western Lesser Sunda Islands. For instance in^37^, where one species of fanged frogs (*Limnonectes kardasani*) an endemic to Lombok was included but the absolute age is not provided for its divergence between the Javanese sister species. More often the focus is on mobile organisms, i.e. butterflies and birds, which have endemic species on the larger and more isolated Eastern islands, e.g. Sumba, Timor, or Wetar, while the islands of Bali to Flores are usually inhabited by the same widespread species^30^,^31^,^32^,^56^. This highlights the need for more biogeographical studies on less vagile taxa, e.g. frogs, molluscs and flightless arthropods from the Lesser Sunda Islands.

Patterns of organisms transgressing Wallace’s Line are often complex, with frequent back-colonizations^32^,^57^,^58^. Organisms with weaker dispersal abilities may exhibit fewer transgressions leading to an increase in diversification rates^59^ or resulting in a single radiation^60^,^61^. The range expansion of *Trigonopterus* is rather unidirectional and shows a conspicuous absence of back-colonization, similar to a pattern found in another group of flightless weevils^33^. It is remarkable that a genus of flightless insects managed to colonize an area spanning >9,000 km from east to west, including expanses of deep open ocean of the Western Pacific. While our reconstructions show high conservatism to a given terrane, inter-island dispersal does occur and seems to increase among smaller close-by islands, i.e. the Lesser Sunda Islands. Additionally our biogeographical model shows strong support for distance limiting dispersal in this taxon. Few means of dispersal appear possible, but presumably drift by sea currents is the most likely one. Little is known on the biology of *Trigonopterus*, but presumably larvae develop in dead wood like many other Cryptorhynchinae^62^. It is thus possible that these weevils get swept to the sea with floating twigs or other substrate and are then carried as flotsam to another shore. Sea currents in the region of the Indonesian Throughflow^52^,^63^,^64^ are subject to seasonal and tectonic changes, but in general the South Equatorial Current follows the New Guinea coastline from East to West. During the Oligocene this would have meant a direct connection from the area of New Guinea to the Sunda Shelf^65^. Once Sulawesi was formed partly blocking the gateway between Pacific and Indian oceans, patterns changed and a strong southward current in the Makassar Strait became dominant. This current would constantly bring flotsam from Sulawesi to the Lesser Sunda Islands. Basic biological information would allow the modelling of dispersal by sea currents, e.g. the duration and substrate of larval development, survival in sea water etc.^66^; in the absence of these data we can only observe that the patterns found are well compatible with a scenario of sea current dispersal. It would also explain the unidirectionality since a drift from the Sunda Arc northwards to Sulawesi would be highly unlikely with the present system of sea currents.

The exchange between Sahul and Sunda floras is dominated by dispersal from West to East, and zoochorous lineages were overrepresented among dispersers suggesting mainly birds or bats as primary means of long-distance dispersal between the two shelves^67^,^68^. However, a study on the entire flora of Java finds it closely related to Sulawesi and the Lesser Sunda Islands, which is attributed to climatic reasons by Van Welzen *et al.*^69^. This is not a suitable explanation for the similar patterns exhibited by *Trigonopterus* weevils usually restricted to pockets of everwet rainforest, even in highly seasonal areas. Possibly, these common patterns between plants and weevils are more strongly influenced by other factors than climate, i.e. the sea currents directed form Sulawesi to Java and the Lesser Sunda Islands.

Reconstructions of Pleistocene vegetation types^70^,^71^ are at odds with local radiations of *Trigonopterus* in East Java and the Lesser Sunda Islands dating back to the Miocene (Fig. 1, S1, clades H, I, J) as these areas are supposedly covered with seasonally dry forests during the Pleistocene. Even today, *Trigonopterus* species are highly restricted to small pockets of wet montane forests and these must have persisted to explain current patterns. Most likely, climate models are not detailed enough to account for localized areas of high precipitation on isolated mountains, but these montane pockets do matter as long-term refugia for an endemic arthropod fauna. Thus, geology, distance and sea currents were probably the dominant factors shaping *Trigonopterus* biogeography, not climate.

## Materials and Methods

### Taxon sampling and DNA sequencing

We sampled 96 *Trigonopterus* species from Sundaland and the Lesser Sunda Islands representing all species known from that region except for two species from Java and one from Flores^43^ (Supplement 3). We added 94 representatives from neighbouring areas (Sulawesi: 52; Moluccas: 13; New Guinea: 29). Altogether, our dataset contains 190 *Trigonopterus* species (one represented by two subspecies) plus four outgroup representatives of other cryptorhynchine species from Australia, New Guinea and Java (*Critomerus iliacus* (Pascoe); *Microporopterus* cf. *setosus* Voss; *Ouporopterus squamiventris* Lea; *Miocalles* sp.). All *Trigonopterus* species from the Sunda region and some from New Guinea possess a valid name^43^,^72^, while most of the species from Sulawesi are currently being revised and described. Undescribed species are referred to by unique species numbers that will be provided in future taxonomic treatments. All the species were monophyletic in a phylogeny using CO1 data of multiple specimens per species, and also well delineated by male genital characters. The high diversity of *Trigonopterus* in New Guinea^42^ was represented by the selection of species used earlier to represent major lineages^38^.

### Extraction, PCR, sequencing and alignment

DNA was extracted non-destructively using the DNeasy and NucleoSpin 96 Tissue kits (Qiagen, Hilden; Macherey-Nagel, Düren, Germany). Voucher specimens are used for subsequent taxonomic studies (e.g.^43^,^72^) and are stored in our museum collections (MZB, SMNK, SMNK). Primers and PCR conditions are listed in Table S10; for additional information on our standard protocols see http://zsm-entomology.de/wiki/The_Beetle_D_N_A_Lab. We sequenced 12 gene fragments with an alignment of 6,511 base pairs (bps) consisting of fragments from cytochrome oxidase subunit 1 (CO1) (2 non-overlapping fragments), 16S mtDNA, Arginine kinase (AK), carbamoyl-phosphate synthetase 2 (CAD) (3 non-overlapping fragments), Elongation factor 1α (EF1 α), Enolase (EN), Histone 4 (H4), 18S and 28S. Sequences were edited using Sequencher 4.10.1 (GeneCodes Corp., Ann Arbor, MI, USA). Coding genes were aligned in MUSCLE^73^, noncoding genes in MAFFT version 7^74^. Sequences were color-coded by amino acid in MEGA6^75^ and checked for stop codons. The final dataset was generated in Sequence Matrix 1.7.2 (Ref. 76).

### Phylogenetic analyses

Relationships among *Trigonopterus* species were reconstructed using maximum parsimony (MP), maximum likelihood (ML) and Bayesian Inference (BI). TNT 1.1^77^,^78^ was used for MP analyses with “traditional search” and 1000 replicates. ML analyses were conducted with the best partitioning scheme selected in PartitionFinder 1.1.1 (79; Table S11) using IQ-Tree^80^,^81^ with standard parameters. We conducted 1000 bootstrap replicates (BS) which are indicating the level of support at each node. A calculated BS ≥ 70 was considered to indicate strong support for a given clade in the ML analyses^82^. BI analyses were conducted in MrBayes 3.2^83^, using the different partitions recovered by PartitionFinder; instead of using the selected substitution models, reversible-jump MCMC has been used to explore the entire space of substitution models^84^. We sampled 60 million generations of two independent runs consisting of eight Markov Chains Monte Carlo (MCMC) sampling every 5000th generation. Split-frequencies and log-likelihood curves were examined in Tracer 1.5 (http://beast.bio.ed.ac.uk/Tracer); the first 2.000 out of 12.000 trees sampled were removed as burnin and a 50% majority rule consensus tree was constructed based on the remaining trees. A calculated PP ≥ 0.95 was considered to indicate strong support for a given clade in the BI analyses^85^.

### Estimation of divergence times

Phylogenetic trees were dated using the Bayesian relaxed clock method implemented in BEAST 1.8.1^86^. Each of the Markov chain Monte Carlo (MCMC) analyses were run for 100 million generations to reach stationarity, with trees and model parameters sampled from the stationary posterior distribution. Stationarity was assessed using the program Tracer version 1.5. Exploratory analyses have shown a much too old dating when using published rates for Coleoptera, e.g. calibration with 0.0177 substitutions per site per Ma per lineage^50^ placed the earliest node of *Trigonopterus* at 66.05 Ma (95% HPD: 49.99–89.51 Ma) which would predate the node of non-cryptorhynchine Molytinae and the cryptorhynchine *Acalles*, an early genus of the group producing *Trigonopterus* (87; unpublished data). This is not entirely surprising as the rate of molecular evolution in flightless beetles, especially groups inhabiting stable habitats might be highly accelerated (Ikeda *et al.*, 2012; Vogler & Timmermans, 2012; Mitterboeck & Adamowicz, 2013)^93^,^94^,^95^ which could in part also explain the high interspecific divergence in *Trigonopterus*^38^. There are no *Trigonopterus* fossils and general weevil fossils cannot be used due to the uncertain monophyly of Cryptorhynchinae and the unresolved placement of *Trigonopterus* within this subfamily. The only time-calibrated tree of Curculionidae available^87^ did not recover Cryptorhynchinae as monophyletic, perhaps because >30% of the data was missing. Furthermore, the scant fossil record of this subfamily does not offer a taxon to which *Trigonopterus* could be safely attributed. As a result, we were not able to use a secondary calibration for the *Trigonopterus* radiation. Here we used the emergence of more extensive amounts of land of New Guinea at 30 Ma^11^,^52^,^54^ (analysis 1, Fig. S2) and alternatively the emergence of Java at 10 Ma – following a complete inundation by shallow sea^11^,^44^ (analysis 2, Fig. 1) as two geological constraints. Although these two calibration points enforce maximum ages for these islands, they are the best source of calibrations currently available for this group. We performed two analyses with two geological calibration points (see above). The analyses were performed under a Speciation: Birth-Death model using an estimated relaxed clock rate (uncorrelated lognormal) since the hypothesis of a strict molecular clock was tested and rejected in PAUP* (p-value < 0.001)^88^. The MCMC parameters were fixed to 100 million generations with sampling every 10.000th generation (20.000 trees) and discarding 4.000 trees as burn-in. In order to reduce the computational time and the parameter space to explore, we fixed the best BI topology from which we removed all outgroups by manually editing the .xml file created in BEAUTi 1.8.1^86^. A 50% majority rule consensus tree was created in TreeAnnotator 1.8.1.

### Ancestral state reconstruction

We used Mesquite^89^ for a Maximum Parsimony approach to infer ancestral areas. This is free of assumptions on likelihood of dispersal and/or vicariance, on distance between areas, and on changing historical landmass configurations. Map figures were made using GeoMapApp v3.4.1^90^ http://www.geomapapp.org and modified in Adobe Photoshop CS2.

### Biogeographical model fitting and ancestral range estimation

We used the R package BioGeoBEARS to test a variety of model-based approaches that incorporate information on clade age, distance and/or connectivity between areas^45^,^91^. BioGeoBEARS requires as inputs (1) a dated phylogeny, which we derived from BEAST and (2) a file of geographic ranges indicating the presence/absence of each species in each area in the analysis. The sampling localities were grouped into the following 12 discrete areas: Bali, Borneo, Flores, E-Java, W-Java, Lombok, Moluccas, New Guinea, Sulawesi, Sumatra, Sumbawa, Philippines (Figs 1 and 2). We allowed for a maximum of three areas at each node, resulting in a total of 298 possible states (geographic ranges) in the state space. We chose to have no more than three areas at a node because most *Trigonopterus* species are micro-endemics. In the analysis, a few nodes were assigned a multi-area range, e.g. the ancestor is hypothesized as having occupied a joint range of New Guinea and W-Java (in clade A of analysis 1). We find this joint range highly unlikely for the same reasons; species of *Trigonopterus* usually occur in very restricted areas, e.g. a single mountain range or a single island and readily speciate, even given small geographic barriers.

A map of the region was produced in GeoMapApp v3.4.1 (http://www.geomapapp.org) and the shape of each area was traced in QGIS software (www.qgis.org). Distances between the areas were defined in QGIS (Fig. 1), with the closest distance between two areas measured in kilometres. These distances were used in the constrained-distance-dependent dispersal matrices (Tables S2–S7). The resulting shape files were imported into R to construct distance and connectivity matrices between regions.

There are three basic models that we used: first, the dispersal extinction cladogenesis (DEC) model of Ree *et al.*^92^ and Ree & Smith^46^; second, the DEC + J model, incorporating founder event speciation^45^,^91^ where ranges can expand to novel areas at cladogenesis; third, DEC + J + x incorporating additionally distance-dependent dispersal. We implemented 12 different variants of these models with two different sets of constraints: (1) the DEC model, unconstrained, (2) DEC + J unconstrained, (3) DEC model with dispersal constrained to adjacent areas, (4) the DEC + J model with dispersal constrained to adjacent areas, (5) DEC model with dispersal constrained to adjacent areas except for the Sunda Arc (W-Java, E-Java, Bali, Lombok, Sumbawa, Flores), (6) the DEC + J model with dispersal constrained to adjacent areas except for the Sunda Arc (W-Java, E-Java, Bali, Lombok, Sumbawa, Flores), (7) DEC + x dispersal is not limited to adjacent areas (unconstrained dispersal) and modified by the exponent *x* describing distance, (8) DEC + J + x dispersal is not limited to adjacent areas (unconstrained dispersal), which adds jump dispersal, also modified by distance *x*. (9) DEC + x model, where dispersal is limited to adjacent areas, (10) DEC + J + x model, where dispersal is limited to adjacent areas, (11) DEC + x model with dispersal constrained to adjacent areas except for the Sunda Arc and (12) DEC + J + x model with dispersal constrained to adjacent areas except for the Sunda Arc (Suppl. Fig. 6).

The “+x” model in BioGeoBEARS takes the distance in kilometres from area A to B and multiplies the rates of dispersal by the exponent of the distance from A to B. For a more thorough explanation of the “+x” models in BioGeoBEARS. Here we used a value of 9,999 km to indicate an unlikely dispersal event in our distance matrix and turned off the constraint matrix in BioGeoBEARS. This configuration would allow dispersal between areas set to a distance of 9,999 km but these dispersal events would be highly unlikely. By turning off the hard constraints, it makes dispersal possible but unlikely given the long dispersal distances. We also rescaled our distance matrix to see if that had any effect on the results. We repeated the analyses with the distances re-scaled to a maximum distance 1, and the others fractions of it, achieving the same results as an input of raw distances in kilometres. We found that rescaling the distance matrix had no effect, and so proceeded with using distance in kilometres.

### Time-stratified analyses

Here we used the 12 models outlined above and additionally we used four separate time intervals for which we measured the distances between areas based on the geological reconstructions of Hall^11^. As our stem node dated to c. 14 Ma we used two longer intervals (15–10 Ma and 10–5 Ma) and two shorter, more recent intervals from 5–2.58 Ma and 2.58 Ma until present. The most recent of these time slices used the modern geological configuration. In the 15–10 Ma time slice, we excluded W-Java, E-Java, Bali, Lombok, Sumbawa and Flores. All areas were allowed in the other time slices. For the manual dispersal probability multipliers we used a value of 0.5 to reflect the uncertainty^11^,^12^,^52^ in the formation of land for Bali, Lombok, Sumbawa and Flores. This was only implemented during the 10–5 Ma time slice. For the distance measurements between areas we used the reconstructions of Hall^11^ except for the 2.58 Ma to modern time slice, where we used the modern configuration. Figures were digitized and converted into the Spherical Mercator projection to take measurements. See supporting information for constraints, distance matrixes and shape files.

### Statistical model choice

The DEC-type models are nested within DEC + J type models; these models could be compared in pairwise fashion using the LRT, a chi-squared test with one degree of freedom (DEC 2 free parameters, DEC + J 3 free parameters). For comparing models on a particular dataset, Akaike information criterion (AIC) and AIC corrected for sample size (AICc) were used. AICc-based weights were calculated and used to calculate relative model probabilities as percentages (Burham & Anderson, 2002)^96^ indicating which model received the most support given the geographic range data.

## Additional Information

**Accession codes:** Sequences have been deposited in EMBL Protein Knowledgebase database under accession numbers HG939627-HG940360, HE615185-HE616067, LM655408-LM656033, LN884315-LN885085, LN880278-LN880449. Data on genetic material contained in this paper is published for non-commercial use only. Utilization for purposes other than non-commercial scientific research may infringe the conditions under which the genetic resources were originally accessed, and should not be undertaken without contacting the corresponding author of the paper and/or seeking permission from the original provider of the genetic material.

**How to cite this article**: Tänzler, R. *et al.* Macroevolution of hyperdiverse flightless beetles reflects the complex geological history of the Sunda Arc. *Sci. Rep.* **6**, 18793; doi: 10.1038/srep18793 (2016).

## Supplementary Material

Supplementary Information S1

Supplementary Information S2

Supplementary Information S3

## Figures and Tables

**Table 1:** Comparison of the different biogeographical models (time-stratified vs. non-time-stratified) with the results of the likelihood under each model, and evaluation with Akaike Information Criterion corrected for sample size (AICc), and the AICc weights and relative model probabilities.

Model Time Stratified	Number of free parameters	LnL results	AICc values	AICc weights	Relative model probabilities, based on AICc
DEC	2	−361.5730074	727.210	6.78645E-57	0%
DEC + J	3	−257.5495221	521.227	3.63279E-12	0%
DEC constraints	2	−368.641341	741.346	5.77969E-60	0%
DEC + J constraints	3	−296.8981293	599.924	2.96045E-29	0%
DEC constraints Lesser Sunda open	2	−356.8097945	717.683	7.9484E-55	0%
DEC + J constraints Lesser Sunda open	3	−260.6508287	527.429	1.63441E-13	0%
DEC + x allDist unconstrained	3	−348.4018278	702.931	1.26936E-51	0%
DEC + J + x allDist unconstrained	4	−233.2309378	474.676	0.046627816	4%
DEC + x constraints	3	−347.2869076	700.701	3.87069E-51	0%
DEC + J + x constraints	4	−239.6466376	487.507	7.62679E-05	0%
DEC + x constraints Lesser Sunda open	3	−337.8727214	681.873	4.74589E-47	0%
DEC + J + x constraints Lesser Sunda open	4	−230.1653798	468.545	1	96%
**Model Non-Time Stratified**	**Number of free parameters**	**LnL results**	**AICc values**	**AICc weights**	**Relative model probabilities, based on AICc**
DEC	2	−357.3507865	718.765	1.57771E-61	0%
DEC + J	3	−252.5532517	511.234	1.83143E-16	0%
DEC constraints	2	−362.479737	729.023	9.34442E-64	0%
DEC + J constraints	3	−292.0505998	590.229	1.2862E-33	0%
DEC constraints Lesser Sunda open	2	−349.6247388	703.313	3.57608E-58	0%
DEC + J constraints Lesser Sunda open	3	−252.0925651	510.313	2.90311E-16	0%
DEC + x allDist unconstrained	3	−337.6891775	681.506	1.94414E-53	0%
DEC + J + x allDist unconstrained	4	−219.0452023	446.304	0.023021246	2%
DEC + x constraints	3	−339.3845311	684.897	3.56816E-54	0%
DEC + J + x constraints	4	−230.6067985	469.428	2.19276E-07	0%
DEC + x constraints Lesser Sunda open	3	−335.597475	677.323	1.5745E-52	0%
DEC + J + x constraints Lesser Sunda open	4	−215.2738645	438.762	1	98%
