# Supplementary material for: Macroevolution of hyperdiverse flightless beetles reflects the complex geological history of the Sunda Arc
Source: Sci Rep. 2016 Jan 8;6:18793. doi: 10.1038/srep18793 (PMC4732383; doi:10.1038/srep18793)
Supplement: Supplementary Information S1 [file srep18793-s1.pdf]

Appendix S1 TABLE OF CONTENT .... Page

Phylogenetic relationships of *Trigonopterus* weevils and historical biogeography inferred from parsimony optimization 2

Historical biogeography of *Trigonopterus* weevils inferred from model-based analysis, dated using the emergence of New Guinea at 30Ma 3

Phylogenetic relationships of *Trigonopterus* weevils. Inferred by ML reconstruction as implemented in IQTree 4

Beast tree with 95% confidence intervals 5

BioGeoBEARS constraints on biogeographical reconstructions on time-stratified

DEC & DEC+J areas allowed 6

DEC & DEC+J unconstrained dispersal 7

DEC & DEC+J with dispersal constrained to adjacent areas 8

DEC & DEC+J with dispersal constrained to adjacent areas except for the

Sunda Arc (Java-E, Java-W, Bali, Lombok, Sumbawa and Flores) 9

DEC+x & DEC+J+x unconstrained dispersal 10

DEC+x & DEC+J+x with dispersal is limited to adjacent areas 11

DEC+x & DEC+J+x with dispersal constrained to adjacent areas except for the Sunda Arc 12

BioGeoBEARS results table 13

Likelihood ratio test results between pairs of models and AIC ratio weights table 14

Table of primers and PCR programs used in this study. D= Denaturation, A= Annealing, E= Elongation 15

Partitioning strategies as used for the Bayesian and maximum likelihood inferences 16

Overview of the species used in this study, collecting locations, and gene fragments used in the dataset 17–19

Figure S1

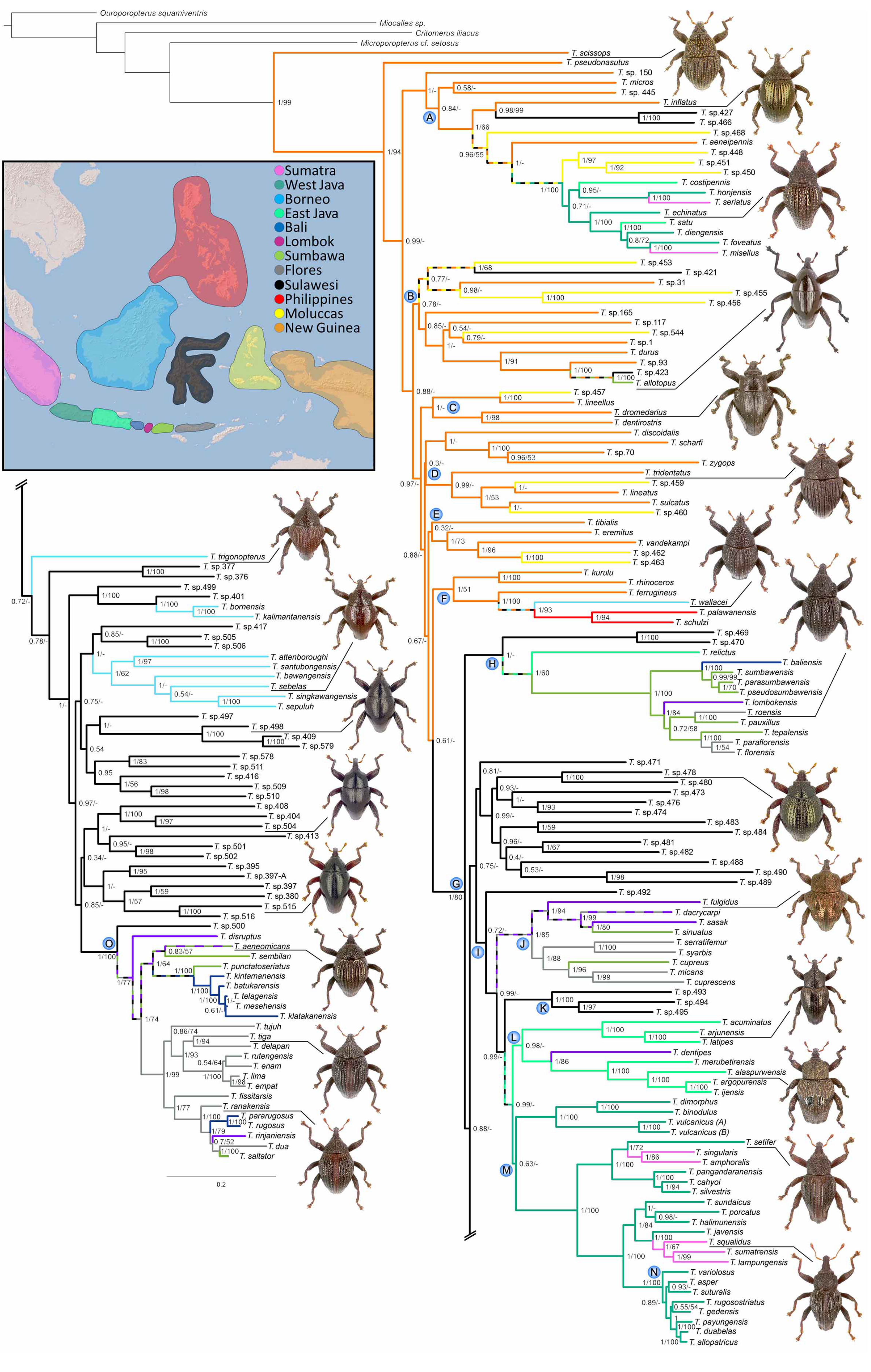

Phylogenetic relationships of *Trigonopterus* weevils and historical biogeography inferred from parsimony optimization. Values at each node (a/b) are a) posterior probability of BI analysis and b) bootstrap support value of the parsimony analysis using TNT; a hyphen indicates absence of this node in the parsimony based topology. Branch colors correspond to the coding of areas in the inset map. Dashed lines indicate ambiguous ancestral areas, respectively species occurring in more than one area. Nodes referred to in the text are marked by letters A to O. Photographs of *Trigonopterus* species earlier published by Riedel A, Tänzler R, Balke M, Rahmadi C, Suhardjono YR (2014) Ninety-eight new species of *Trigonopterus* weevils from Sundaland and the Lesser Sunda Islands. ZooKeys 467: 1–162. and: Riedel A., Sagata K., Surbakti S., Tänzler R., Balke M. 2013. One hundred and one new species of *Trigonopterus* weevils from New Guinea. ZooKeys 280: 1–150.

Figure S2

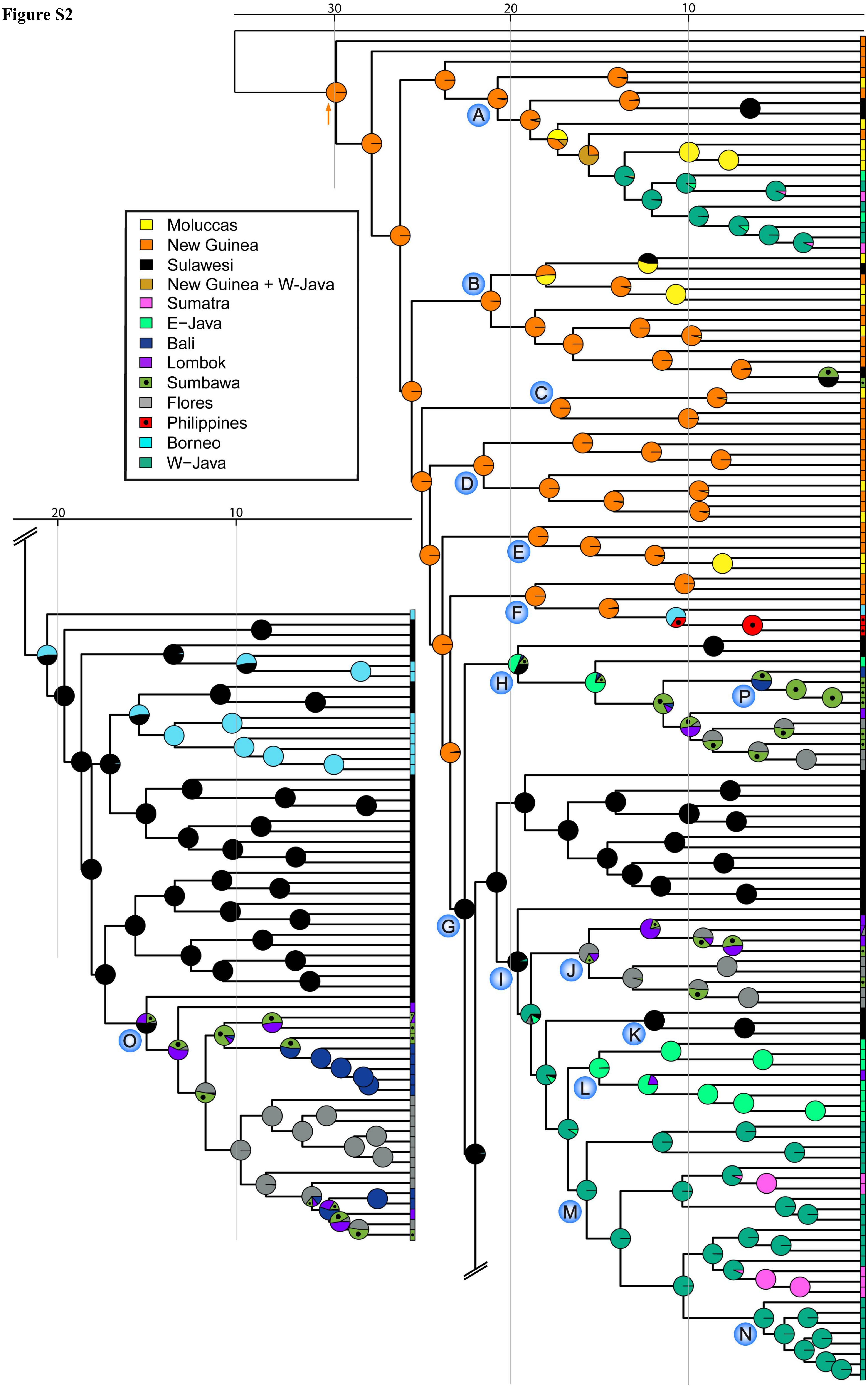

Historical biogeography of *Trigonopterus* weevils inferred from model-based analysis. The Bayesian phylogeny was dated using the emergence of New Guinea at 30 Ma (analysis 1). The chronogram is presenting the median divergence time estimates resulting from the BEAST analysis. Ancestral areas were inferred using a DEC+J+x model. The distribution of each taxon is given in a geographical matrix on the right side of the chronogram with colours as coded in the inset. Coloured pie-charts indicate the likelihood of ancestral areas as recovered at each node. Nodes referred to in the text are marked by letters A to O.

Figure S3

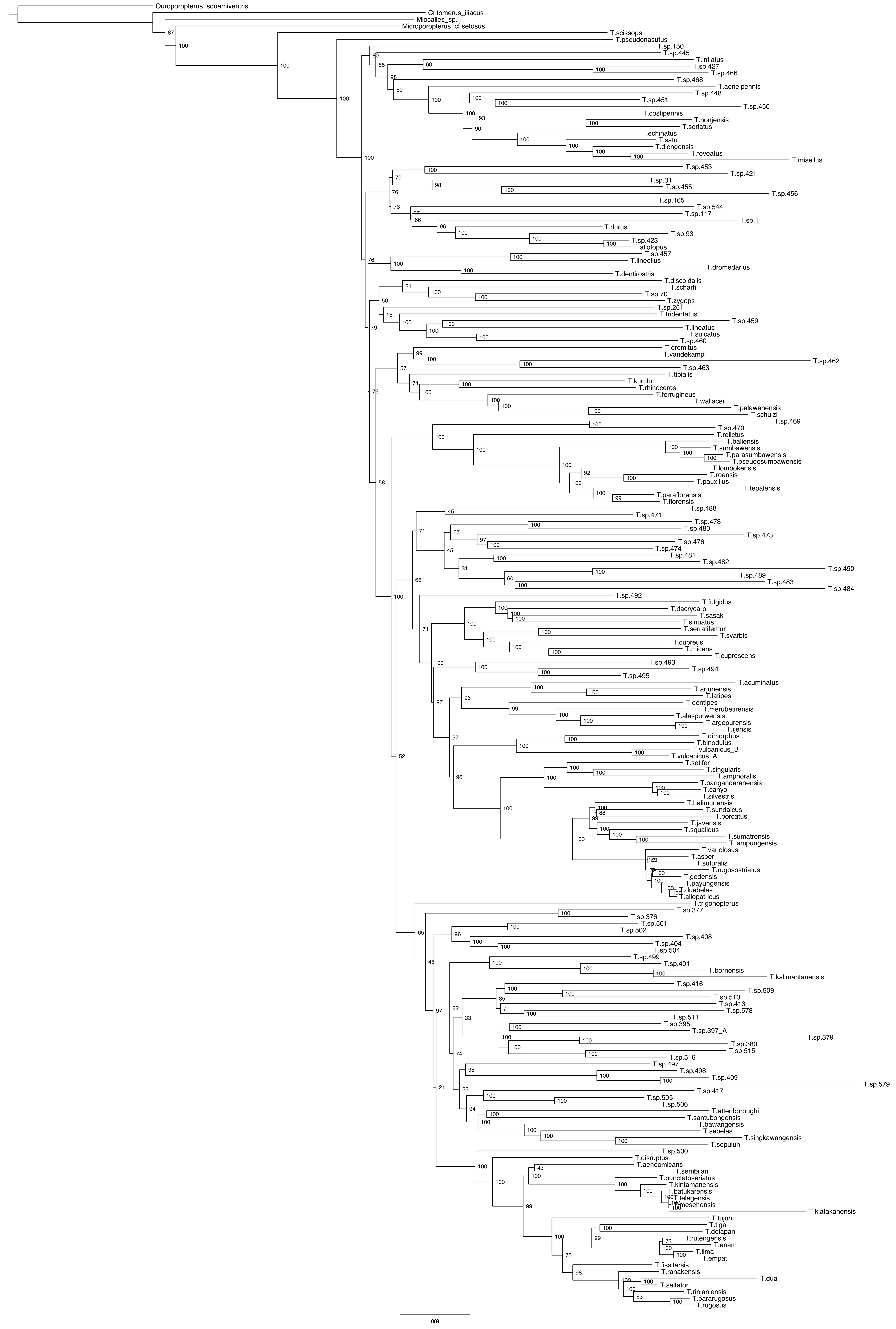

Ouroporopterus_squamiventris
Critomerus_iliacus
Miocalles_sp.
Microporopterus_cf.setosus
T.scissops
T.pseudonasutus
T.sp.150
T.sp.445
T.inflatus
T.sp.427
T.sp.466
T.sp.468
T.aeneipennis
T.sp.448
T.sp.451
T.sp.450
T.costipennis
T.honjensis
T.seriatus
T.echinatus
T.satu
T.diengensis
T.foveatus
T.misellus
T.sp.453
T.sp.421
T.sp.31
T.sp.455
T.sp.456
T.sp.165
T.sp.544
T.sp.117
T.sp.1
T.durus
T.sp.93
T.sp.423
T.allotopus
T.sp.457
T.lineellus
T.dromedarius
T.dentirostris
T.discoidalis
T.scharfi
T.sp.70
T.zygops
T.sp.251
T.tridentatus
T.sp.459
T.lineatus
T.sulcatus
T.sp.460
T.eremitus
T.vandekampi
T.sp.462
T.sp.463
T.tibialis
T.kurulu
T.rhinoceros
T.ferrugineus
T.wallacei
T.palawanensis
T.schulzi
T.sp.469
T.sp.470
T.relictus
T.baliensis
T.sumbawensis
T.parasumbawensis
T.pseudosumbawensis
T.lombokensis
T.roensis
T.pauxillus
T.tepalensis
T.paraflorensis
T.florensis
T.sp.488
T.sp.471
T.sp.478
T.sp.480
T.sp.473
T.sp.476
T.sp.474
T.sp.481
T.sp.482
T.sp.490
T.sp.489
T.sp.483
T.sp.484
T.sp.492
T.fulgidus
T.dacrycarpi
T.sasak
T.sinuatus
T.serratifemur
T.syarbis
T.cupreus
T.micans
T.cuprescens
T.sp.493
T.sp.494
T.sp.495
T.acuminatus
T.arjunensis
T.latipes
T.dentipes
T.merubetirensis
T.alaspurwensis
T.argopurensis
T.ijensis
T.dimorphus
T.binodulus
T.vulcanicus_B
T.vulcanicus_A
T.setifer
T.singularis
T.amphoralis
T.pangandaranensis
T.cahyoi
T.silvestris
T.halimunensis
T.sundaicus
T.porcatus
T.javensis
T.squalidus
T.sumatrensis
T.lampungensis
T.variolosus
T.asper
T.suturalis
T.rugosostriatus
T.gedensis
T.payungensis
T.duabelas
T.allopatricus
T.trigonopterus
T.sp.377
T.sp.376
T.sp.501
T.sp.502
T.sp.408
T.sp.404
T.sp.504
T.sp.499
T.sp.401
T.bornensis
T.kalimantanensis
T.sp.416
T.sp.509
T.sp.510
T.sp.413
T.sp.578
T.sp.511
T.sp.395
T.sp.397_A
T.sp.379
T.sp.380
T.sp.515
T.sp.516
T.sp.497
T.sp.498
T.sp.409
T.sp.579
T.sp.417
T.sp.505
T.sp.506
T.attenboroughi
T.santubongensis
T.bawangensis
T.sebelas
T.singkawangensis
T.sepuluh
T.sp.500
T.disruptus
T.aeneomicans
T.sembilan
T.punctatoseriatus
T.kintamanensis
T.batukarensis
T.telagensis
T.mesehensis
T.klatakanensis
T.tujuh
T.tiga
T.delapan
T.rutengensis
T.enam
T.lima
T.empat
T.fissitarsis
T.ranakensis
T.dua
T.saltator
T.rinjaniensis
T.pararugosus
T.rugosus

Figure S4

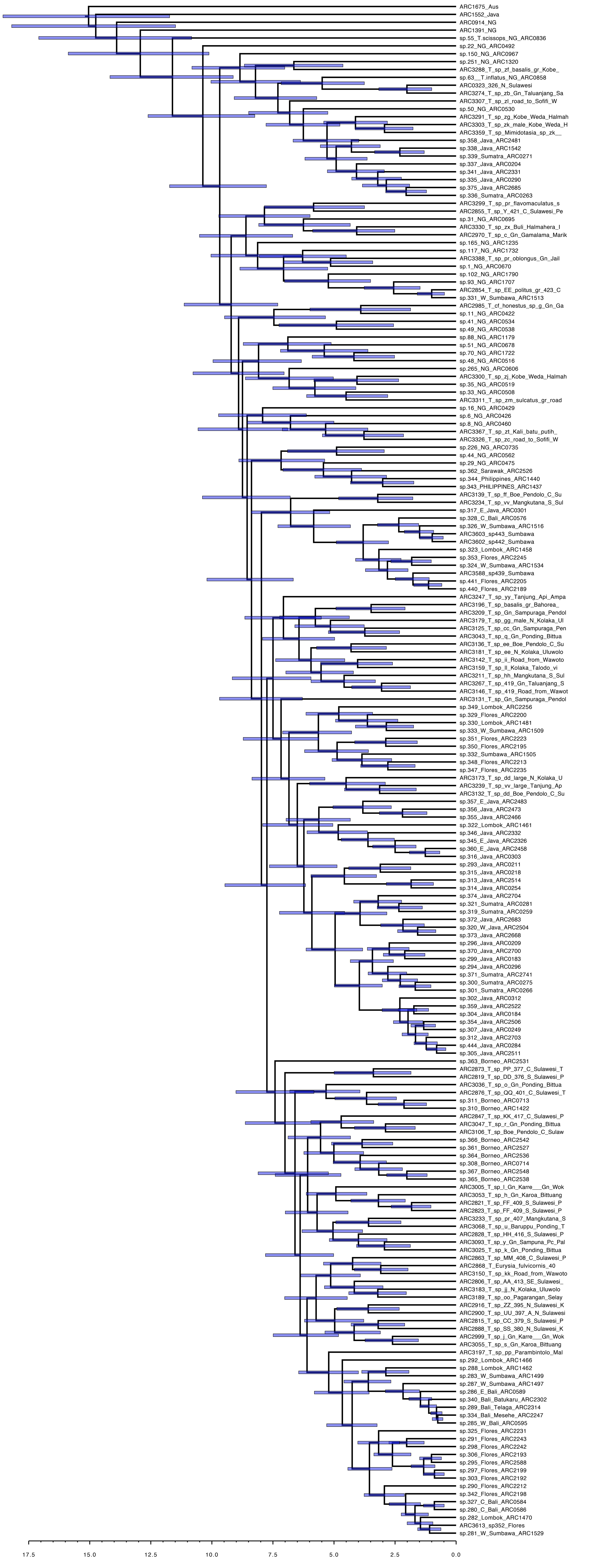
ARC1675_Aus
ARC1552_Java
ARC0914_NG
ARC1391_NG
sp.55_T.scissops_NG_ARC0836
sp.22_NG_ARC0492
sp.150_NG_ARC0967
sp.251_NG_ARC1320
ARC3288_T_sp_zf_basalis_gr_Kobe_
sp.63__T.inflatus_NG_ARC0858
ARC0323_326_N_Sulawesi
ARC3274_T_sp_zb_Gn_Taluanjang_Sa
ARC3307_T_sp_zl_road_to_Sofifi_W
sp.50_NG_ARC0530
ARC3291_T_sp_zg_Kobe_Weda_Halmah
ARC3303_T_sp_zk_male_Kobe_Weda_H
ARC3359_T_sp_Mimidotasia_sp_zk__
sp.358_Java_ARC2481
sp.338_Java_ARC1542
sp.339_Sumatra_ARC0271
sp.337_Java_ARC0204
sp.341_Java_ARC2331
sp.335_Java_ARC0290
sp.375_Java_ARC2685
sp.336_Sumatra_ARC0263
ARC3299_T_sp_pr_flavomaculatus_s
ARC2855_T_sp_Y_421_C_Sulawesi_Pe
sp.31_NG_ARC0695
ARC3330_T_sp_zx_Buli_Halmahera_I
ARC2970_T_sp_c_Gn_Gamalama_Marik
sp.165_NG_ARC1235
sp.117_NG_ARC1732
ARC3388_T_sp_pr_oblongus_Gn_Jail
sp.1_NG_ARC0670
sp.102_NG_ARC1790
sp.93_NG_ARC1707
ARC2854_T_sp_EE_politus_gr_423_C
sp.331_W_Sumbawa_ARC1513
ARC2985_T_cf_honestus_sp_g_Gn_Ga
sp.11_NG_ARC0422
sp.41_NG_ARC0534
sp.49_NG_ARC0538
sp.88_NG_ARC1179
sp.51_NG_ARC0678
sp.70_NG_ARC1722
sp.48_NG_ARC0516
sp.265_NG_ARC0606
ARC3300_T_sp_zj_Kobe_Weda_Halmah
sp.35_NG_ARC0519
sp.33_NG_ARC0508
ARC3311_T_sp_zm_sulcatus_gr_road
sp.16_NG_ARC0429
sp.6_NG_ARC0426
sp.8_NG_ARC0460
ARC3367_T_sp_zt_Kali_batu_putih_
ARC3326_T_sp_zc_road_to_Sofifi_W
sp.226_NG_ARC0735
sp.44_NG_ARC0562
sp.29_NG_ARC0475
sp.362_Sarawak_ARC2526
sp.344_Philippines_ARC1440
sp.343_PHILIPPINES_ARC1437
ARC3139_T_sp_ff_Boe_Pendolo_C_Su
ARC3234_T_sp_vv_Mangkutana_S_Sul
sp.317_E_Java_ARC0301
sp.328_C_Bali_ARC0576
sp.326_W_Sumbawa_ARC1516
ARC3603_sp443_Sumbawa
ARC3602_sp442_Sumbawa
sp.323_Lombok_ARC1458
sp.353_Flores_ARC2245
sp.324_W_Sumbawa_ARC1534
ARC3588_sp439_Sumbawa
sp.441_Flores_ARC2205
sp.440_Flores_ARC2189
ARC3247_T_sp_yy_Tanjung_Api_Ampa
ARC3196_T_sp_basalis_gr_Bahorea_
ARC3209_T_sp_Gn_Sampuraga_Pendol
ARC3179_T_sp_gg_male_N_Kolaka_Ul
ARC3125_T_sp_cc_Gn_Sampuraga_Pen
ARC3043_T_sp_q_Gn_Ponding_Bittua
ARC3136_T_sp_ee_Boe_Pendolo_C_Su
ARC3181_T_sp_ee_N_Kolaka_Uluwolo
ARC3142_T_sp_ii_Road_from_Wawoto
ARC3159_T_sp_ll_Kolaka_Talodo_vi
ARC3211_T_sp_hh_Mangkutana_S_Sul
ARC3267_T_sp_419_Gn_Taluanjang_S
ARC3146_T_sp_419_Road_from_Wawot
ARC3131_T_sp_Gn_Sampuraga_Pendol
sp.349_Lombok_ARC2256
sp.329_Flores_ARC2200
sp.330_Lombok_ARC1481
sp.333_W_Sumbawa_ARC1509
sp.351_Flores_ARC2223
sp.350_Flores_ARC2195
sp.332_Sumbawa_ARC1505
sp.348_Flores_ARC2213
sp.347_Flores_ARC2235
ARC3173_T_sp_dd_large_N_Kolaka_U
ARC3239_T_sp_vv_large_Tanjung_Ap
ARC3132_T_sp_dd_Boe_Pendolo_C_Su
sp.357_E_Java_ARC2483
sp.356_Java_ARC2473
sp.355_Java_ARC2466
sp.322_Lombok_ARC1461
sp.346_Java_ARC2332
sp.345_E_Java_ARC2326
sp.360_E_Java_ARC2458
sp.316_Java_ARC0303
sp.293_Java_ARC0211
sp.315_Java_ARC0218
sp.313_Java_ARC2514
sp.314_Java_ARC0254
sp.374_Java_ARC2704
sp.321_Sumatra_ARC0281
sp.319_Sumatra_ARC0259
sp.372_Java_ARC2683
sp.320_W_Java_ARC2504
sp.373_Java_ARC2668
sp.296_Java_ARC0209
sp.370_Java_ARC2700
sp.299_Java_ARC0183
sp.294_Java_ARC0296
sp.371_Sumatra_ARC2741
sp.300_Sumatra_ARC0275
sp.301_Sumatra_ARC0266
sp.302_Java_ARC0312
sp.359_Java_ARC2522
sp.304_Java_ARC0184
sp.354_Java_ARC2506
sp.307_Java_ARC0249
sp.312_Java_ARC2703
sp.444_Java_ARC0284
sp.305_Java_ARC2511
sp.363_Borneo_ARC2531
ARC2873_T_sp_PP_377_C_Sulawesi_T
ARC2819_T_sp_DD_376_S_Sulawesi_P
ARC3036_T_sp_o_Gn_Ponding_Bittua
ARC2876_T_sp_QQ_401_C_Sulawesi_T
sp.311_Borneo_ARC0713
sp.310_Borneo_ARC1422
ARC2847_T_sp_KK_417_C_Sulawesi_P
ARC3047_T_sp_r_Gn_Ponding_Bittua
ARC3106_T_sp_Boe_Pendolo_C_Sulaw
sp.366_Borneo_ARC2542
sp.361_Borneo_ARC2527
sp.364_Borneo_ARC2536
sp.308_Borneo_ARC0714
sp.367_Borneo_ARC2548
sp.365_Borneo_ARC2538
ARC3005_T_sp_l_Gn_Karre___Gn_Wok
ARC3053_T_sp_h_Gn_Karoa_Bittuang
ARC2821_T_sp_FF_409_S_Sulawesi_P
ARC2823_T_sp_FF_409_S_Sulawesi_P
ARC3233_T_sp_pr_407_Mangkutana_S
ARC3068_T_sp_u_Baruppu_Ponding_T
ARC2828_T_sp_HH_416_S_Sulawesi_P
ARC3093_T_sp_y_Gn_Sampuna_Pc_Pal
ARC3025_T_sp_k_Gn_Ponding_Bittua
ARC2863_T_sp_MM_408_C_Sulawesi_P
ARC2868_T_Eurysia_fulvicornis_40
ARC3150_T_sp_kk_Road_from_Wawoto
ARC2806_T_sp_AA_413_SE_Sulawesi_
ARC3183_T_sp_jj_N_Kolaka_Uluwolo
ARC3189_T_sp_oo_Pagarangan_Selay
ARC2916_T_sp_ZZ_395_N_Sulawesi_K
ARC2900_T_sp_UU_397_A_N_Sulawesi
ARC2815_T_sp_CC_379_S_Sulawesi_P
ARC2888_T_sp_SS_380_N_Sulawesi_K
ARC2999_T_sp_j_Gn_Karre___Gn_Wok
ARC3055_T_sp_s_Gn_Karoa_Bittuang
ARC3197_T_sp_pp_Parambintolo_Mal
sp.292_Lombok_ARC1466
sp.288_Lombok_ARC1462
sp.283_W_Sumbawa_ARC1499
sp.287_W_Sumbawa_ARC1497
sp.286_E_Bali_ARC0589
sp.340_Bali_Batukaru_ARC2302
sp.289_Bali_Telaga_ARC2314
sp.334_Bali_Mesehe_ARC2247
sp.285_W_Bali_ARC0595
sp.325_Flores_ARC2231
sp.291_Flores_ARC2243
sp.298_Flores_ARC2242
sp.306_Flores_ARC2193
sp.295_Flores_ARC2588
sp.297_Flores_ARC2199
sp.303_Flores_ARC2192
sp.290_Flores_ARC2212
sp.342_Flores_ARC2198
sp.327_C_Bali_ARC0584
sp.280_C_Bali_ARC0586
sp.282_Lombok_ARC1470
ARC3613_sp352_Flores
sp.281_W_Sumbawa_ARC1529
17.5
15.0
12.5
10.0
7.5
5.0
2.5
0.0

Table S1 Areas allowed

Maluku	New Guinea	Sulawesi	Sumatra	E-Java	Bali	Lombok	Sumbawa	Flores	Philippines	Borneo	W-Java
1	1	1	1	1	1	1	1	1	1	1	1
1	1	1	1	1	1	1	1	1	1	1	1
1	1	1	1	1	1	1	1	1	1	1	1
1	1	1	1	1	1	1	1	1	1	1	1
1	1	1	1	1	1	1	1	1	1	1	1
1	1	1	1	1	1	1	1	1	1	1	1
1	1	1	1	1	1	1	1	1	1	1	1
1	1	1	1	1	1	1	1	1	1	1	1
1	1	1	1	1	1	1	1	1	1	1	1
1	1	1	1	1	1	1	1	1	1	1	1
1	1	1	1	1	1	1	1	1	1	1	1
1	1	1	1	1	1	1	1	1	1	1	1

Maluku	New Guinea	Sulawesi	Sumatra	E-Java	Bali	Lombok	Sumbawa	Flores	Philippines	Borneo	W-Java
1	1	1	1	1	1	1	1	1	1	1	1
1	1	1	1	1	1	1	1	1	1	1	1
1	1	1	1	1	1	1	1	1	1	1	1
1	1	1	1	1	1	1	1	1	1	1	1
1	1	1	1	1	1	1	1	1	1	1	1
1	1	1	1	1	1	1	1	1	1	1	1
1	1	1	1	1	1	1	1	1	1	1	1
1	1	1	1	1	1	1	1	1	1	1	1
1	1	1	1	1	1	1	1	1	1	1	1
1	1	1	1	1	1	1	1	1	1	1	1
1	1	1	1	1	1	1	1	1	1	1	1
1	1	1	1	1	1	1	1	1	1	1	1

Maluku	New Guinea	Sulawesi	Sumatra	E-Java	Bali	Lombok	Sumbawa	Flores	Philippines	Borneo	W-Java
1	1	1	1	1	1	1	1	1	1	1	1
1	1	1	1	1	1	1	1	1	1	1	1
1	1	1	1	1	1	1	1	1	1	1	1
1	1	1	1	1	1	1	1	1	1	1	1
1	1	1	1	1	1	1	1	1	1	1	1
1	1	1	1	1	1	1	1	1	1	1	1
1	1	1	1	1	1	1	1	1	1	1	1
1	1	1	1	1	1	1	1	1	1	1	1
1	1	1	1	1	1	1	1	1	1	1	1
1	1	1	1	1	1	1	1	1	1	1	1
1	1	1	1	1	1	1	1	1	1	1	1
1	1	1	1	1	1	1	1	1	1	1	1

Maluku	New Guinea	Sulawesi	Sumatra	E-Java	Bali	Lombok	Sumbawa	Flores	Philippines	Borneo	W-Java
1	1	1	1	0	0	0	0	0	1	1	0
1	1	1	1	0	0	0	0	0	1	1	0
1	1	1	1	0	0	0	0	0	1	1	0
1	1	1	1	0	0	0	0	0	1	1	0
0	0	0	0	0	0	0	0	0	0	0	0
0	0	0	0	0	0	0	0	0	0	0	0
0	0	0	0	0	0	0	0	0	0	0	0
0	0	0	0	0	0	0	0	0	0	0	0
0	0	0	0	0	0	0	0	0	0	0	0
1	1	1	1	0	0	0	0	0	1	1	0
1	1	1	1	0	0	0	0	0	1	1	0
0	0	0	0	0	0	0	0	0	0	0	0

END

Table S2 Manual dispersal multipliers, dispersal unconstrained

Maluku	New Guinea		Sulawesi		Sumatra	E-Java	Bali	Lombok	Sumbawa	Flores	Philippines	Borneo	W-Java
1	1	1	1	1	1	1	1	1	1	1	1		
1	1	1	1	1	1	1	1	1	1	1	1		
1	1	1	1	1	1	1	1	1	1	1	1		
1	1	1	1	1	1	1	1	1	1	1	1		
1	1	1	1	1	1	1	1	1	1	1	1		
1	1	1	1	1	1	1	1	1	1	1	1		
1	1	1	1	1	1	1	1	1	1	1	1		
1	1	1	1	1	1	1	1	1	1	1	1		
1	1	1	1	1	1	1	1	1	1	1	1		
1	1	1	1	1	1	1	1	1	1	1	1		
1	1	1	1	1	1	1	1	1	1	1	1		
1	1	1	1	1	1	1	1	1	1	1	1		

Maluku	New Guinea		Sulawesi		Sumatra	E-Java	Bali	Lombok	Sumbawa	Flores	Philippines	Borneo	W-Java
1	1	1	1	1	1	1	1	1	1	1	1		
1	1	1	1	1	1	1	1	1	1	1	1		
1	1	1	1	1	1	1	1	1	1	1	1		
1	1	1	1	1	1	1	1	1	1	1	1		
1	1	1	1	1	1	1	1	1	1	1	1		
1	1	1	1	1	1	1	1	1	1	1	1		
1	1	1	1	1	1	1	1	1	1	1	1		
1	1	1	1	1	1	1	1	1	1	1	1		
1	1	1	1	1	1	1	1	1	1	1	1		
1	1	1	1	1	1	1	1	1	1	1	1		
1	1	1	1	1	1	1	1	1	1	1	1		
1	1	1	1	1	1	1	1	1	1	1	1		

Maluku	New Guinea		Sulawesi		Sumatra	E-Java	Bali	Lombok	Sumbawa	Flores	Philippines	Borneo	W-Java
1	1	1	1	1	0.5	0.5	0.5	0.5	1	1	1		
1	1	1	1	1	0.5	0.5	0.5	0.5	1	1	1		
1	1	1	1	1	0.5	0.5	0.5	0.5	1	1	1		
1	1	1	1	1	0.5	0.5	0.5	0.5	1	1	1		
1	1	1	1	1	0.5	0.5	0.5	0.5	1	1	1		
0.5	0.5	0.5	0.5	0.5	0.5	0.5	0.5	0.5	0.5	0.5	0.5		
0.5	0.5	0.5	0.5	0.5	0.5	0.5	0.5	0.5	0.5	0.5	0.5		
0.5	0.5	0.5	0.5	0.5	0.5	0.5	0.5	0.5	0.5	0.5	0.5		
0.5	0.5	0.5	0.5	0.5	0.5	0.5	0.5	0.5	0.5	0.5	0.5		
1	1	1	1	1	0.5	0.5	0.5	0.5	1	1	1		
1	1	1	1	1	0.5	0.5	0.5	0.5	1	1	1		
1	1	1	1	1	0.5	0.5	0.5	0.5	1	1	1		

Maluku	New Guinea		Sulawesi		Sumatra	E-Java	Bali	Lombok	Sumbawa	Flores	Philippines	Borneo	W-Java
1	1	1	1	0	0	0	0	0	1	1	0		
1	1	1	1	0	0	0	0	0	1	1	0		
1	1	1	1	0	0	0	0	0	1	1	0		
1	1	1	1	0	0	0	0	0	1	1	0		
0	0	0	0	0	0	0	0	0	0	0	0		
0	0	0	0	0	0	0	0	0	0	0	0		
0	0	0	0	0	0	0	0	0	0	0	0		
0	0	0	0	0	0	0	0	0	0	0	0		
0	0	0	0	0	0	0	0	0	0	0	0		
1	1	1	1	0	0	0	0	0	1	1	0		
1	1	1	1	0	0	0	0	0	1	1	0		
0	0	0	0	0	0	0	0	0	0	0	0		

END

Table S3 Manual dispersal multipliers, dispersal constrained to adjacent areas

Maluku	New Guinea		Sulawesi		Sumatra	E-Java	Bali	Lombok	Sumbawa	Flores	Philippines	Borneo	W-Java
1	1	1	0	0	0	0	0	1	1	1	0		
1	1	1	0	0	0	0	1	1	1	1	0		
1	1	1	0	1	1	1	1	1	1	1	1		
0	0	0	1	0	0	0	0	0	0	1	1		
0	0	1	0	1	1	0	0	0	0	1	1		
0	0	1	0	1	1	1	0	0	0	1	0		
0	0	1	0	0	1	1	1	0	0	1	0		
0	1	1	0	0	0	1	1	1	0	0	0		
1	1	1	0	0	0	0	1	1	0	1	0		
1	1	1	0	0	0	0	0	0	1	1	0		
1	1	1	1	1	1	1	0	1	1	1	1		
0	0	1	1	1	0	0	0	0	0	1	1		

Maluku	New Guinea		Sulawesi		Sumatra	E-Java	Bali	Lombok	Sumbawa	Flores	Philippines	Borneo	W-Java
1	1	1	0	0	0	0	0	1	1	1	0		
1	1	1	0	0	0	0	1	1	1	1	0		
1	1	1	0	1	1	1	1	1	1	1	1		
0	0	0	1	0	0	0	0	0	0	1	1		
0	0	1	0	1	1	0	0	0	0	1	1		
0	0	1	0	1	1	1	0	0	0	1	0		
0	0	1	0	0	1	1	1	0	0	1	0		
0	1	1	0	0	0	1	1	1	0	0	0		
1	1	1	0	0	0	0	1	1	0	1	0		
1	1	1	0	0	0	0	0	0	1	1	0		
1	1	1	1	1	1	1	0	1	1	1	1		
0	0	1	1	1	0	0	0	0	0	1	1		

Maluku	New Guinea		Sulawesi		Sumatra	E-Java	Bali	Lombok	Sumbawa	Flores	Philippines	Borneo	W-Java
1	1	1	0	0	0	0	0	0.5	1	1	0		
1	1	1	0	0	0	0	0.5	0.5	1	1	0		
1	1	1	0	1	0.5	0.5	0.5	0.5	1	1	1		
0	0	0	1	0	0	0	0	0	0	1	1		
0	0	1	0	1	0.5	0	0	0	0	1	1		
0	0	0.5	0	0.5	0.5	0.5	0	0	0	0.5	0		
0	0	0.5	0	0	0.5	0.5	0.5	0	0	0.5	0		
0	0.5	0.5	0	0	0	0.5	0.5	0.5	0	0	0		
0.5	0.5	0.5	0	0	0	0	0.5	0.5	0	0.5	0		
1	1	1	0	0	0	0	0	0	1	1	0		
1	1	1	1	1	0.5	0.5	0	0.5	1	1	1		
0	0	1	1	1	0	0	0	0	0	1	1		

Maluku	New Guinea		Sulawesi		Sumatra	E-Java	Bali	Lombok	Sumbawa	Flores	Philippines	Borneo	W-Java
1	1	1	0	0	0	0	0	0	1	1	0		
1	1	1	0	0	0	0	0	0	1	1	0		
1	1	1	0	0	0	0	0	0	1	1	0		
0	0	0	1	0	0	0	0	0	0	1	0		
0	0	0	0	0	0	0	0	0	0	0	0		
0	0	0	0	0	0	0	0	0	0	0	0		
0	0	0	0	0	0	0	0	0	0	0	0		
0	0	0	0	0	0	0	0	0	0	0	0		
0	0	0	0	0	0	0	0	0	0	0	0		
1	1	1	0	0	0	0	0	0	1	1	0		
1	1	1	1	0	0	0	0	0	1	1	0		
0	0	0	0	0	0	0	0	0	0	0	0		

END

Table S4 Manual dispersal multipliers, dispersal constrained to adjacent areas except for the Sunda Arc

Maluku	New Guinea		Sulawesi		Sumatra	E-Java	Bali	Lombok	Sumbawa	Flores	Philippines	Borneo	W-Java
1	1	1	0	0	0	0	0	1	1	1	0		
1	1	1	0	0	0	0	1	1	1	1	0		
1	1	1	0	1	1	1	1	1	1	1	1		
0	0	0	1	0	0	0	0	0	0	1	1		
0	0	1	0	1	1	1	1	1	0	1	1		
0	0	1	0	1	1	1	1	1	0	1	1		
0	0	1	0	1	1	1	1	1	0	1	1		
0	1	1	0	1	1	1	1	1	0	0	1		
1	1	1	0	1	1	1	1	1	0	1	1		
1	1	1	0	0	0	0	0	0	1	1	0		
1	1	1	1	1	1	1	0	1	1	1	1		
0	0	1	1	1	1	1	1	1	0	1	1		

Maluku	New Guinea		Sulawesi		Sumatra	E-Java	Bali	Lombok	Sumbawa	Flores	Philippines	Borneo	W-Java
1	1	1	0	0	0	0	0	1	1	1	0		
1	1	1	0	0	0	0	1	1	1	1	0		
1	1	1	0	1	1	1	1	1	1	1	1		
0	0	0	1	0	0	0	0	0	0	1	1		
0	0	1	0	1	1	1	1	1	0	1	1		
0	0	1	0	1	1	1	1	1	0	1	1		
0	0	1	0	1	1	1	1	1	0	1	1		
0	1	1	0	1	1	1	1	1	0	0	1		
1	1	1	0	1	1	1	1	1	0	1	1		
1	1	1	0	0	0	0	0	0	1	1	0		
1	1	1	1	1	1	1	0	1	1	1	1		
0	0	1	1	1	1	1	1	1	0	1	1		

Maluku	New Guinea		Sulawesi		Sumatra	E-Java	Bali	Lombok	Sumbawa	Flores	Philippines	Borneo	W-Java
1	1	1	0	0	0	0	0	0.5	1	1	0		
1	1	1	0	0	0	0	0.5	0.5	1	1	0		
1	1	1	0	1	0.5	0.5	0.5	0.5	1	1	1		
0	0	0	1	0	0	0	0	0	0	1	1		
0	0	1	0	0.5	0.5	0.5	0.5	0.5	0	1	1		
0	0	0.5	0	0.5	0.5	0.5	0.5	0.5	0	0.5	0.5		
0	0	0.5	0	0.5	0.5	0.5	0.5	0.5	0	0.5	0.5		
0	0.5	0.5	0	0.5	0.5	0.5	0.5	0.5	0	0	0.5		
0.5	0.5	0.5	0	0.5	0.5	0.5	0.5	0.5	0	0.5	0.5		
1	1	1	0	0	0	0	0	0	1	1	0		
1	1	1	1	1	0.5	0.5	0	0.5	1	1	1		
0	0	1	1	1	0.5	0.5	0.5	0.5	0	1	1		

Maluku	New Guinea		Sulawesi		Sumatra	E-Java	Bali	Lombok	Sumbawa	Flores	Philippines	Borneo	W-Java
1	1	1	0	0	0	0	0	0	1	1	0		
1	1	1	0	0	0	0	0	0	1	1	0		
1	1	1	0	0	0	0	0	0	1	1	0		
0	0	0	1	0	0	0	0	0	0	1	0		
0	0	0	0	0	0	0	0	0	0	0	0		
0	0	0	0	0	0	0	0	0	0	0	0		
0	0	0	0	0	0	0	0	0	0	0	0		
0	0	0	0	0	0	0	0	0	0	0	0		
0	0	0	0	0	0	0	0	0	0	0	0		
1	1	1	0	0	0	0	0	0	1	1	0		
1	1	1	1	0	0	0	0	0	1	1	0		
0	0	0	0	0	0	0	0	0	0	0	0		

END

Table S5 Distances matrix, dispersal unconstrained

Maluku	New Guinea		Sulawesi		Sumatra	E-Java	Bali	Lombok	Sumbawa	Flores	Philippines	Borneo	W-Java
0	215.6	289.4	2432	1667	1553	1461	1244	990	557.7	1090	2030		
215.6	0	819.4	2825	1991	1888	1777	1562	1180	962.9	1487	2380		
289.4	819.4	0	1552	648.6	557	461.9	321.3	314.1	458.6	148.6	1052		
2432	2825	1552	0	561.6	1097	1232	1383	1645	1816	547.1	66.36		
1667	1991	648.6	561.6	0	27.5	202.1	285	608.7	1959	425.2	29.61		
1553	1888	557	1097	27.5	0	58.34	147.6	461.4	1853	475.6	461.9		
1461	1777	461.9	1232	202.1	58.34	0	22.76	343.3	1875	510.7	630.3		
1244	1562	321.3	1383	285	147.6	22.76	0	67.5	1889	571.7	713.2		
990	1180	314.1	1645	608.7	461.4	343.3	67.5	0	1928	689.9	1039		
557.7	962.9	458.6	1816	1959	1853	1875	1889	1928	0	180.7	2180		
1090	1487	148.6	547.1	425.2	475.6	510.7	571.7	689.9	180.7	0	445.8		
2030	2380	1052	66.36	29.61	461.9	630.3	713.2	1039	2180	445.8	0		

Maluku	New Guinea		Sulawesi		Sumatra	E-Java	Bali	Lombok	Sumbawa	Flores	Philippines	Borneo	W-Java
0	366.7	874.6	2879	2069	1986	1935	1717	1457	840.5	1467	2450		
366.7	0	934.2	2976	2043	1966	1872	1640	1295	1162	1599	2483		
874.6	934.2	0	1587	910.1	869.3	776.9	601.2	526.6	809.2	374.6	1296		
2879	2976	1587	0	820.4	1046	1149	1324	1693	2679	317.1	84.61		
2069	2043	910.1	820.4	0	34.36	128.2	339.4	713.9	2139	510.1	262.7		
1986	1966	869.3	1046	34.36	0	81.97	252.6	603.5	2175	547.2	505.5		
1935	1872	776.9	1149	128.2	81.97	0	169.3	505.2	2135	563	582.7		
1717	1640	601.2	1324	339.4	252.6	169.3	0	256.6	1983	613.4	784.2		
1457	1295	526.6	1693	713.9	603.5	505.2	256.6	0	1872	825.6	1163		
840.5	1162	809.2	2679	2139	2175	2135	1983	1872	0	314.3	2361		
1467	1599	374.6	317.1	510.1	547.2	563	613.4	825.6	314.3	0	513.9		
2450	2483	1296	84.61	262.7	505.5	582.7	784.2	1163	2361	513.9	0		

Maluku	New Guinea		Sulawesi		Sumatra	E-Java	Bali	Lombok	Sumbawa	Flores	Philippines	Borneo	W-Java
0	145.4	1142	3064	2148	2023	1954	1761	1573	865.8	1906	2760		
145.4	0	930.2	2815	1899	1767	1688	1531	1326	904	1779	2510		
1142	930.2	0	1593	784.5	673.7	619.8	490.4	414.2	547.4	421.7	1322		
3064	2815	1593	0	699.8	1050	1145	1314	1512	2700	185.4	165.6		
2148	1899	784.5	699.8	0	84.91	210.6	385.1	588.1	1999	512.9	336		
2023	1767	673.7	1050	84.91	0	67.63	251.9	484.7	1898	591.2	709.7		
1954	1688	619.8	1145	210.6	67.63	0	170	373.3	1855	600.2	807.2		
1761	1531	490.4	1314	385.1	251.9	170	0	173.2	1712	671.5	991.7		
1573	1326	414.2	1512	588.1	484.7	373.3	173.2	0	1570	742.2	1251		
865.8	904	547.4	2700	1999	1898	1855	1712	1570	0	275.6	2492		
1906	1779	421.7	185.4	512.9	591.2	600.2	671.5	742.2	275.6	0	346.5		
2760	2510	1322	165.6	336	709.7	807.2	991.7	1251	2492	346.5	0		

Maluku	New Guinea		Sulawesi		Sumatra	E-Java	Bali	Lombok	Sumbawa	Flores	Philippines	Borneo	W-Java
0	116.1	1551	3505	9999	9999	9999	9999	9999	1374	2235	9999		
116.1	0	1537	3515	9999	9999	9999	9999	9999	1498	2276	9999		
1551	1537	0	1668	9999	9999	9999	9999	9999	1071	559.5	9999		
3505	3515	1668	0	9999	9999	9999	9999	9999	2699	559.3	9999		
9999	9999	9999	9999	0	9999	9999	9999	9999	9999	9999	9999		
9999	9999	9999	9999	9999	0	9999	9999	9999	9999	9999	9999		
9999	9999	9999	9999	9999	9999	0	9999	9999	9999	9999	9999		
9999	9999	9999	9999	9999	9999	9999	0	9999	9999	9999	9999		
9999	9999	9999	9999	9999	9999	9999	9999	0	9999	9999	9999		
1374	1498	1071	2699	9999	9999	9999	9999	9999	0	445.5	9999		
2235	2276	559.5	559.3	9999	9999	9999	9999	9999	445.5	0	9999		
9999	9999	9999	9999	9999	9999	9999	9999	9999	9999	9999	0		

END

Table S6 Distances matrix, dispersal constrained to adjacent areas

Maluku	New Guinea	Sulawesi	Sumatra	E-Java	Bali	Lombok	Sumbawa	Flores	Philippines	Borneo	W-Java
0	215.6	289.4	9999	9999	9999	9999	9999	990	557.7	1090	9999
215.6	0	819.4	9999	9999	9999	9999	1562	1180	962.9	1487	9999
289.4	819.4	0	9999	648.6	557	461.9	321.3	314.1	458.6	148.6	1052
9999	9999	9999	0	9999	9999	9999	9999	9999	9999	547.1	66.36
9999	9999	648.6	9999	0	27.5	9999	9999	9999	9999	425.2	29.61
9999	9999	557	9999	27.5	0	58.34	9999	9999	9999	475.6	9999
9999	9999	461.9	9999	9999	58.34	0	22.76	9999	9999	510.7	9999
9999	1562	321.3	9999	9999	9999	22.76	0	67.5	9999	9999	9999
990	1180	314.1	9999	9999	9999	9999	67.5	0	9999	689.9	9999
557.7	962.9	458.6	9999	9999	9999	9999	9999	9999	0	180.7	9999
1090	1487	148.6	547.1	425.2	475.6	510.7	9999	689.9	180.7	0	445.8
9999	9999	1052	66.36	29.61	9999	9999	9999	9999	9999	445.8	0

Maluku	New Guinea	Sulawesi	Sumatra	E-Java	Bali	Lombok	Sumbawa	Flores	Philippines	Borneo	W-Java
0	366.7	874.6	9999	9999	9999	9999	9999	1457	840.5	1467	9999
366.7	0	934.2	9999	9999	9999	9999	1640	1295	1162	1599	9999
874.6	934.2	0	9999	910.1	869.3	776.9	601.2	526.6	809.2	374.6	1296
9999	9999	9999	0	9999	9999	9999	9999	9999	9999	317.1	84.61
9999	9999	910.1	9999	0	34.36	9999	9999	9999	9999	510.1	262.7
9999	9999	869.3	9999	34.36	0	81.97	9999	9999	9999	547.2	9999
9999	9999	776.9	9999	9999	81.97	0	169.3	9999	9999	563	9999
9999	1640	601.2	9999	9999	9999	169.3	0	256.6	9999	613.4	9999
1457	1295	526.6	9999	9999	9999	9999	256.6	0	9999	825.6	9999
840.5	1162	809.2	9999	9999	9999	9999	9999	9999	0	314.3	9999
1467	1599	374.6	317.1	510.1	547.2	563	613.4	825.6	314.3	0	513.9
9999	9999	1296	84.61	262.7	9999	9999	9999	9999	9999	513.9	0

Maluku	New Guinea	Sulawesi	Sumatra	E-Java	Bali	Lombok	Sumbawa	Flores	Philippines	Borneo	W-Java
0	145.4	1142	9999	9999	9999	9999	9999	1573	865.8	1906	9999
145.4	0	930.2	9999	9999	9999	9999	1531	1326	904	1779	9999
1142	930.2	0	9999	784.5	673.7	619.8	490.4	414.2	547.4	421.7	1322
9999	9999	9999	0	9999	9999	9999	9999	9999	9999	185.4	165.6
9999	9999	784.5	9999	0	84.91	9999	9999	9999	9999	512.9	336
9999	9999	673.7	9999	84.91	0	67.63	9999	9999	9999	591.2	9999
9999	9999	619.8	9999	9999	67.63	0	170	9999	9999	600.2	9999
9999	1531	490.4	9999	9999	9999	170	0	173.2	9999	671.5	9999
1573	1326	414.2	9999	9999	9999	9999	173.2	0	9999	742.2	9999
865.8	904	547.4	9999	9999	9999	9999	9999	9999	0	275.6	9999
1906	1779	421.7	185.4	512.9	591.2	600.2	671.5	742.2	275.6	0	346.5
9999	9999	1322	165.6	336	9999	9999	9999	9999	9999	346.5	0

Maluku	New Guinea	Sulawesi	Sumatra	E-Java	Bali	Lombok	Sumbawa	Flores	Philippines	Borneo	W-Java
0	116.1	1551	9999	9999	9999	9999	9999	9999	1374	2235	9999
116.1	0	1537	9999	9999	9999	9999	9999	9999	1498	2276	9999
1551	1537	0	9999	9999	9999	9999	9999	9999	1071	559.5	9999
9999	9999	9999	0	9999	9999	9999	9999	9999	9999	559.3	9999
9999	9999	9999	9999	0	9999	9999	9999	9999	9999	9999	9999
9999	9999	9999	9999	9999	0	9999	9999	9999	9999	9999	9999
9999	9999	9999	9999	9999	9999	0	9999	9999	9999	9999	9999
9999	9999	9999	9999	9999	9999	9999	0	9999	9999	9999	9999
9999	9999	9999	9999	9999	9999	9999	9999	0	9999	9999	9999
1374	1498	1071	9999	9999	9999	9999	9999	9999	0	445.5	9999
2235	2276	559.5	559.3	9999	9999	9999	9999	9999	445.5	0	9999
9999	9999	9999	9999	9999	9999	9999	9999	9999	9999	9999	0

END

Table S7 Distances matrix, dispersal constrained to adjacent areas except for the Sunda Arc

Maluku	New Guinea		Sulawesi		Sumatra	E-Java	Bali	Lombok	Sumbawa	Flores	Philippines	Borneo	W-Java
0	215.6	289.4	9999	9999	9999	9999	9999	990	557.7	1090	9999		
215.6	0	819.4	9999	9999	9999	9999	1562	1180	962.9	1487	9999		
289.4	819.4	0	9999	648.6	557	461.9	321.3	314.1	458.6	148.6	1052		
9999	9999	9999	0	9999	9999	9999	9999	9999	9999	547.1	66.36		
9999	9999	648.6	9999	0	27.5	202.1	285	608.7	9999	425.2	29.61		
9999	9999	557	9999	27.5	0	58.34	147.6	461.4	9999	475.6	461.9		
9999	9999	461.9	9999	202.1	58.34	0	22.76	343.3	9999	510.7	630.3		
9999	1562	321.3	9999	285	147.6	22.76	0	67.5	9999	9999	713.2		
990	1180	314.1	9999	608.7	461.4	343.3	67.5	0	9999	689.9	1039		
557.7	962.9	458.6	9999	9999	9999	9999	9999	9999	0	180.7	9999		
1090	1487	148.6	547.1	425.2	475.6	510.7	9999	689.9	180.7	0	445.8		
9999	9999	1052	66.36	29.61	461.9	630.3	713.2	1039	9999	445.8	0		

Maluku	New Guinea		Sulawesi		Sumatra	E-Java	Bali	Lombok	Sumbawa	Flores	Philippines	Borneo	W-Java
0	366.7	874.6	9999	9999	9999	9999	9999	1457	840.5	1467	9999		
366.7	0	934.2	9999	9999	9999	9999	1640	1295	1162	1599	9999		
874.6	934.2	0	9999	910.1	869.3	776.9	601.2	526.6	809.2	374.6	1296		
9999	9999	9999	0	9999	9999	9999	9999	9999	9999	317.1	84.61		
9999	9999	910.1	9999	0	34.36	128.2	339.4	713.9	9999	510.1	262.7		
9999	9999	869.3	9999	34.36	0	81.97	252.6	603.5	9999	547.2	505.5		
9999	9999	776.9	9999	128.2	81.97	0	169.3	505.2	9999	563	582.7		
9999	1640	601.2	9999	339.4	252.6	169.3	0	256.6	9999	613.4	784.2		
1457	1295	526.6	9999	713.9	603.5	505.2	256.6	0	9999	825.6	1163		
840.5	1162	809.2	9999	9999	9999	9999	9999	9999	0	314.3	9999		
1467	1599	374.6	317.1	510.1	547.2	563	613.4	825.6	314.3	0	513.9		
9999	9999	1296	84.61	262.7	505.5	582.7	784.2	1163	9999	513.9	0		

Maluku	New Guinea		Sulawesi		Sumatra	E-Java	Bali	Lombok	Sumbawa	Flores	Philippines	Borneo	W-Java
0	145.4	1142	9999	9999	9999	9999	9999	1573	865.8	1906	9999		
145.4	0	930.2	9999	9999	9999	9999	1531	1326	904	1779	9999		
1142	930.2	0	9999	784.5	673.7	619.8	490.4	414.2	547.4	421.7	1322		
9999	9999	9999	0	9999	9999	9999	9999	9999	9999	185.4	165.6		
9999	9999	784.5	9999	0	84.91	210.6	385.1	588.1	9999	512.9	336		
9999	9999	673.7	9999	84.91	0	67.63	251.9	484.7	9999	591.2	709.7		
9999	9999	619.8	9999	210.6	67.63	0	170	373.3	9999	600.2	807.2		
9999	1531	490.4	9999	385.1	251.9	170	0	173.2	9999	671.5	991.7		
1573	1326	414.2	9999	588.1	484.7	373.3	173.2	0	9999	742.2	1251		
865.8	904	547.4	9999	9999	9999	9999	9999	9999	0	275.6	9999		
1906	1779	421.7	185.4	512.9	591.2	600.2	671.5	742.2	275.6	0	346.5		
9999	9999	1322	165.6	336	709.7	807.2	991.7	1251	9999	346.5	0		

Maluku	New Guinea		Sulawesi		Sumatra	E-Java	Bali	Lombok	Sumbawa	Flores	Philippines	Borneo	W-Java
0	116.1	1551	9999	9999	9999	9999	9999	9999	1374	2235	9999		
116.1	0	1537	9999	9999	9999	9999	9999	9999	1498	2276	9999		
1551	1537	0	9999	9999	9999	9999	9999	9999	1071	559.5	9999		
9999	9999	9999	0	9999	9999	9999	9999	9999	9999	559.3	9999		
9999	9999	9999	9999	0	9999	9999	9999	9999	9999	9999	9999		
9999	9999	9999	9999	9999	0	9999	9999	9999	9999	9999	9999		
9999	9999	9999	9999	9999	9999	0	9999	9999	9999	9999	9999		
9999	9999	9999	9999	9999	9999	9999	0	9999	9999	9999	9999		
9999	9999	9999	9999	9999	9999	9999	9999	0	9999	9999	9999		
1374	1498	1071	9999	9999	9999	9999	9999	9999	0	445.5	9999		
2235	2276	559.5	559.3	9999	9999	9999	9999	9999	445.5	0	9999		
9999	9999	9999	9999	9999	9999	9999	9999	9999	9999	9999	0		

END

Table S8 BioGeoBEARS results table

MODEL non-time stratified 15Ma	LnL	numparams	d	e	j	x
DEC	-357.3507865	2	0.004588233	0.008465865	0	0
DEC+J	-252.5532517	3	1.00E-15	1.00E-15	0.016142793	0
DEC constraints	-362.479737	2	0.00819725	0.017574331	0	0
DEC+J constraints	-292.0505998	3	0.001095877	0.00502133	0.028321211	0
DEC constraints Lower Sunda open	-349.6247388	2	0.006849648	0.011886564	0	0
DEC+J constraints Lower Sunda open	-252.0925651	3	0.00015386	0.001534244	0.019918057	0
DEC+x allDist unconstrained	-337.6891775	3	0.060388614	0.006537113	0	-0.406852004
DEC+J+x allDist unconstrained	-219.0452023	4	1.00E-15	1.00E-15	0.968138333	-0.763384999
DEC+x constraints	-339.3845311	3	0.057813802	0.006277669	0	-0.400315207
DEC+J+x constraints	-230.6067985	4	1.00E-15	1.00E-15	0.240276704	-0.420505549
DEC+x constraints Sunda Arc	-335.5974		0.048854685	0.011290447	0	-0.384880173
DEC+J+x constraints Lower Sunda open	-215.2738645	4	5.00E-09	1.00E-15	1.734420641	-0.919247154

MODEL time stratified 15Ma	LnL	numparams	d	e	j	x
DEC	-361.5730074	2	0.004848366	0.008808697	0	0
DEC+J	-257.5495221	3	0.00014674	1.00E-15	0.017927619	0
DEC constraints	-368.641341	2	0.008733267	0.020314423	0	0
DEC+J constraints	-296.8981293	3	0.001292323	0.006175104	0.030670049	0
DEC constraints Lower Sunda open	-356.8097945	2	0.007335636	0.01463237	0	0
DEC+J constraints Lower Sunda open	-260.6508287	3	0.000352308	0.002360563	0.024776204	0
DEC+x allDist unconstrained	-348.4018278	3	0.02951805	0.008444624	0	-0.280064083
DEC+J+x allDist unconstrained	-233.2309378	4	0.009088673	1.00E-20	0.978741367	-0.71195548
DEC+x constraints	-347.2869076	3	0.041890168	0.009221709	0	-0.310890206
DEC+J+x constraints	-239.6466376	4	0.007195995	1.00E-20	0.820924938	-0.619404451
DEC+x constraints Lower Sunda open	-337.8727214	3	0.20003603	0.010647834	0	-0.582500046
DEC+J+x constraints Lower Sunda open	-230.1653798	4	0.015308829	1.00E-20	1.284045148	-0.76221697

Table S9 Likelihood ratio test results between pairs of models and AIC ratio weights.

alt	null	LnLalt	LnLnull	DFalt	DFnull	DF	Dstatistic	pval	test	tail	AIC1	AIC2	AICwt1	AICwt2	AICweight_ratio_model1	AICweight_ratio_model2
resDECj	resDEC	-252.6	-357.4	3	2	1	209.6	1.70E-47	chi-squared	one-tailed	511.1	718.7	1	8.30E-46	1.20E+45	8.30E-46
resDECj_constraints	resDEC_constraints	-292.1	-362.5	3	2	1	140.9	1.70E-32	chi-squared	one-tailed	590.1	729	1	7.00E-31	1.42E+30	7.00E-31
resDECj_Lower_Sunda_Open	resDECs_Lower_Sunda_Open	-252.1	-349.6	3	2	1	195.1	2.50E-44	chi-squared	one-tailed	510.2	703.2	1	1.20E-42	8.38E+41	1.20E-42
resDECjxallDist	resDECxallDist	-219	-337.7	4	3	1	237.3	1.50E-53	chi-squared	one-tailed	446.1	681.4	1	8.10E-52	1.24E+51	8.10E-52
resDECjx_constraints	resDECx_constraints	-230.6	-339.4	4	3	1	217.6	3.10E-49	chi-squared	one-tailed	469.2	684.8	1	1.60E-47	6.42E+46	1.60E-47
resDECjx_Lower_Sunda_Open	resDECx_Lower_Sunda_Open	-215.3	-335.6	4	3	1	240.6	2.80E-54	chi-squared	one-tailed	438.5	677.2	1	1.50E-52	6.63E+51	1.50E-52

alt	null	LnLalt	LnLnull	DFalt	DFnull	DF	Dstatistic	pval	test	tail	AIC1	AIC2	AICwt1	AICwt2	AICweight_ratio_model1	AICweight_ratio_model2
resDECj_time	resDEC_time	-257.5	-361.6	3	2	1	208	3.70E-47	chi-squared	one-tailed	521.1	727.1	1	1.80E-45	5.53E+44	1.80E-45
resDECj_constraints_time	resDEC_constraints_time	-296.9	-368.6	3	2	1	143.5	4.60E-33	chi-squared	one-tailed	599.8	741.3	1	1.90E-31	5.29E+30	1.90E-31
resDECj_Lower_Sunda_Open_time	resDEC_Lower_Sunda_Open_time	-260.7	-356.8	3	2	1	192.3	9.90E-44	chi-squared	one-tailed	527.3	717.6	1	4.70E-42	2.12E+41	4.70E-42
resDECjx_allopen_time	resDECx_allopen_time	-233.2	-348.4	4	3	1	230.3	5.00E-52	chi-squared	one-tailed	474.5	702.8	1	2.60E-50	3.84E+49	2.60E-50
resDECjx_constraints_time	resDECx_constraints_time	-239.6	-347.3	4	3	1	215.3	9.70E-49	chi-squared	one-tailed	487.3	700.6	1	4.90E-47	2.06E+46	4.90E-47
resDECjx_Lower_Sunda_Open_time	resDECx_Lower_Sunda_Open_time	-230.2	-337.9	4	3	1	215.4	9.00E-49	chi-squared	one-tailed	468.3	681.7	1	4.50E-47	2.20E+46	4.50E-47

Table S10 Primers and PCR programs used in this study. D= Denaturation, A= Annealing, E= Elongation

Gene	Location	Primer	Direction	Sequence (5' -> 3')	PCR protocol	References
Cytochrome c oxidase 1	mitochondrial	LCO1490-JJ	FOR	CHACWAAYCATAAAGATATYGG	D:94°C for 30 sec; A:47(52)°C for 40 sec; E:72°C for 60 sec	*Astrin & Stüben, 2008*
		HCO2198-JJ	REV	AWACTTCVGGRTGVCCAAARAATCA	5 cycles 47°C, 30 cycles 52°C	*Astrin & Stüben, 2008*
Cytochrome c oxidase 1	mitochondrial	Jerry	FOR	CAACATTTATTTTGATTTTTTGG	D:94°C for 30 sec; A:47(52)°C for 40 sec; E:72°C for 60 sec	Simon et al., 1994
		Pat	REV	TCCAATGCACTAATCTGCCATATTA	5 cycles 47°C, 30 cycles 52°C	Simon et al., 1994
16S	mitochondrial	1472-JJ	FOR	AGATAGAAACCRACCTGG	D:94°C for 30 sec; A:47(52)°C for 40 sec; E:72°C for 60 sec	*Astrin & Stüben, 2008*
		ar-JJ	REV	CRCCTGTTTATTAAAAACAT	5 cycles 47°C, 30 cycles 52°C	*Astrin & Stüben, 2008*
18S	nuclear	18S 5'	FOR	GACAACCTGGTTGATCCTGCCAGT	D:94°C for 30 sec; A:47(52)°C for 40 sec; E:72°C for 60 sec	Shull et al., 2001
		18S b5.0	REV	TAACCGCAACAACTTTAAT	5 cycles 47°C, 30 cycles 52°C	Shull et al., 2001
CAD1	nuclear	CD439F	FOR	TTCAGTGTACARTTYCAYCCHGARCAYAC	D:94°C for 30 sec; A:53°C for 30 sec; E:72°C for 60 sec	*Wild & Maddison, 2008*
		CD688R	REV	TGTATACCTAGAGGATCDACRTTYTCCATRTTRCA	35 cycles	*Wild & Maddison, 2008*
CAD2	nuclear	CD667F	FOR	GGATGGAAGGAAGTDGARTAYGARGT	D:94°C for 30 sec; A:53°C for 30 sec; E:72°C for 60 sec	*Wild & Maddison, 2008*
		CD851R	REV	GGATCGAAGCCATTHACATTYTCRTCHACCAT	35 cycles	*Wild & Maddison, 2008*
CAD3	nuclear	CD821F	FOR	AGCACGAAAATHGGNAGYTCNATGAARAG	D:94°C for 30 sec; A:53°C for 30 sec; E:72°C for 60 sec	*Wild & Maddison, 2008*
		CD1098R2	REV	GCTATGTTGTTNGGNAGYTGDCCNCCCAT	35 cycles	*Wild & Maddison, 2008*
Enolase	nuclear	EN37F	FOR	GACTCTCGTGGNAAYCCNACNGTNGAGGT	D:94°C for 30 sec; A:53°C for 30 sec; E:72°C for 60 sec	*Wild & Maddison, 2008*
		EN731R	REV	CTTGTAGAACTCNGANGCNGCNACRTCCAT	35 cycles	*Wild & Maddison, 2008*
28S	nuclear	28SJJ-a	FOR	ATGGATGGCGCTGAAGCGTCGT	D:94°C for 30 sec; A:48°C for 30 sec; E:72°C for 60 sec	*Astrin & Stüben, 2008*
		28SJJ-b	REV	CCGACGAACTCTCTTGCGAGG	35 cycles	*Astrin & Stüben, 2008*
Arginine kinase	nuclear	AK183F	FOR	GATTCTGGAGTCGGNATYTAYGCNCCYGAYGC	D:94°C for 30 sec; A:53°C for 30 sec; E:72°C for 60 sec	*Wild & Maddison, 2008*
		AK939R	REV	GCCNCCYTCRGCYTCRGTGTGYTC	35 cycles	*Wild & Maddison, 2008*
Elongation factor 1alpha	nuclear	efs372	FOR	CTGGTGAATTTGAAGCYGGTA	D:94°C for 30 sec; A:58-42°C for 30 sec; E:72°C for 60 sec	*McKenna et al, 2005*
		efa754	REV	CCACCAATTTTGTAGACATC	[illegible]T -2°C every 3 cycles (58-44°C); 18 cycles 42°C	*Normark et al, 1999*
Histone 4	nuclear	H4F2s	FOR	TSCGIGAYAACATYCAGGGIATCAC	D:94°C for 30 sec; A:49°C for 30 sec; E:72°C for 60 sec	*Pineau et al, 2004*
		H4F2er	REV	CKYTTIAGIGCRTAIACCACRTCCAT	35 cycles	*Pineau et al, 2004*

Table S11 Partitioning strategies as used for the Bayesian and maximum likelihood inferences.

**Partition1**	16S, 18S, 28S, AK_CP1, AK_CP2, Cad1_CP1, Cad1_CP2, Cad2_CP2, Cad2_CP3, Cad3_CP2, Cad3_CP3, EF1a_CP1, EF1a_CP2, EN_CP1, EN_CP2, H4_CP1, H4_CP2, HCOLCO_CP1, HCOLCO_CP2, PJ_CP1, PJ_CP2
**Partition2**	AK_CP3, Cad1_CP3, Cad2_CP1, Cad3_CP1, EF1a_CP3, EN_CP3, H4_CP3
**Partition3**	HCOLCO_CP3, PJ_CP3

Table S12 Overview of the species used in this study, collecting locations, and gene fragments used in the dataset. Successful sequencing of a gene fragment is indicated by "+". Numbers behind the primers indicate the length of the fragment.

Specimen code	Species	Locality	HcoLco (657)	PatJerry (689)	AK (722)	H4 (200)	EF1a (326)	18S (578)	16S (635)	CAD1 (462)	CAD2 (595)	CAD3 (631)	28S (353)	EN (663)
ARC0183	Trigonopterus halimunensis	Java-West	+	+	+	+	-	+	+	+	-	+	-	+
ARC0184	Trigonopterus suturalis	Java-West	+	+	-	+	+	-	+	+	-	+	-	+
ARC0204	Trigonopterus echinatus	Java-West	+	+	-	+	+	-	+	+	-	+	-	+
ARC0209	Trigonopterus sundaicus	Java-West	+	+	+	+	+	-	+	+	-	+	-	+
ARC0211	Trigonopterus dimorphus	Java-West	+	+	+	+	-	-	+	+	-	+	-	+
ARC0218	Trigonopterus binodulus	Java-West	+	+	+	+	-	-	+	+	+	+	+	+
ARC0249	Trigonopterus gedensis	Java-West	+	+	-	-	+	+	+	-	-	-	+	-
ARC0254	Trigonopterus vulcanicus	Java-West	+	+	+	+	+	+	+	-	-	+	+	+
ARC0259	Trigonopterus amphoralis	Sumatra	+	+	+	+	+	-	+	+	-	+	-	+
ARC0263	Trigonopterus misellus	Sumatra	+	+	-	-	+	-	-	+	-	+	-	+
ARC0266	Trigonopterus lampungensis	Sumatra	+	+	+	-	+	-	+	+	-	+	-	+
ARC0271	Trigonopterus seriatus	Sumatra	+	-	+	+	-	-	+	+	-	+	+	-
ARC0275	Trigonopterus sumatrensis	Sumatra	+	+	+	-	-	-	+	+	-	+	-	+
ARC0281	Trigonopterus singularis	Sumatra	+	+	+	-	+	-	+	+	-	+	-	+
ARC0284	Trigonopterus duabelas	Java-East	+	-	-	+	+	-	+	-	-	+	-	+
ARC0290	Trigonopterus diengensis	Java-East	+	+	-	+	+	-	-	-	-	-	-	-
ARC0296	Trigonopterus javensis	Java-East	+	+	+	+	+	-	-	-	-	+	-	+
ARC0301	Trigonopterus relictus	Java-East	+	+	-	+	-	+	+	+	-	+	+	+
ARC0303	Trigonopterus ijensis	Java-East	+	+	+	+	+	-	+	-	-	+	+	+
ARC0312	Trigonopterus variolosus	Java-West	+	-	-	+	+	-	-	+	-	+	-	+
ARC0323	Trigonopterus sp. 427	Sulawesi	+	-	-	+	+	+	+	-	-	-	+	-
ARC0422	Trigonopterus lineellus	New Guinea	+	-	-	+	+	+	+	+	+	+	+	+
ARC0426	Trigonopterus eremitus	New Guinea	+	+	+	+	+	+	+	+	+	+	+	+
ARC0429	Trigonopterus tibialis	New Guinea	+	+	+	+	-	+	+	+	+	+	+	+
ARC0460	Trigonopterus vandekampi	New Guinea	+	+	+	-	+	+	+	+	-	+	+	+
ARC0475	Trigonopterus ferrugineus	New Guinea	+	+	+	-	+	+	+	-	+	+	+	+
ARC0492	Trigonopterus pseudonasutus	New Guinea	+	+	+	+	+	+	+	-	-	+	+	+
ARC0508	Trigonopterus sulcatus	New Guinea	+	+	+	+	+	+	-	-	-	+	+	+
ARC0516	Trigonopterus zygops	New Guinea	+	+	+	+	+	+	+	-	+	+	+	+
ARC0519	Trigonopterus lineatus	New Guinea	+	-	+	+	+	+	+	+	+	+	+	+
ARC0530	Trigonopterus aeneipennis	New Guinea	+	+	-	-	+	+	+	-	+	+	+	+
ARC0534	Trigonopterus dromedarius	New Guinea	+	-	+	-	+	+	+	+	+	+	+	+
ARC0538	Trigonopterus dentirostris	New Guinea	+	-	+	+	+	+	+	+	+	+	+	+
ARC0562	Trigonopterus rhinoceros	New Guinea	+	+	+	+	+	+	-	-	+	+	+	+
ARC0576	Trigonopterus baliensis	Bali	+	+	+	+	-	+	+	+	-	+	+	+
ARC0584	Trigonopterus pararugosus	Bali	+	+	-	-	+	+	+	+	+	+	+	+
ARC0586	Trigonopterus rugosus	Bali	+	+	-	+	-	+	-	-	+	+	+	-
ARC0589	Trigonopterus kintamanensis	Bali	+	-	+	+	+	+	+	-	-	+	+	+
ARC0595	Trigonopterus klatakanensis	Bali	+	+	+	+	+	+	+	-	-	+	+	+
ARC0606	Trigonopterus tridentatus	New Guinea	+	+	-	+	+	+	-	-	+	-	+	+
ARC0670	Trigonopterus sp. 1	New Guinea	+	+	+	+	+	+	+	+	+	+	+	+
ARC0678	Trigonopterus scharfi	New Guinea	+	+	+	+	+	+	+	+	-	+	+	-
ARC0695	Trigonopterus sp. 31	New Guinea	+	+	+	+	+	+	+	-	+	-	+	-
ARC0713	Trigonopterus bornensis	Borneo	+	-	-	+	+	-	+	-	-	+	-	+
ARC0714	Trigonopterus sebelas	Borneo	+	+	+	+	+	+	+	-	+	+	+	-
ARC0735	Trigonopterus kurulu	New Guinea	+	+	+	-	+	+	+	-	+	+	+	+
ARC0836	Trigonopterus scissops	New Guinea	+	+	+	+	+	+	+	+	+	+	+	+
ARC0858	Trigonopterus inflatus	New Guinea	+	+	+	+	-	+	+	-	+	+	+	+
ARC0914	Critomerus iliacus	New Guinea	+	+	+	+	+	+	-	+	+	+	+	+
ARC0967	Trigonopterus sp. 150	New Guinea	+	+	+	+	+	+	+	+	+	+	+	+
ARC1179	Trigonopterus discoidalis	New Guinea	+	+	+	+	-	+	+	-	-	+	+	+
ARC1235	Trigonopterus sp. 165	New Guinea	+	+	-	+	-	+	+	-	-	-	+	-
ARC1320	Trigonopterus micros	New Guinea	+	+	+	+	+	+	+	-	-	-	+	-
ARC1391	Microporopterus cf. setosus	New Guinea	+	+	+	+	+	+	+	-	-	+	+	-
ARC1422	Trigonopterus kalimantanensis	Borneo	+	+	-	-	-	+	+	-	-	-	-	+
ARC1437	Trigonopterus schulzi	Philippines	+	+	+	-	+	+	+	-	+	+	+	+
ARC1440	Trigonopterus palawanensis	Philippines	+	+	+	+	+	-	-	+	-	-	-	+
ARC1458	Trigonopterus lombokensis	Lombok	+	+	+	+	-	+	+	+	-	+	+	+
ARC1461	Trigonopterus dentipes	Lombok	+	+	+	+	+	+	+	-	-	+	+	+
ARC1462	Trigonopterus aeneomicans	Lombok	+	+	+	+	+	+	+	-	-	+	+	+
ARC1466	Trigonopterus disruptus	Lombok	+	+	+	+	+	+	+	-	+	+	+	+
ARC1470	Trigonopterus rinjaniensis	Lombok	+	+	+	+	+	+	+	+	-	+	+	+
ARC1481	Trigonopterus sasak	Lombok	+	+	+	+	+	+	+	+	-	+	+	+
ARC1497	Trigonopterus punctatoseriatus	Sumbawa	+	+	+	+	+	+	+	-	-	+	+	+
ARC1499	Trigonopterus sembilan	Sumbawa	+	+	+	+	+	+	+	+	-	+	+	+
ARC1505	Trigonopterus cupreus	Sumbawa	+	+	+	+	-	-	-	+	-	+	-	+
ARC1509	Trigonopterus sinuatus	Sumbawa	+	+	+	+	+	+	+	+	-	+	+	+
ARC1513	Trigonopterus allotopus	Sumbawa	+	-	+	+	+	+	+	+	-	+	+	-
ARC1516	Trigonopterus sumbawensis	Sumbawa	+	+	+	-	+	+	+	+	-	+	+	+
ARC1529	Trigonopterus saltator	Sumbawa	+	+	+	+	+	+	+	+	-	+	+	+
ARC1534	Trigonopterus pauxillus	Sumbawa	+	+	+	+	+	+	+	+	-	-	+	+
ARC1542	Trigonopterus honjensis	Java-West	+	+	+	+	+	-	+	+	-	+	-	+
ARC1552	Miocalles sp.	Java-West	+	+	+	+	+	+	+	+	-	-	+	+

Specimen code	Species	Locality	HcoLco (657)	PatJerry (689)	AK (722)	H4 (200)	EF1a (326)	18S (578)	16S (635)	CAD1 (462)	CAD2 (595)	CAD3 (631)	28S (353)	EN (663)
ARC1675	Ouroporopterus squamiventris	Australia	+	+	+	+	-	+	+	+	-	+	+	-
ARC1707	Trigonopterus sp. 93	New Guinea	+	+	-	+	+	+	+	+	-	+	+	-
ARC1722	Trigonopterus sp. 70	New Guinea	+	+	-	-	-	+	+	+	+	+	+	+
ARC1732	Trigonopterus sp. 117	New Guinea	+	-	-	+	+	+	+	+	+	+	+	+
ARC1790	Trigonopterus durus	New Guinea	+	+	-	+	+	+	+	-	-	+	+	+
ARC2189	Trigonopterus florensis	Flores	+	+	+	-	-	+	+	+	+	+	+	+
ARC2192	Trigonopterus empat	Flores	+	+	+	-	+	+	+	+	+	+	+	+
ARC2193	Trigonopterus rutengensis	Flores	+	-	+	+	+	-	+	-	+	+	-	+
ARC2195	Trigonopterus syarbis	Flores	+	-	+	+	-	-	+	+	+	+	-	+
ARC2198	Trigonopterus ranakensis	Flores	+	+	+	+	+	+	+	+	+	+	+	+
ARC2199	Trigonopterus lima	Flores	+	+	+	-	+	-	+	+	+	+	-	+
ARC2200	Trigonopterus dacrycarpi	Flores	+	+	+	+	+	-	+	+	+	+	-	+
ARC2205	Trigonopterus paraflorensis	Flores	+	-	+	+	+	-	+	+	+	+	-	+
ARC2212	Trigonopterus fissitarsis	Flores	+	-	+	+	-	+	+	-	+	+	+	+
ARC2213	Trigonopterus micans	Flores	+	+	+	+	+	+	+	-	+	+	+	+
ARC2223	Trigonopterus serratifemur	Flores	+	+	+	-	-	-	+	+	+	+	-	+
ARC2231	Trigonopterus tujuh	Flores	+	+	+	+	+	+	+	+	+	+	+	+
ARC2235	Trigonopterus cuprescens	Flores	+	+	+	+	-	-	+	-	+	+	-	+
ARC2242	Trigonopterus delapan	Flores	+	+	-	+	+	+	+	-	-	+	+	+
ARC2243	Trigonopterus tiga	Flores	+	+	+	+	-	+	+	+	+	+	-	+
ARC2245	Trigonopterus roensis	Flores	+	+	+	+	+	+	+	+	-	+	+	+
ARC2247	Trigonopterus mesehensis	Bali	+	+	-	+	-	+	+	+	+	+	+	+
ARC2256	Trigonopterus fulgidus	Lombok	+	+	+	+	-	+	+	-	+	+	+	+
ARC2302	Trigonopterus batukarensis	Bali	+	-	+	+	+	+	+	+	+	+	+	+
ARC2314	Trigonopterus telagensis	Bali	+	+	+	+	-	+	+	+	+	+	+	+
ARC2326	Trigonopterus alaspurwensis	Java-East	+	+	+	+	+	+	+	+	-	+	+	+
ARC2331	Trigonopterus satu	Java-East	+	-	+	+	-	-	+	+	+	+	-	+
ARC2332	Trigonopterus merubetirensis	Java-East	+	+	+	+	-	-	+	-	-	+	-	+
ARC2458	Trigonopterus argopurensis	Java-East	+	+	+	-	+	+	+	-	-	+	+	+
ARC2466	Trigonopterus latipes	Java-East	+	+	+	+	+	-	+	+	-	+	-	+
ARC2473	Trigonopterus arjunensis	Java-East	+	+	+	+	+	-	+	+	-	+	-	+
ARC2481	Trigonopterus costipennis	Java-East	+	-	+	-	-	+	+	+	+	+	+	+
ARC2483	Trigonopterus acuminatus	Java-East	+	+	+	+	+	+	-	+	-	-	+	+
ARC2504	Trigonopterus cahyoi	Java-West	+	+	+	+	+	+	+	+	-	+	+	+
ARC2506	Trigonopterus rugosostriatus	Java-West	+	+	-	+	+	-	+	+	-	+	-	+
ARC2511	Trigonopterus allopatricus	Java-West	+	+	+	+	+	-	+	+	-	+	-	+
ARC2514	Trigonopterus vulcanicus	Java-West	+	+	+	+	-	-	+	+	-	+	-	+
ARC2522	Trigonopterus asper	Java-West	+	+	+	+	+	-	-	+	-	+	-	+
ARC2526	Trigonopterus wallacei	Borneo	+	-	+	+	+	+	+	+	-	+	+	-
ARC2527	Trigonopterus santubongensis	Borneo	+	-	+	+	+	-	+	-	-	+	-	+
ARC2531	Trigonopterus trigonopterus	Borneo	+	+	+	+	-	+	+	+	+	+	+	+
ARC2536	Trigonopterus bawangensis	Borneo	+	+	+	+	-	-	+	-	-	+	-	+
ARC2538	Trigonopterus sepuluh	Borneo	+	+	+	+	-	+	+	+	-	+	+	+
ARC2542	Trigonopterus attenboroughi	Borneo	+	+	+	+	-	+	+	-	-	+	+	+
ARC2548	Trigonopterus singkawangensis	Borneo	+	-	+	+	+	-	+	-	-	-	-	-
ARC2588	Trigonopterus enam	Flores	+	+	+	+	+	+	+	+	-	+	+	+
ARC2668	Trigonopterus silvestris	Java-West	+	+	+	+	+	-	+	-	-	+	-	+
ARC2683	Trigonopterus pangandaranensis	Java-West	+	+	+	+	+	-	+	-	-	+	-	+
ARC2685	Trigonopterus foveatus	Java-West	+	+	+	+	+	-	+	-	-	+	-	+
ARC2700	Trigonopterus porcatus	Java-West	+	-	+	+	+	-	+	-	-	+	-	+
ARC2703	Trigonopterus payungensis	Java-West	+	+	+	+	+	-	+	-	-	+	-	+
ARC2704	Trigonopterus setifer	Java-West	+	+	+	+	+	-	+	-	-	+	-	+
ARC2741	Trigonopterus squalidus	Sumatra	+	+	+	-	+	-	+	-	-	+	-	+
ARC2806	Trigonopterus sp. 413	Sulawesi	+	+	+	+	+	-	+	-	-	+	-	+
ARC2815	Trigonopterus sp. 379	Sulawesi	+	-	+	+	-	-	+	-	-	+	-	+
ARC2819	Trigonopterus sp. 376	Sulawesi	+	+	-	+	-	+	+	-	-	-	-	+
ARC2821	Trigonopterus sp. 409	Sulawesi	+	+	+	+	+	-	+	-	-	+	-	+
ARC2823	Trigonopterus sp. 579	Sulawesi	+	+	-	+	-	-	+	-	-	-	-	-
ARC2828	Trigonopterus sp. 416	Sulawesi	+	-	+	+	+	-	+	-	-	+	-	-
ARC2847	Trigonopterus sp. 417	Sulawesi	+	-	+	-	-	-	+	-	-	+	-	+
ARC2854	Trigonopterus sp. 423	Sulawesi	+	-	+	+	+	-	+	-	-	+	-	-
ARC2855	Trigonopterus sp. 421	Sulawesi	+	+	+	+	+	+	+	-	-	+	-	+
ARC2863	Trigonopterus sp. 408	Sulawesi	+	+	+	-	+	+	+	-	-	+	-	+
ARC2868	Trigonopterus sp. 404	Sulawesi	+	+	+	-	+	-	+	-	-	+	-	+
ARC2873	Trigonopterus sp. 377	Sulawesi	+	+	+	+	+	+	+	-	-	+	-	+
ARC2876	Trigonopterus sp. 401	Sulawesi	+	+	+	+	-	+	+	-	-	+	-	+
ARC2888	Trigonopterus sp. 380	Sulawesi	+	+	-	-	-	+	+	-	-	+	-	-
ARC2900	Trigonopterus sp. 397-A	Sulawesi	+	+	+	-	+	+	+	-	-	+	-	+
ARC2916	Trigonopterus sp. 395	Sulawesi	+	+	+	+	-	+	+	-	-	+	-	+
ARC2970	Trigonopterus sp. 456	Maluku	+	+	+	-	+	+	+	-	-	+	+	+
ARC2985	Trigonopterus sp. 457	Maluku	+	+	+	-	-	+	+	+	-	+	+	+
ARC2999	Trigonopterus sp. 515	Sulawesi	+	+	+	+	+	+	+	-	-	+	+	+
ARC3005	Trigonopterus sp. 497	Sulawesi	+	+	-	+	+	+	+	-	-	+	+	+

Specimen code	Species	Locality	HcoLco (657)	PatJerry (689)	AK (722)	H4 (200)	EF1a (326)	18S (578)	16S (635)	CAD1 (462)	CAD2 (595)	CAD3 (631)	28S (353)	EN (663)
ARC3025	Trigonopterus sp. 510	Sulawesi	+	+	+	+	+	-	+	-	-	+	+	+
ARC3036	Trigonopterus sp. 499	Sulawesi	+	+	+	+	+	+	+	-	-	+	+	+
ARC3043	Trigonopterus sp. 474	Sulawesi	+	+	+	+	+	+	+	-	-	+	+	+
ARC3047	Trigonopterus sp. 505	Sulawesi	+	+	+	+	-	+	+	-	-	+	+	+
ARC3053	Trigonopterus sp. 498	Sulawesi	+	-	-	-	-	+	+	-	-	+	-	+
ARC3055	Trigonopterus sp. 516	Sulawesi	+	+	+	+	+	+	+	-	-	+	+	+
ARC3068	Trigonopterus sp. 511	Sulawesi	+	+	+	+	+	+	+	-	-	+	+	+
ARC3093	Trigonopterus sp. 509	Sulawesi	+	+	+	+	+	+	+	-	-	+	+	+
ARC3106	Trigonopterus sp. 506	Sulawesi	+	+	+	+	-	+	+	-	-	+	-	+
ARC3125	Trigonopterus sp. 476	Sulawesi	+	+	-	+	+	+	+	-	-	+	+	+
ARC3131	Trigonopterus sp. 492	Sulawesi	+	+	-	+	+	+	+	-	-	-	+	+
ARC3132	Trigonopterus sp. 495	Sulawesi	+	+	+	+	+	+	+	+	-	+	+	+
ARC3136	Trigonopterus sp. 483	Sulawesi	+	+	+	+	+	+	+	+	-	+	+	+
ARC3139	Trigonopterus sp. 469	Sulawesi	+	+	-	-	+	+	+	+	-	+	+	+
ARC3142	Trigonopterus sp. 481	Sulawesi	+	+	+	+	-	+	+	-	-	+	-	+
ARC3146	Trigonopterus sp. 489	Sulawesi	+	+	-	+	-	+	+	-	-	+	+	+
ARC3150	Trigonopterus sp. 504	Sulawesi	+	+	+	-	-	+	+	-	-	+	+	+
ARC3159	Trigonopterus sp. 482	Sulawesi	+	+	-	+	-	+	+	-	-	+	+	+
ARC3173	Trigonopterus sp. 493	Sulawesi	+	+	+	-	-	+	+	+	-	+	-	+
ARC3179	Trigonopterus sp. 473	Sulawesi	+	-	-	+	-	+	+	+	-	+	+	+
ARC3181	Trigonopterus sp. 484	Sulawesi	+	+	+	+	-	+	+	-	-	+	-	-
ARC3183	Trigonopterus sp. 501	Sulawesi	+	+	+	+	-	+	+	+	-	+	+	+
ARC3189	Trigonopterus sp. 502	Sulawesi	+	+	-	-	+	+	+	-	-	+	+	-
ARC3196	Trigonopterus sp. 478	Sulawesi	+	+	+	+	+	+	+	-	-	+	-	+
ARC3197	Trigonopterus sp. 500	Sulawesi	+	+	+	+	+	+	+	+	-	+	+	+
ARC3209	Trigonopterus sp. 480	Sulawesi	+	+	+	+	+	+	-	-	-	+	+	+
ARC3211	Trigonopterus sp. 488	Sulawesi	+	+	+	-	+	+	+	+	-	+	+	+
ARC3233	Trigonopterus sp. 578	Sulawesi	+	+	+	+	+	+	+	-	-	+	-	+
ARC3234	Trigonopterus sp. 470	Sulawesi	+	+	+	-	+	+	+	+	-	+	+	+
ARC3239	Trigonopterus sp. 494	Sulawesi	+	+	+	+	+	+	+	-	-	+	+	+
ARC3247	Trigonopterus sp. 471	Sulawesi	+	+	+	+	-	+	-	-	-	+	+	+
ARC3267	Trigonopterus sp. 490	Sulawesi	+	+	+	+	-	+	-	-	-	+	+	-
ARC3274	Trigonopterus sp. 466	Sulawesi	+	-	+	+	+	+	+	+	-	+	+	-
ARC3288	Trigonopterus sp. 445	Maluku	+	+	-	+	-	+	+	+	-	+	+	+
ARC3291	Trigonopterus sp. 448	Maluku	+	+	-	+	-	+	+	+	-	+	+	+
ARC3299	Trigonopterus sp. 453	Maluku	+	+	+	-	-	+	+	+	-	+	+	+
ARC3300	Trigonopterus sp. 459	Maluku	+	+	+	+	+	+	+	-	-	+	+	+
ARC3303	Trigonopterus sp. 451	Maluku	+	+	-	+	-	+	+	+	-	+	+	+
ARC3307	Trigonopterus sp. 468	Maluku	+	-	-	+	+	+	+	-	-	+	+	-
ARC3311	Trigonopterus sp. 460	Maluku	+	+	-	+	+	+	+	+	-	-	+	-
ARC3326	Trigonopterus sp. 463	Maluku	+	+	+	+	+	+	+	+	-	-	+	-
ARC3330	Trigonopterus sp. 455	Maluku	+	+	+	+	+	+	+	-	-	+	+	+
ARC3359	Trigonopterus sp. 450	Maluku	+	-	-	+	-	+	+	+	-	+	+	+
ARC3367	Trigonopterus sp. 462	Maluku	+	+	+	-	-	+	+	+	-	+	+	-
ARC3388	Trigonopterus sp. 544	Maluku	+	-	-	+	+	+	+	+	-	+	-	+
ARC3588	Trigonopterus tepalensis	Sumbawa	+	+	+	+	+	-	+	+	+	-	+	+
ARC3602	Trigonopterus pseudosumbawensis	Sumbawa	+	+	+	-	+	-	+	+	+	+	+	+
ARC3603	Trigonopterus parasumbawensis	Sumbawa	+	+	+	-	+	-	+	+	+	+	+	+
ARC3613	Trigonopterus dua	Flores	+	+	-	+	+	-	+	+	+	+	+	+
