# Supplementary material for: Macroevolution of hyperdiverse flightless beetles reflects the complex geological history of the Sunda Arc
Source: Sci Rep. 2016 Jan 8;6:18793. doi: 10.1038/srep18793 (PMC4732383; doi:10.1038/srep18793)

Appendix S2 TABLE OF CONTENT .... Page

BioGeoBEARS constraints and results using a time calibrated tree with the emergence of Java

BioGeoBEARS results of biogeographical reconstructions

DEC & DEC+J unconstrained dispersal 3–6

DEC & DEC+J with dispersal constrained to adjacent areas 7–10

DEC & DEC+J with dispersal constrained to adjacent areas except for the Lower Sunda island chain (Java-E, Java-W, Bali, Lomboc, Sumbawa and Flores) 11–14

DEC+x & DEC+J+x unconstrained dispersal 15–18

DEC+x & DEC+J+x with dispersal is limited to adjacent areas 19–22

DEC+x & DEC+J+x with dispersal constrained to adjacent areas except for the Lower Sunda island chain 23–26

BioGeoBEARS results time-stratified

DEC & DEC+J unconstrained dispersal 27–30

DEC & DEC+J with dispersal constrained to adjacent areas 31–34

DEC & DEC+J with dispersal constrained to adjacent areas except for the Lower Sunda island chain (Java-E, Java-W, Bali, Lomboc, Sumbawa and Flores) 35–38

DEC+x & DEC+J+x unconstrained dispersal 39–42

DEC+x & DEC+J+x with dispersal is limited to adjacent areas 43–46

DEC+x & DEC+J+x with dispersal constrained to adjacent areas except for the Lower Sunda island chain 47–50

BioGeoBEARS constraints and results using a time calibrated tree with the emergence of New Guinea at 30Ma

BioGeoBEARS areas allowed matrix 51

BioGeoBEARS manual dispersal multipliers, time-stratified 52–54

BioGeoBEARS distances matrices, time-stratified 55–57

BioGeoBEARS results model weights based on AICc 58

BioGeoBEARS results model parameter estimates table 59

BioGeoBEARS results Likelihood ratio test results and AIC values table 60

BioGeoBEARS constraints and results of biogeographical reconstructions

DEC & DEC+J unconstrained dispersal 61–64

DEC & DEC+J with dispersal constrained to adjacent areas 65–68

DEC & DEC+J with dispersal constrained to adjacent areas except for the Lower Sunda island chain (Java-E, Java-W, Bali, Lomboc, Sumbawa and Flores) 69–72

DEC+x & DEC+J+x unconstrained dispersal 73–76

DEC+x & DEC+J+x with dispersal is limited to adjacent areas 77–80

DEC+x & DEC+J+x with dispersal constrained to adjacent areas except for the Lower Sunda island chain 81–84

BioGeoBEARS results time-stratified

DEC & DEC+J unconstrained dispersal 85–88

DEC & DEC+J with dispersal constrained to adjacent areas 89–92

DEC & DEC+J with dispersal constrained to adjacent areas except for the Lower Sunda island chain (Java-E, Java-W, Bali, Lomboc, Sumbawa and Flores) 93–96

DEC+x & DEC+J+x unconstrained dispersal 97–100

DEC+x & DEC+J+x with dispersal is limited to adjacent areas 101–104

DEC+x & DEC+J+x with dispersal constrained to adjacent areas except for the Lower Sunda island chain 105–108

BioGeoBEARS DEC on Trigonopterus
ancstates: global optim, 3 areas max. d=0.0046; e=0.0085; j=0; x=0; LnL=−357.35

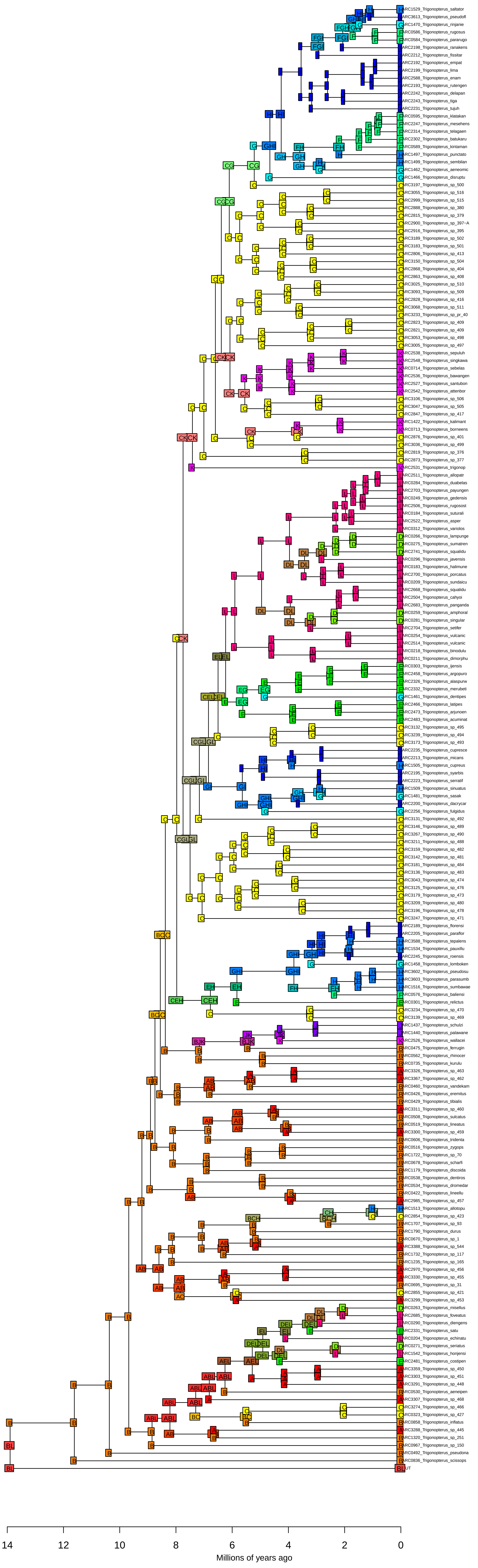

BioGeoBEARS DEC on Trigonopterus

ancstates: global optim, 3 areas max. d=0.0046; e=0.0085; j=0; x=0; LnL=−357.35

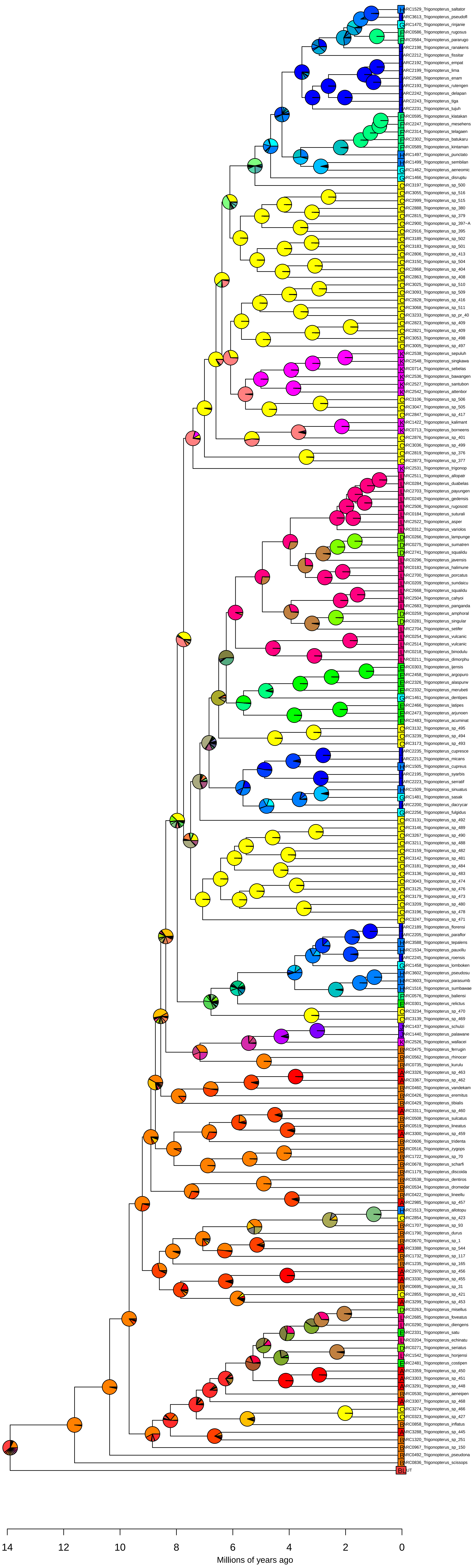

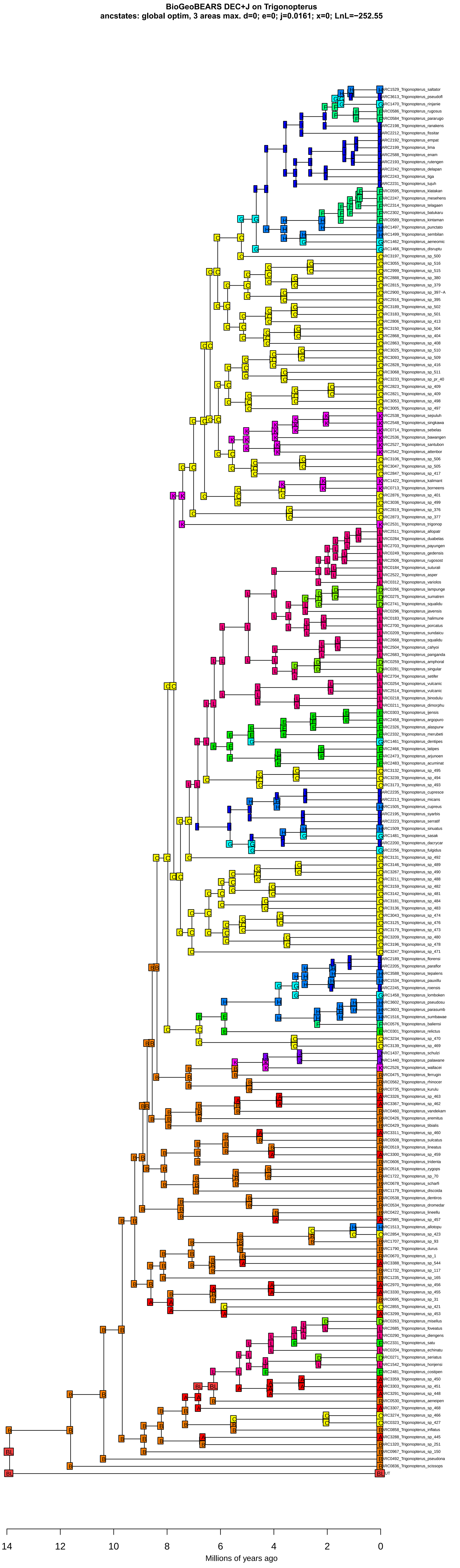
BioGeoBEARS DEC+J on Trigonopterus
ancstates: global optim, 3 areas max. d=0; e=0; j=0.0161; x=0; LnL=−252.55
14 12 10 8 6 4 2 0
Millions of years ago

BioGeoBEARS DEC+J on Trigonopterus
ancstates: global optim, 3 areas max. d=0; e=0; j=0.0161; x=0; LnL=−252.55

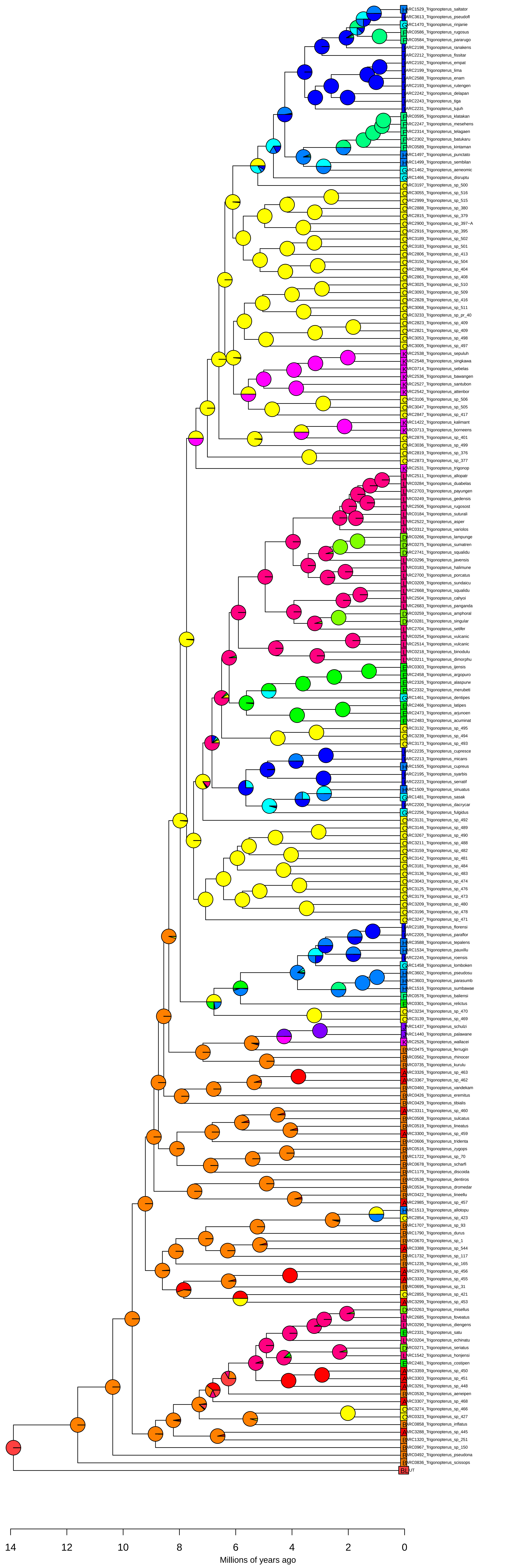

BioGeoBEARS DEC constraints on Trigonopterus
ancstates: global optim, 3 areas max. d=0.0082; e=0.0176; j=0; x=0; LnL=−362.48

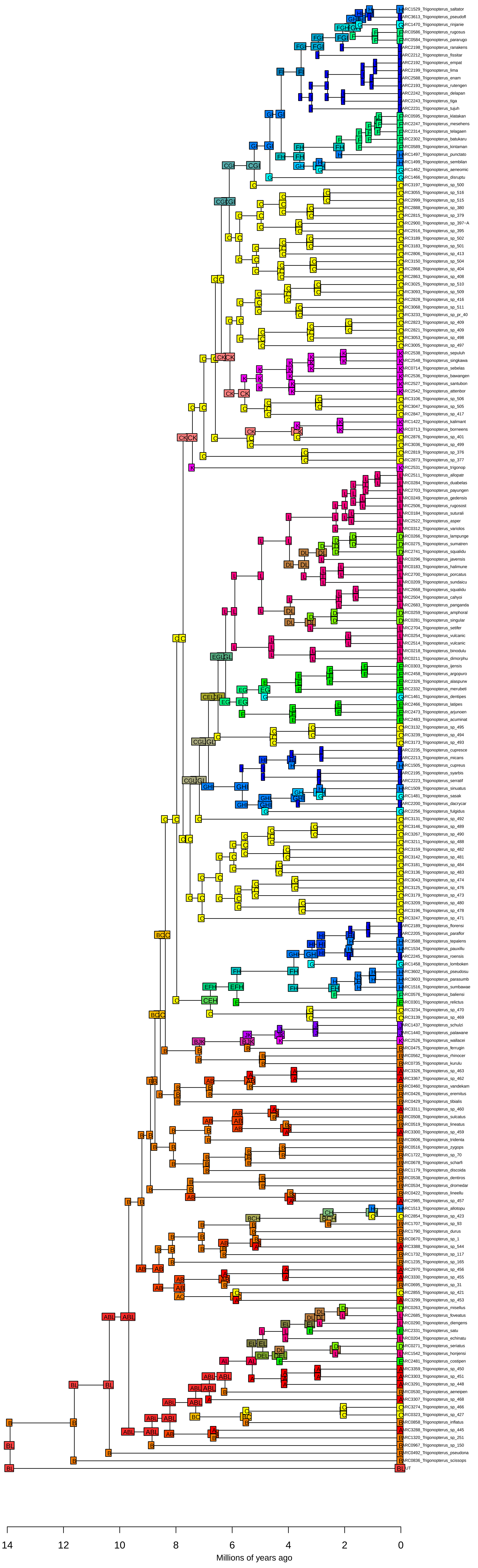

BioGeoBEARS DEC constraints on Trigonopterus
ancstates: global optim, 3 areas max. d=0.0082; e=0.0176; j=0; x=0; LnL=−362.48

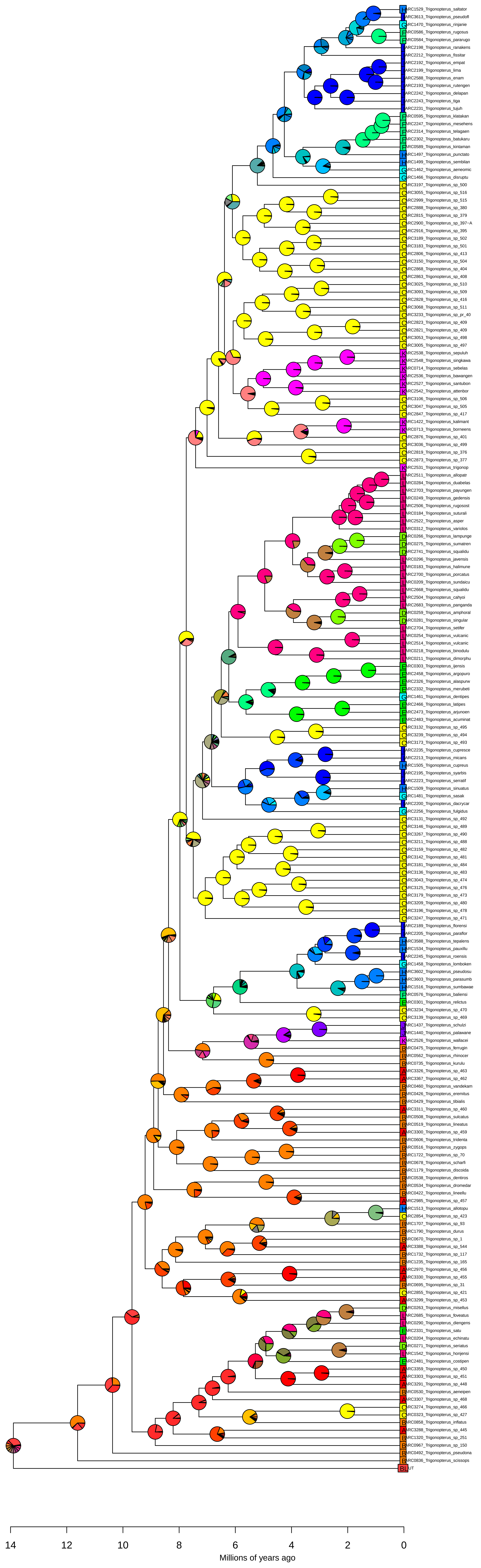

BioGeoBEARS DEC+J constraints on Trigonopterus
ancstates: global optim, 3 areas max. d=0.0011; e=0.005; j=0.0283; x=0; LnL=−292.05

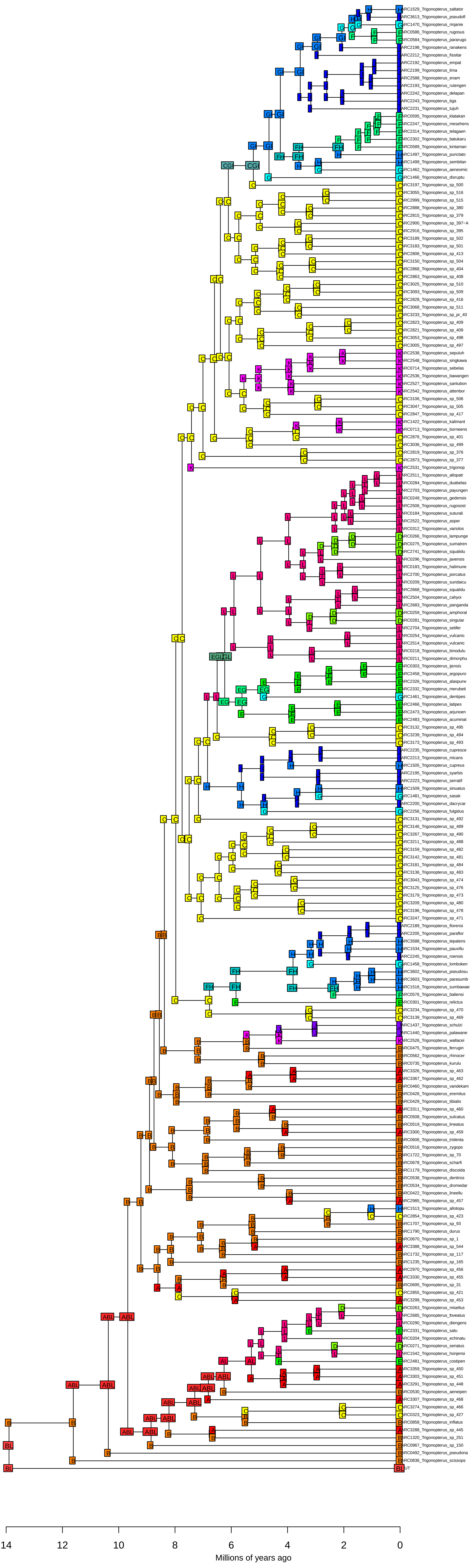

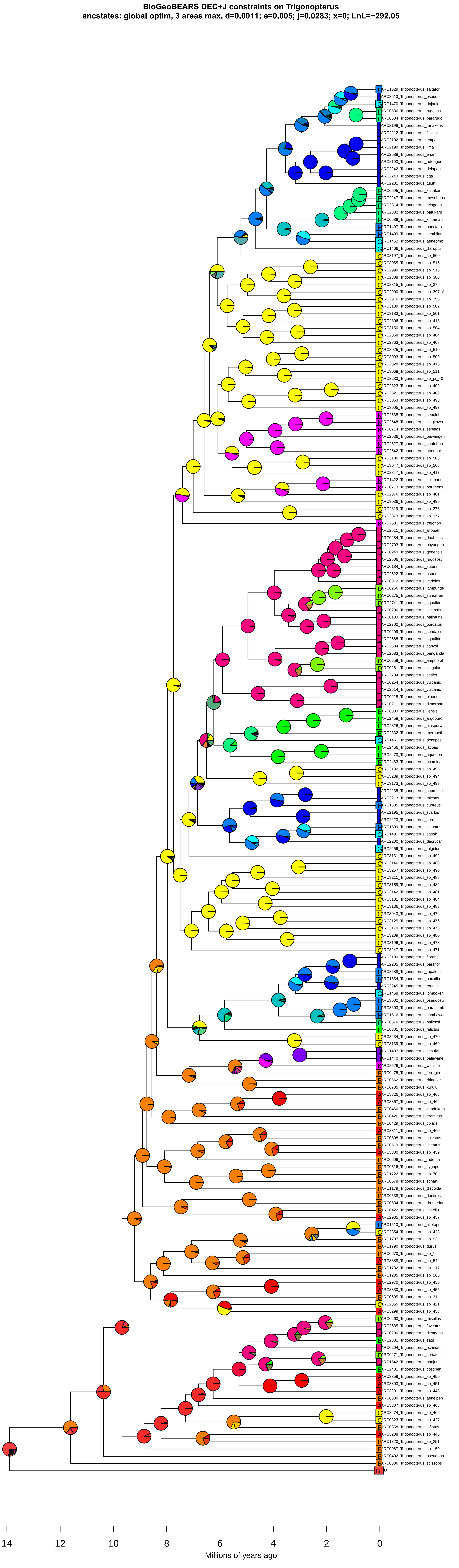
BioGeoBEARS DEC+J constraints on Trigonopterus
ancstates: global optim, 3 areas max. d=0.0011; e=0.005; j=0.0283; x=0; LnL=−292.05
14
12
10
8
6
4
2
0
Millions of years ago

BioGeoBEARS DEC constraints Lower Sunda Open on Trigonopterus
ancstates: global optim, 3 areas max. d=0.0068; e=0.0119; j=0; LnL=−349.62

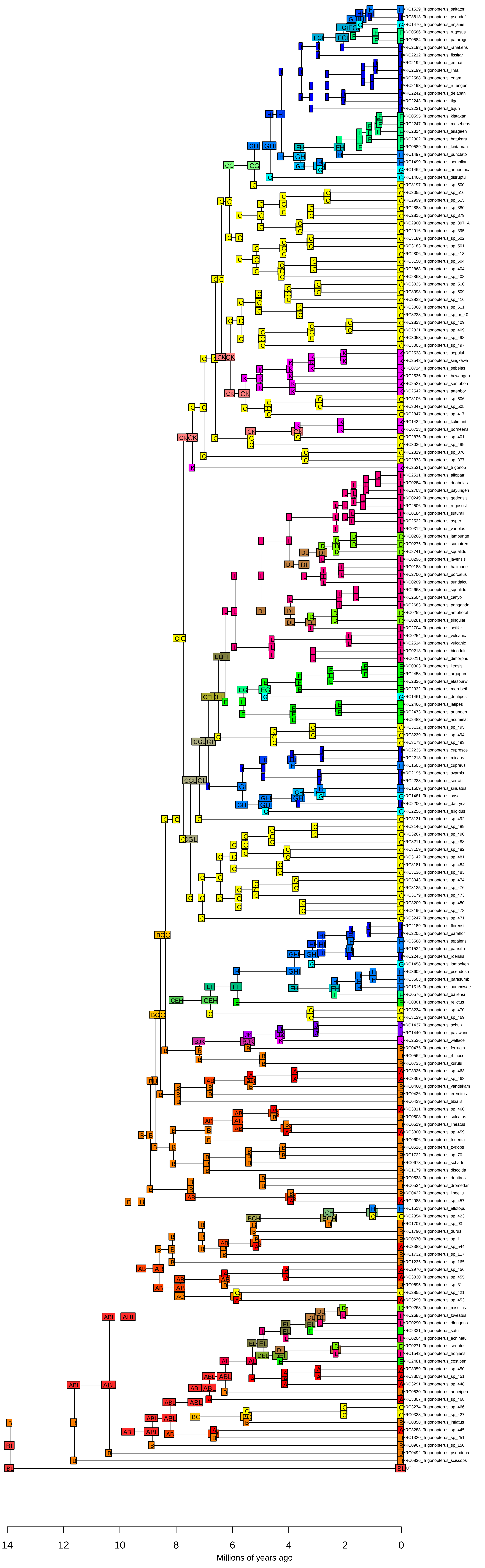

BioGeoBEARS DEC constraints Lower Sunda Open on Trigonopterus
ancstates: global optim, 3 areas max. d=0.0068; e=0.0119; j=0; LnL=−349.62

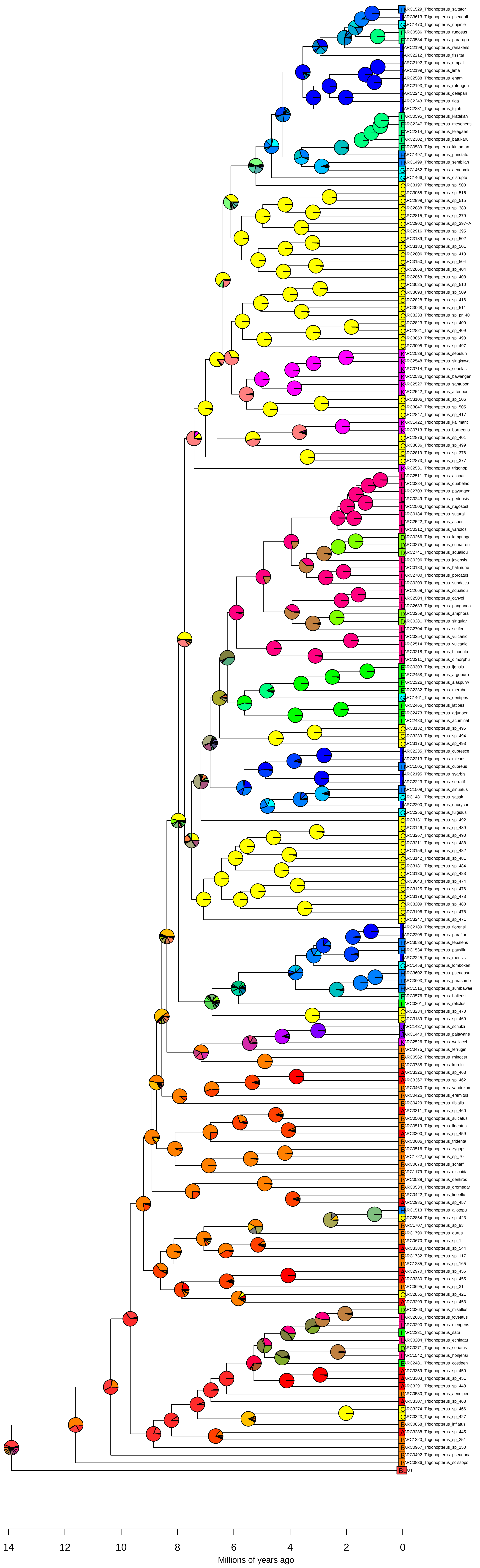

BioGeoBEARS DEC+J constraints Lower Sunda Open on Trigonopterus
ancstates: global optim, 3 areas max. d=2e−04; e=0.0015; j=0.0199; LnL=−252.09

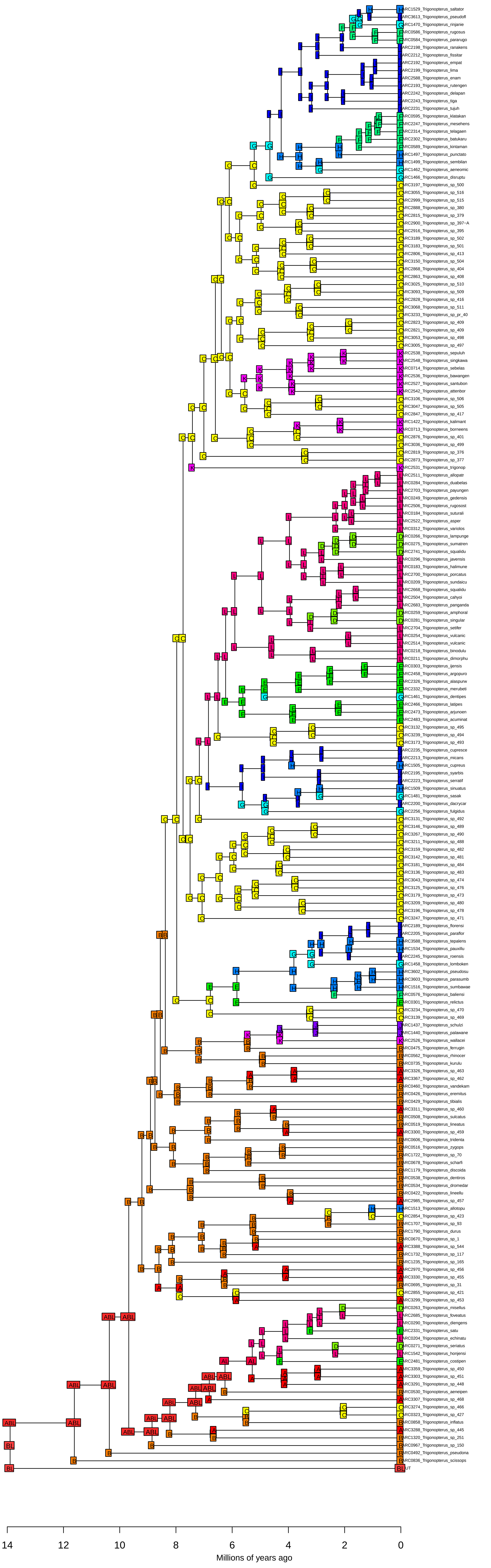

BioGeoBEARS DEC+J constraints Lower Sunda Open on Trigonopterus
ancstates: global optim, 3 areas max. d=2e−04; e=0.0015; j=0.0199; LnL=−252.09

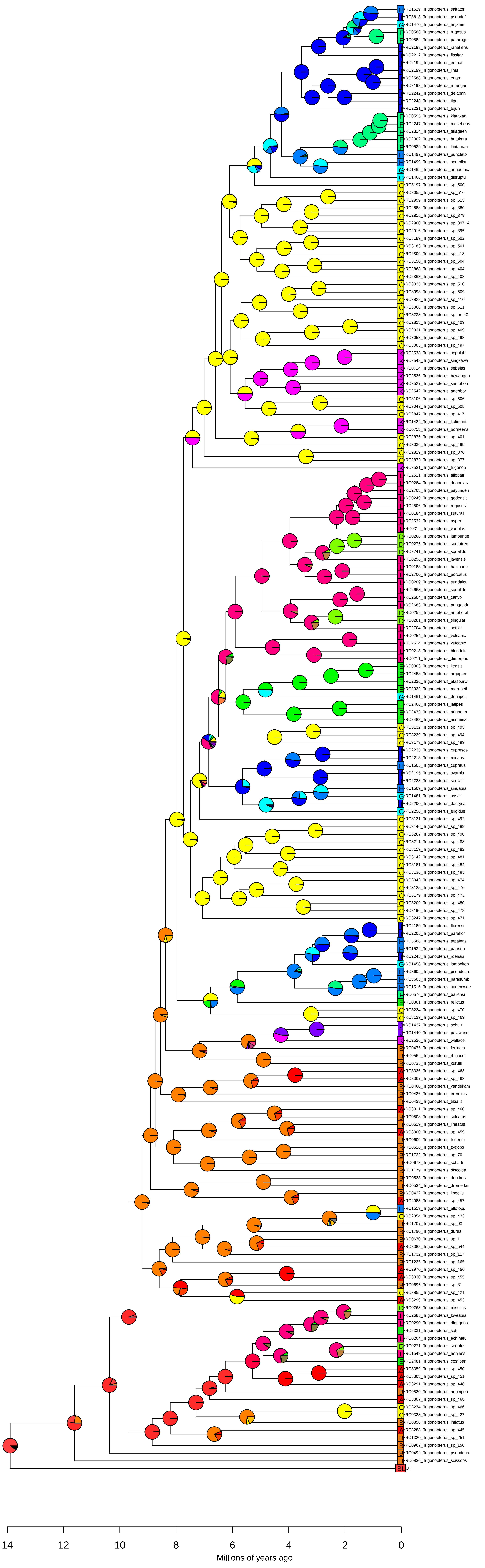

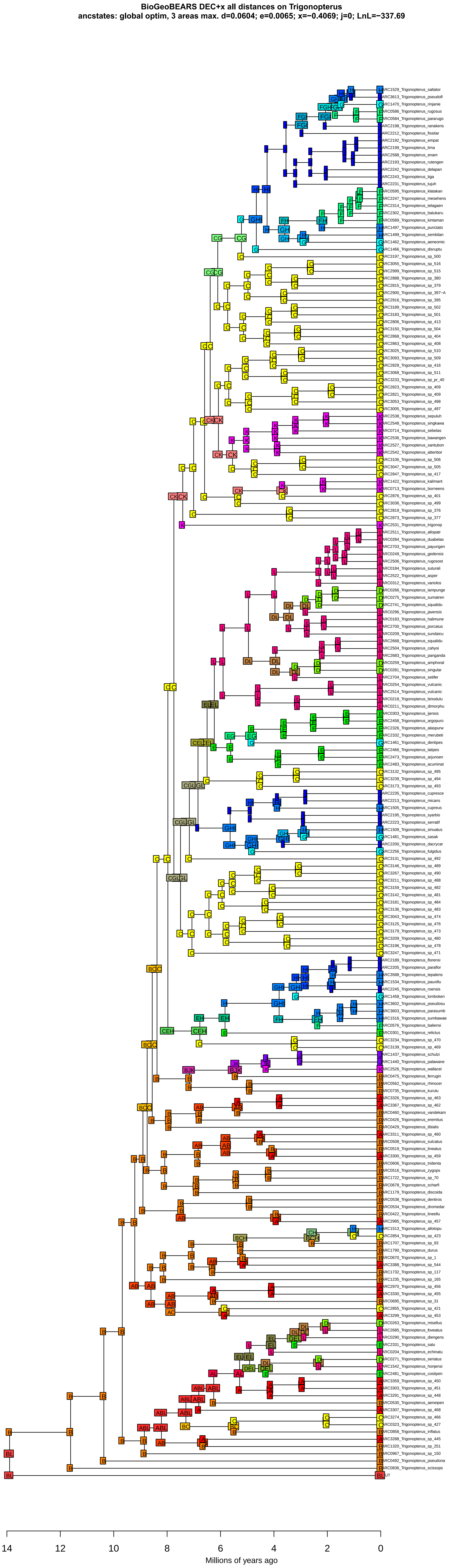
BioGeoBEARS DEC+x all distances on Trigonopterus
ancstates: global optim, 3 areas max. d=0.0604; e=0.0065; x=−0.4069; j=0; LnL=−337.69
14
12
10
8
6
4
2
0
Millions of years ago

BioGeoBEARS DEC+x all distances on Trigonopterus
ancstates: global optim, 3 areas max. d=0.0604; e=0.0065; x=−0.4069; j=0; LnL=−337.69

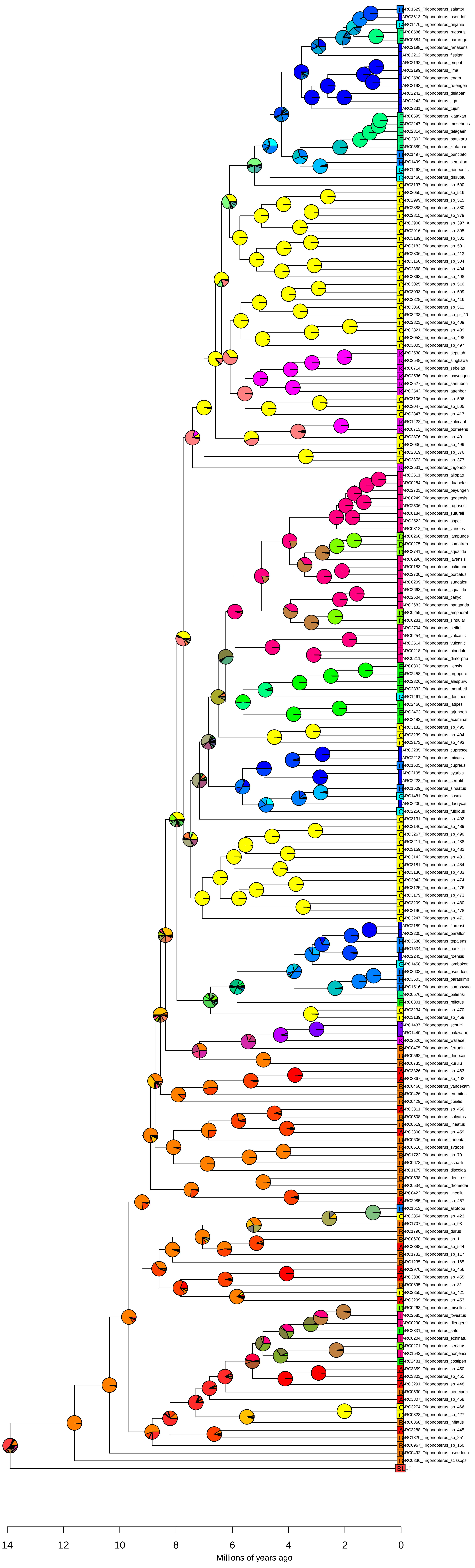

BioGeoBEARS DEC+J+x all distances on Trigonopterus
ancstates: global optim, 3 areas max. d=0; e=0; x=−0.7634; j=0.9681; LnL=−219.05

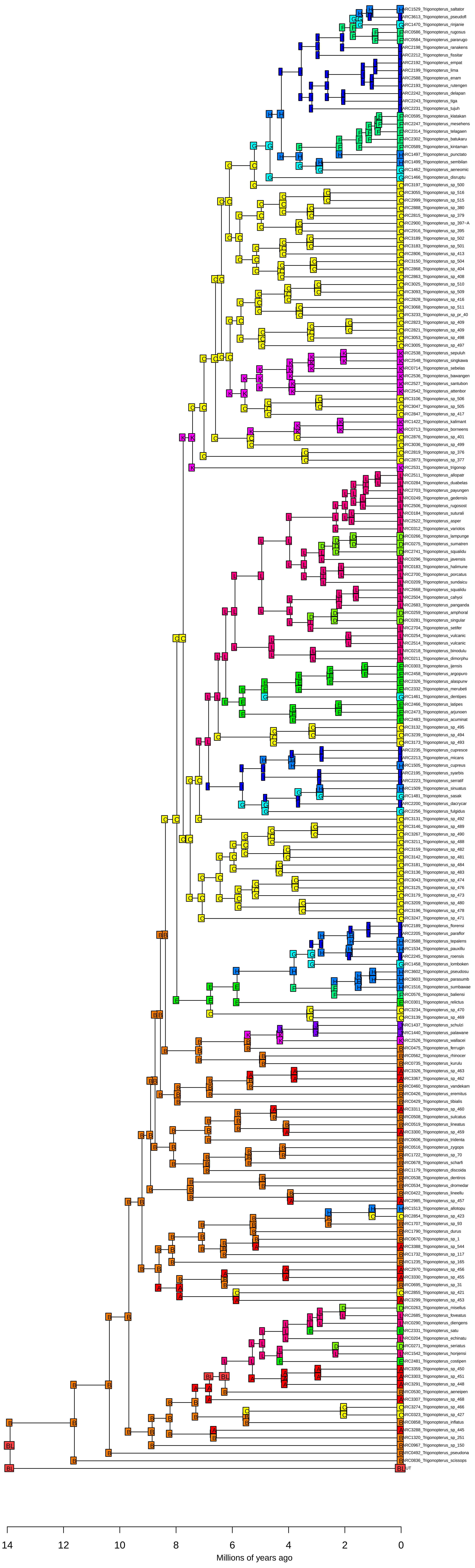

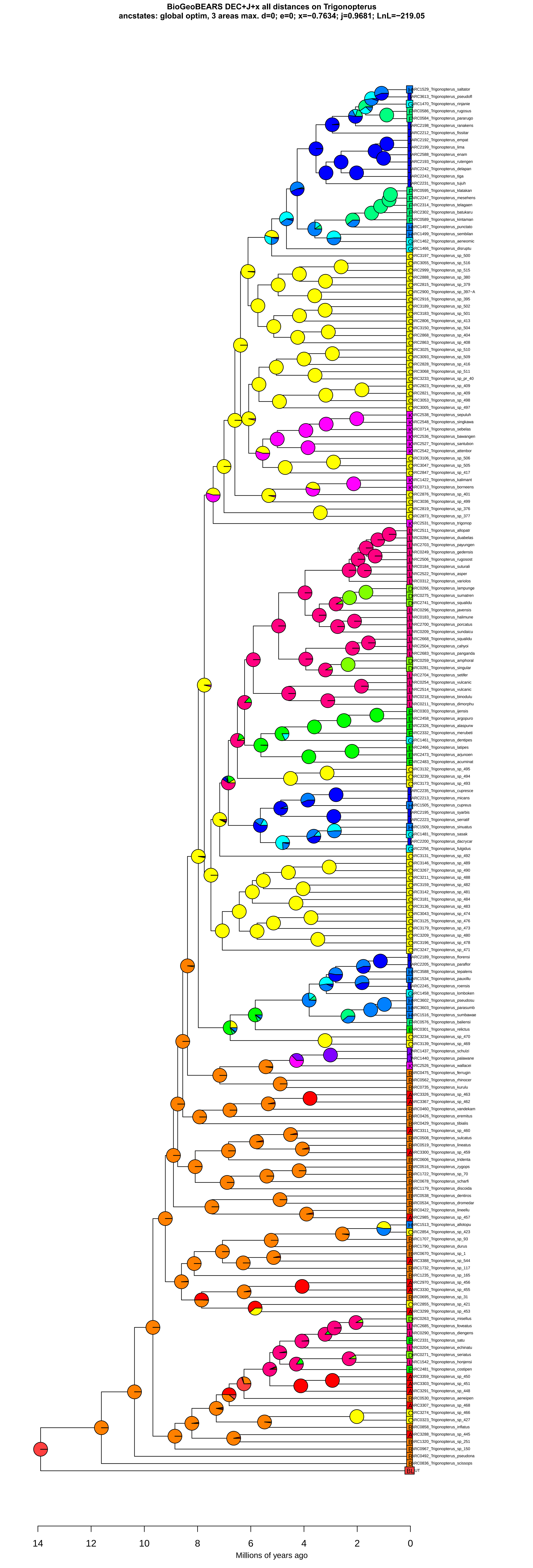
BioGeoBEARS DEC+J+x all distances on Trigonopterus
ancstates: global optim, 3 areas max. d=0; e=0; x=−0.7634; j=0.9681; LnL=−219.05
14
12
10
8
6
4
2
0
Millions of years ago

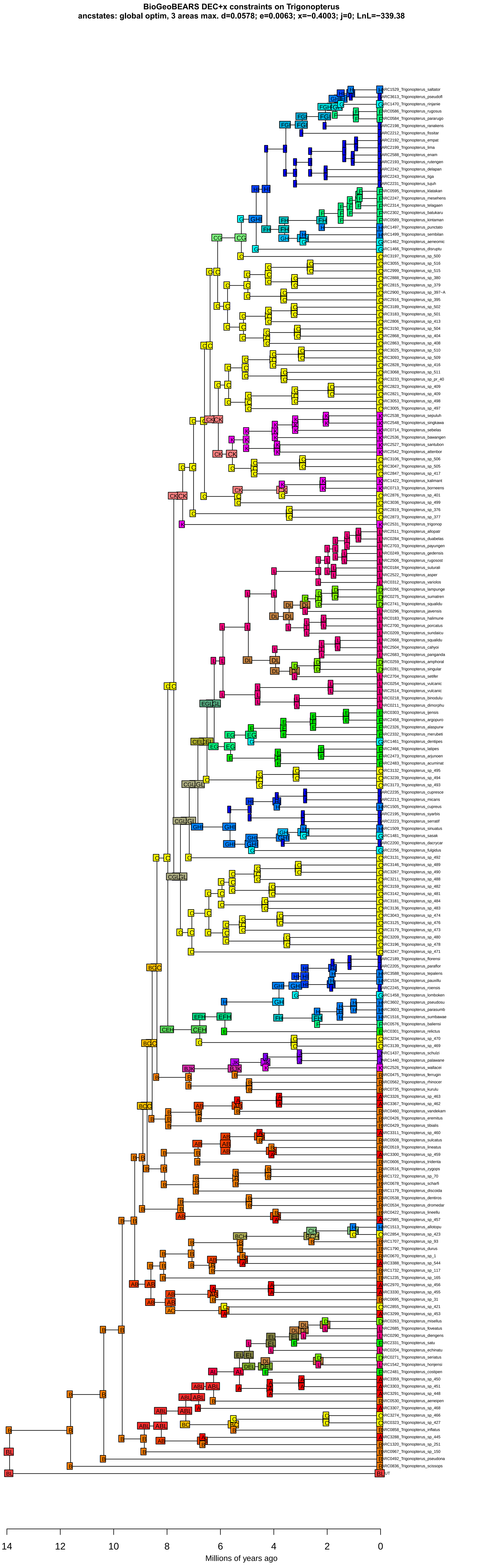
BioGeoBEARS DEC+x constraints on Trigonopterus
ancstates: global optim, 3 areas max. d=0.0578; e=0.0063; x=−0.4003; j=0; LnL=−339.38
14
12
10
8
6
4
2
0
Millions of years ago

BioGeoBEARS DEC+x constraints on Trigonopterus
ancstates: global optim, 3 areas max. d=0.0578; e=0.0063; x=−0.4003; j=0; LnL=−339.38

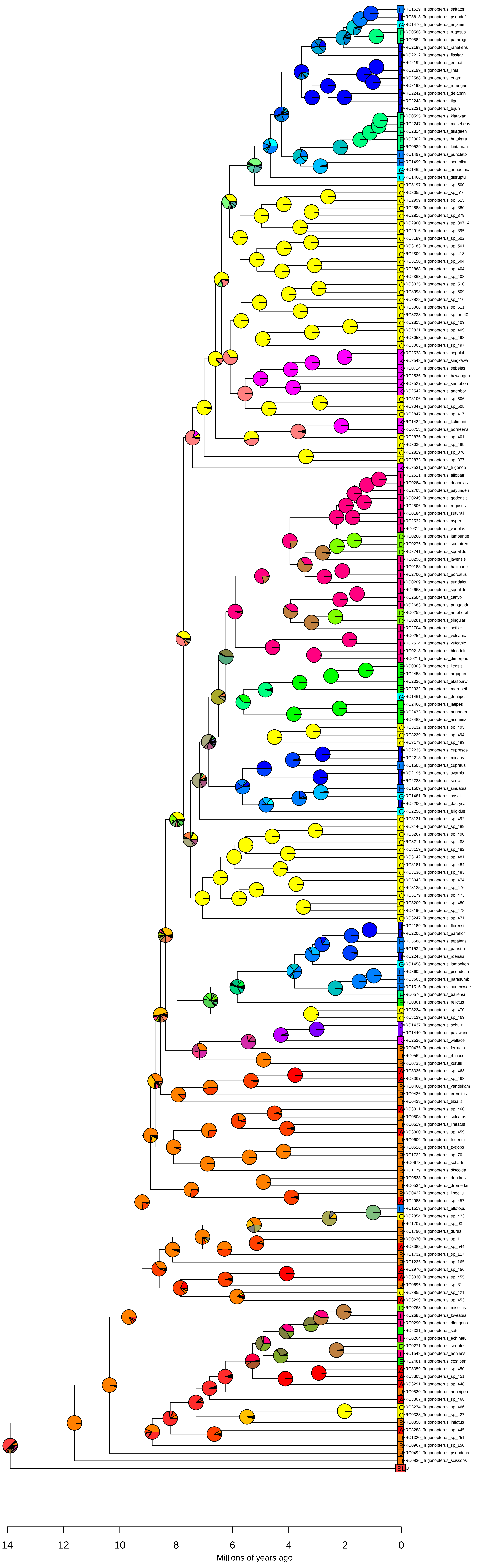

BioGeoBEARS DEC+J+x constraints on Trigonopterus
ancstates: global optim, 3 areas max. d=0; e=0; x=−0.4205; j=0.2403; LnL=−230.61

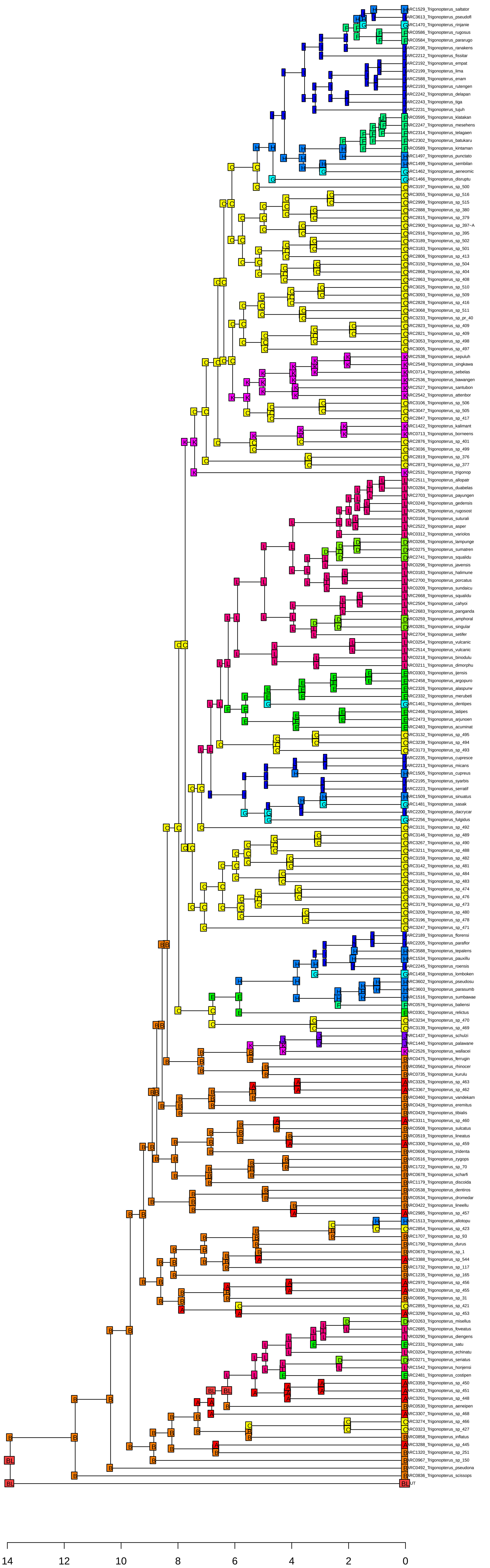

BioGeoBEARS DEC+J+x constraints on Trigonopterus
ancstates: global optim, 3 areas max. d=0; e=0; x=−0.4205; j=0.2403; LnL=−230.61

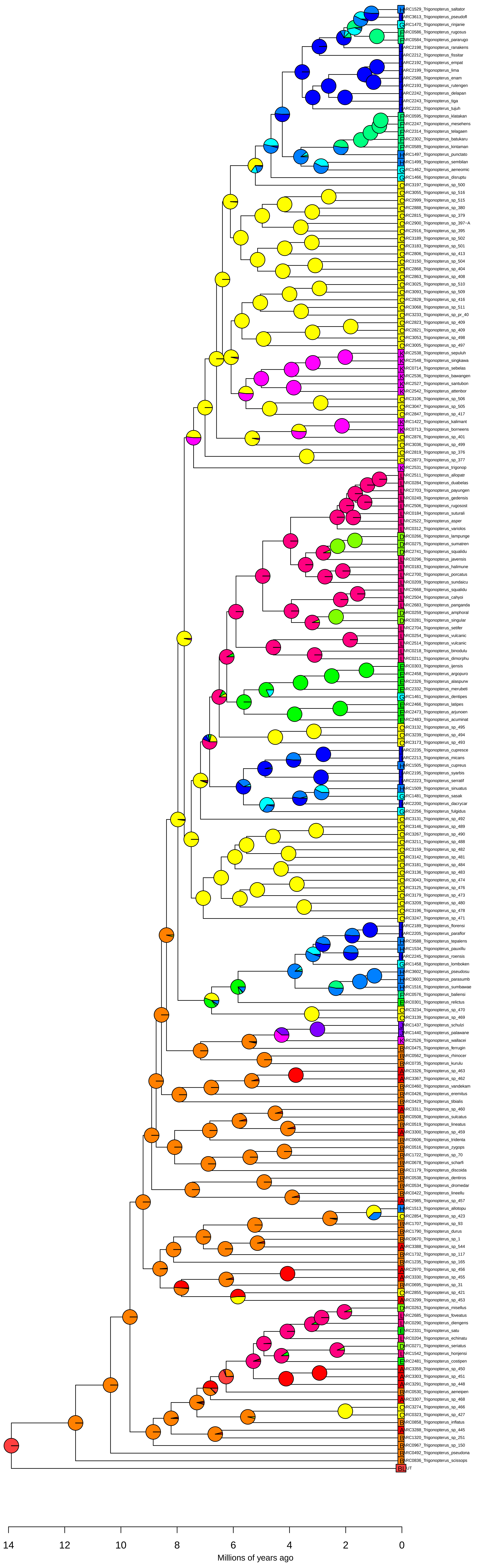

BioGeoBEARS DEC+x Lower Sunda Open on Trigonopterus
ancstates: global optim, 3 areas max. d=0.0489; e=0.0113; x=−0.3849; j=0; LnL=−335.60

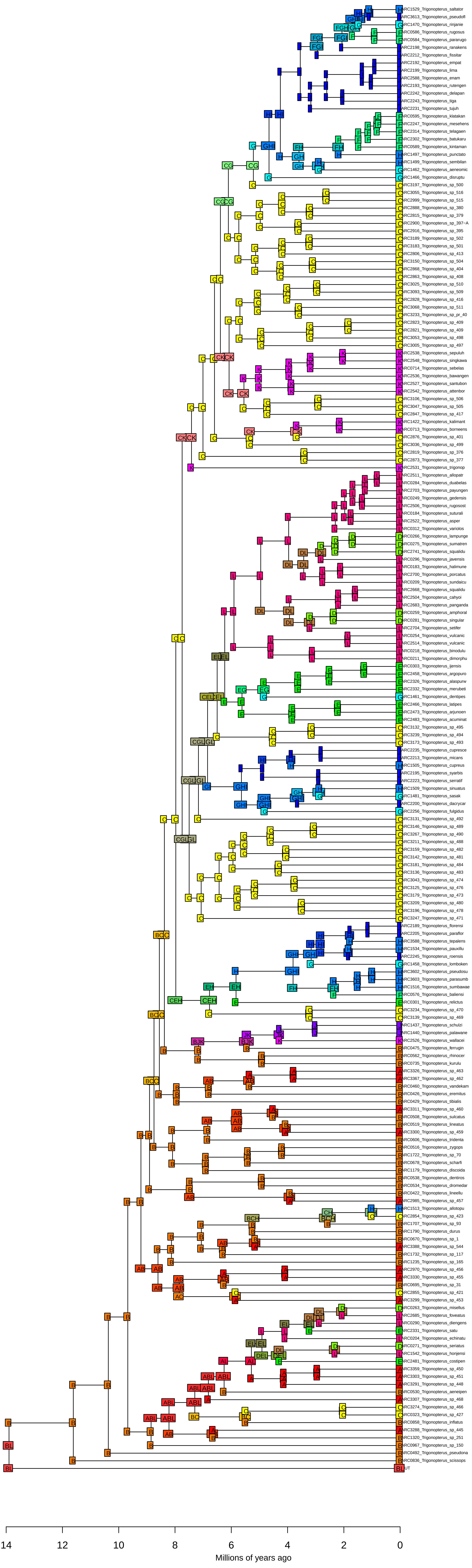

BioGeoBEARS DEC+x Lower Sunda Open on Trigonopterus
ancstates: global optim, 3 areas max. d=0.0489; e=0.0113; x=−0.3849; j=0; LnL=−335.60

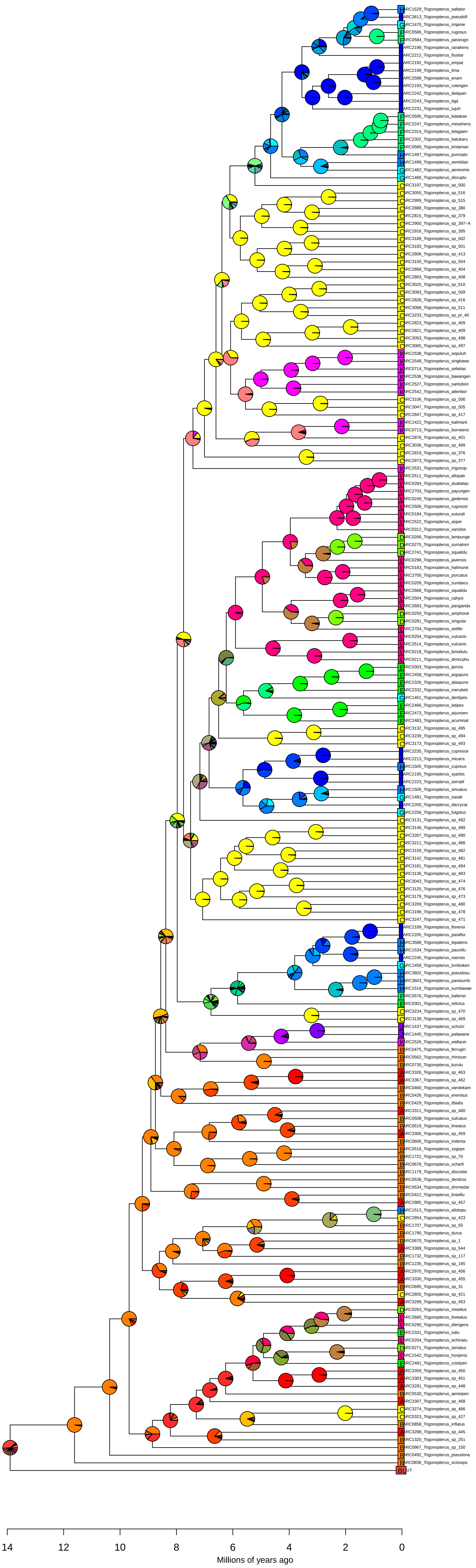

BioGeoBEARS DEC+J+x Lower Sunda Open on Trigonopterus
ancstates: global optim, 3 areas max. d=0; e=0; x=−0.9192; j=1.7344; LnL=−215.27

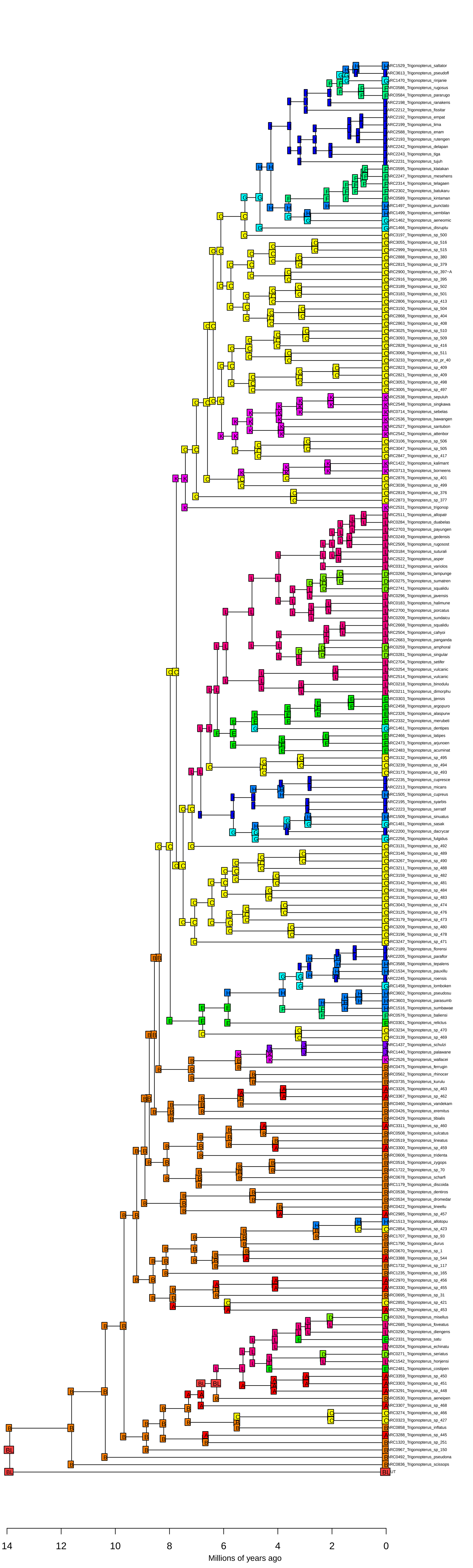

BioGeoBEARS DEC+J+x Lower Sunda Open on Trigonopterus
ancstates: global optim, 3 areas max. d=0; e=0; x=−0.9192; j=1.7344; LnL=−215.27

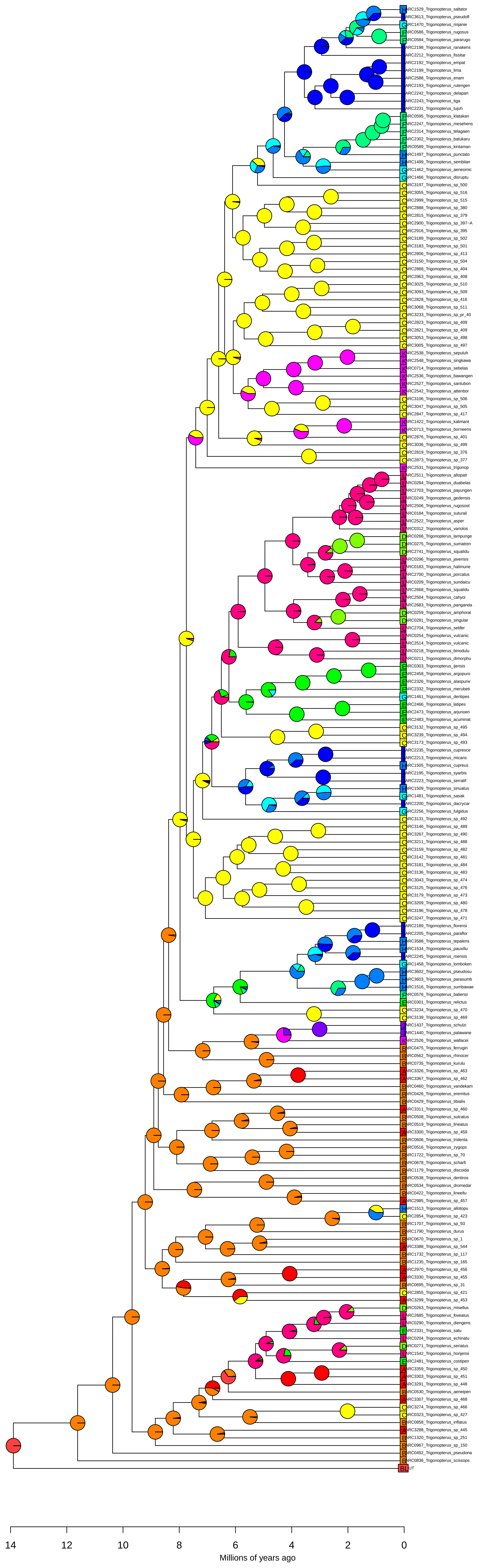

BioGeoBEARS DEC on Trigonopterus time slice
ancstates: global optim, 3 areas max. d=0.0048; e=0.0088; j=0; x=0; LnL=−361.57

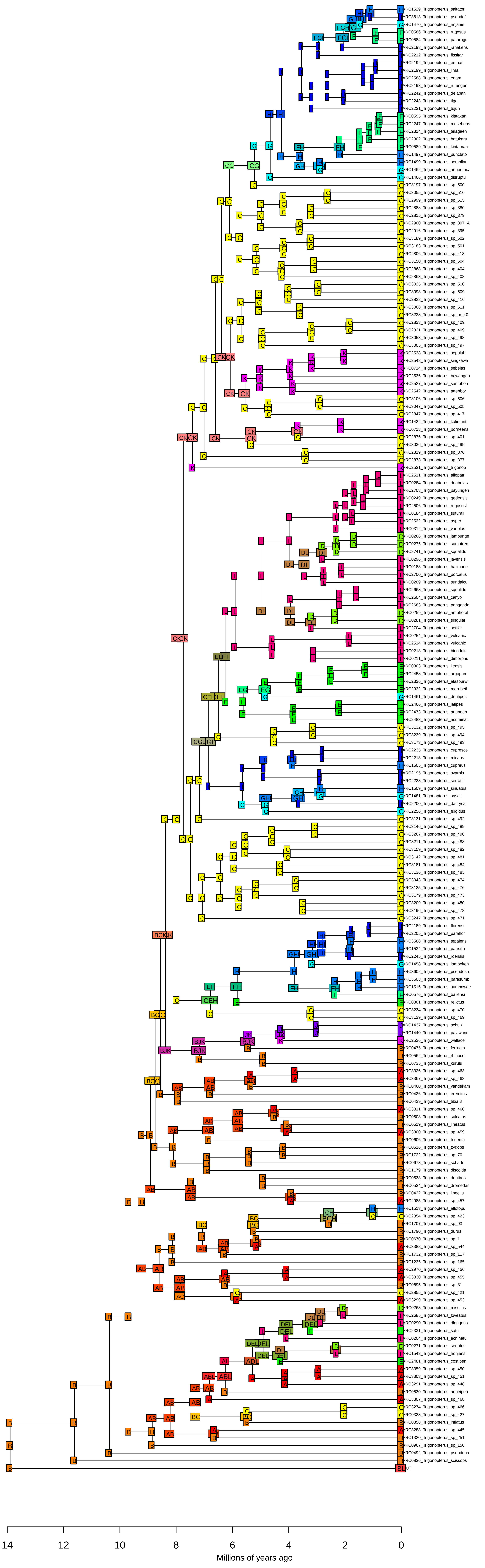

BioGeoBEARS DEC on Trigonopterus time slice
ancstates: global optim, 3 areas max. d=0.0048; e=0.0088; j=0; x=0; LnL=−361.57

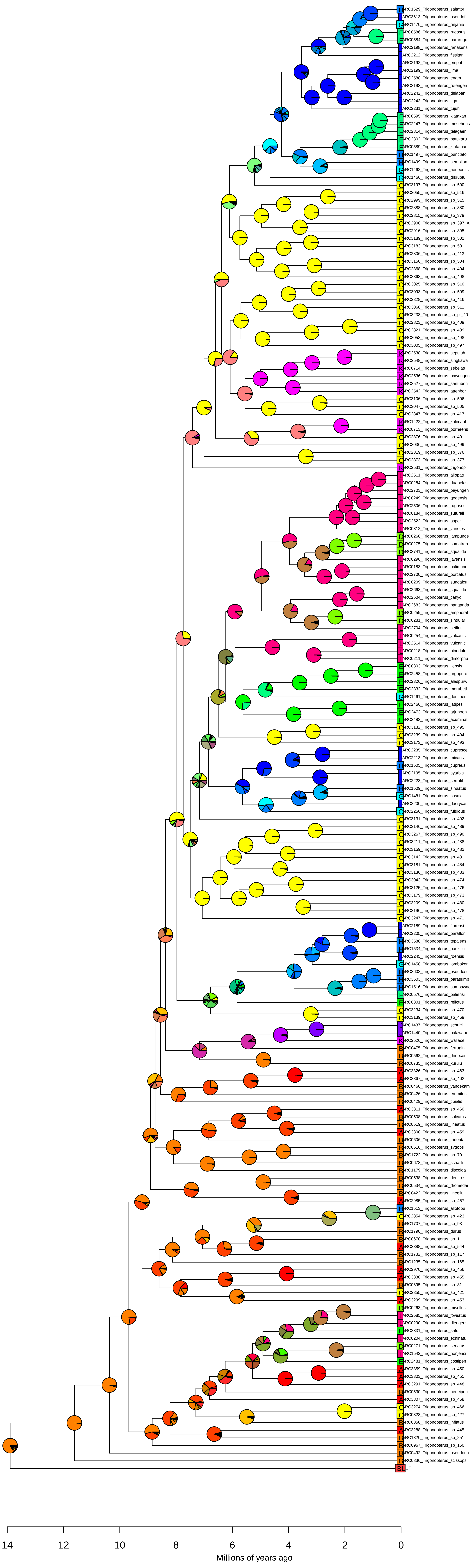

BioGeoBEARS DEC+J on Trigonopterus time slice
ancstates: global optim, 3 areas max. d=0; e=1e−04; j=0.017; x=0; LnL=−258.01

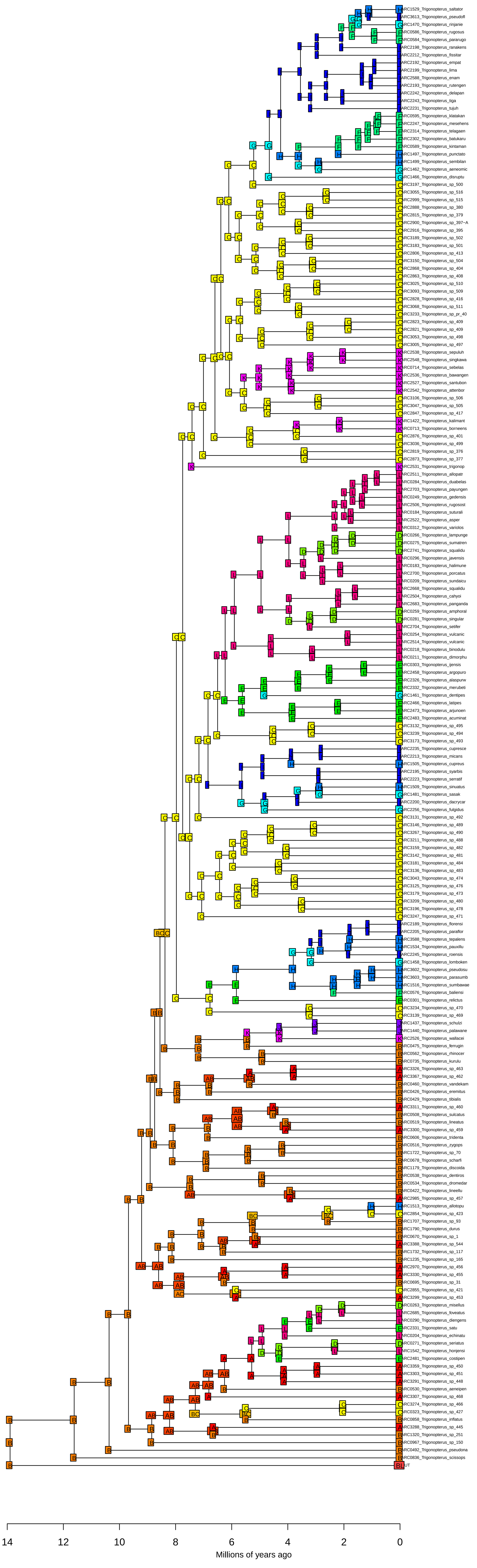

BioGeoBEARS DEC+J on Trigonopterus time slice
ancstates: global optim, 3 areas max. d=0; e=1e−04; j=0.017; x=0; LnL=−258.01

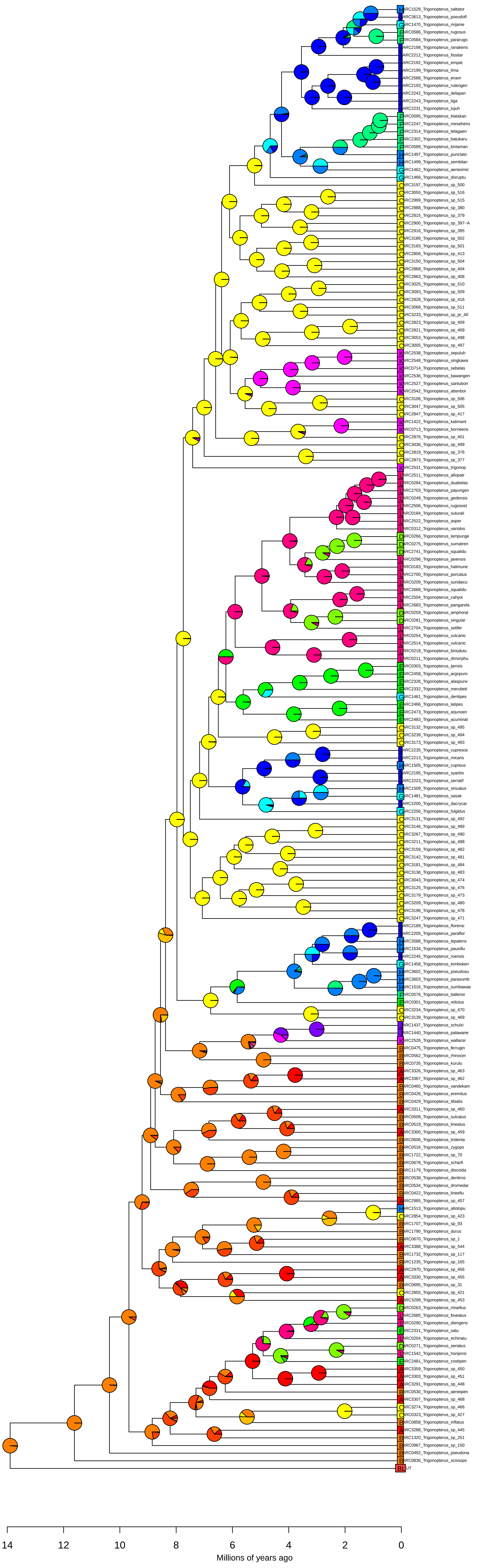

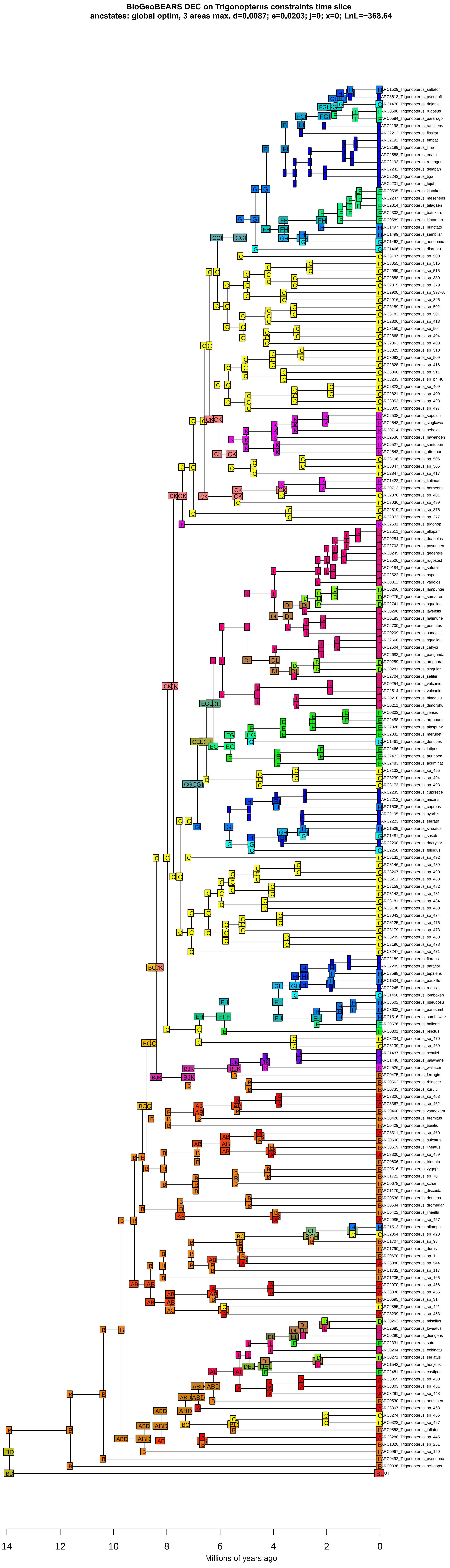
BioGeoBEARS DEC on Trigonopterus constraints time slice
ancstates: global optim, 3 areas max. d=0.0087; e=0.0203; j=0; x=0; LnL=−368.64
14
12
10
8
6
4
2
0
Millions of years ago

BioGeoBEARS DEC on Trigonopterus constraints time slice
ancstates: global optim, 3 areas max. d=0.0087; e=0.0203; j=0; x=0; LnL=−368.64

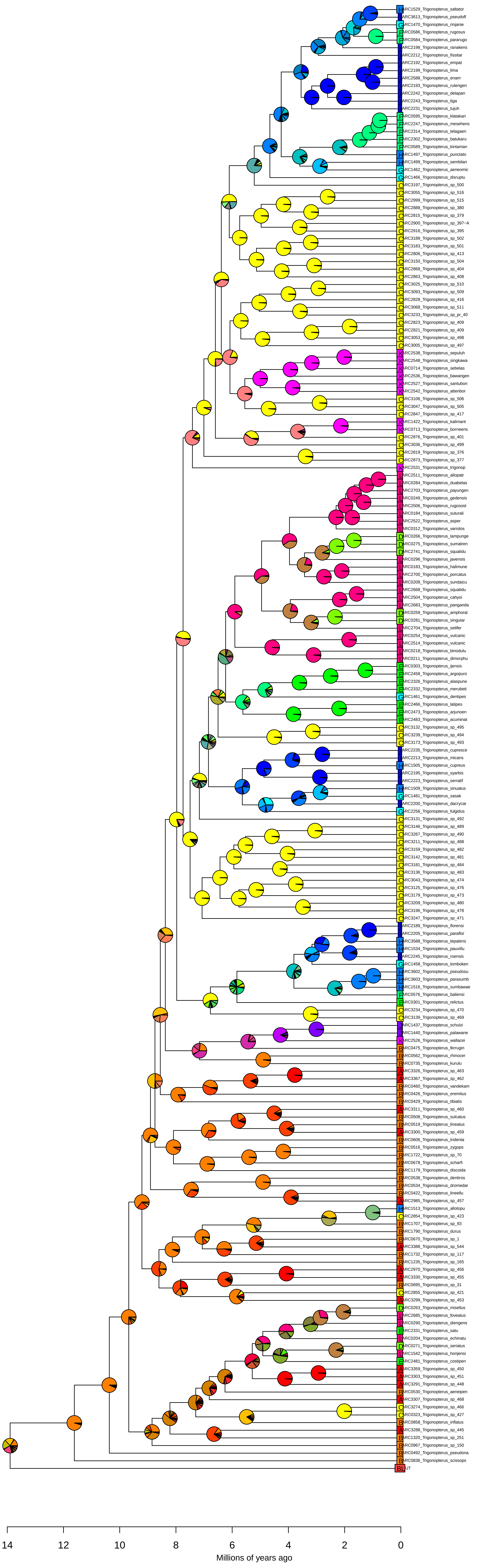

BioGeoBEARS DEC+J on Trigonopterus constraints time slice
ancstates: global optim, 3 areas max. d=0.0128; e=0.079; j=0.9942; x=0; LnL=-567.44

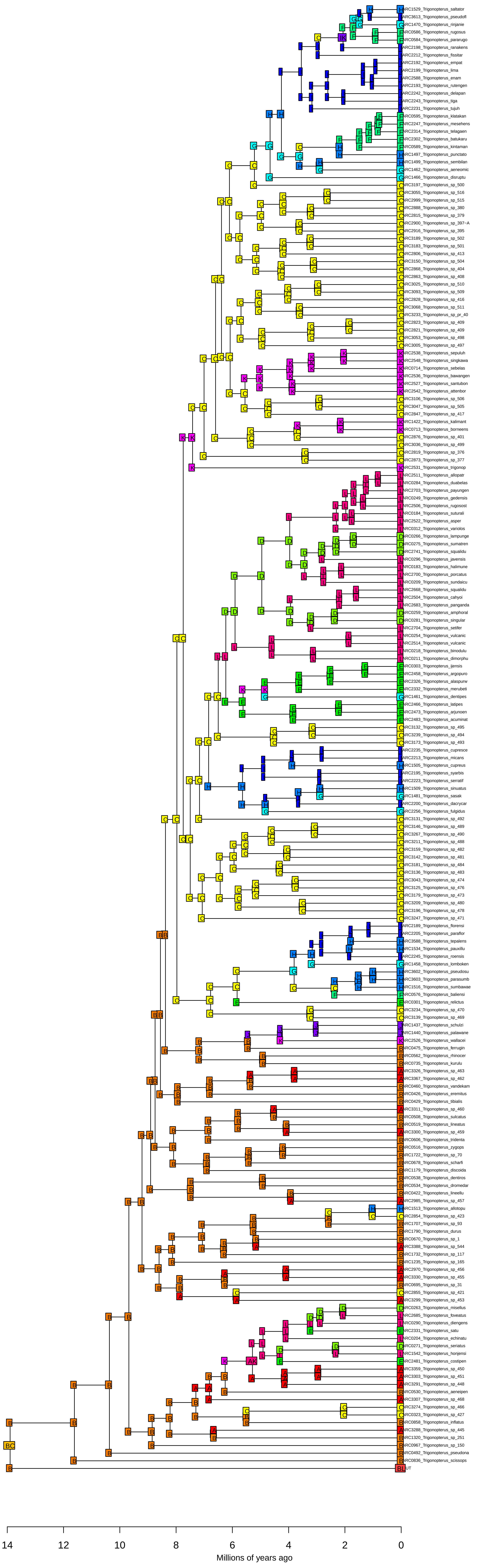

BioGeoBEARS DEC+J on Trigonopterus constraints time slice
ancstates: global optim, 3 areas max. d=0.0128; e=0.079; j=0.9942; x=0; LnL=-567.44

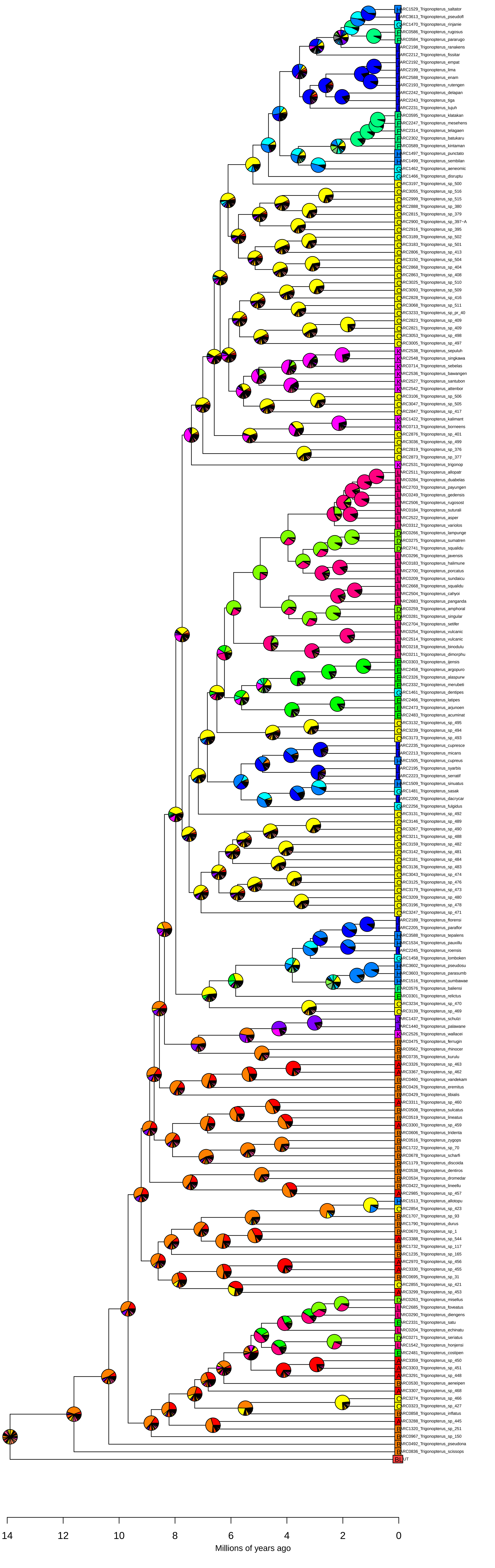

BioGeoBEARS DEC on Trigonopterus Lower Sunda Open time slice
ancstates: global optim, 3 areas max. d=0.0073; e=0.0146; j=0; x=0; LnL=−356.81

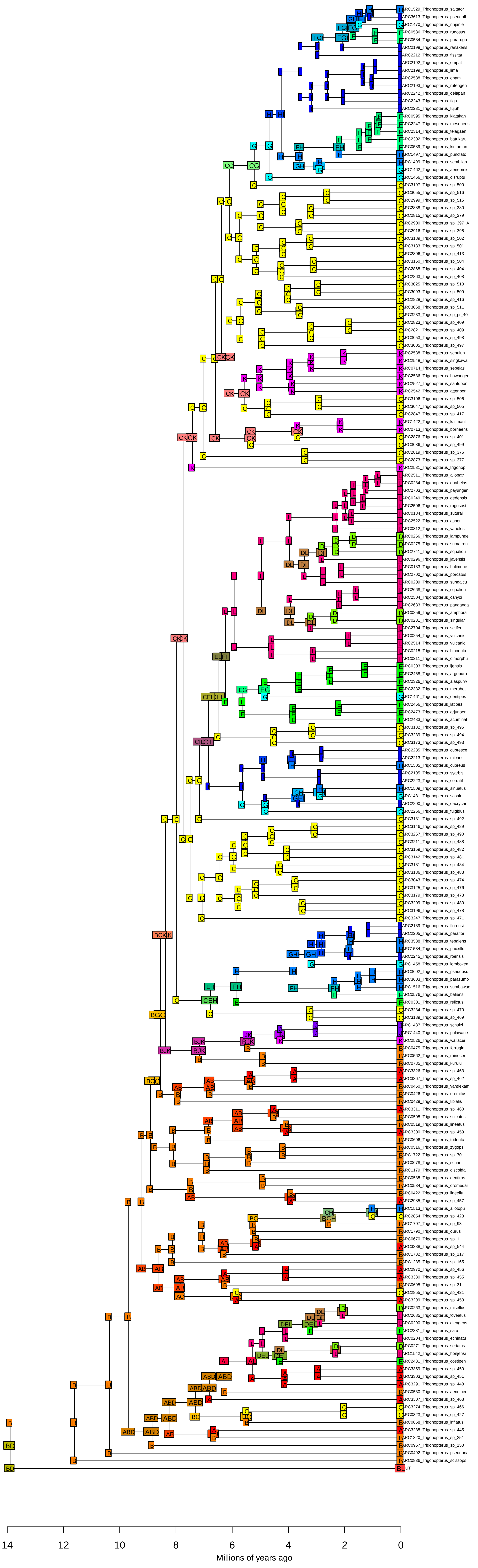

BioGeoBEARS DEC on Trigonopterus Lower Sunda Open time slice
ancstates: global optim, 3 areas max. d=0.0073; e=0.0146; j=0; x=0; LnL=-356.81

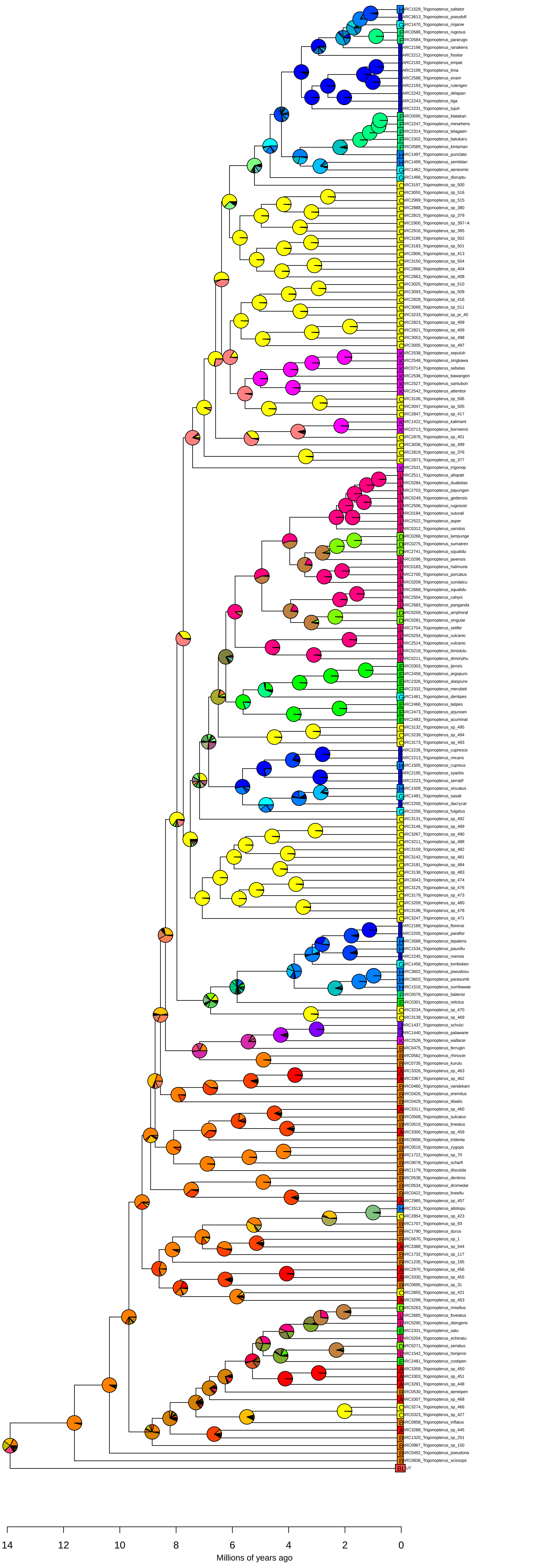

BioGeoBEARS DEC+J on Trigonopterus Lower Sunda Open time slice
ancstates: global optim, 3 areas max. d=0.0149; e=0.0776; j=0.9983; x=0; LnL=−599.52

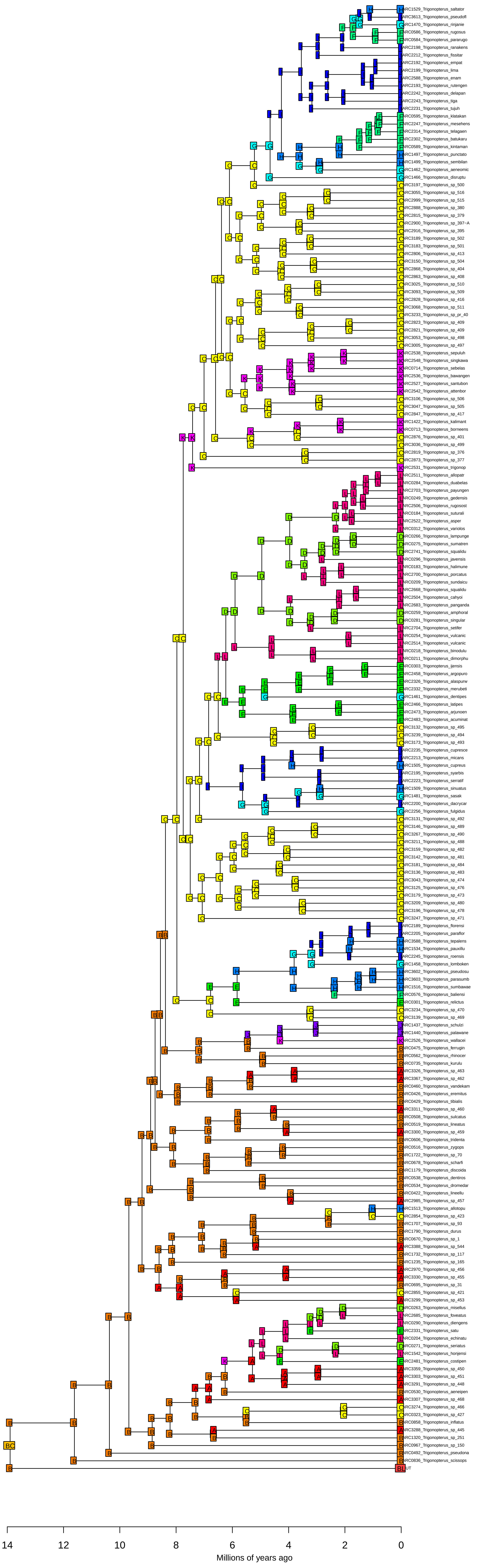

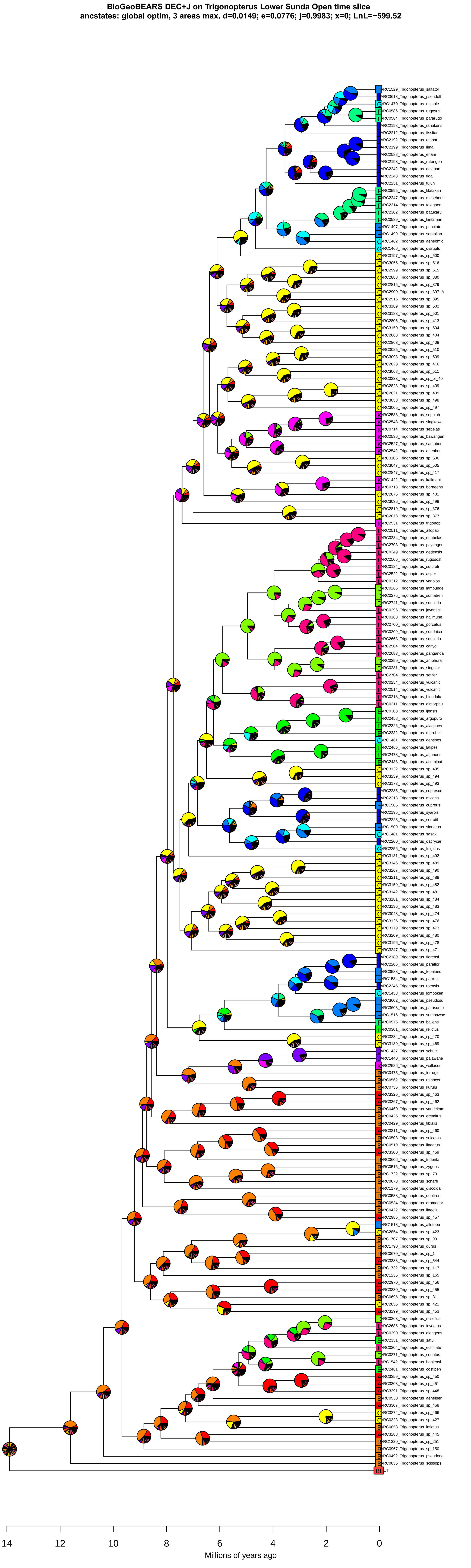
BioGeoBEARS DEC+J on Trigonopterus Lower Sunda Open time slice
ancstates: global optim, 3 areas max. d=0.0149; e=0.0776; j=0.9983; x=0; LnL=-599.52
14
12
10
8
6
4
2
0
Millions of years ago

BioGeoBEARS DEC+x on Trigonopterus allopen time slice
ancstates: global optim, 3 areas max. d=0.0295; e=0.0084; x=−0.2801; j=0; LnL=−348.40

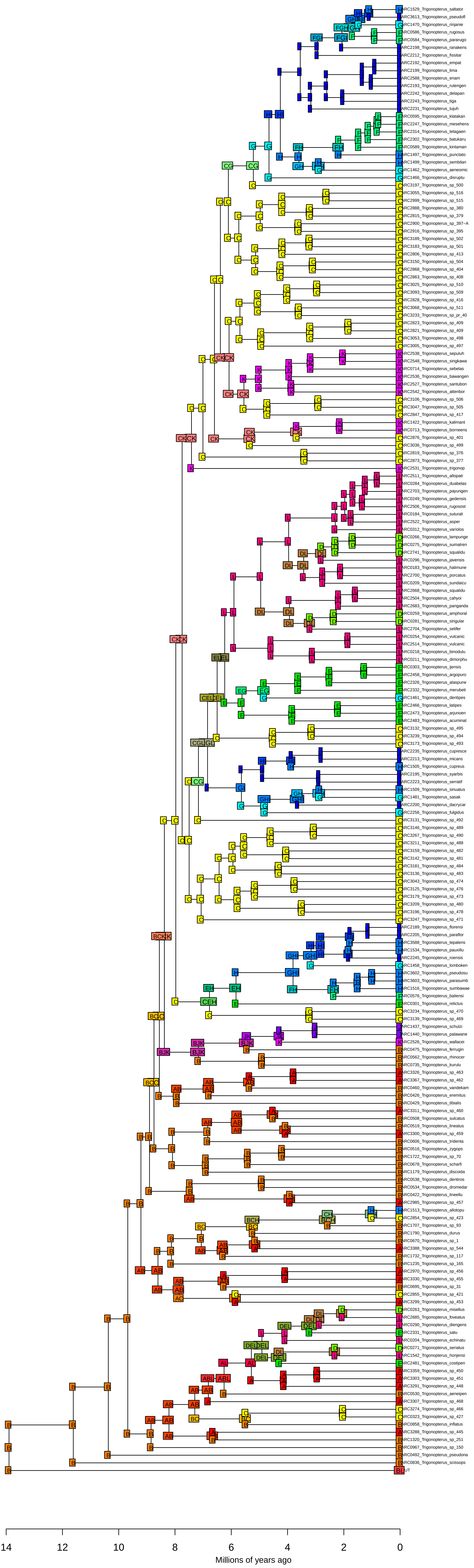

BioGeoBEARS DEC+x on Trigonopterus allopen time slice
ancstates: global optim, 3 areas max. d=0.0295; e=0.0084; x=−0.2801; j=0; LnL=−348.40

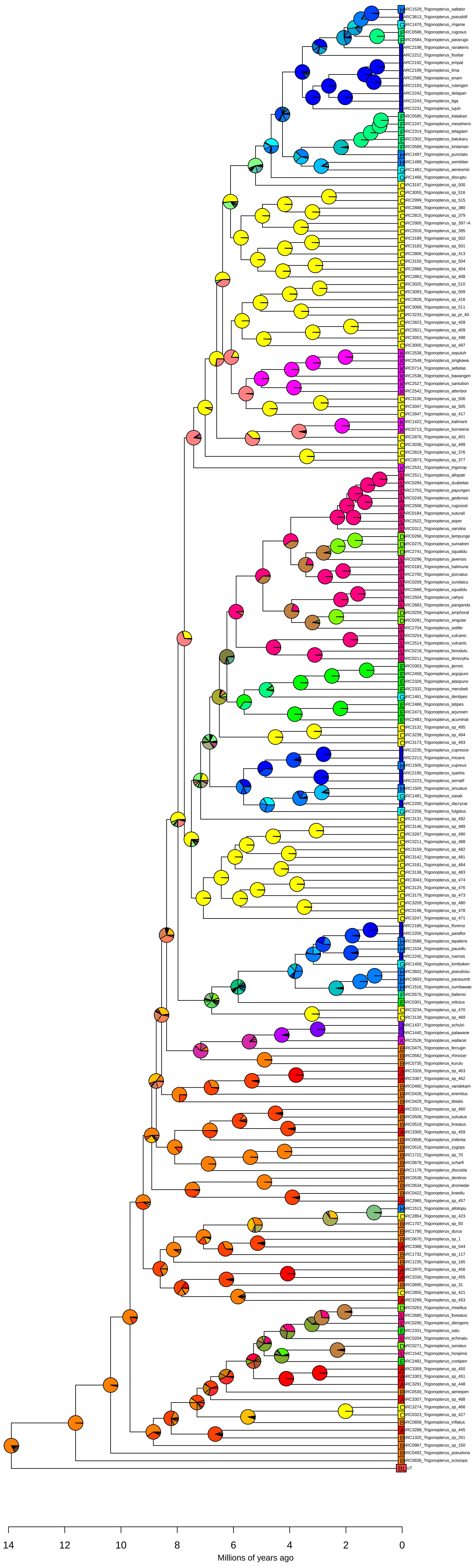

BioGeoBEARS DEC+J+x on Trigonopterus allopen time slice
ancstates: global optim, 3 areas max. d=0.0091; e=0; x=−0.712; j=0.9787; LnL=−233.23

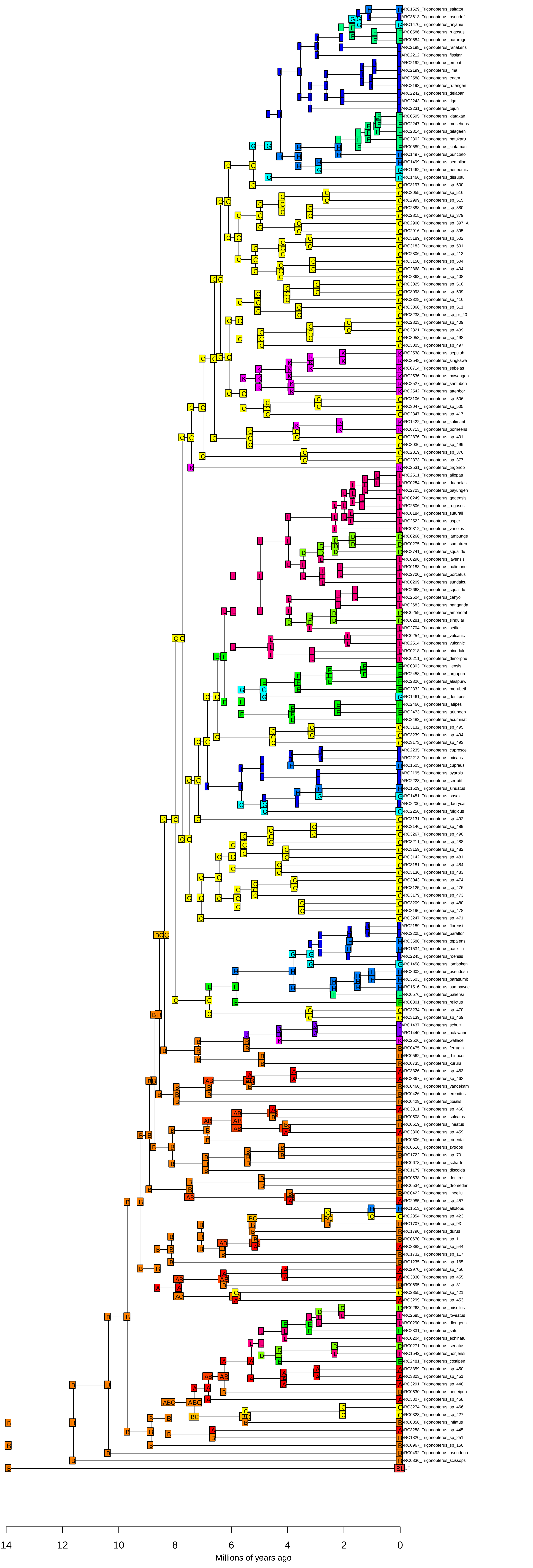

BioGeoBEARS DEC+J+x on Trigonopterus allopen time slice
ancstates: global optim, 3 areas max. d=0.0091; e=0; x=−0.712; j=0.9787; LnL=−233.23

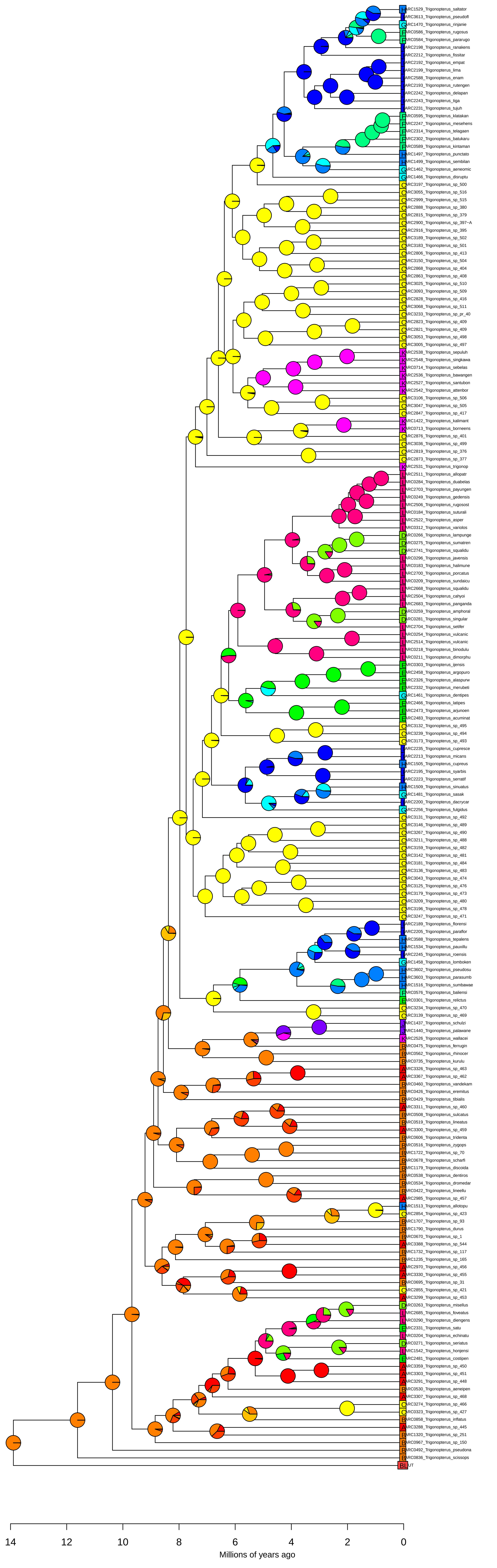

BioGeoBEARS DEC+x on Trigonopterus constraints time slice
ancstates: global optim, 3 areas max. d=0.0419; e=0.0092; x=−0.3109; j=0; LnL=−347.29

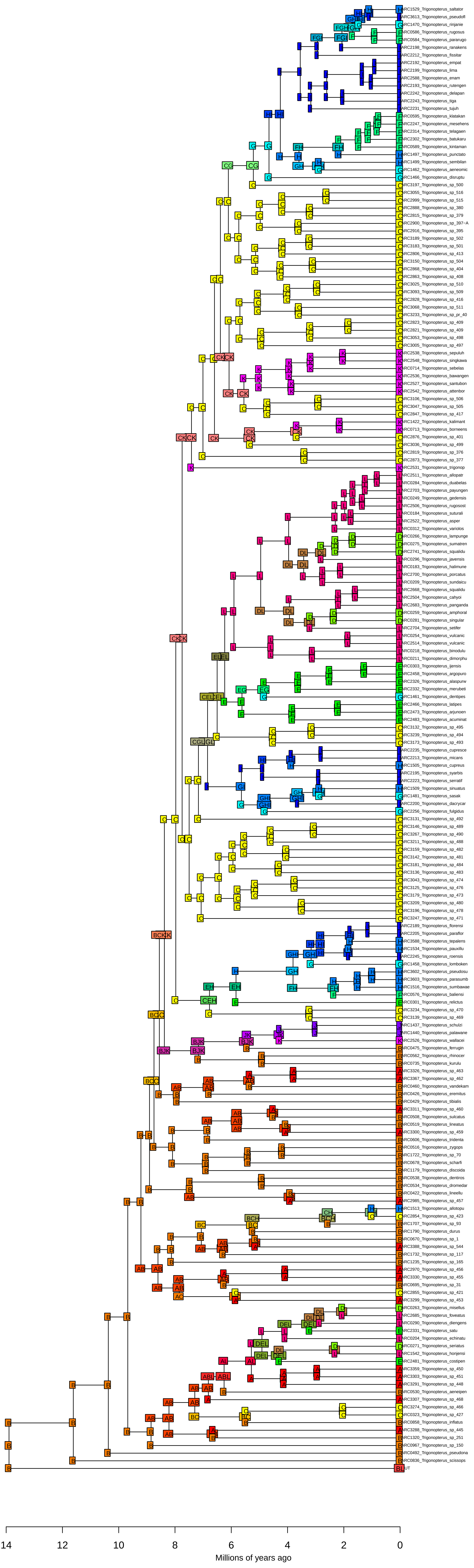

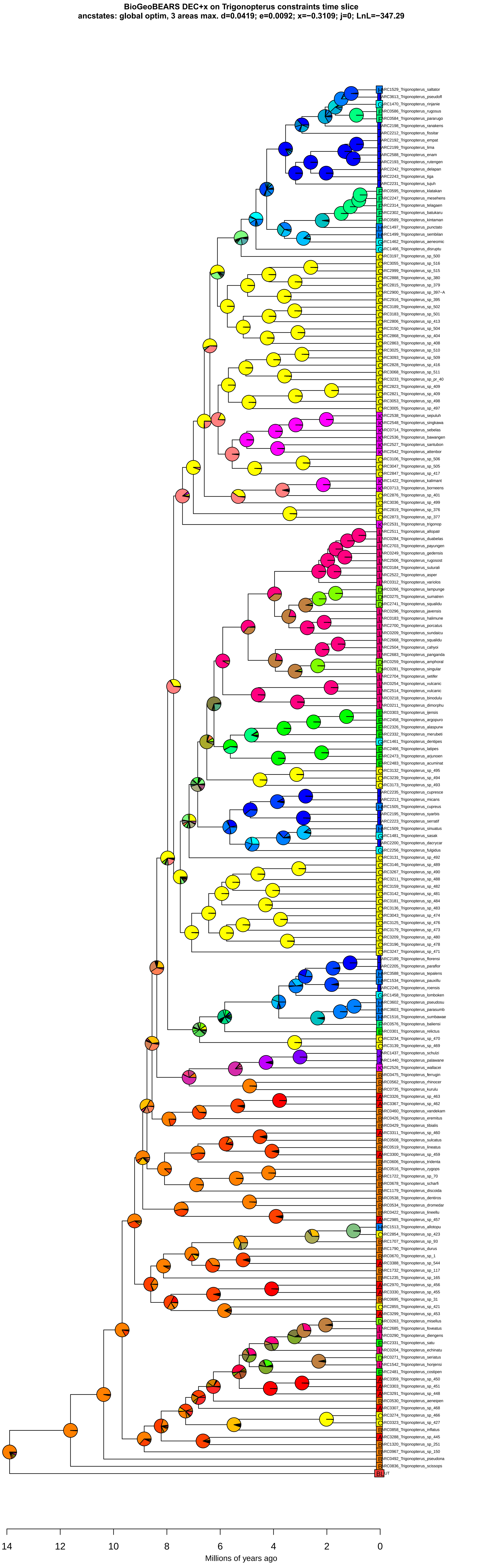
BioGeoBEARS DEC+x on Trigonopterus constraints time slice
ancstates: global optim, 3 areas max. d=0.0419; e=0.0092; x=−0.3109; j=0; LnL=−347.29
14
12
10
8
6
4
2
0
Millions of years ago

BioGeoBEARS DEC+J+x on Trigonopterus constraints time slice
ancstates: global optim, 3 areas max. d=0.0072; e=0; x=−0.6194; j=0.8209; LnL=−239.65

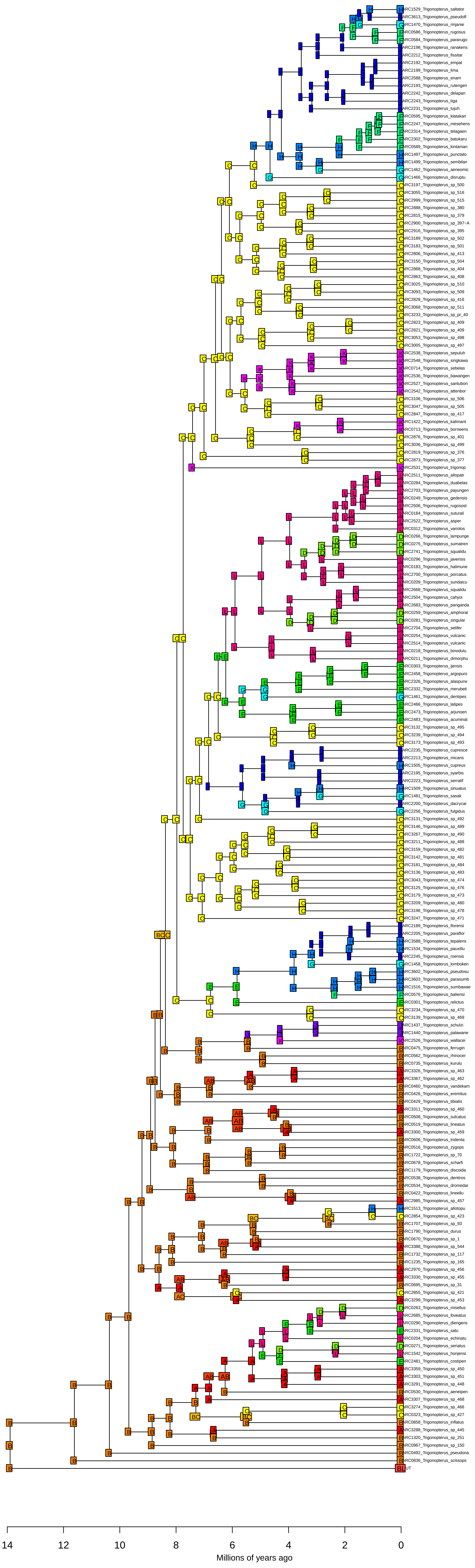

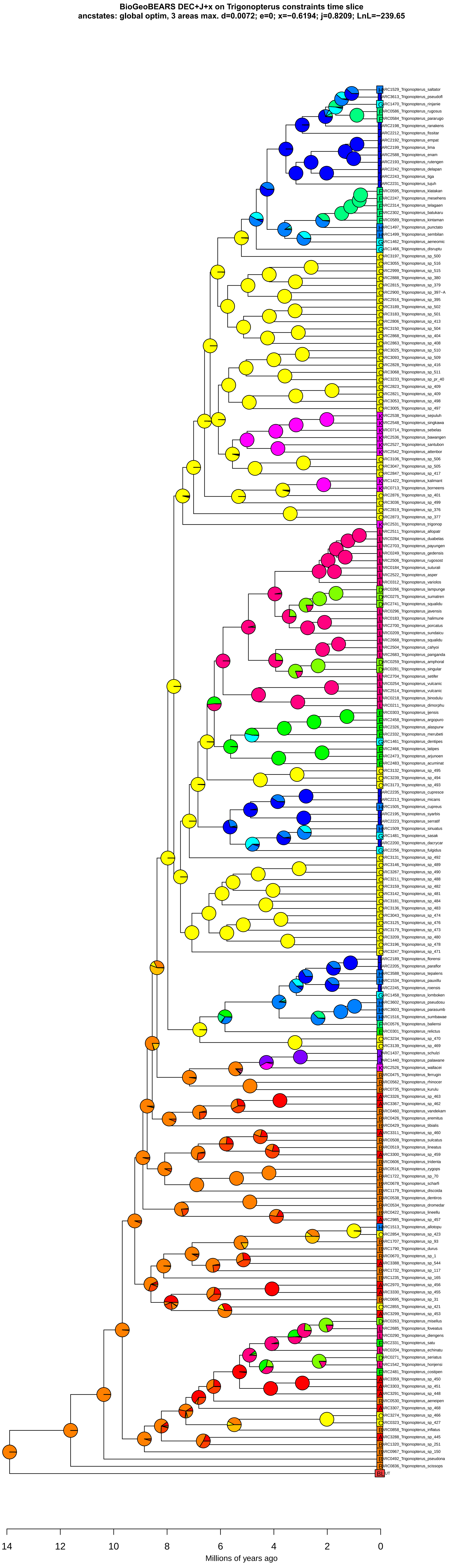
BioGeoBEARS DEC+J+x on Trigonopterus constraints time slice
ancstates: global optim, 3 areas max. d=0.0072; e=0; x=−0.6194; j=0.8209; LnL=−239.65
14
12
10
8
6
4
2
0
Millions of years ago

BioGeoBEARS DEC+x on Trigonopterus Lower Sunda Open time slice
ancstates: global optim, 3 areas max. d=0.2; e=0.0106; x=−0.5825; j=0; LnL=−337.87

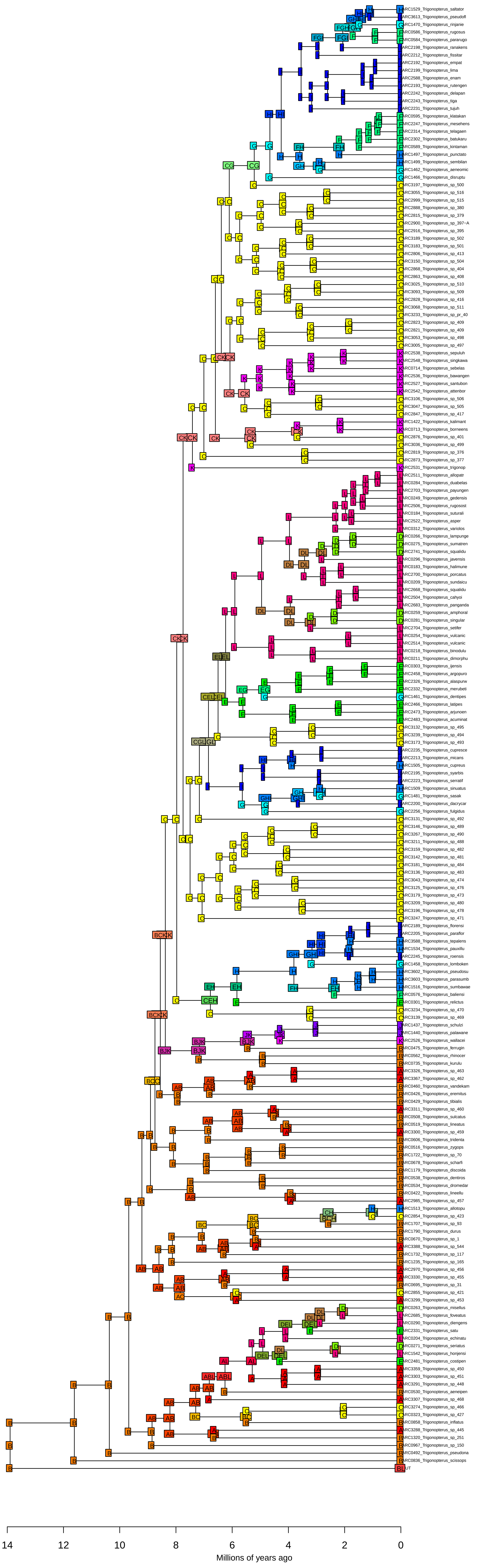

BioGeoBEARS DEC+x on Trigonopterus Lower Sunda Open time slice
ancstates: global optim, 3 areas max. d=0.2; e=0.0106; x=−0.5825; j=0; LnL=−337.87

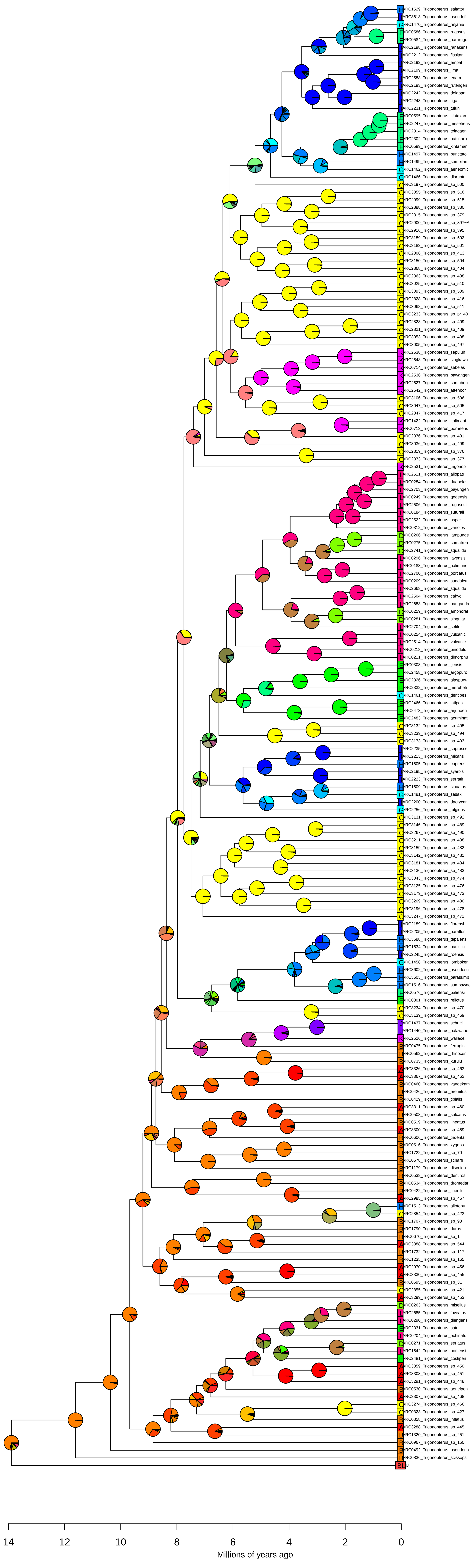

BioGeoBEARS DEC+J+x on Trigonopterus Lower Sunda Open time slice
ancstates: global optim, 3 areas max. d=0.0153; e=0; x=−0.7622; j=1.284; LnL=−230.17

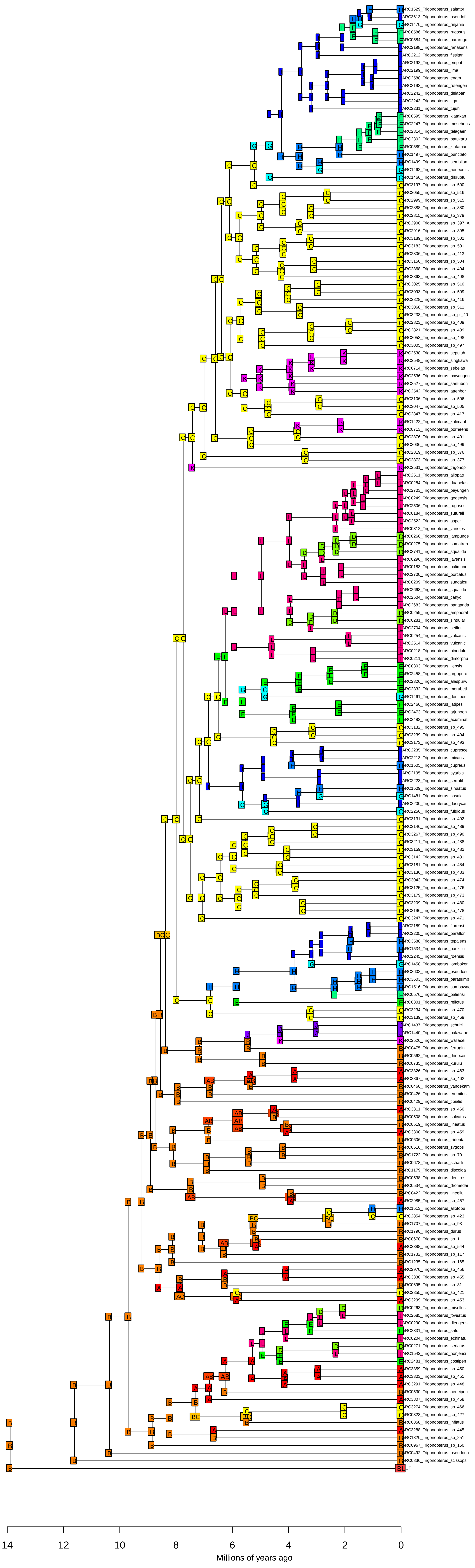

BioGeoBEARS DEC+J+x on Trigonopterus Lower Sunda Open time slice
ancstates: global optim, 3 areas max. d=0.0153; e=0; x=−0.7622; j=1.284; LnL=−230.17

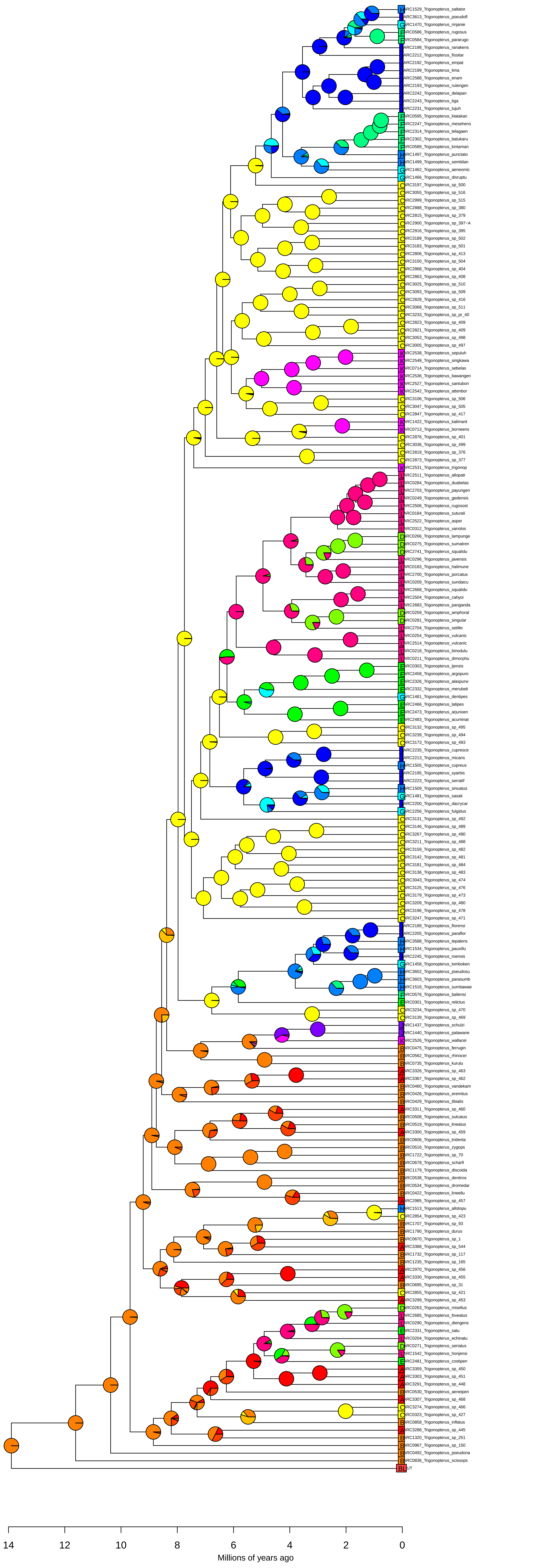

Time Periods (2, 5, 10, 15, 20, 45Ma)

Areas allowed

Maluku	New Guinea		Sulawesi		Sumatra	E-Java	Bali	Lomboc	Sumbawa	Flores	Philippines	Borneo	W-Java
1	1	1	1	1	1	1	1	1	1	1	1		
1	1	1	1	1	1	1	1	1	1	1	1		
1	1	1	1	1	1	1	1	1	1	1	1		
1	1	1	1	1	1	1	1	1	1	1	1		
1	1	1	1	1	1	1	1	1	1	1	1		
1	1	1	1	1	1	1	1	1	1	1	1		
1	1	1	1	1	1	1	1	1	1	1	1		
1	1	1	1	1	1	1	1	1	1	1	1		
1	1	1	1	1	1	1	1	1	1	1	1		
1	1	1	1	1	1	1	1	1	1	1	1		
1	1	1	1	1	1	1	1	1	1	1	1		
1	1	1	1	1	1	1	1	1	1	1	1		

Maluku	New Guinea		Sulawesi		Sumatra	E-Java	Bali	Lomboc	Sumbawa	Flores	Philippines	Borneo	W-Java
1	1	1	1	1	1	1	1	1	1	1	1		
1	1	1	1	1	1	1	1	1	1	1	1		
1	1	1	1	1	1	1	1	1	1	1	1		
1	1	1	1	1	1	1	1	1	1	1	1		
1	1	1	1	1	1	1	1	1	1	1	1		
1	1	1	1	1	1	1	1	1	1	1	1		
1	1	1	1	1	1	1	1	1	1	1	1		
1	1	1	1	1	1	1	1	1	1	1	1		
1	1	1	1	1	1	1	1	1	1	1	1		
1	1	1	1	1	1	1	1	1	1	1	1		
1	1	1	1	1	1	1	1	1	1	1	1		
1	1	1	1	1	1	1	1	1	1	1	1		

Maluku	New Guinea		Sulawesi		Sumatra	E-Java	Bali	Lomboc	Sumbawa	Flores	Philippines	Borneo	W-Java
1	1	1	1	1	0	0	1	1	1	1	1		
1	1	1	1	1	0	0	1	1	1	1	1		
1	1	1	1	1	0	0	1	1	1	1	1		
1	1	1	1	1	0	0	1	1	1	1	1		
1	1	1	1	1	0	0	1	1	1	1	1		
0	0	0	0	0	0	0	0	0	0	0	0		
0	0	0	0	0	0	0	0	0	0	0	0		
1	1	1	1	1	0	0	1	1	1	1	1		
1	1	1	1	1	0	0	1	1	1	1	1		
1	1	1	1	1	0	0	1	1	1	1	1		
1	1	1	1	1	0	0	1	1	1	1	1		
1	1	1	1	1	0	0	1	1	1	1	1		

Maluku	New Guinea		Sulawesi		Sumatra	E-Java	Bali	Lomboc	Sumbawa	Flores	Philippines	Borneo	W-Java
1	1	1	1	0	0	0	0	0	1	1	0		
1	1	1	1	0	0	0	0	0	1	1	0		
1	1	1	1	0	0	0	0	0	1	1	0		
1	1	1	1	0	0	0	0	0	1	1	0		
0	0	0	0	0	0	0	0	0	0	0	0		
0	0	0	0	0	0	0	0	0	0	0	0		
0	0	0	0	0	0	0	0	0	0	0	0		
0	0	0	0	0	0	0	0	0	0	0	0		
0	0	0	0	0	0	0	0	0	0	0	0		
1	1	1	1	0	0	0	0	0	1	1	0		
1	1	1	1	0	0	0	0	0	1	1	0		
0	0	0	0	0	0	0	0	0	0	0	0		

Maluku	New Guinea		Sulawesi		Sumatra	E-Java	Bali	Lomboc	Sumbawa	Flores	Philippines	Borneo	W-Java
1	1	1	1	0	0	0	0	0	1	1	0		
1	1	1	1	0	0	0	0	0	1	1	0		
1	1	1	1	0	0	0	0	0	1	1	0		
1	1	1	1	0	0	0	0	0	1	1	0		
0	0	0	0	0	0	0	0	0	0	0	0		
0	0	0	0	0	0	0	0	0	0	0	0		
0	0	0	0	0	0	0	0	0	0	0	0		
0	0	0	0	0	0	0	0	0	0	0	0		
0	0	0	0	0	0	0	0	0	0	0	0		
1	1	1	1	0	0	0	0	0	1	1	0		
1	1	1	1	0	0	0	0	0	1	1	0		
0	0	0	0	0	0	0	0	0	0	0	0		

Maluku	New Guinea		Sulawesi		Sumatra	E-Java	Bali	Lomboc	Sumbawa	Flores	Philippines	Borneo	W-Java
0	0	0	0	0	0	0	0	0	0	0	0		
0	1	1	1	0	0	0	0	0	1	1	0		
0	1	1	1	0	0	0	0	0	1	1	0		
0	1	1	1	0	0	0	0	0	1	1	0		
0	0	0	0	0	0	0	0	0	0	0	0		
0	0	0	0	0	0	0	0	0	0	0	0		
0	0	0	0	0	0	0	0	0	0	0	0		
0	0	0	0	0	0	0	0	0	0	0	0		
0	0	0	0	0	0	0	0	0	0	0	0		
0	1	1	1	0	0	0	0	0	1	1	0		
0	1	1	1	0	0	0	0	0	1	1	0		
0	0	0	0	0	0	0	0	0	0	0	0		

END

Manual dispersal multipliers, dispersal unconstrained

Maluku	New Guinea		Sulawesi		Sumatra	E-Java	Bali	Lomboc	Sumbawa	Flores	Philippines		Borneo	W-Java
1	1	1	1	1	1	1	1	1	1	1	1			
1	1	1	1	1	1	1	1	1	1	1	1			
1	1	1	1	1	1	1	1	1	1	1	1			
1	1	1	1	1	1	1	1	1	1	1	1			
1	1	1	1	1	1	1	1	1	1	1	1			
1	1	1	1	1	1	1	1	1	1	1	1			
1	1	1	1	1	1	1	1	1	1	1	1			
1	1	1	1	1	1	1	1	1	1	1	1			
1	1	1	1	1	1	1	1	1	1	1	1			
1	1	1	1	1	1	1	1	1	1	1	1			
1	1	1	1	1	1	1	1	1	1	1	1			
1	1	1	1	1	1	1	1	1	1	1	1			

Maluku	New Guinea		Sulawesi		Sumatra	E-Java	Bali	Lomboc	Sumbawa	Flores	Philippines		Borneo	W-Java
1	1	1	1	1	1	1	1	1	1	1	1			
1	1	1	1	1	1	1	1	1	1	1	1			
1	1	1	1	1	1	1	1	1	1	1	1			
1	1	1	1	1	1	1	1	1	1	1	1			
1	1	1	1	1	1	1	1	1	1	1	1			
1	1	1	1	1	1	1	1	1	1	1	1			
1	1	1	1	1	1	1	1	1	1	1	1			
1	1	1	1	1	1	1	1	1	1	1	1			
1	1	1	1	1	1	1	1	1	1	1	1			
1	1	1	1	1	1	1	1	1	1	1	1			
1	1	1	1	1	1	1	1	1	1	1	1			
1	1	1	1	1	1	1	1	1	1	1	1			

Maluku	New Guinea		Sulawesi		Sumatra	E-Java	Bali	Lomboc	Sumbawa	Flores	Philippines		Borneo	W-Java
1	1	1	1	1	0	0	1	1	1	1	1			
1	1	1	1	1	0	0	1	1	1	1	1			
1	1	1	1	1	0	0	1	1	1	1	1			
1	1	1	1	1	0	0	1	1	1	1	1			
1	1	1	1	1	0	0	1	1	1	1	1			
0	0	0	0	0	0	0	0	0	0	0	0			
0	0	0	0	0	0	0	0	0	0	0	0			
1	1	1	1	1	0	0	1	1	1	1	1			
1	1	1	1	1	0	0	1	1	1	1	1			
1	1	1	1	1	0	0	1	1	1	1	1			
1	1	1	1	1	0	0	1	1	1	1	1			
1	1	1	1	1	0	0	1	1	1	1	1			

Maluku	New Guinea		Sulawesi		Sumatra	E-Java	Bali	Lomboc	Sumbawa	Flores	Philippines		Borneo	W-Java
1	1	1	1	0	0	0	0	0	1	1	0			
1	1	1	1	0	0	0	0	0	1	1	0			
1	1	1	1	0	0	0	0	0	1	1	0			
1	1	1	1	0	0	0	0	0	1	1	0			
0	0	0	0	0	0	0	0	0	0	0	0			
0	0	0	0	0	0	0	0	0	0	0	0			
0	0	0	0	0	0	0	0	0	0	0	0			
0	0	0	0	0	0	0	0	0	0	0	0			
0	0	0	0	0	0	0	0	0	0	0	0			
1	1	1	1	0	0	0	0	0	1	1	0			
1	1	1	1	0	0	0	0	0	1	1	0			
0	0	0	0	0	0	0	0	0	0	0	0			

Maluku	New Guinea		Sulawesi		Sumatra	E-Java	Bali	Lomboc	Sumbawa	Flores	Philippines		Borneo	W-Java
1	1	1	1	0	0	0	0	0	1	1	0			
1	1	1	1	0	0	0	0	0	1	1	0			
1	1	1	1	0	0	0	0	0	1	1	0			
1	1	1	1	0	0	0	0	0	1	1	0			
0	0	0	0	0	0	0	0	0	0	0	0			
0	0	0	0	0	0	0	0	0	0	0	0			
0	0	0	0	0	0	0	0	0	0	0	0			
0	0	0	0	0	0	0	0	0	0	0	0			
0	0	0	0	0	0	0	0	0	0	0	0			
1	1	1	1	0	0	0	0	0	1	1	0			
1	1	1	1	0	0	0	0	0	1	1	0			
0	0	0	0	0	0	0	0	0	0	0	0			

Maluku	New Guinea		Sulawesi		Sumatra	E-Java	Bali	Lomboc	Sumbawa	Flores	Philippines		Borneo	W-Java
0	0	0	0	0	0	0	0	0	0	0	0			
0	1	1	1	0	0	0	0	0	1	1	0			
0	1	1	1	0	0	0	0	0	1	1	0			
0	1	1	1	0	0	0	0	0	1	1	0			
0	0	0	0	0	0	0	0	0	0	0	0			
0	0	0	0	0	0	0	0	0	0	0	0			
0	0	0	0	0	0	0	0	0	0	0	0			
0	0	0	0	0	0	0	0	0	0	0	0			
0	0	0	0	0	0	0	0	0	0	0	0			
0	1	1	1	0	0	0	0	0	1	1	0			
0	1	1	1	0	0	0	0	0	1	1	0			
0	0	0	0	0	0	0	0	0	0	0	0			

END

Manual dispersal multipliers, dispersal constrained to adjacent areas

Maluku	New Guinea		Sulawesi		Sumatra	E-Java	Bali	Lomboc	Sumbawa	Flores	Philippines	Borneo	W-Java
1	1	1	0	0	0	0	0	1	1	1	0		
1	1	1	0	0	0	0	1	1	1	1	0		
1	1	1	0	1	1	1	1	1	1	1	1		
0	0	0	1	0	0	0	0	0	0	1	1		
0	0	1	0	1	1	0	0	0	0	1	1		
0	0	1	0	1	1	1	0	0	0	1	0		
0	0	1	0	0	1	1	1	0	0	1	0		
0	1	1	0	0	0	1	1	1	0	0	0		
1	1	1	0	0	0	0	1	1	0	1	0		
1	1	1	0	0	0	0	0	0	1	1	0		
1	1	1	1	1	1	1	0	1	1	1	1		
0	0	1	1	1	0	0	0	0	0	1	1		

Maluku	New Guinea		Sulawesi		Sumatra	E-Java	Bali	Lomboc	Sumbawa	Flores	Philippines	Borneo	W-Java
1	1	1	0	0	0	0	0	1	1	1	0		
1	1	1	0	0	0	0	1	1	1	1	0		
1	1	1	0	1	1	1	1	1	1	1	1		
0	0	0	1	0	0	0	0	0	0	1	1		
0	0	1	0	1	1	0	0	0	0	1	1		
0	0	1	0	1	1	1	0	0	0	1	0		
0	0	1	0	0	1	1	1	0	0	1	0		
0	1	1	0	0	0	1	1	1	0	0	0		
1	1	1	0	0	0	0	1	1	0	1	0		
1	1	1	0	0	0	0	0	0	1	1	0		
1	1	1	1	1	1	1	0	1	1	1	1		
0	0	1	1	1	0	0	0	0	0	1	1		

Maluku	New Guinea		Sulawesi		Sumatra	E-Java	Bali	Lomboc	Sumbawa	Flores	Philippines	Borneo	W-Java
1	1	1	0	0	0	0	0	1	1	1	0		
1	1	1	0	0	0	0	1	1	1	1	0		
1	1	1	0	1	0	0	1	1	1	1	1		
0	0	0	1	0	0	0	0	0	0	1	1		
0	0	1	0	1	0	0	0	0	0	1	1		
0	0	0	0	0	0	0	0	0	0	0	0		
0	0	0	0	0	0	0	0	0	0	0	0		
0	1	1	0	0	0	0	1	1	0	0	0		
1	1	1	0	0	0	0	1	1	0	1	0		
1	1	1	0	0	0	0	0	0	1	1	0		
1	1	1	1	1	0	0	0	1	1	1	1		
0	0	1	1	1	0	0	0	0	0	1	1		

Maluku	New Guinea		Sulawesi		Sumatra	E-Java	Bali	Lomboc	Sumbawa	Flores	Philippines	Borneo	W-Java
1	1	1	0	0	0	0	0	0	1	1	0		
1	1	1	0	0	0	0	0	0	1	1	0		
1	1	1	0	0	0	0	0	0	1	1	0		
0	0	0	1	0	0	0	0	0	0	1	0		
0	0	0	0	0	0	0	0	0	0	0	0		
0	0	0	0	0	0	0	0	0	0	0	0		
0	0	0	0	0	0	0	0	0	0	0	0		
0	0	0	0	0	0	0	0	0	0	0	0		
0	0	0	0	0	0	0	0	0	0	0	0		
1	1	1	0	0	0	0	0	0	1	1	0		
1	1	1	1	0	0	0	0	0	1	1	0		
0	0	0	0	0	0	0	0	0	0	0	0		

Maluku	New Guinea		Sulawesi		Sumatra	E-Java	Bali	Lomboc	Sumbawa	Flores	Philippines	Borneo	W-Java
1	1	1	0	0	0	0	0	0	1	1	0		
1	1	1	0	0	0	0	0	0	1	1	0		
1	1	1	0	0	0	0	0	0	1	1	0		
0	0	0	1	0	0	0	0	0	0	1	0		
0	0	0	0	0	0	0	0	0	0	0	0		
0	0	0	0	0	0	0	0	0	0	0	0		
0	0	0	0	0	0	0	0	0	0	0	0		
0	0	0	0	0	0	0	0	0	0	0	0		
0	0	0	0	0	0	0	0	0	0	0	0		
1	1	1	0	0	0	0	0	0	1	1	0		
1	1	1	1	0	0	0	0	0	1	1	0		
0	0	0	0	0	0	0	0	0	0	0	0		

Maluku	New Guinea		Sulawesi		Sumatra	E-Java	Bali	Lomboc	Sumbawa	Flores	Philippines	Borneo	W-Java
0	0	0	0	0	0	0	0	0	0	0	0		
0	1	1	0	0	0	0	0	0	1	1	0		
0	1	1	0	0	0	0	0	0	1	1	0		
0	0	0	1	0	0	0	0	0	0	1	0		
0	0	0	0	0	0	0	0	0	0	0	0		
0	0	0	0	0	0	0	0	0	0	0	0		
0	0	0	0	0	0	0	0	0	0	0	0		
0	0	0	0	0	0	0	0	0	0	0	0		
0	0	0	0	0	0	0	0	0	0	0	0		
0	1	1	0	0	0	0	0	0	1	1	0		
0	1	1	1	0	0	0	0	0	1	1	0		
0	0	0	0	0	0	0	0	0	0	0	0		

END

Manual dispersal multipliers, dispersal constrained to adjacent areas except for the Lower Sunda island chain

Maluku	New Guinea	Sulawesi	Sumatra	E-Java	Bali	Lomboc	Sumbawa	Flores	Philippines	Borneo	W-Java
1	1	1	0	0	0	0	0	1	1	1	0
1	1	1	0	0	0	0	1	1	1	1	0
1	1	1	0	1	1	1	1	1	1	1	1
0	0	0	1	0	0	0	0	0	0	1	1
0	0	1	0	1	1	1	1	1	0	1	1
0	0	1	0	1	1	1	1	1	0	1	1
0	0	1	0	1	1	1	1	1	0	1	1
0	1	1	0	1	1	1	1	1	0	0	1
1	1	1	0	1	1	1	1	1	0	1	1
1	1	1	0	0	0	0	0	0	1	1	0
1	1	1	1	1	1	1	0	1	1	1	1
0	0	1	1	1	1	1	1	1	0	1	1

Maluku	New Guinea	Sulawesi	Sumatra	E-Java	Bali	Lomboc	Sumbawa	Flores	Philippines	Borneo	W-Java
1	1	1	0	0	0	0	0	1	1	1	0
1	1	1	0	0	0	0	1	1	1	1	0
1	1	1	0	1	1	1	1	1	1	1	1
0	0	0	1	0	0	0	0	0	0	1	1
0	0	1	0	1	1	1	1	1	0	1	1
0	0	1	0	1	1	1	1	1	0	1	1
0	0	1	0	1	1	1	1	1	0	1	1
0	1	1	0	1	1	1	1	1	0	0	1
1	1	1	0	1	1	1	1	1	0	1	1
1	1	1	0	0	0	0	0	0	1	1	0
1	1	1	1	1	1	1	0	1	1	1	1
0	0	1	1	1	1	1	1	1	0	1	1

Maluku	New Guinea	Sulawesi	Sumatra	E-Java	Bali	Lomboc	Sumbawa	Flores	Philippines	Borneo	W-Java
1	1	1	1	0	0	0	0	1	1	1	0
1	1	1	0	0	0	0	1	1	1	1	0
1	1	1	1	1	0	0	0	1	1	1	1
0	0	0	0	0	0	0	1	0	0	1	1
0	0	1	0	1	0	0	1	1	0	1	1
0	0	0	0	0	0	0	0	0	0	0	0
0	0	0	0	0	0	0	0	0	0	0	0
0	1	1	0	1	0	0	1	1	0	0	1
1	1	1	0	1	0	0	1	1	0	1	1
1	1	1	0	0	0	0	0	0	1	1	0
1	1	1	1	1	0	0	0	1	1	1	1
0	0	1	1	1	0	0	1	1	0	1	1

Maluku	New Guinea	Sulawesi	Sumatra	E-Java	Bali	Lomboc	Sumbawa	Flores	Philippines	Borneo	W-Java
1	1	1	1	0	0	0	0	0	1	1	0
1	1	1	0	0	0	0	0	0	1	1	0
1	1	1	1	0	0	0	0	0	1	1	0
0	0	0	0	0	0	0	0	0	0	1	0
0	0	0	0	0	0	0	0	0	0	0	0
0	0	0	0	0	0	0	0	0	0	0	0
0	0	0	0	0	0	0	0	0	0	0	0
0	0	0	0	0	0	0	0	0	0	0	0
0	0	0	0	0	0	0	0	0	0	0	0
1	1	1	0	0	0	0	0	0	1	1	0
1	1	1	1	0	0	0	0	0	1	1	0
0	0	0	0	0	0	0	0	0	0	0	0

Maluku	New Guinea	Sulawesi	Sumatra	E-Java	Bali	Lomboc	Sumbawa	Flores	Philippines	Borneo	W-Java
1	1	1	1	0	0	0	0	0	1	1	0
1	1	1	0	0	0	0	0	0	1	1	0
1	1	1	1	0	0	0	0	0	1	1	0
0	0	0	0	0	0	0	0	0	0	1	0
0	0	0	0	0	0	0	0	0	0	0	0
0	0	0	0	0	0	0	0	0	0	0	0
0	0	0	0	0	0	0	0	0	0	0	0
0	0	0	0	0	0	0	0	0	0	0	0
0	0	0	0	0	0	0	0	0	0	0	0
1	1	1	0	0	0	0	0	0	1	1	0
1	1	1	1	0	0	0	0	0	1	1	0
0	0	0	0	0	0	0	0	0	0	0	0

Maluku	New Guinea	Sulawesi	Sumatra	E-Java	Bali	Lomboc	Sumbawa	Flores	Philippines	Borneo	W-Java
0	0	0	0	0	0	0	0	0	0	0	0
0	1	1	0	0	0	0	0	0	1	1	0
0	1	1	1	0	0	0	0	0	1	1	0
0	0	0	0	0	0	0	0	0	0	1	0
0	0	0	0	0	0	0	0	0	0	0	0
0	0	0	0	0	0	0	0	0	0	0	0
0	0	0	0	0	0	0	0	0	0	0	0
0	0	0	0	0	0	0	0	0	0	0	0
0	0	0	0	0	0	0	0	0	0	0	0
0	1	1	0	0	0	0	0	0	1	1	0
0	1	1	1	0	0	0	0	0	1	1	0
0	0	0	0	0	0	0	0	0	0	0	0

END

Distances matrix, dispersal unconstrained

Maluku	New Guinea		Sulawesi		Sumatra	E-Java	Bali	Lomboc	Sumbawa	Flores	Philippines	Borneo	W-Java
0	215.6	289.4	2432	1667	1553	1461	1244	990	557.7	1090	2030		
215.6	0	819.4	2825	1991	1888	1777	1562	1180	962.9	1487	2380		
289.4	819.4	0	1552	648.6	557	461.9	321.3	314.1	458.6	148.6	1052		
2432	2825	1552	0	561.6	1097	1232	1383	1645	1816	547.1	66.36		
1667	1991	648.6	561.6	0	27.5	202.1	285	608.7	1959	425.2	29.61		
1553	1888	557	1097	27.5	0	58.34	147.6	461.4	1853	475.6	461.9		
1461	1777	461.9	1232	202.1	58.34	0	22.76	343.3	1875	510.7	630.3		
1244	1562	321.3	1383	285	147.6	22.76	0	67.5	1889	571.7	713.2		
990	1180	314.1	1645	608.7	461.4	343.3	67.5	0	1928	689.9	1039		
557.7	962.9	458.6	1816	1959	1853	1875	1889	1928	0	180.7	2180		
1090	1487	148.6	547.1	425.2	475.6	510.7	571.7	689.9	180.7	0	445.8		
2030	2380	1052	66.36	29.61	461.9	630.3	713.2	1039	2180	445.8	0		

Maluku	New Guinea		Sulawesi		Sumatra	E-Java	Bali	Lomboc	Sumbawa	Flores	Philippines	Borneo	W-Java
0	215.6	289.4	2432	1667	1553	1461	1244	990	557.7	1090	2030		
215.6	0	819.4	2825	1991	1888	1777	1562	1180	962.9	1487	2380		
289.4	819.4	0	1552	648.6	557	461.9	321.3	314.1	458.6	148.6	1052		
2432	2825	1552	0	561.6	1097	1232	1383	1645	1816	547.1	66.36		
1667	1991	648.6	561.6	0	27.5	202.1	285	608.7	1959	425.2	29.61		
1553	1888	557	1097	27.5	0	58.34	147.6	461.4	1853	475.6	461.9		
1461	1777	461.9	1232	202.1	58.34	0	22.76	343.3	1875	510.7	630.3		
1244	1562	321.3	1383	285	147.6	22.76	0	67.5	1889	571.7	713.2		
990	1180	314.1	1645	608.7	461.4	343.3	67.5	0	1928	689.9	1039		
557.7	962.9	458.6	1816	1959	1853	1875	1889	1928	0	180.7	2180		
1090	1487	148.6	547.1	425.2	475.6	510.7	571.7	689.9	180.7	0	445.8		
2030	2380	1052	66.36	29.61	461.9	630.3	713.2	1039	2180	445.8	0		

Maluku	New Guinea		Sulawesi		Sumatra	E-Java	Bali	Lomboc	Sumbawa	Flores	Philippines	Borneo	W-Java
0	215.6	289.4	2432	1667	9999	9999	1244	990	557.7	1090	2030		
215.6	0	819.4	2825	1991	9999	9999	1562	1180	962.9	1487	2380		
289.4	819.4	0	1552	648.6	9999	9999	321.3	314.1	458.6	148.6	1052		
2432	2825	1552	0	561.6	9999	9999	1383	1645	1816	547.1	66.36		
1667	1991	648.6	561.6	0	9999	9999	285	608.7	1959	425.2	29.61		
9999	9999	9999	9999	9999	0	9999	9999	9999	9999	9999	9999		
9999	9999	9999	9999	9999	9999	0	9999	9999	9999	9999	9999		
1244	1562	321.3	1383	285	9999	9999	0	67.5	1889	571.7	713.2		
990	1180	314.1	1645	608.7	9999	9999	67.5	0	1928	689.9	1039		
557.7	962.9	458.6	1816	1959	9999	9999	1889	1928	0	180.7	2180		
1090	1487	148.6	547.1	425.2	9999	9999	571.7	689.9	180.7	0	445.8		
2030	2380	1052	66.36	29.61	9999	9999	713.2	1039	2180	445.8	0		

Maluku	New Guinea		Sulawesi		Sumatra	E-Java	Bali	Lomboc	Sumbawa	Flores	Philippines	Borneo	W-Java
0	215.6	289.4	2432	9999	9999	9999	9999	9999	557.7	1090	9999		
215.6	0	819.4	2825	9999	9999	9999	9999	9999	962.9	1487	9999		
289.4	819.4	0	1552	9999	9999	9999	9999	9999	458.6	148.6	9999		
2432	2825	1552	0	9999	9999	9999	9999	9999	1816	547.1	9999		
9999	9999	9999	9999	0	9999	9999	9999	9999	9999	9999	9999		
9999	9999	9999	9999	9999	0	9999	9999	9999	9999	9999	9999		
9999	9999	9999	9999	9999	9999	0	9999	9999	9999	9999	9999		
9999	9999	9999	9999	9999	9999	9999	0	9999	9999	9999	9999		
9999	9999	9999	9999	9999	9999	9999	9999	0	9999	9999	9999		
557.7	962.9	458.6	1816	9999	9999	9999	9999	9999	0	180.7	9999		
1090	1487	148.6	547.1	9999	9999	9999	9999	9999	180.7	0	9999		
9999	9999	9999	9999	9999	9999	9999	9999	9999	9999	9999	0		

Maluku	New Guinea		Sulawesi		Sumatra	E-Java	Bali	Lomboc	Sumbawa	Flores	Philippines	Borneo	W-Java
0	215.6	289.4	2432	9999	9999	9999	9999	9999	557.7	1090	9999		
215.6	0	819.4	2825	9999	9999	9999	9999	9999	962.9	1487	9999		
289.4	819.4	0	1552	9999	9999	9999	9999	9999	458.6	148.6	9999		
2432	2825	1552	0	9999	9999	9999	9999	9999	1816	547.1	9999		
9999	9999	9999	9999	0	9999	9999	9999	9999	9999	9999	9999		
9999	9999	9999	9999	9999	0	9999	9999	9999	9999	9999	9999		
9999	9999	9999	9999	9999	9999	0	9999	9999	9999	9999	9999		
9999	9999	9999	9999	9999	9999	9999	0	9999	9999	9999	9999		
9999	9999	9999	9999	9999	9999	9999	9999	0	9999	9999	9999		
557.7	962.9	458.6	1816	9999	9999	9999	9999	9999	0	180.7	9999		
1090	1487	148.6	547.1	9999	9999	9999	9999	9999	180.7	0	9999		
9999	9999	9999	9999	9999	9999	9999	9999	9999	9999	9999	0		

Maluku	New Guinea		Sulawesi		Sumatra	E-Java	Bali	Lomboc	Sumbawa	Flores	Philippines	Borneo	W-Java
0	9999	9999	9999	9999	9999	9999	9999	9999	9999	9999	9999		
9999	0	819.4	282	9999	9999	9999	9999	9999	962.9	1487	9999		
9999	819.4	0	155	9999	9999	9999	9999	9999	458.6	148.6	9999		
9999	2825	1552	0	9999	9999	9999	9999	9999	1816	547.1	9999		
9999	9999	9999	9999	0	9999	9999	9999	9999	9999	9999	9999		
9999	9999	9999	9999	9999	0	9999	9999	9999	9999	9999	9999		
9999	9999	9999	9999	9999	9999	0	9999	9999	9999	9999	9999		
9999	9999	9999	9999	9999	9999	9999	0	9999	9999	9999	9999		
9999	9999	9999	9999	9999	9999	9999	9999	0	9999	9999	9999		
9999	962.9	458.6	1816	9999	9999	9999	9999	9999	0	180.7	9999		
9999	1487	148.6	547.1	9999	9999	9999	9999	9999	180.7	0	9999		
9999	9999	9999	9999	9999	9999	9999	9999	9999	9999	9999	0		

END

Distances matrix, dispersal constrained to adjacent areas

Maluku	New Guinea		Sulawesi		Sumatra	E-Java	Bali	Lomboc	Sumbawa	Flores	Philippines		Borneo	W-Java
0	215.6	289.4	9999	9999	9999	9999	9999	990	557.7	1090	9999			
215.6	0	819.4	9999	9999	9999	9999	1562	1180	962.9	1487	9999			
289.4	819.4	0	9999	648.6	557	461.9	321.3	314.1	458.6	148.6	1052			
9999	9999	9999	0	9999	9999	9999	9999	9999	9999	547.1	66.36			
9999	9999	648.6	9999	0	27.5	9999	9999	9999	9999	425.2	29.61			
9999	9999	557	9999	27.5	0	58.34	9999	9999	9999	475.6	9999			
9999	9999	461.9	9999	9999	58.34	0	22.76	9999	9999	510.7	9999			
9999	1562	321.3	9999	9999	9999	22.76	0	67.5	9999	9999	9999			
990	1180	314.1	9999	9999	9999	9999	67.5	0	9999	689.9	9999			
557.7	962.9	458.6	9999	9999	9999	9999	9999	9999	0	180.7	9999			
1090	1487	148.6	547.1	425.2	475.6	510.7	9999	689.9	180.7	0	445.8			
9999	9999	1052	66.36	29.61	9999	9999	9999	9999	9999	445.8	0			

Maluku	New Guinea		Sulawesi		Sumatra	E-Java	Bali	Lomboc	Sumbawa	Flores	Philippines		Borneo	W-Java
0	215.6	289.4	9999	9999	9999	9999	9999	990	557.7	1090	9999			
215.6	0	819.4	9999	9999	9999	9999	1562	1180	962.9	1487	9999			
289.4	819.4	0	9999	648.6	557	461.9	321.3	314.1	458.6	148.6	1052			
9999	9999	9999	0	9999	9999	9999	9999	9999	9999	547.1	66.36			
9999	9999	648.6	9999	0	27.5	9999	9999	9999	9999	425.2	29.61			
9999	9999	557	9999	27.5	0	58.34	9999	9999	9999	475.6	9999			
9999	9999	461.9	9999	9999	58.34	0	22.76	9999	9999	510.7	9999			
9999	1562	321.3	9999	9999	9999	22.76	0	67.5	9999	9999	9999			
990	1180	314.1	9999	9999	9999	9999	67.5	0	9999	689.9	9999			
557.7	962.9	458.6	9999	9999	9999	9999	9999	9999	0	180.7	9999			
1090	1487	148.6	547.1	425.2	475.6	510.7	9999	689.9	180.7	0	445.8			
9999	9999	1052	66.36	29.61	9999	9999	9999	9999	9999	445.8	0			

Maluku	New Guinea		Sulawesi		Sumatra	E-Java	Bali	Lomboc	Sumbawa	Flores	Philippines		Borneo	W-Java
0	215.6	289.4	9999	9999	9999	9999	9999	990	557.7	1090	9999			
215.6	0	819.4	9999	9999	9999	9999	1562	1180	962.9	1487	9999			
289.4	819.4	0	9999	648.6	9999	9999	321.3	314.1	458.6	148.6	1052			
9999	9999	9999	0	9999	9999	9999	9999	9999	9999	547.1	66.36			
9999	9999	648.6	9999	0	9999	9999	9999	9999	9999	425.2	29.61			
9999	9999	9999	9999	9999	0	9999	9999	9999	9999	9999	9999			
9999	9999	9999	9999	9999	9999	0	9999	9999	9999	9999	9999			
9999	1562	321.3	9999	9999	9999	9999	0	67.5	9999	9999	9999			
990	1180	314.1	9999	9999	9999	9999	67.5	0	9999	689.9	9999			
557.7	962.9	458.6	9999	9999	9999	9999	9999	9999	0	180.7	9999			
1090	1487	148.6	547.1	425.2	9999	9999	9999	689.9	180.7	0	445.8			
9999	9999	1052	66.36	29.61	9999	9999	9999	9999	9999	445.8	0			

Maluku	New Guinea		Sulawesi		Sumatra	E-Java	Bali	Lomboc	Sumbawa	Flores	Philippines		Borneo	W-Java
0	215.6	289.4	9999	9999	9999	9999	9999	9999	557.7	1090	9999			
215.6	0	819.4	9999	9999	9999	9999	9999	9999	962.9	1487	9999			
289.4	819.4	0	9999	9999	9999	9999	9999	9999	458.6	148.6	9999			
9999	9999	9999	0	9999	9999	9999	9999	9999	9999	547.1	9999			
9999	9999	9999	9999	0	9999	9999	9999	9999	9999	9999	9999			
9999	9999	9999	9999	9999	0	9999	9999	9999	9999	9999	9999			
9999	9999	9999	9999	9999	9999	0	9999	9999	9999	9999	9999			
9999	9999	9999	9999	9999	9999	9999	0	9999	9999	9999	9999			
9999	9999	9999	9999	9999	9999	9999	9999	0	9999	9999	9999			
557.7	962.9	458.6	9999	9999	9999	9999	9999	9999	0	180.7	9999			
1090	1487	148.6	547.1	9999	9999	9999	9999	9999	180.7	0	9999			
9999	9999	9999	9999	9999	9999	9999	9999	9999	9999	9999	0			

Maluku	New Guinea		Sulawesi		Sumatra	E-Java	Bali	Lomboc	Sumbawa	Flores	Philippines		Borneo	W-Java
0	215.6	289.4	9999	9999	9999	9999	9999	9999	557.7	1090	9999			
215.6	0	819.4	9999	9999	9999	9999	9999	9999	962.9	1487	9999			
289.4	819.4	0	9999	9999	9999	9999	9999	9999	458.6	148.6	9999			
9999	9999	9999	0	9999	9999	9999	9999	9999	9999	547.1	9999			
9999	9999	9999	9999	0	9999	9999	9999	9999	9999	9999	9999			
9999	9999	9999	9999	9999	0	9999	9999	9999	9999	9999	9999			
9999	9999	9999	9999	9999	9999	0	9999	9999	9999	9999	9999			
9999	9999	9999	9999	9999	9999	9999	0	9999	9999	9999	9999			
9999	9999	9999	9999	9999	9999	9999	9999	0	9999	9999	9999			
557.7	962.9	458.6	9999	9999	9999	9999	9999	9999	0	180.7	9999			
1090	1487	148.6	547.1	9999	9999	9999	9999	9999	180.7	0	9999			
9999	9999	9999	9999	9999	9999	9999	9999	9999	9999	9999	0			

Maluku	New Guinea		Sulawesi		Sumatra	E-Java	Bali	Lomboc	Sumbawa	Flores	Philippines		Borneo	W-Java
0	9999	9999	9999	9999	9999	9999	9999	9999	9999	9999	9999			
9999	0	819.4	9999	9999	9999	9999	9999	9999	962.9	1487	9999			
9999	819.4	0	9999	9999	9999	9999	9999	9999	458.6	148.6	9999			
9999	9999	9999	0	9999	9999	9999	9999	9999	9999	547.1	9999			
9999	9999	9999	9999	0	9999	9999	9999	9999	9999	9999	9999			
9999	9999	9999	9999	9999	0	9999	9999	9999	9999	9999	9999			
9999	9999	9999	9999	9999	9999	0	9999	9999	9999	9999	9999			
9999	9999	9999	9999	9999	9999	9999	0	9999	9999	9999	9999			
9999	9999	9999	9999	9999	9999	9999	9999	0	9999	9999	9999			
9999	962.9	458.6	9999	9999	9999	9999	9999	9999	0	180.7	9999			
9999	1487	148.6	547.1	9999	9999	9999	9999	9999	180.7	0	9999			
9999	9999	9999	9999	9999	9999	9999	9999	9999	9999	9999	0			

END

Distances matrix, dispersal constrained to adjacent areas except for the Lower Sunda island chain

Maluku	New Guinea	Sulawesi	Sumatra	E-Java	Bali	Lomboc	Sumbawa	Flores	Philippines	Borneo	W-Java
0	215.6	289.4	9999	9999	9999	9999	9999	990	557.7	1090	9999
215.6	0	819.4	9999	9999	9999	9999	1562	1180	962.9	1487	9999
289.4	819.4	0	9999	648.6	557	461.9	321.3	314.1	458.6	148.6	1052
9999	9999	9999	0	9999	9999	9999	9999	9999	9999	547.1	66.36
9999	9999	648.6	9999	0	27.5	202.1	285	608.7	9999	425.2	29.61
9999	9999	557	9999	27.5	0	58.34	147.6	461.4	9999	475.6	461.9
9999	9999	461.9	9999	202.1	58.34	0	22.76	343.3	9999	510.7	630.3
9999	1562	321.3	9999	285	147.6	22.76	0	67.5	9999	9999	713.2
990	1180	314.1	9999	608.7	461.4	343.3	67.5	0	9999	689.9	1039
557.7	962.9	458.6	9999	9999	9999	9999	9999	9999	0	180.7	9999
1090	1487	148.6	547.1	425.2	475.6	510.7	9999	689.9	180.7	0	445.8
9999	9999	1052	66.36	29.61	461.9	630.3	713.2	1039	9999	445.8	0

Maluku	New Guinea	Sulawesi	Sumatra	E-Java	Bali	Lomboc	Sumbawa	Flores	Philippines	Borneo	W-Java
0	215.6	289.4	9999	9999	9999	9999	9999	990	557.7	1090	9999
215.6	0	819.4	9999	9999	9999	9999	1562	1180	962.9	1487	9999
289.4	819.4	0	9999	648.6	557	461.9	321.3	314.1	458.6	148.6	1052
9999	9999	9999	0	9999	9999	9999	9999	9999	9999	547.1	66.36
9999	9999	648.6	9999	0	27.5	202.1	285	608.7	9999	425.2	29.61
9999	9999	557	9999	27.5	0	58.34	147.6	461.4	9999	475.6	461.9
9999	9999	461.9	9999	202.1	58.34	0	22.76	343.3	9999	510.7	630.3
9999	1562	321.3	9999	285	147.6	22.76	0	67.5	9999	9999	713.2
990	1180	314.1	9999	608.7	461.4	343.3	67.5	0	9999	689.9	1039
557.7	962.9	458.6	9999	9999	9999	9999	9999	9999	0	180.7	9999
1090	1487	148.6	547.1	425.2	475.6	510.7	9999	689.9	180.7	0	445.8
9999	9999	1052	66.36	29.61	461.9	630.3	713.2	1039	9999	445.8	0

Maluku	New Guinea	Sulawesi	Sumatra	E-Java	Bali	Lomboc	Sumbawa	Flores	Philippines	Borneo	W-Java
0	215.6	289.4	9999	9999	9999	9999	9999	990	557.7	1090	9999
215.6	0	819.4	9999	9999	9999	9999	1562	1180	962.9	1487	9999
289.4	819.4	0	9999	648.6	9999	9999	321.3	314.1	458.6	148.6	1052
9999	9999	9999	0	9999	9999	9999	9999	9999	9999	547.1	66.36
9999	9999	648.6	9999	0	9999	9999	285	608.7	9999	425.2	29.61
9999	9999	9999	9999	9999	0	9999	9999	9999	9999	9999	9999
9999	9999	9999	9999	9999	9999	0	9999	9999	9999	9999	9999
9999	1562	321.3	9999	285	9999	9999	0	67.5	9999	9999	713.2
990	1180	314.1	9999	608.7	9999	9999	67.5	0	9999	689.9	1039
557.7	962.9	458.6	9999	9999	9999	9999	9999	9999	0	180.7	9999
1090	1487	148.6	547.1	425.2	9999	9999	9999	689.9	180.7	0	445.8
9999	9999	1052	66.36	29.61	9999	9999	713.2	1039	9999	445.8	0

Maluku	New Guinea	Sulawesi	Sumatra	E-Java	Bali	Lomboc	Sumbawa	Flores	Philippines	Borneo	W-Java
0	215.6	289.4	9999	9999	9999	9999	9999	9999	557.7	1090	9999
215.6	0	819.4	9999	9999	9999	9999	9999	9999	962.9	1487	9999
289.4	819.4	0	9999	648.6	9999	9999	9999	9999	458.6	148.6	9999
9999	9999	9999	0	9999	9999	9999	9999	9999	9999	547.1	9999
9999	9999	9999	9999	0	9999	9999	9999	9999	9999	9999	9999
9999	9999	9999	9999	9999	0	9999	9999	9999	9999	9999	9999
9999	9999	9999	9999	9999	9999	0	9999	9999	9999	9999	9999
9999	9999	9999	9999	9999	9999	9999	0	9999	9999	9999	9999
9999	9999	9999	9999	9999	9999	9999	9999	0	9999	9999	9999
557.7	962.9	458.6	9999	9999	9999	9999	9999	9999	0	180.7	9999
1090	1487	148.6	547.1	425.2	9999	9999	9999	9999	180.7	0	9999
9999	9999	9999	9999	9999	9999	9999	9999	9999	9999	9999	0

Maluku	New Guinea	Sulawesi	Sumatra	E-Java	Bali	Lomboc	Sumbawa	Flores	Philippines	Borneo	W-Java
0	215.6	289.4	9999	9999	9999	9999	9999	9999	557.7	1090	9999
215.6	0	819.4	9999	9999	9999	9999	9999	9999	962.9	1487	9999
289.4	819.4	0	9999	648.6	9999	9999	9999	9999	458.6	148.6	9999
9999	9999	9999	0	9999	9999	9999	9999	9999	9999	547.1	9999
9999	9999	9999	9999	0	9999	9999	9999	9999	9999	9999	9999
9999	9999	9999	9999	9999	0	9999	9999	9999	9999	9999	9999
9999	9999	9999	9999	9999	9999	0	9999	9999	9999	9999	9999
9999	9999	9999	9999	9999	9999	9999	0	9999	9999	9999	9999
9999	9999	9999	9999	9999	9999	9999	9999	0	9999	9999	9999
557.7	962.9	458.6	9999	9999	9999	9999	9999	9999	0	180.7	9999
1090	1487	148.6	547.1	425.2	9999	9999	9999	9999	180.7	0	9999
9999	9999	9999	9999	9999	9999	9999	9999	9999	9999	9999	0

Maluku	New Guinea	Sulawesi	Sumatra	E-Java	Bali	Lomboc	Sumbawa	Flores	Philippines	Borneo	W-Java
0	9999	9999	9999	9999	9999	9999	9999	9999	9999	9999	9999
215.6	0	819.4	9999	9999	9999	9999	9999	9999	962.9	1487	9999
289.4	819.4	0	9999	648.6	9999	9999	9999	9999	458.6	148.6	9999
9999	9999	9999	0	9999	9999	9999	9999	9999	9999	547.1	9999
9999	9999	9999	9999	0	9999	9999	9999	9999	9999	9999	9999
9999	9999	9999	9999	9999	0	9999	9999	9999	9999	9999	9999
9999	9999	9999	9999	9999	9999	0	9999	9999	9999	9999	9999
9999	9999	9999	9999	9999	9999	9999	0	9999	9999	9999	9999
9999	9999	9999	9999	9999	9999	9999	9999	0	9999	9999	9999
557.7	962.9	458.6	9999	9999	9999	9999	9999	9999	0	180.7	9999
1090	1487	148.6	547.1	425.2	9999	9999	9999	9999	180.7	0	9999
9999	9999	9999	9999	9999	9999	9999	9999	9999	9999	9999	0

END

Model non-time stratified 30Ma	Number of free parameters	LnL results	AICc values	AICc weights	Relative model probabilities, based on AICc
DEC	2	-352.532	709.128	3.03141E-57	0.00%
DEC+J	3	-252.553	511.234	2.84238E-14	0.00%
DEC constraints	2	-357.325	718.714	2.51159E-59	0.00%
DEC+J constraints	3	-291.329	588.787	4.1057E-31	0.00%
DEC constraints Lower Sunda open	2	-345.038	694.139	5.44893E-54	0.00%
DEC+J constraints Lower Sunda open	3	-255.861	517.850	1.04027E-15	0.00%
DEC+x allDist unconstrained	3	-350.617	707.362	7.33069E-57	0.00%
DEC+J+x allDist unconstrained	4	-230.640	469.493	3.29295E-05	0.00%
DEC+x constraints	3	-351.022	708.171	4.89144E-57	0.00%
DEC+J+x constraints	4	-226.409	461.032	0.002264586	0.23%
DEC+x constraints Lower Sunda open	3	-350.998	708.124	5.00599E-57	0.00%
DEC+J+x constraints Lower Sunda open	4	-220.319	448.851	1	99.77%

Model time stratified 30Ma	Number of free parameters	LnL results	AICc values	AICc weights	Relative model probabilities, based on AICc
DEC	2	-476.095	956.253	1.44399E-22	0.00%
DEC+J	3	-429.986	866.100	0.005445123	0.54%
DEC constraints	2	-472.233	948.529	6.86752E-21	0.00%
DEC+J constraints	3	-424.773	855.674	1	98.75%
DEC constraints Lower Sunda open	2	-478.371	960.805	1.48277E-23	0.00%
DEC+J constraints Lower Sunda open	3	-443.583	893.293	6.77799E-09	0.00%
DEC+x allDist unconstrained	3	-474.134	954.396	3.65338E-22	0.00%
DEC+J+x allDist unconstrained	4	-429.830	867.874	0.00224255	0.22%
DEC+x constraints	3	-459.796	925.720	6.16275E-16	0.00%
DEC+J+x constraints	4	-429.685	867.584	0.002592236	0.26%
DEC+x constraints Lower Sunda open	3	-474.036	954.200	4.02963E-22	0.00%
DEC+J+x constraints Lower Sunda open	4	-429.786	867.786	0.002344212	0.23%

BioGeoBEARS results table

MODEL non-time stratified 30Ma	LnL	numparams	d	e	j	x
DEC	-352.5321107	2	0.001804609	0.002237991	0	0
DEC+J	-252.5532528	3	1.00E-15	1.00E-15	0.016144995	0
DEC constraints	-357.3253909	2	0.00323435	0.006323042	0	0
DEC+J constraints	-291.3294666	3	0.00044759	0.002024199	0.028052577	0
DEC constraints Lower Sunda open	-345.037964	2	0.002696973	0.003902033	0	0
DEC+J constraints Lower Sunda open	-255.861	3	0.000135066	0.002158548	0.01586227	0
DEC+x allDist unconstrained	-350.6169854	3	0.002280324	0.002146059	0	-0.034500434
DEC+J+x allDist unconstrained	-230.6397228	4	1.00E-15	1.00E-15	0.175770261	-0.392820086
DEC+x constraints	-351.0215677	3	0.002387943	0.002959685	0	-0.026967085
DEC+J+x constraints	-226.4089459	4	1.00E-15	1.00E-15	0.700061134	-0.62151675
DEC+x constraints Lower Sunda open	-350.9984188	3	0.002080811	0.001808575	0	-0.020800079
DEC+J+x constraints Lower Sunda open	-220.3185825	4	1.00E-15	1.00E-15	0.487842775	-0.610014842

MODEL time stratified 30Ma	LnL	numparams	d	e	j	x
DEC	-476.0946797	2	0.003377692	0.019364703	0	0
DEC+J	-429.9861663	3	0.001427139	0.011526146	0.022206813	0
DEC constraints	-472.2326988	2	0.005436496	0.020452513	0	0
DEC+J constraints	-424.7731314	3	0.00225242	0.011714398	0.033641868	0
DEC constraints Lower Sunda open	-478.3707587	2	0.004699242	0.021411811	0	0
DEC+J constraints Lower Sunda open	-443.5827169	3	0.00236686	0.013848154	0.02324906	0
DEC+x allDist unconstrained	-474.1343498	3	0.004480022	0.019119747	0	-0.044133195
DEC+J+x allDist unconstrained	-429.830151	4	0.001443845	0.010733527	0.02244249	-0.002856951
DEC+x constraints	-459.7959692	3	0.030761198	0.017730404	0	-0.336720857
DEC+J+x constraints	-429.6852436	4	0.00148032	0.011006807	0.021379046	-0.004441819
DEC+x constraints Lower Sunda open	-474.0363287	3	0.004181548	0.018251131	0	-0.037228098
DEC+J+x constraints Lower Sunda open	-429.7858154	4	0.001447799	0.010940082	0.022482711	-0.003096464

Likelihood ratio test results between pairs of models and AIC ratio weights.

non-time stratified 30Ma

alt	null	LnLalt	LnLnull	DFalt	DFnull	DF	Dstatistic	pval	test	tail	AIC1	AIC2	AICwt1	AICwt2	AICweight_ratio_model1	AICweight_ratio_model2
DEC+J	DEC	-252.6	-352.5	3	2	1	200	2.10E-45	chi-squared	one-tailed	511.1	709.1	1	1.00E-43	9.68E+42	1.00E-43
DEC+J constraints	DEC constraints	-291.3	-357.3	3	2	1	132	1.50E-30	chi-squared	one-tailed	588.7	718.7	1	5.90E-29	1.69E+28	5.90E-29
DEC+J constraints Lower Sunda open	DEC constraints Lower Sunda open	-255.9	-345	3	2	1	178.4	1.10E-40	chi-squared	one-tailed	517.7	694.1	1	5.10E-39	1.97E+38	5.10E-39
DEC+J+x allDist unconstrained	DECx allDist unconstrained	-230.6	-350.6	4	3	1	240	4.00E-54	chi-squared	one-tailed	469.3	707.2	1	2.10E-52	4.69E+51	2.10E-52
DEC+J+x constraints	DEC+x constraints	-226.4	-351	4	3	1	249.2	3.80E-56	chi-squared	one-tailed	460.8	708	1	2.10E-54	4.83E+53	2.10E-54
DEC+J+x constraints Lower Sunda open	DEC+x constraints Lower Sunda open	-220.3	-351	4	3	1	261.4	8.70E-59	chi-squared	one-tailed	448.6	708	1	4.80E-57	2.09E+56	4.80E-57

time stratified 30Ma

alt	null	LnLalt	LnLnull	DFalt	DFnull	DF	Dstatistic	pval	test	tail	AIC1	AIC2	AICwt1	AICwt2	AICweight_ratio_model1	AICweight_ratio_model2
resDECj_time	resDEC_time	-430	-476.1	3	2	1	92.22	7.80E-22	chi-squared	one-tailed	866	956.2	1	2.60E-20	3.89E+19	2.60E-20
resDECj_constraints_time	resDEC_constraints_time	-424.8	-472.2	3	2	1	94.92	2.00E-22	chi-squared	one-tailed	855.5	948.5	1	6.70E-21	1.50E+20	6.70E-21
resDECj_Lower_Sunda_Open_time	resDEC_Lower_Sunda_Open_time	-443.6	-478.4	3	2	1	69.58	7.40E-17	chi-squared	one-tailed	893.2	960.7	1	2.10E-15	4.72E+14	2.10E-15
resDECjx_allopen_time	resDECx_allopen_time	-429.8	-474.1	4	3	1	88.61	4.80E-21	chi-squared	one-tailed	867.7	954.3	1	1.60E-19	6.41E+18	1.60E-19
resDECjx_constraints_time	resDECx_constraints_time	-429.7	-459.8	4	3	1	60.22	8.50E-15	chi-squared	one-tailed	867.4	925.6	1	2.30E-13	4.39E+12	2.30E-13
resDECjx_Lower_Sunda_Open_time	resDECx_Lower_Sunda_Open_time	-429.8	-474	4	3	1	88.5	5.10E-21	chi-squared	one-tailed	867.6	954.1	1	1.60E-19	6.07E+18	1.60E-19

BioGeoBEARS DEC on Trigonopterus
ancstates: global optim, 3 areas max. d=0.0018; e=0.0022; j=0; x=0; u=0; LnL=−352.53

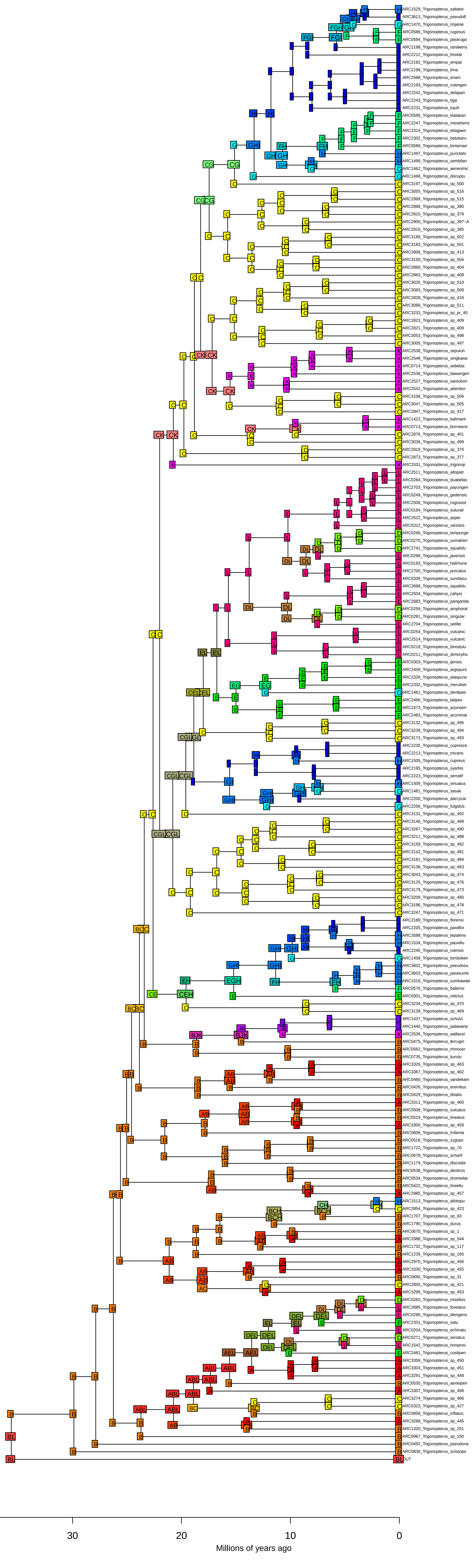

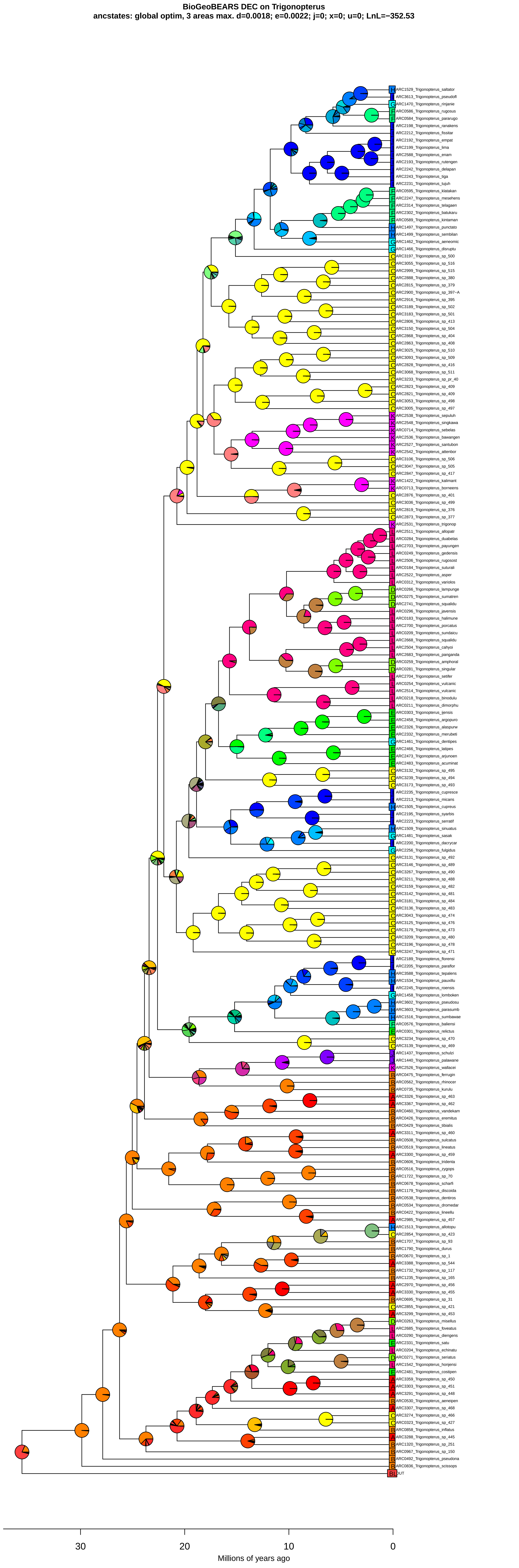
BioGeoBEARS DEC on Trigonopterus
ancstates: global optim, 3 areas max. d=0.0018; e=0.0022; j=0; x=0; u=0; LnL=−352.53
30
20
10
0
Millions of years ago

BioGeoBEARS DEC+J on Trigonopterus
ancstates: global optim, 3 areas max. d=0; e=0; j=0.0161; x=0; u=0; LnL=−252.55

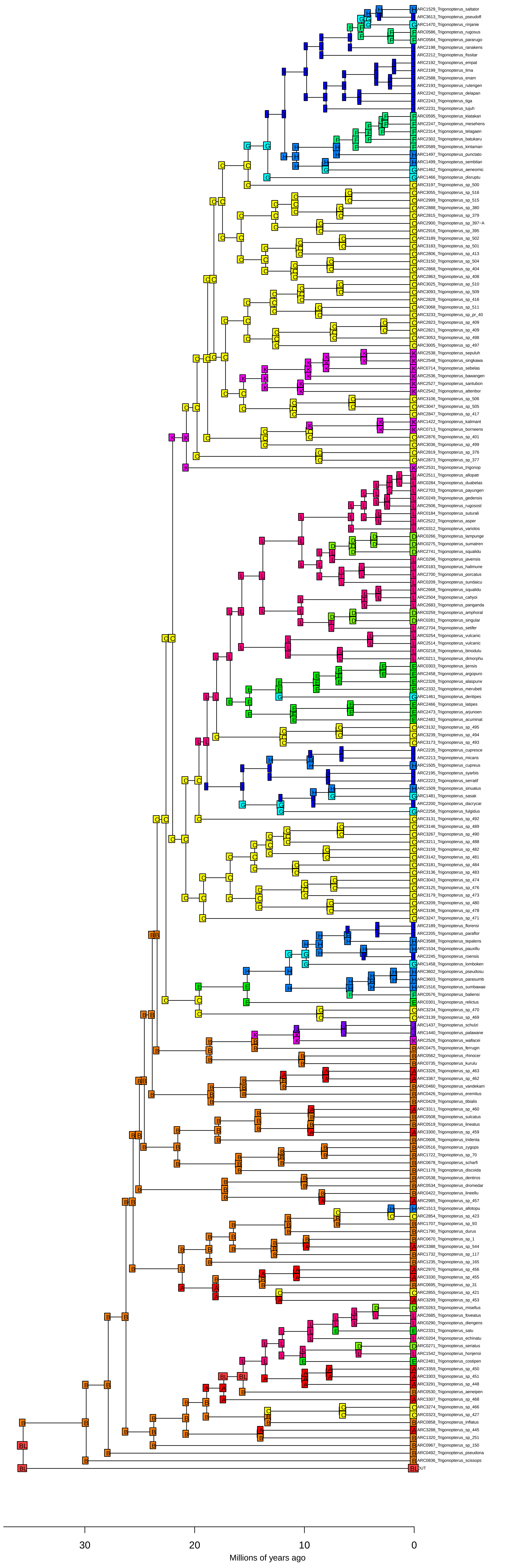

BioGeoBEARS DEC+J on Trigonopterus
ancstates: global optim, 3 areas max. d=0; e=0; j=0.0161; x=0; u=0; LnL=−252.55

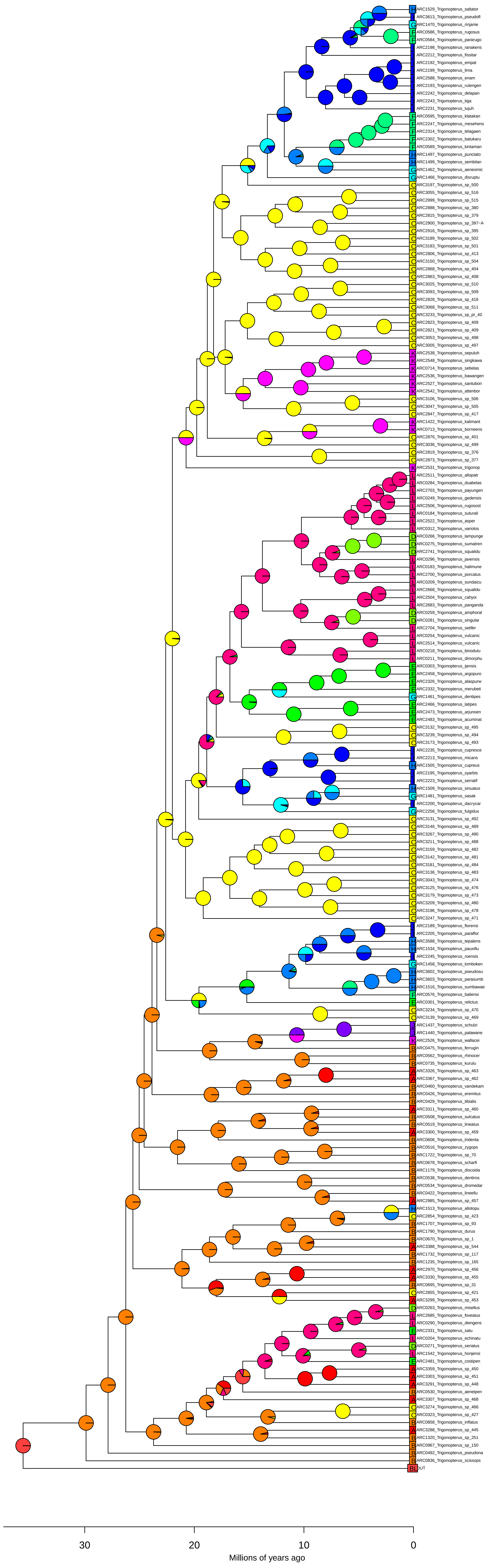

BioGeoBEARS DEC constraints on Trigonopterus
ancstates: global optim, 3 areas max. d=0.0032; e=0.0063; j=0; x=0; u=0; LnL=−357.33

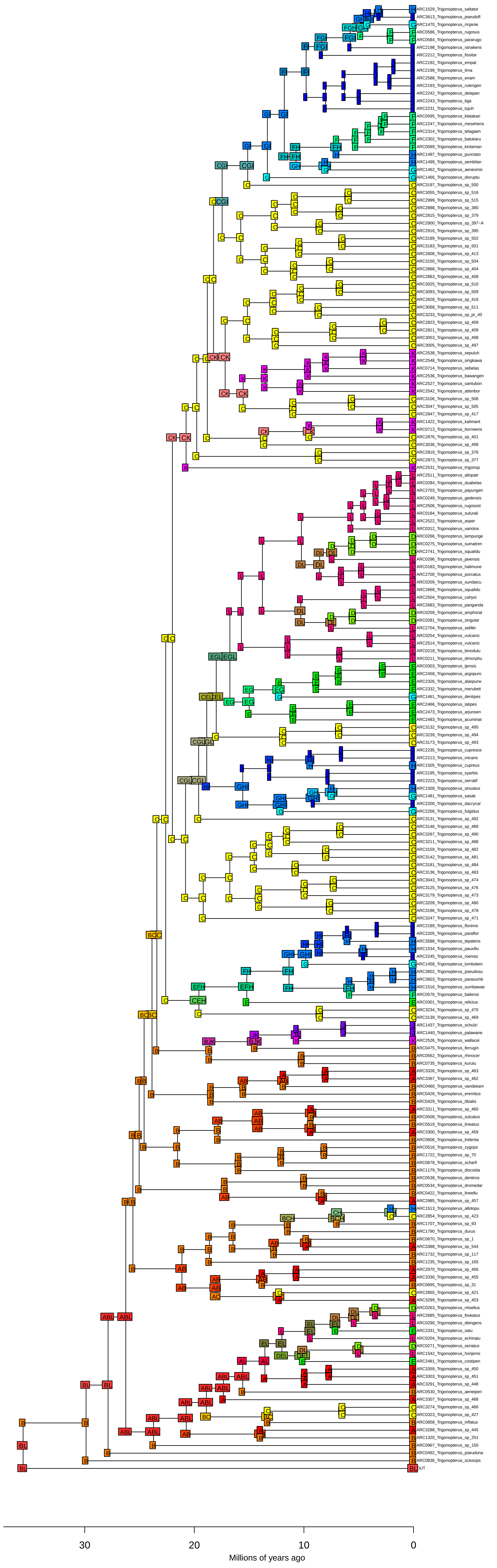

BioGeoBEARS DEC constraints on Trigonopterus
ancstates: global optim, 3 areas max. d=0.0032; e=0.0063; j=0; x=0; u=0; LnL=−357.33

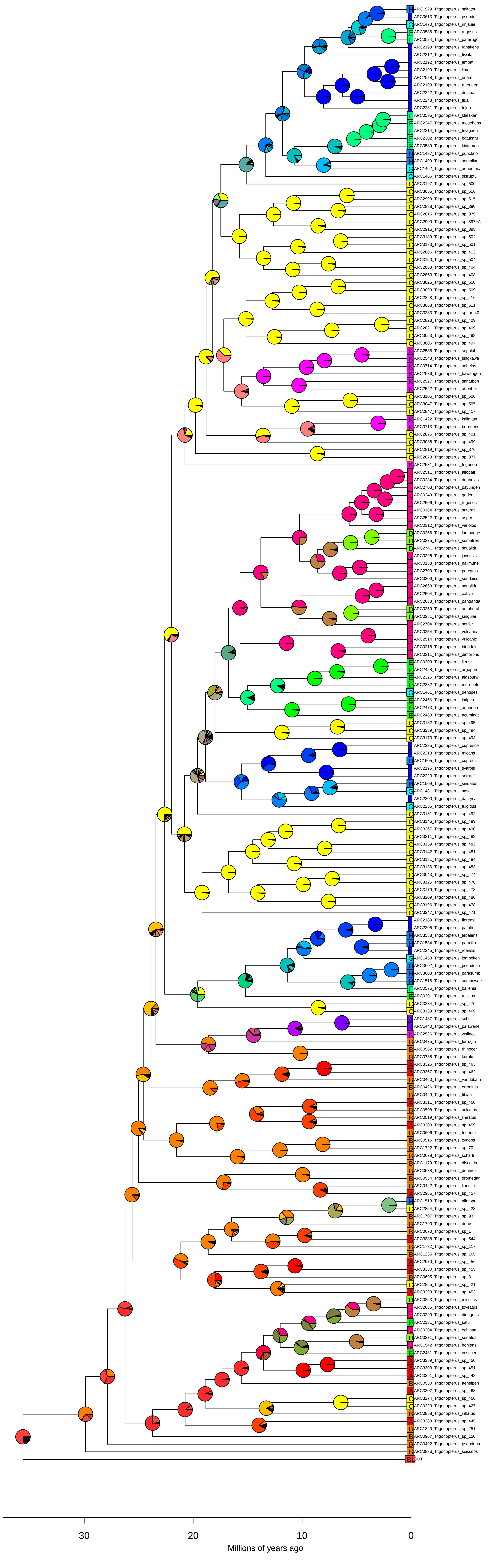

BioGeoBEARS DEC+J constraints on Trigonopterus
ancstates: global optim, 3 areas max. d=4e−04; e=0.002; j=0.0281; x=0; u=0; LnL=−291.33

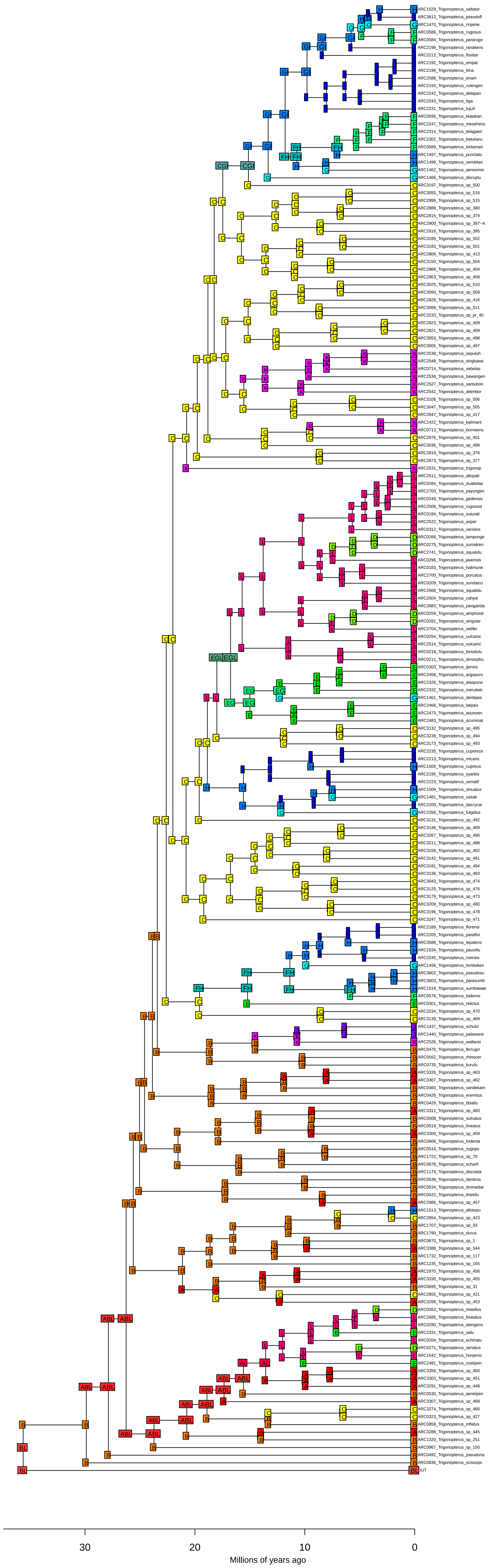

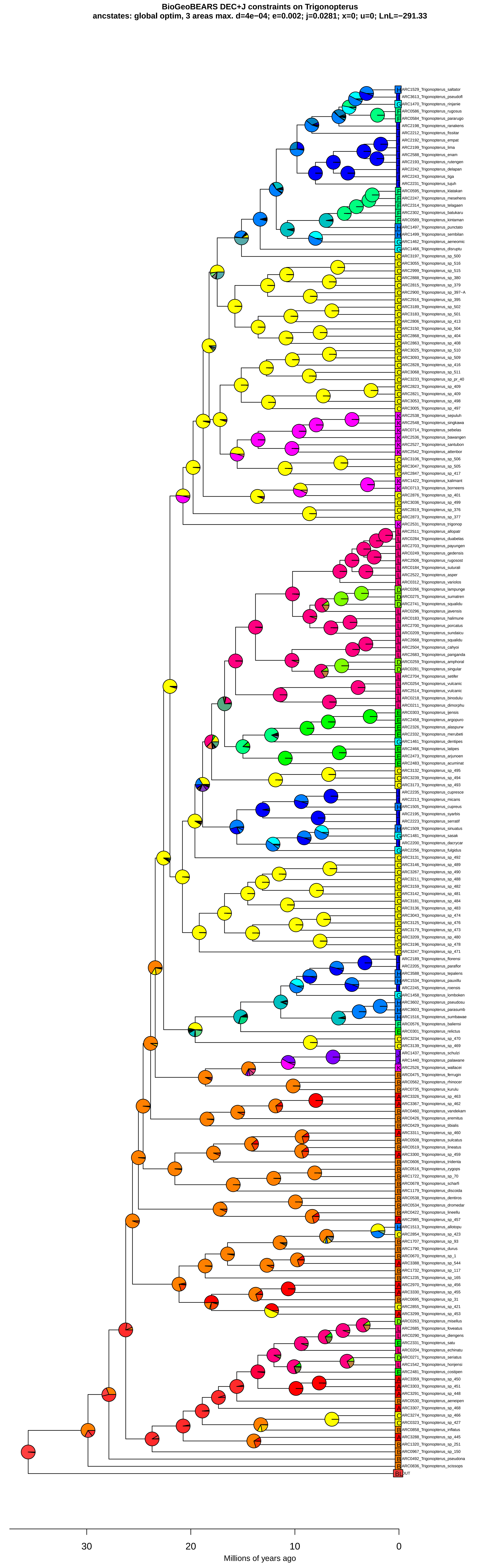
BioGeoBEARS DEC+J constraints on Trigonopterus
ancstates: global optim, 3 areas max. d=4e−04; e=0.002; j=0.0281; x=0; u=0; LnL=−291.33
ARC1529_Trigonopterus_saltator
ARC3613_Trigonopterus_pseudofl
ARC1470_Trigonopterus_rinjanie
ARC0586_Trigonopterus_rugosus
ARC0584_Trigonopterus_pararugo
ARC2198_Trigonopterus_ranakens
ARC2212_Trigonopterus_fissitar
ARC2192_Trigonopterus_empat
ARC2199_Trigonopterus_lima
ARC2588_Trigonopterus_enam
ARC2193_Trigonopterus_rutengen
ARC2242_Trigonopterus_delapan
ARC2243_Trigonopterus_tiga
ARC2231_Trigonopterus_tujuh
ARC0595_Trigonopterus_klatakan
ARC2247_Trigonopterus_mesehens
ARC2314_Trigonopterus_telagaen
ARC2302_Trigonopterus_batukaru
ARC0589_Trigonopterus_kintaman
ARC1497_Trigonopterus_punctato
ARC1499_Trigonopterus_sembilan
ARC1462_Trigonopterus_aeneomic
ARC1466_Trigonopterus_disruptu
ARC3197_Trigonopterus_sp_500
ARC3055_Trigonopterus_sp_516
ARC2999_Trigonopterus_sp_515
ARC2888_Trigonopterus_sp_380
ARC2815_Trigonopterus_sp_379
ARC2900_Trigonopterus_sp_397−A
ARC2916_Trigonopterus_sp_395
ARC3189_Trigonopterus_sp_502
ARC3183_Trigonopterus_sp_501
ARC2806_Trigonopterus_sp_413
ARC3150_Trigonopterus_sp_504
ARC2868_Trigonopterus_sp_404
ARC2863_Trigonopterus_sp_408
ARC3025_Trigonopterus_sp_510
ARC3093_Trigonopterus_sp_509
ARC2828_Trigonopterus_sp_416
ARC3068_Trigonopterus_sp_511
ARC3233_Trigonopterus_sp_pr_40
ARC2823_Trigonopterus_sp_409
ARC2821_Trigonopterus_sp_409
ARC3053_Trigonopterus_sp_498
ARC3005_Trigonopterus_sp_497
ARC2538_Trigonopterus_sepuluh
ARC2548_Trigonopterus_singkawa
ARC0714_Trigonopterus_sebelas
ARC2536_Trigonopterus_bawangen
ARC2527_Trigonopterus_santubon
ARC2542_Trigonopterus_attenbor
ARC3106_Trigonopterus_sp_506
ARC3047_Trigonopterus_sp_505
ARC2847_Trigonopterus_sp_417
ARC1422_Trigonopterus_kalimant
ARC0713_Trigonopterus_borneens
ARC2876_Trigonopterus_sp_401
ARC3036_Trigonopterus_sp_499
ARC2819_Trigonopterus_sp_376
ARC2873_Trigonopterus_sp_377
ARC2531_Trigonopterus_trigonop
ARC2511_Trigonopterus_allopatr
ARC0284_Trigonopterus_duabelas
ARC2703_Trigonopterus_payungen
ARC0249_Trigonopterus_gedensis
ARC2506_Trigonopterus_rugosost
ARC0184_Trigonopterus_suturali
ARC2522_Trigonopterus_asper
ARC0312_Trigonopterus_variolos
ARC0266_Trigonopterus_lampunge
ARC0275_Trigonopterus_sumatren
ARC2741_Trigonopterus_squalidu
ARC0296_Trigonopterus_javensis
ARC0183_Trigonopterus_halimune
ARC2700_Trigonopterus_porcatus
ARC0209_Trigonopterus_sundaicu
ARC2668_Trigonopterus_squalidu
ARC2504_Trigonopterus_cahyoi
ARC2683_Trigonopterus_panganda
ARC0259_Trigonopterus_amphoral
ARC0281_Trigonopterus_singular
ARC2704_Trigonopterus_setifer
ARC0254_Trigonopterus_vulcanic
ARC2514_Trigonopterus_vulcanic
ARC0218_Trigonopterus_binodulu
ARC0211_Trigonopterus_dimorphu
ARC0303_Trigonopterus_ijensis
ARC2458_Trigonopterus_argopuro
ARC2326_Trigonopterus_alaspurw
ARC2332_Trigonopterus_merubeti
ARC1461_Trigonopterus_dentipes
ARC2466_Trigonopterus_latipes
ARC2473_Trigonopterus_arjunoen
ARC2483_Trigonopterus_acuminat
ARC3132_Trigonopterus_sp_495
ARC3239_Trigonopterus_sp_494
ARC3173_Trigonopterus_sp_493
ARC2235_Trigonopterus_cupresce
ARC2213_Trigonopterus_micans
ARC1505_Trigonopterus_cupreus
ARC2195_Trigonopterus_syarbis
ARC2223_Trigonopterus_serratif
ARC1509_Trigonopterus_sinuatus
ARC1481_Trigonopterus_sasak
ARC2200_Trigonopterus_dacrycar
ARC2256_Trigonopterus_fulgidus
ARC3131_Trigonopterus_sp_492
ARC3146_Trigonopterus_sp_489
ARC3267_Trigonopterus_sp_490
ARC3211_Trigonopterus_sp_488
ARC3159_Trigonopterus_sp_482
ARC3142_Trigonopterus_sp_481
ARC3181_Trigonopterus_sp_484
ARC3136_Trigonopterus_sp_483
ARC3043_Trigonopterus_sp_474
ARC3125_Trigonopterus_sp_476
ARC3179_Trigonopterus_sp_473
ARC3209_Trigonopterus_sp_480
ARC3196_Trigonopterus_sp_478
ARC3247_Trigonopterus_sp_471
ARC2189_Trigonopterus_florensi
ARC2205_Trigonopterus_paraflor
ARC3588_Trigonopterus_tepalens
ARC1534_Trigonopterus_pauxillu
ARC2245_Trigonopterus_roensis
ARC1458_Trigonopterus_lomboken
ARC3602_Trigonopterus_pseudosu
ARC3603_Trigonopterus_parasumb
ARC1516_Trigonopterus_sumbawae
ARC0576_Trigonopterus_baliensi
ARC0301_Trigonopterus_relictus
ARC3234_Trigonopterus_sp_470
ARC3139_Trigonopterus_sp_469
ARC1437_Trigonopterus_schulzi
ARC1440_Trigonopterus_palawane
ARC2526_Trigonopterus_wallacei
ARC0475_Trigonopterus_ferrugin
ARC0562_Trigonopterus_rhinocer
ARC0735_Trigonopterus_kurulu
ARC3326_Trigonopterus_sp_463
ARC3367_Trigonopterus_sp_462
ARC0460_Trigonopterus_vandekam
ARC0426_Trigonopterus_eremitus
ARC0429_Trigonopterus_tibialis
ARC3311_Trigonopterus_sp_460
ARC0508_Trigonopterus_sulcatus
ARC0519_Trigonopterus_lineatus
ARC3300_Trigonopterus_sp_459
ARC0606_Trigonopterus_tridenta
ARC0516_Trigonopterus_zygops
ARC1722_Trigonopterus_sp_70
ARC0678_Trigonopterus_scharfi
ARC1179_Trigonopterus_discoida
ARC0538_Trigonopterus_dentiros
ARC0534_Trigonopterus_dromedar
ARC0422_Trigonopterus_lineellu
ARC2985_Trigonopterus_sp_457
ARC1513_Trigonopterus_allotopu
ARC2854_Trigonopterus_sp_423
ARC1707_Trigonopterus_sp_93
ARC1790_Trigonopterus_durus
ARC0670_Trigonopterus_sp_1
ARC3388_Trigonopterus_sp_544
ARC1732_Trigonopterus_sp_117
ARC1235_Trigonopterus_sp_165
ARC2970_Trigonopterus_sp_456
ARC3330_Trigonopterus_sp_455
ARC0695_Trigonopterus_sp_31
ARC2855_Trigonopterus_sp_421
ARC3299_Trigonopterus_sp_453
ARC0263_Trigonopterus_misellus
ARC2685_Trigonopterus_foveatus
ARC0290_Trigonopterus_diengens
ARC2331_Trigonopterus_satu
ARC0204_Trigonopterus_echinatu
ARC0271_Trigonopterus_seriatus
ARC1542_Trigonopterus_honjensi
ARC2481_Trigonopterus_costipen
ARC3359_Trigonopterus_sp_450
ARC3303_Trigonopterus_sp_451
ARC3291_Trigonopterus_sp_448
ARC0530_Trigonopterus_aeneipen
ARC3307_Trigonopterus_sp_468
ARC3274_Trigonopterus_sp_466
ARC0323_Trigonopterus_sp_427
ARC0858_Trigonopterus_inflatus
ARC3288_Trigonopterus_sp_445
ARC1320_Trigonopterus_sp_251
ARC0967_Trigonopterus_sp_150
ARC0492_Trigonopterus_pseudona
ARC0836_Trigonopterus_scissops
BL
OUT
30
20
10
0
Millions of years ago

BioGeoBEARS DEC constraints Lower Sunda Open on Trigonopterus
ancstates: global optim, 3 areas max. d=0.0027; e=0.0039; j=0; x=0; u=0; LnL=−345.04

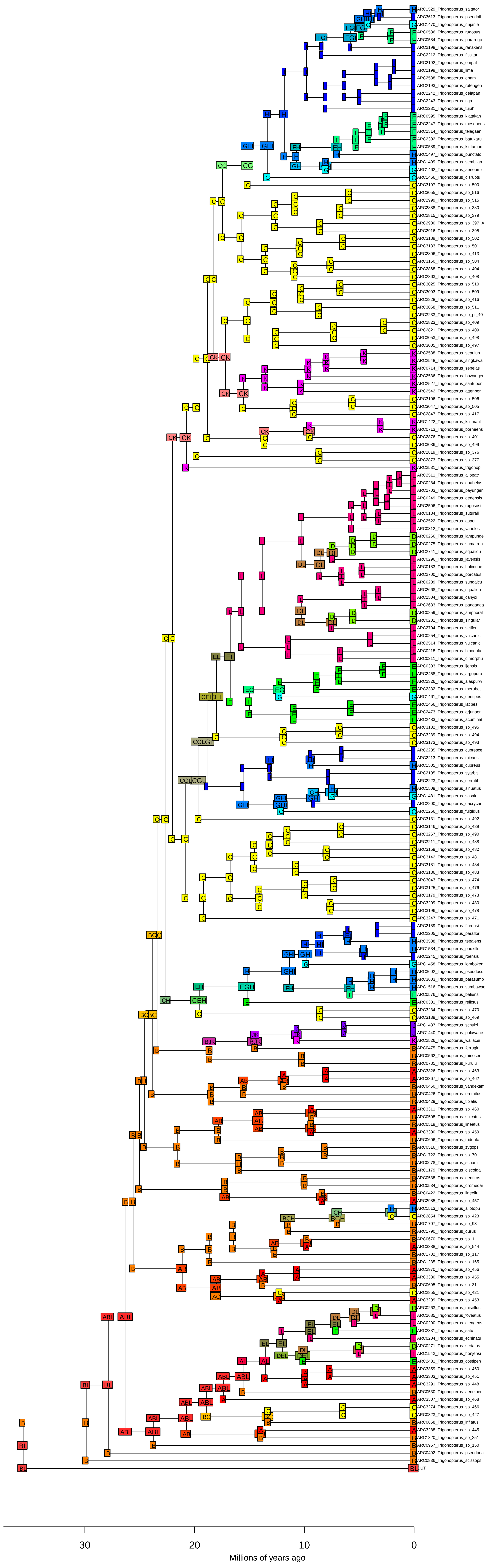

BioGeoBEARS DEC constraints Lower Sunda Open on Trigonopterus
ancstates: global optim, 3 areas max. d=0.0027; e=0.0039; j=0; x=0; u=0; LnL=−345.04

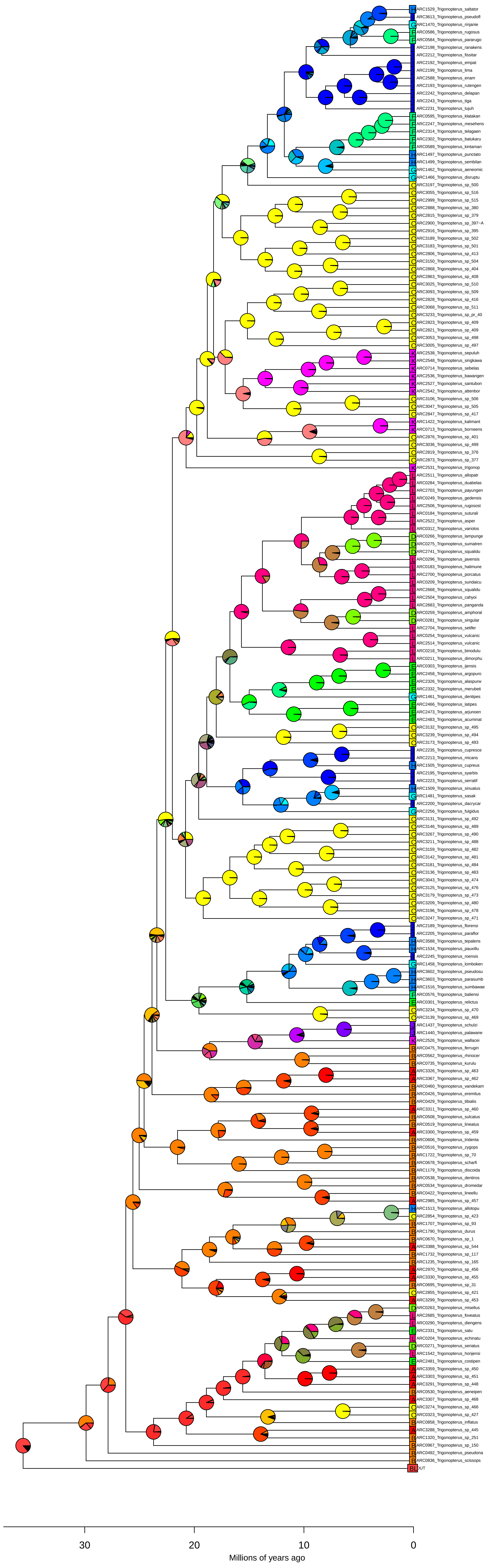

BioGeoBEARS DEC+J constraints Lower Sunda Open on Trigonopterus
ancstates: global optim, 3 areas max. d=1e−04; e=0.0022; j=0.0159; x=0; u=0; LnL=−255.86

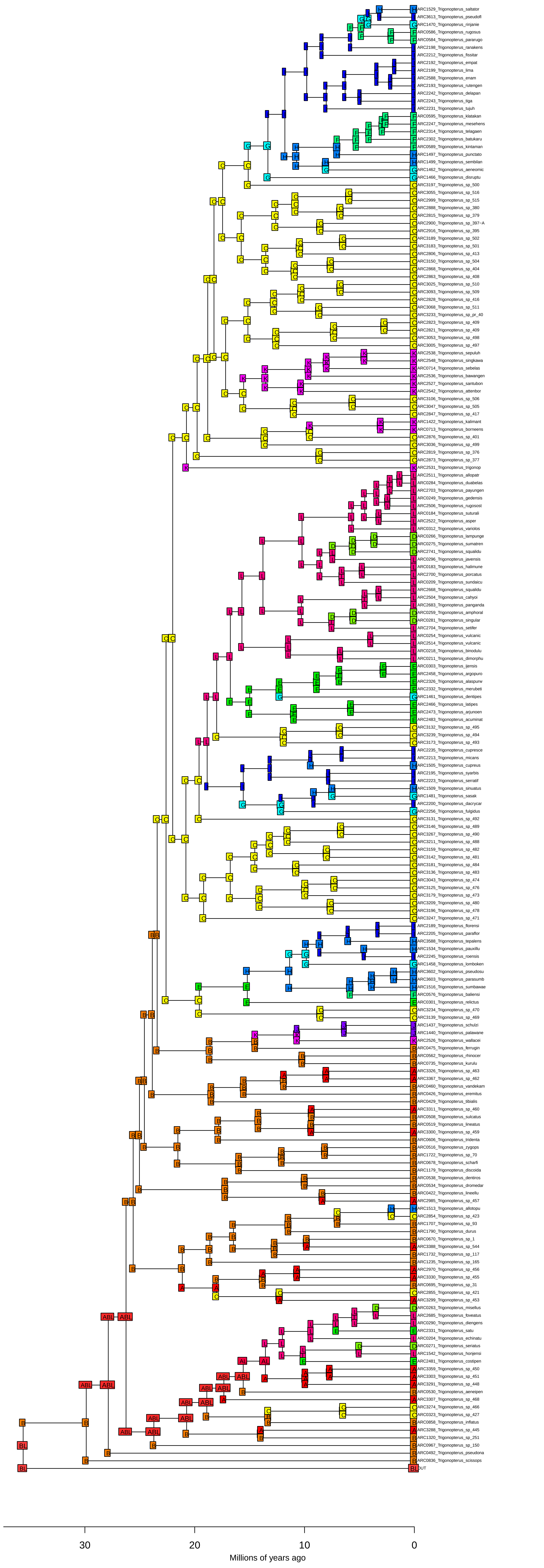

BioGeoBEARS DEC+J constraints Lower Sunda Open on Trigonopterus
ancstates: global optim, 3 areas max. d=1e−04; e=0.0022; j=0.0159; x=0; u=0; LnL=−255.86

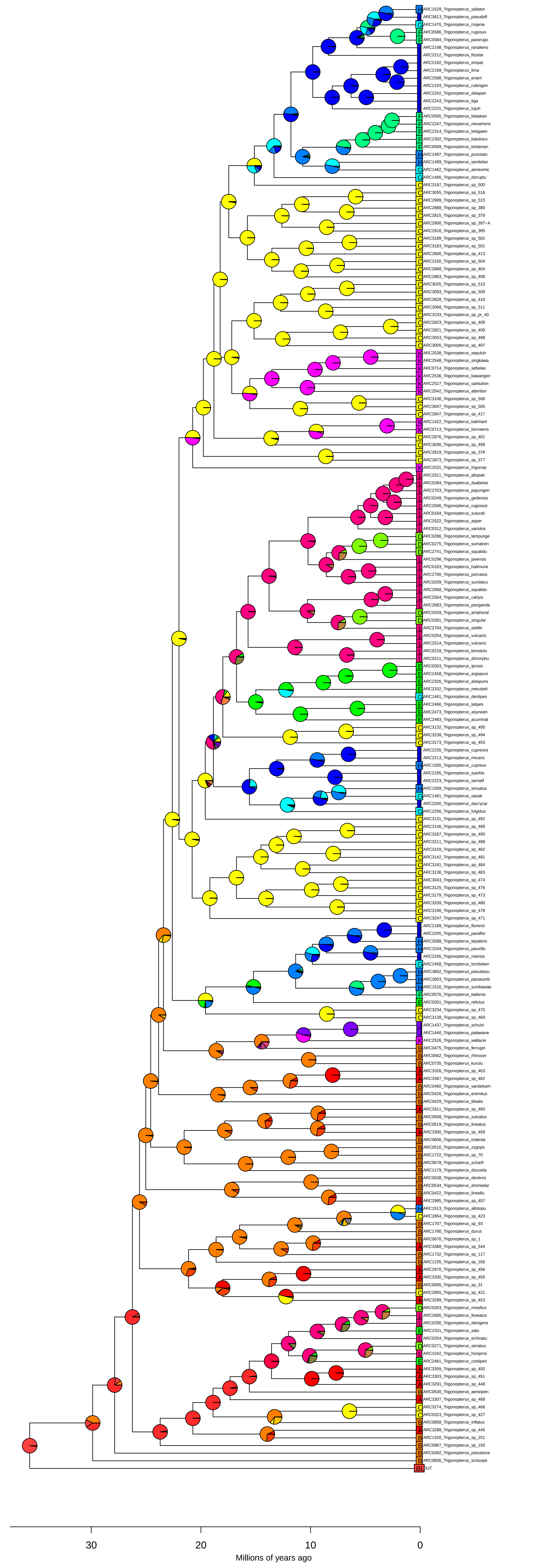

BioGeoBEARS DEC+x all distances on Trigonopterus
ancstates: global optim, 3 areas max. d=0.0023; e=0.0021; x=−0.0345; j=0; u=0; LnL=−350.62

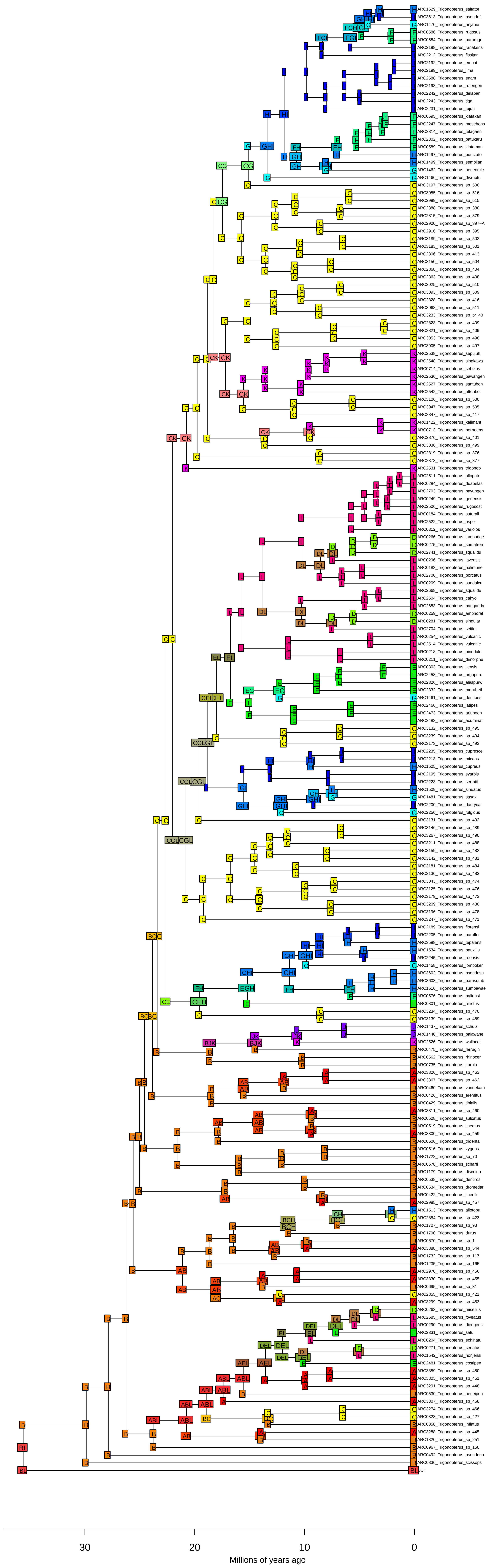

BioGeoBEARS DEC+x all distances on Trigonopterus
ancstates: global optim, 3 areas max. d=0.0023; e=0.0021; x=−0.0345; j=0; u=0; LnL=−350.62

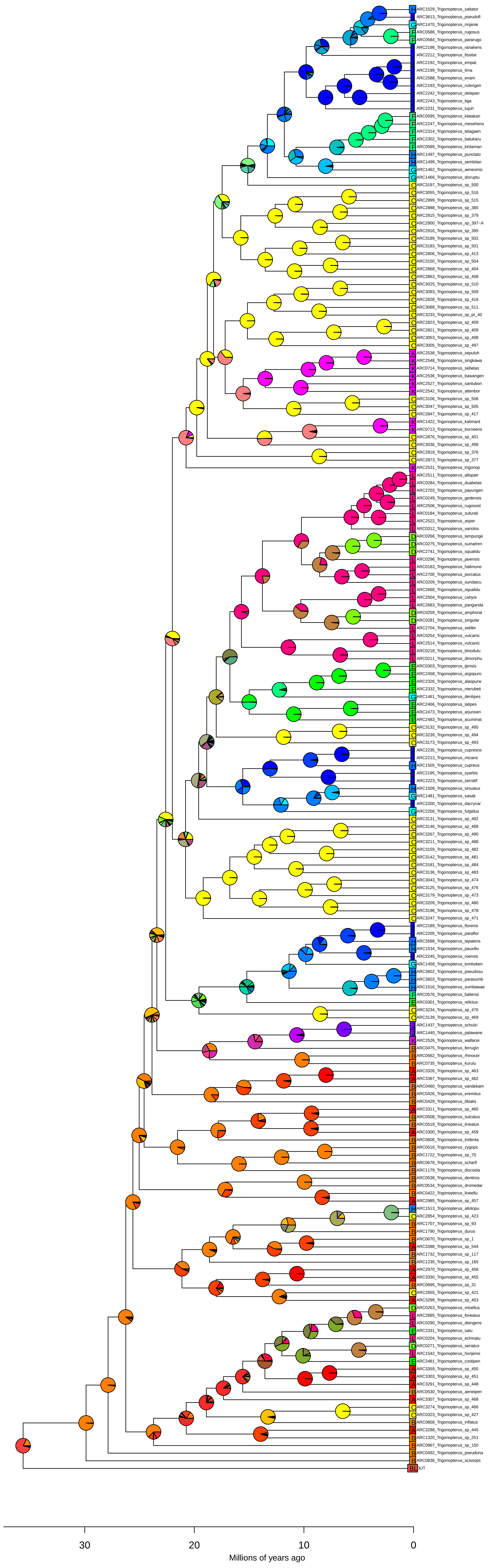

BioGeoBEARS DEC+J+x all distances on Trigonopterus
ancstates: global optim, 3 areas max. d=0; e=0; x=−0.3928; j=0.1758; u=0; LnL=−230.64

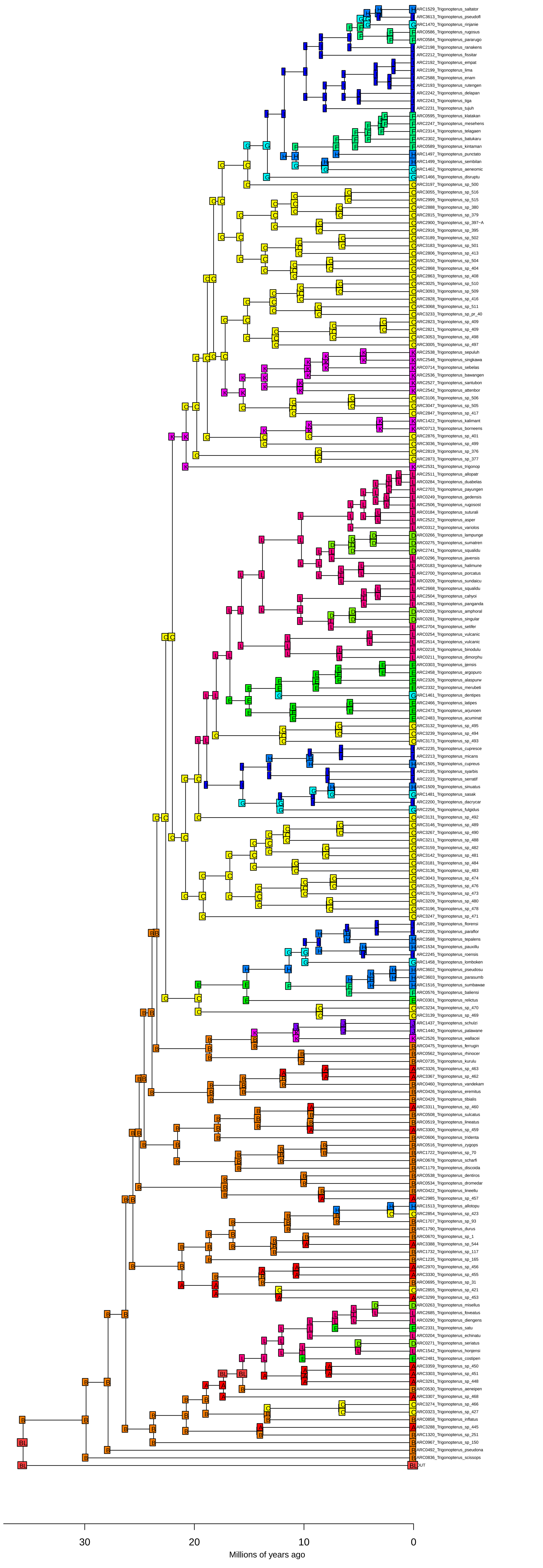

BioGeoBEARS DEC+J+x all distances on Trigonopterus
ancstates: global optim, 3 areas max. d=0; e=0; x=−0.3928; j=0.1758; u=0; LnL=−230.64

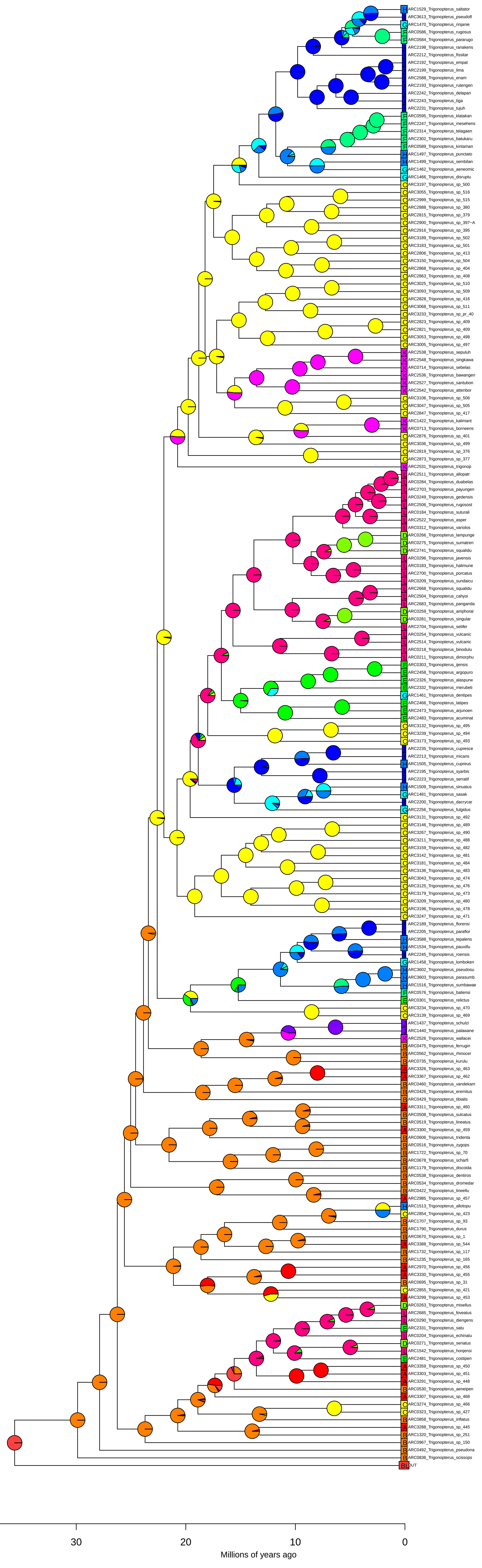

BioGeoBEARS DEC+x constraints on Trigonopterus
ancstates: global optim, 3 areas max. d=0.0024; e=0.003; x=−0.027; j=0; u=0; LnL=−351.02

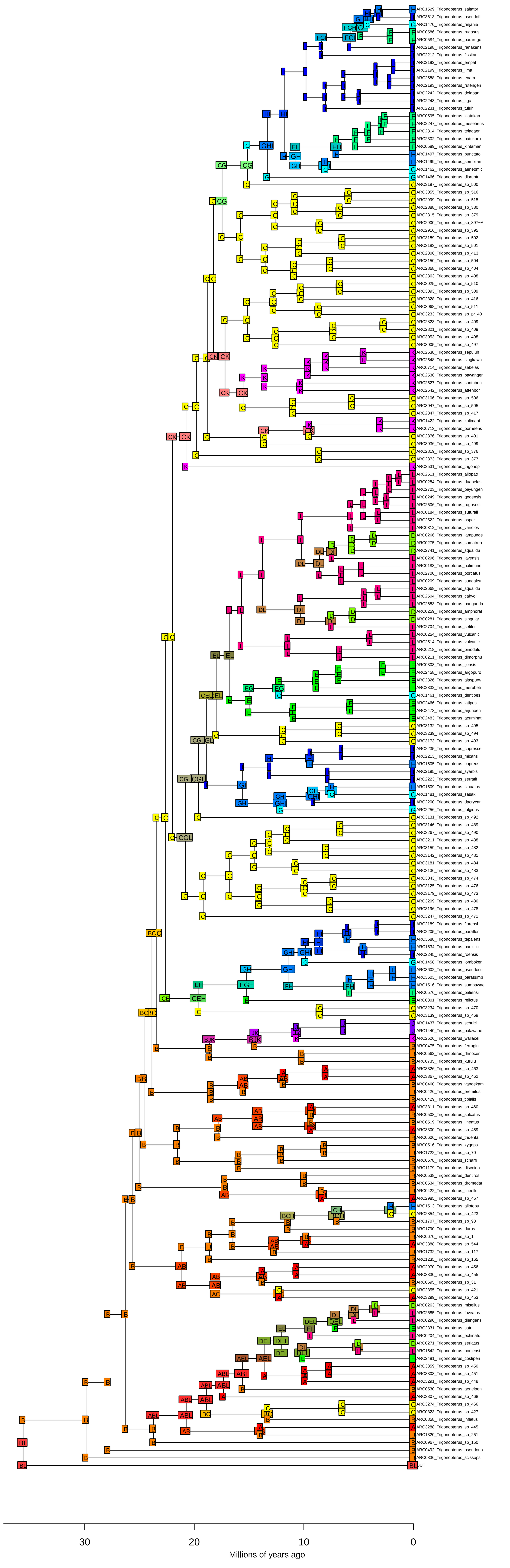

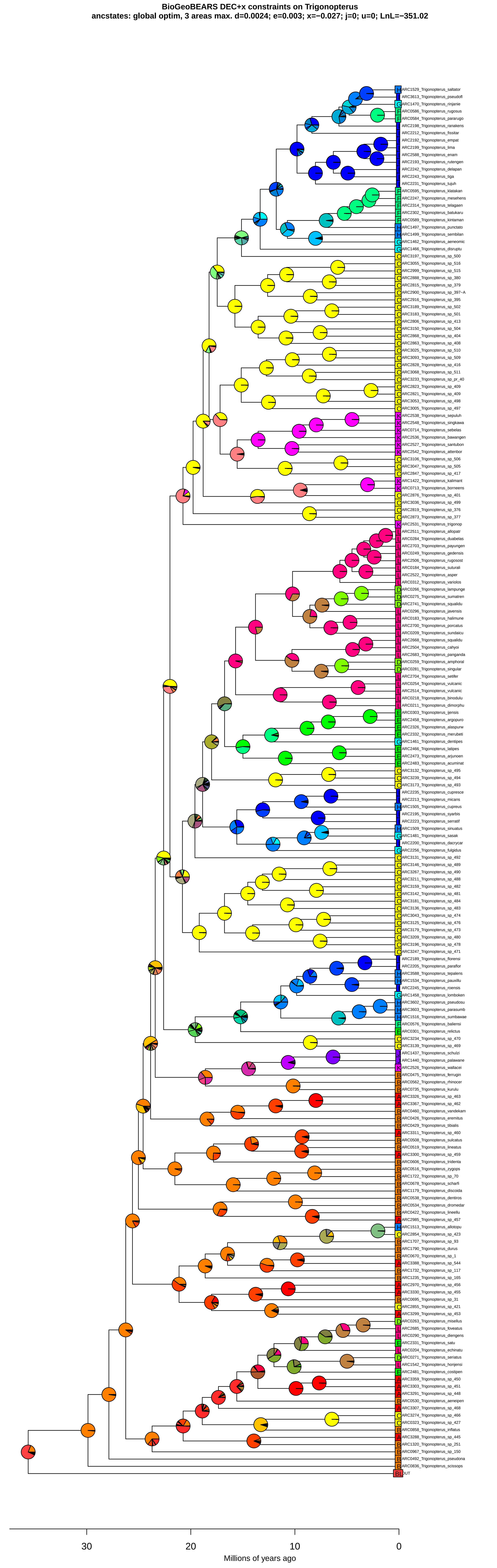
BioGeoBEARS DEC+x constraints on Trigonopterus
ancstates: global optim, 3 areas max. d=0.0024; e=0.003; x=−0.027; j=0; u=0; LnL=−351.02
30
20
10
0
Millions of years ago

BioGeoBEARS DEC+J+x constraints on Trigonopterus
ancstates: global optim, 3 areas max. d=0; e=0; x=−0.6215; j=0.7001; u=0; LnL=−226.41

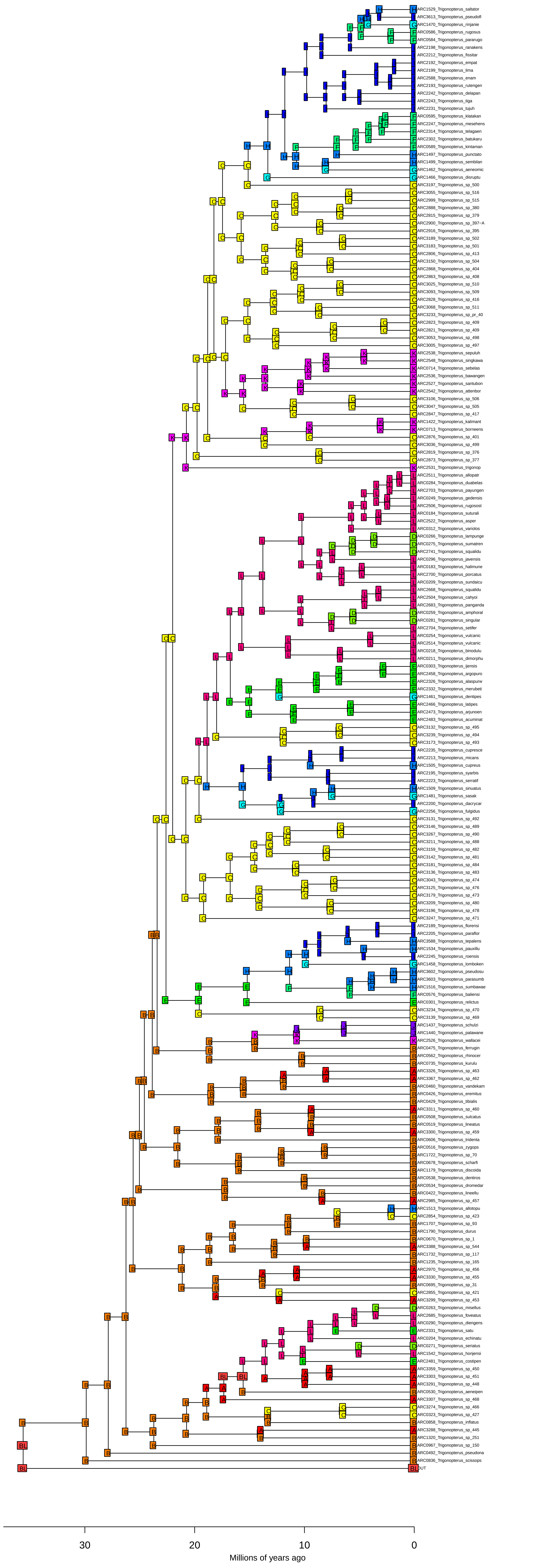

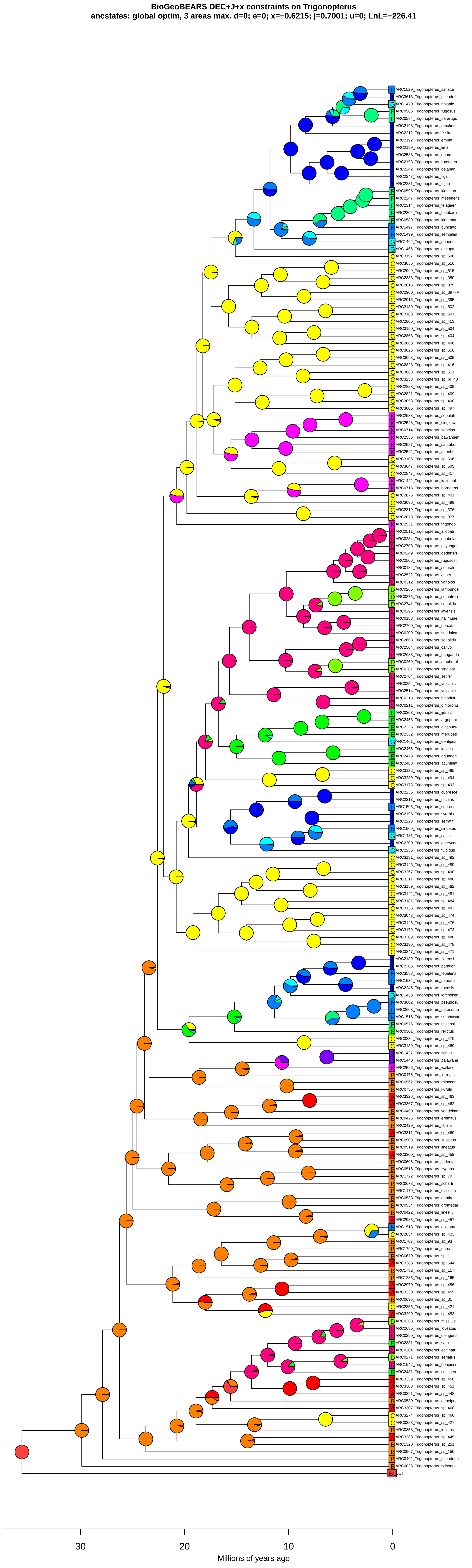
BioGeoBEARS DEC+J+x constraints on Trigonopterus
ancstates: global optim, 3 areas max. d=0; e=0; x=−0.6215; j=0.7001; u=0; LnL=−226.41
30
20
10
0
Millions of years ago

BioGeoBEARS DEC+x Lower Sunda Open on Trigonopterus
ancstates: global optim, 3 areas max. d=0.0021; e=0.0018; x=−0.0208; j=0; u=0; LnL=−351.00

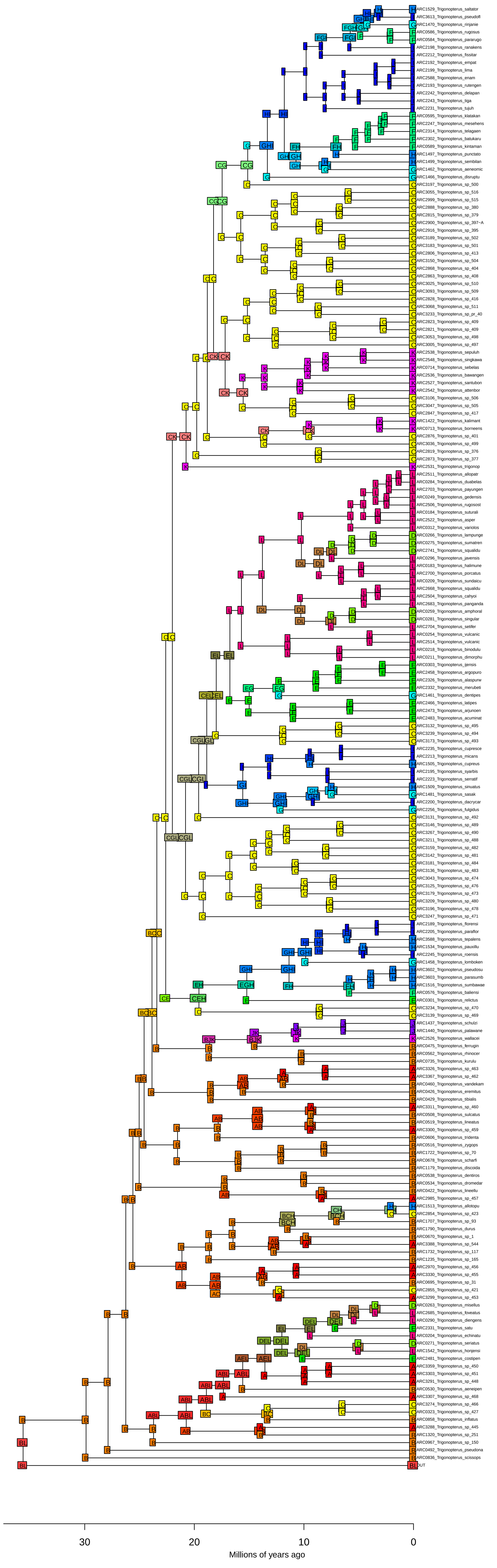

BioGeoBEARS DEC+x Lower Sunda Open on Trigonopterus
ancstates: global optim, 3 areas max. d=0.0021; e=0.0018; x=−0.0208; j=0; u=0; LnL=−351.00

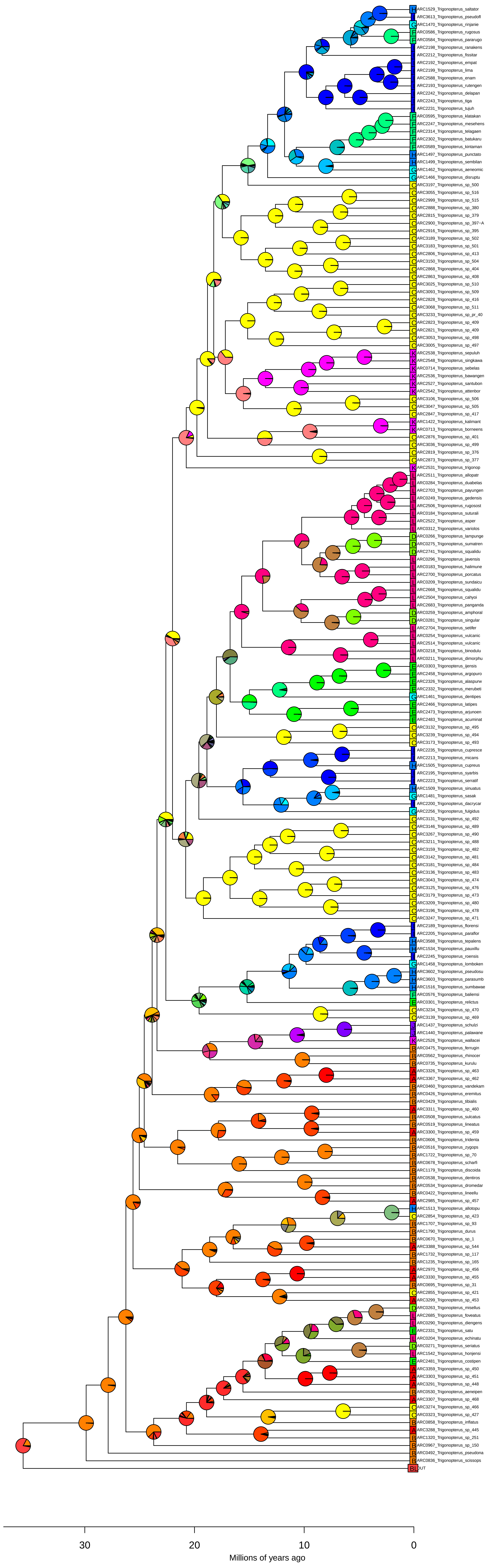

BioGeoBEARS DEC+J+x Lower Sunda Open on Trigonopterus
ancstates: global optim, 3 areas max. d=0; e=0; x=−0.61; j=0.4878; u=0; LnL=−220.32

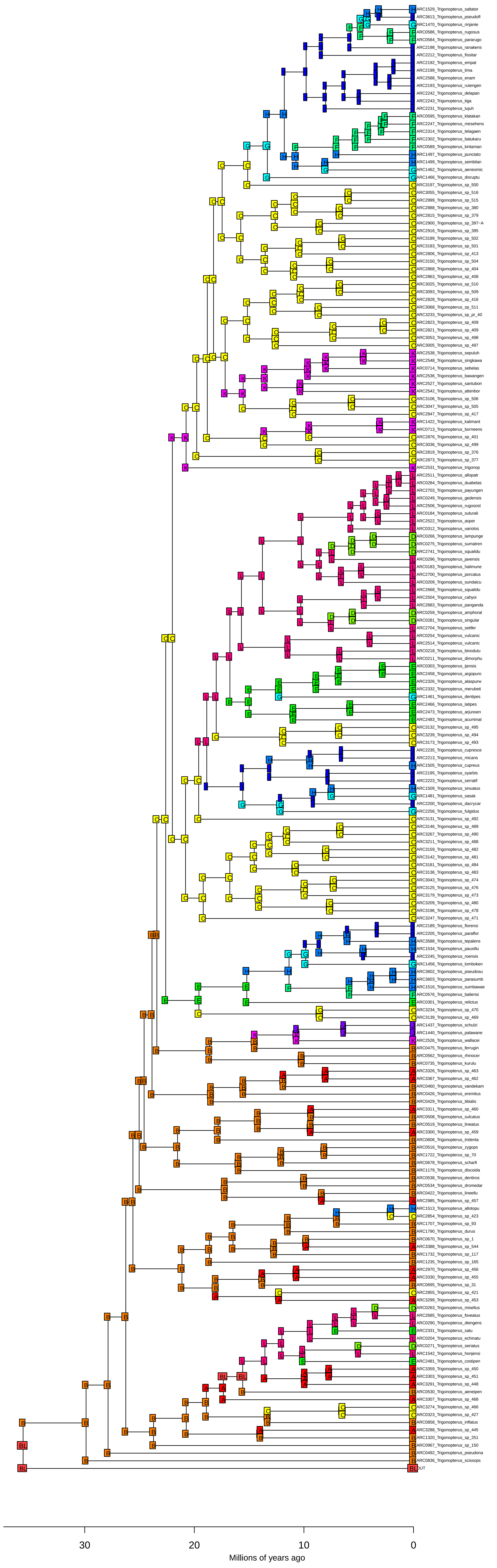

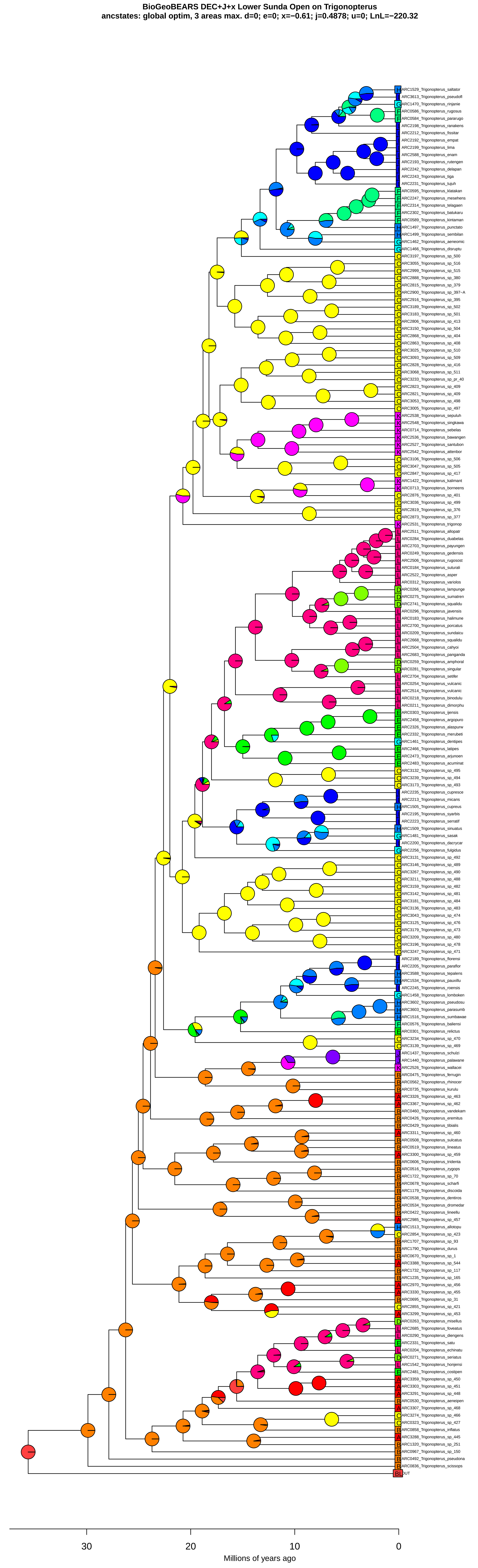
BioGeoBEARS DEC+J+x Lower Sunda Open on Trigonopterus
ancstates: global optim, 3 areas max. d=0; e=0; x=−0.61; j=0.4878; u=0; LnL=−220.32
30
20
10
0
Millions of years ago

BioGeoBEARS DEC on Trigonopterus time slice
ancstates: global optim, 3 areas max. d=0.0034; e=0.0194; j=0; x=0; u=0; LnL=−476.09

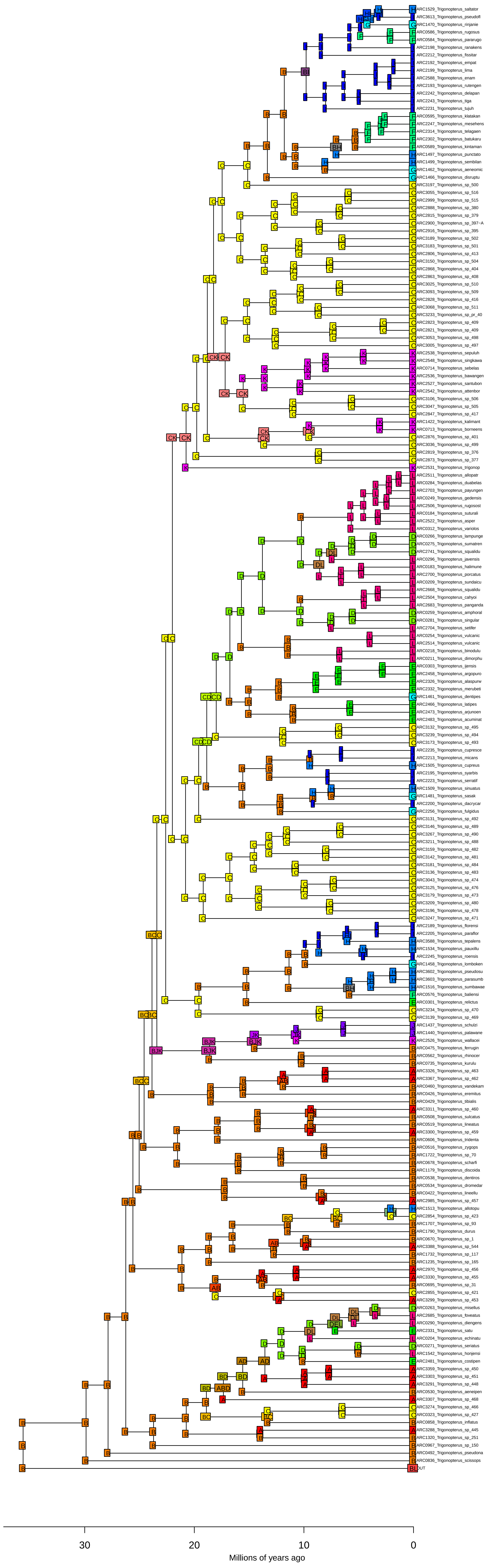

BioGeoBEARS DEC on Trigonopterus time slice
ancstates: global optim, 3 areas max. d=0.0034; e=0.0194; j=0; x=0; u=0; LnL=−476.09

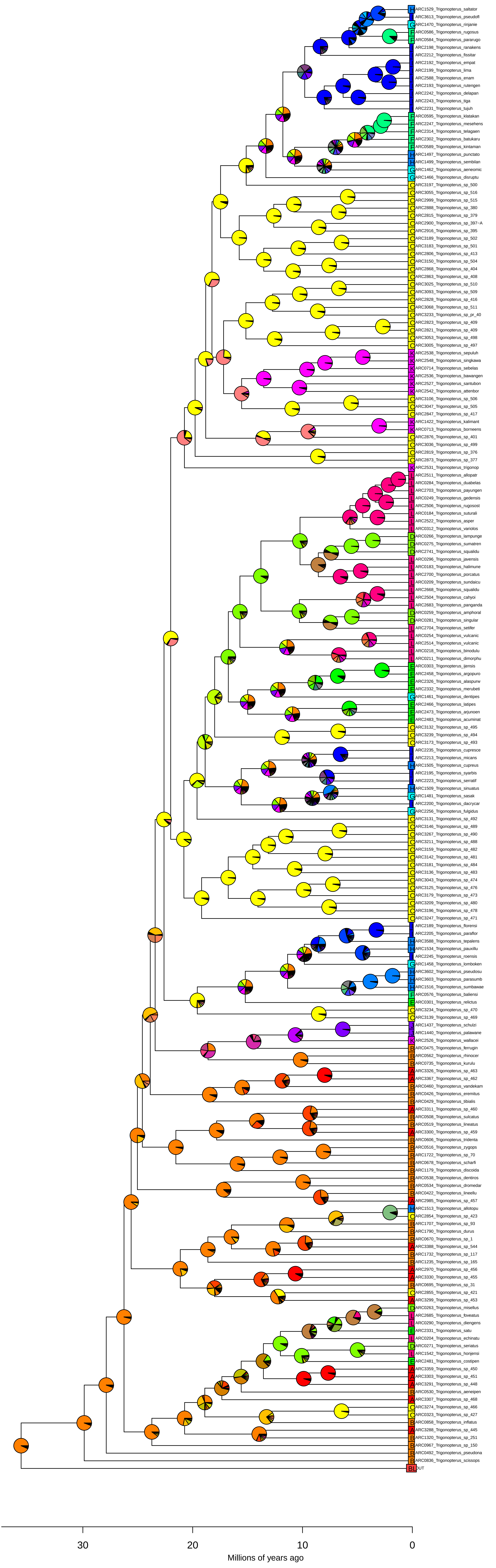

BioGeoBEARS DEC+J on Trigonopterus time slice
ancstates: global optim, 3 areas max. d=0.0014; e=0.0115; j=0.0222; x=0; u=0; LnL=-429.99

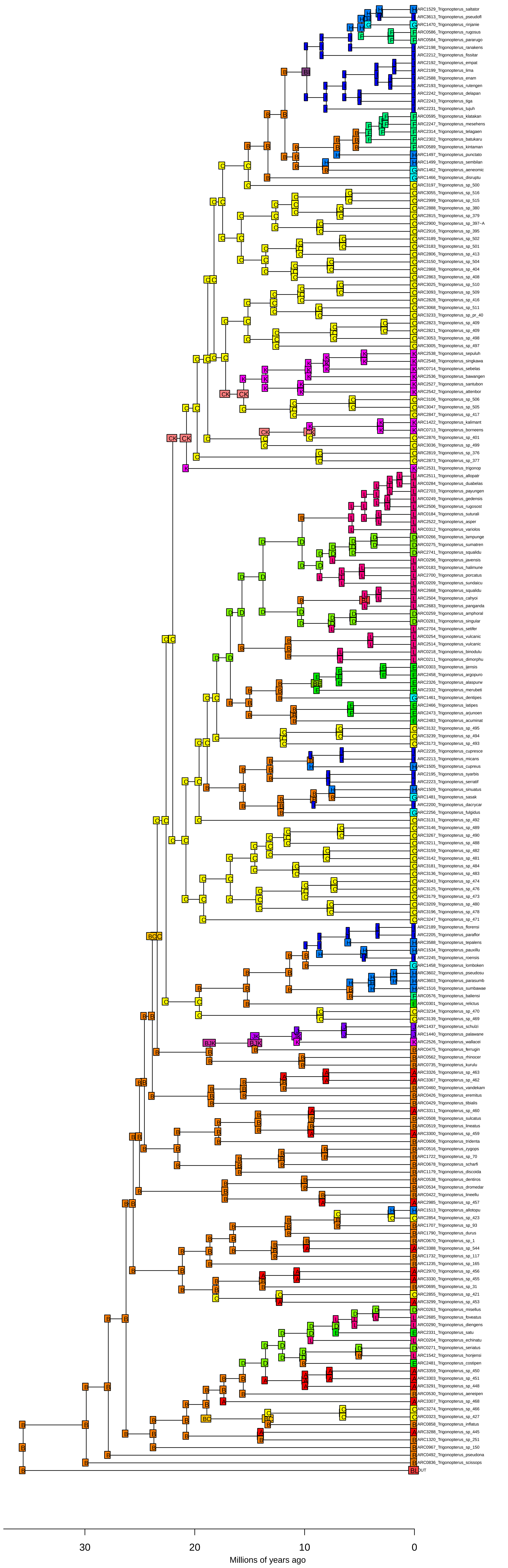

BioGeoBEARS DEC+J on Trigonopterus time slice
ancstates: global optim, 3 areas max. d=0.0014; e=0.0115; j=0.0222; x=0; u=0; LnL=−429.99

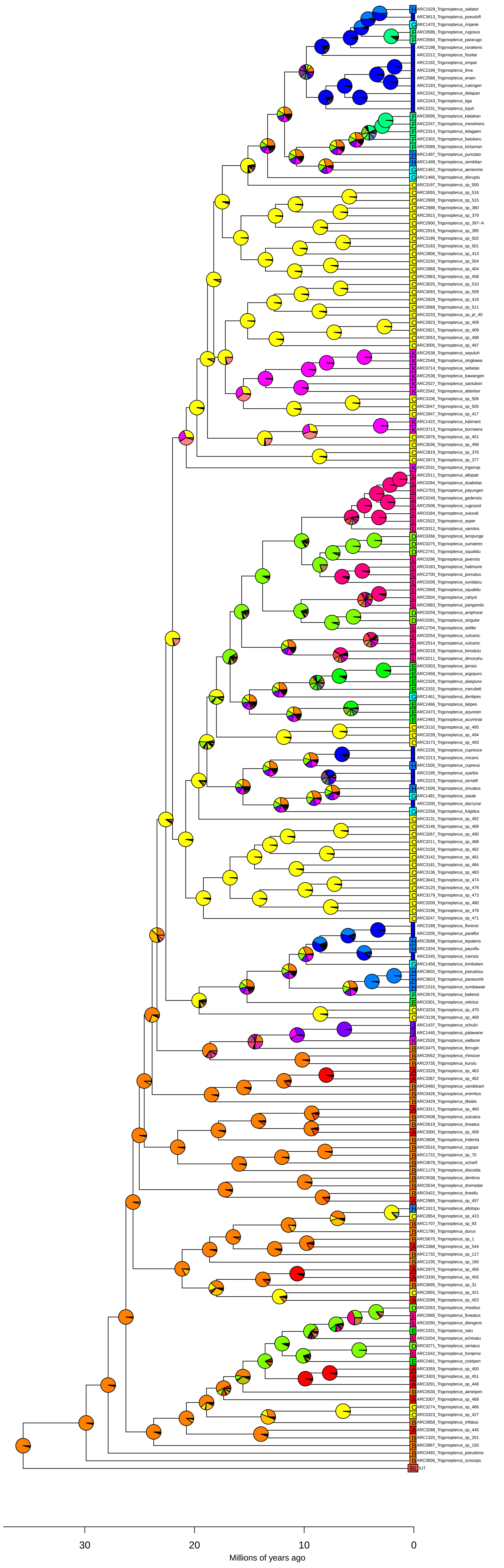

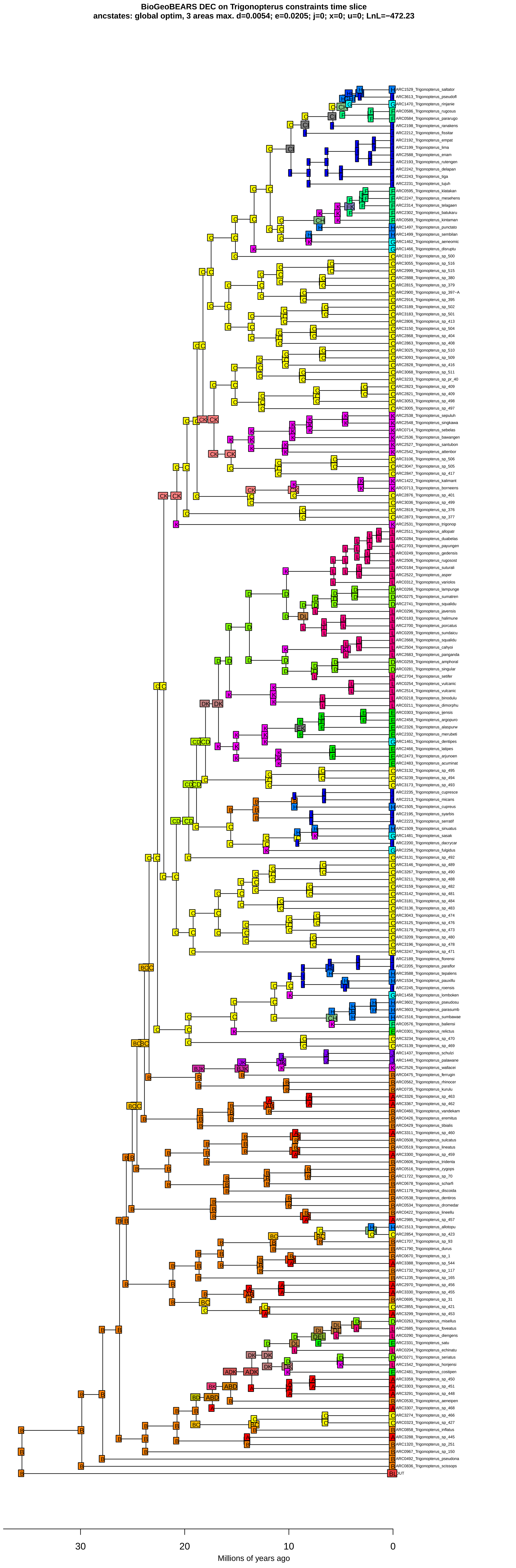
BioGeoBEARS DEC on Trigonopterus constraints time slice
ancstates: global optim, 3 areas max. d=0.0054; e=0.0205; j=0; x=0; u=0; LnL=−472.23
30
20
10
0
Millions of years ago

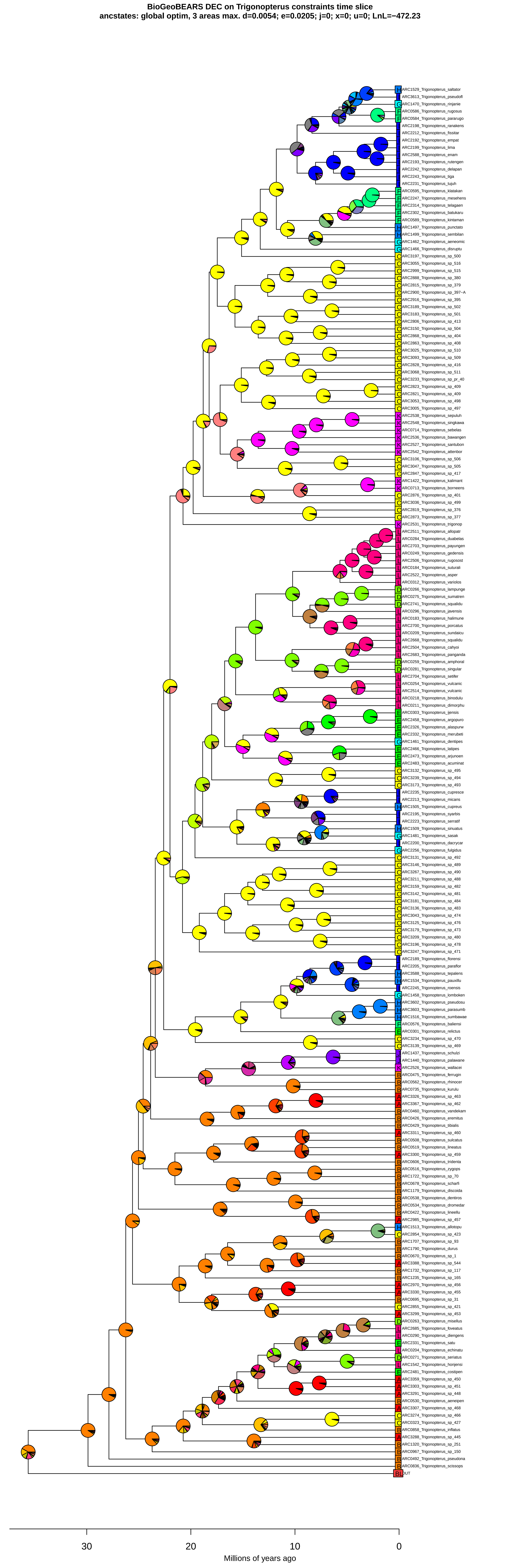
BioGeoBEARS DEC on Trigonopterus constraints time slice
ancstates: global optim, 3 areas max. d=0.0054; e=0.0205; j=0; x=0; u=0; LnL=−472.23
30
20
10
0
Millions of years ago

BioGeoBEARS DEC+J on Trigonopterus constraints time slice
ancstates: global optim, 3 areas max. d=0.0023; e=0.0117; j=0.0336; x=0; u=0; LnL=-424.77

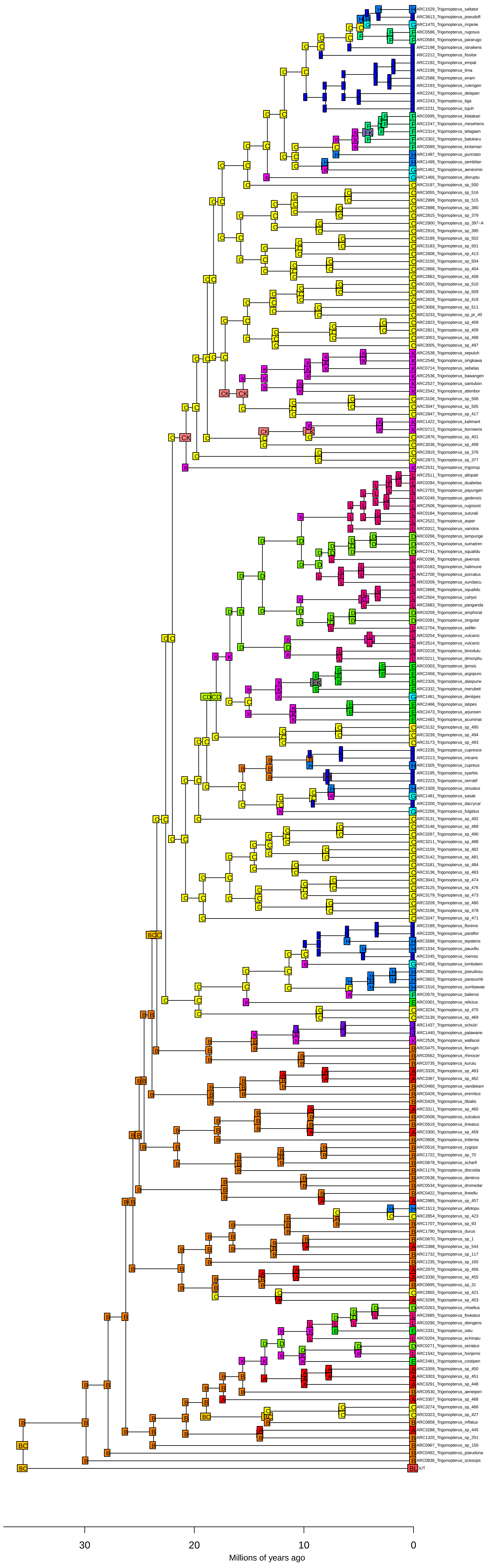

BioGeoBEARS DEC+J on Trigonopterus constraints time slice
ancstates: global optim, 3 areas max. d=0.0023; e=0.0117; j=0.0336; x=0; u=0; LnL=-424.77

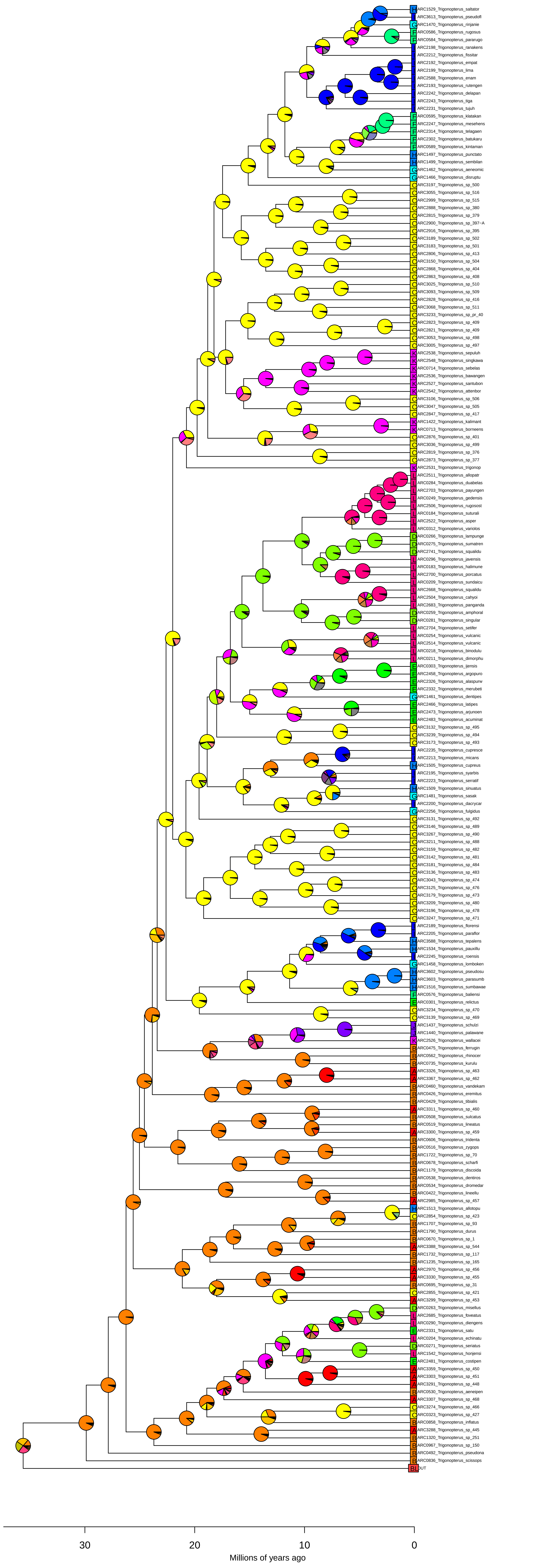

BioGeoBEARS DEC on Trigonopterus Lower Sunda Open time slice
ancstates: global optim, 3 areas max. d=0.0047; e=0.0214; j=0; x=0; u=0; LnL=−478.37

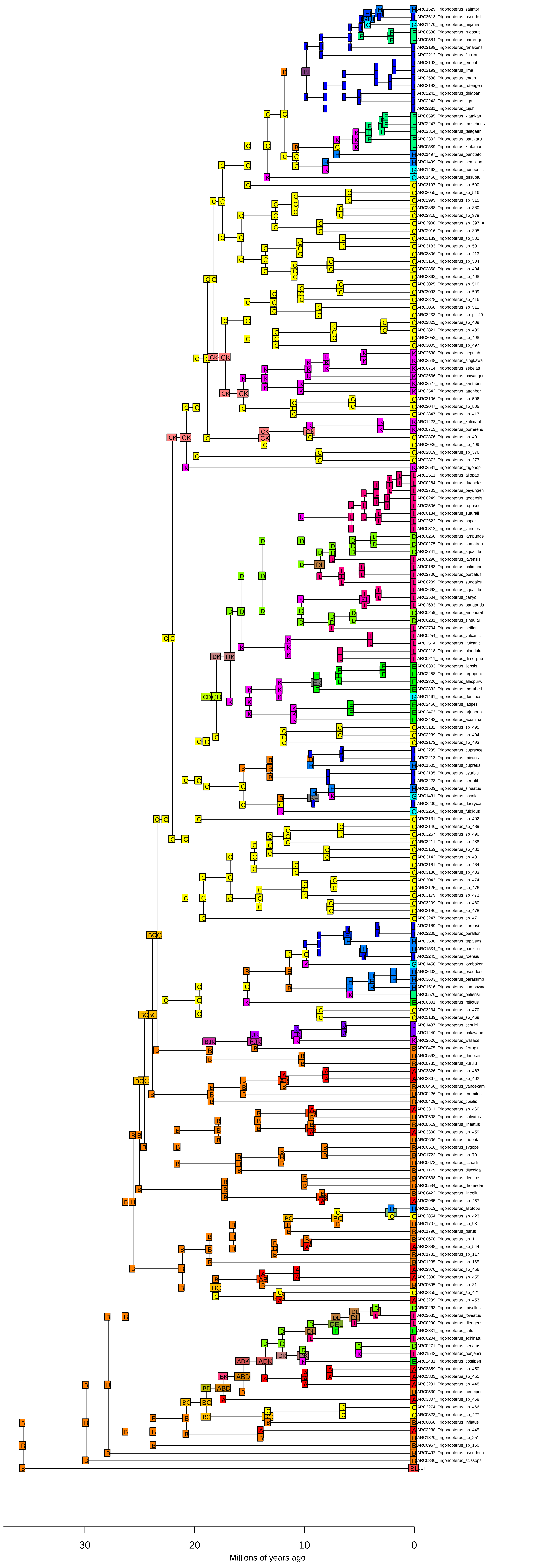

BioGeoBEARS DEC on Trigonopterus Lower Sunda Open time slice
ancstates: global optim, 3 areas max. d=0.0047; e=0.0214; j=0; x=0; u=0; LnL=−478.37

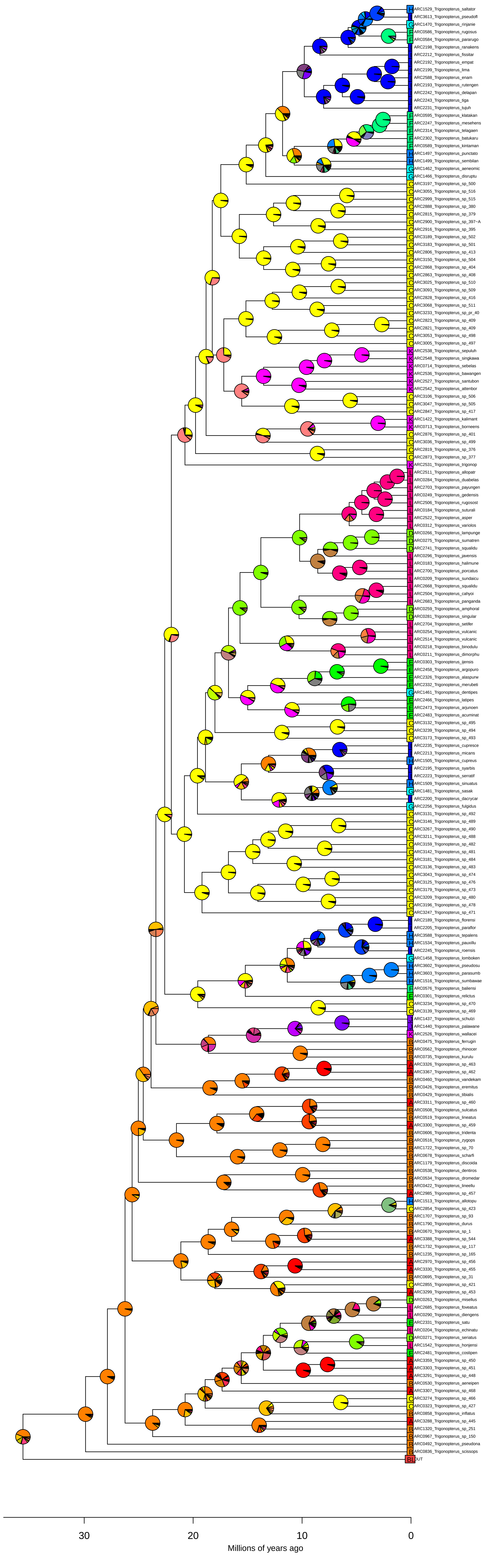

BioGeoBEARS DEC+J on Trigonopterus Lower Sunda Open time slice
ancstates: global optim, 3 areas max. d=0.0024; e=0.0138; j=0.0232; x=0; u=0; LnL=-443.58

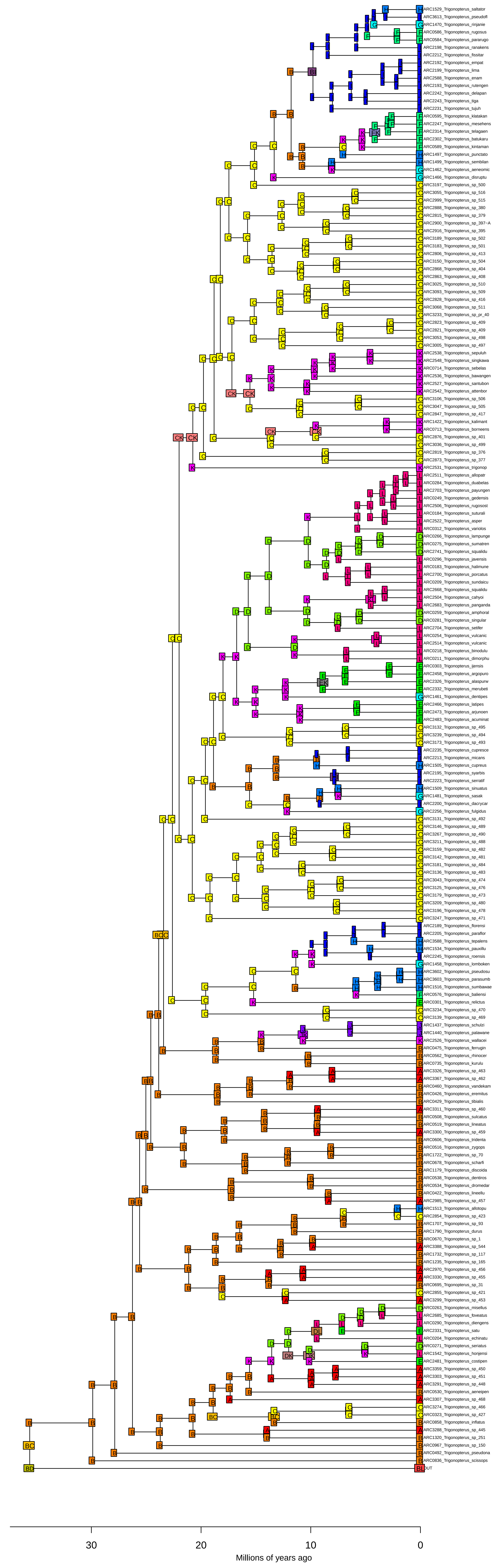

BioGeoBEARS DEC+J on Trigonopterus Lower Sunda Open time slice
ancstates: global optim, 3 areas max. d=0.0024; e=0.0138; j=0.0232; x=0; u=0; LnL=−443.58

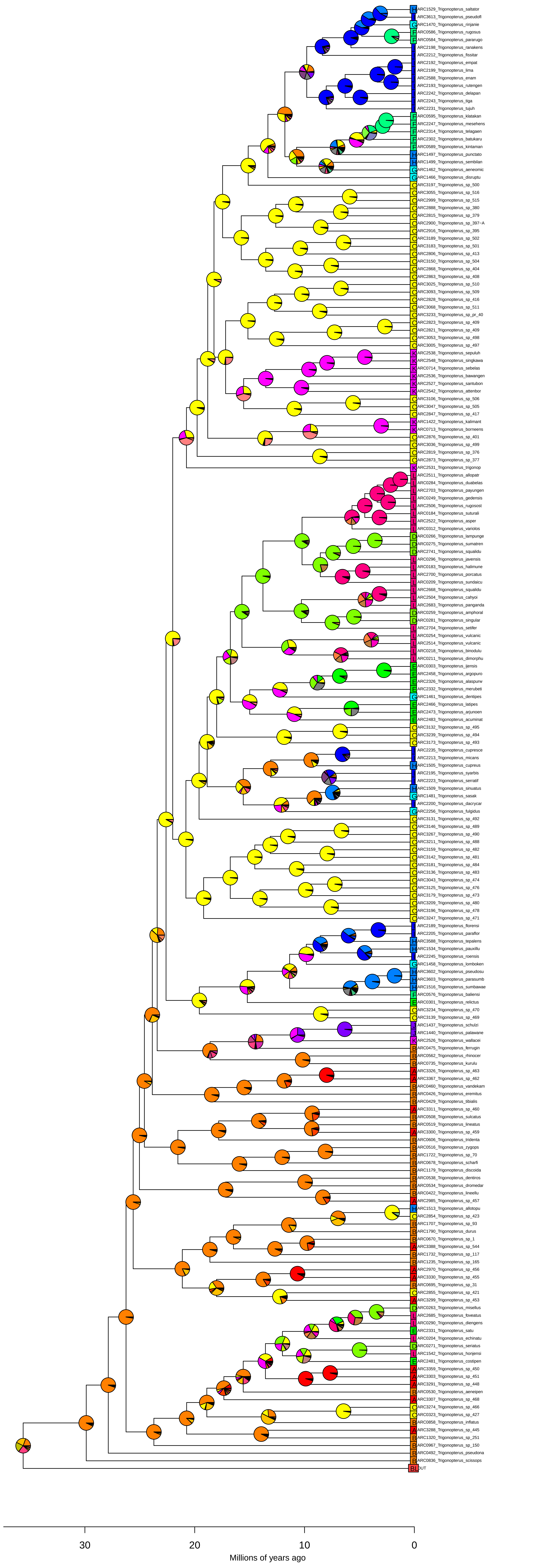

BioGeoBEARS DEC+x on Trigonopterus allopen time slice
ancstates: global optim, 3 areas max. d=0.0045; e=0.0191; x=−0.0441; j=0; LnL=−474.13

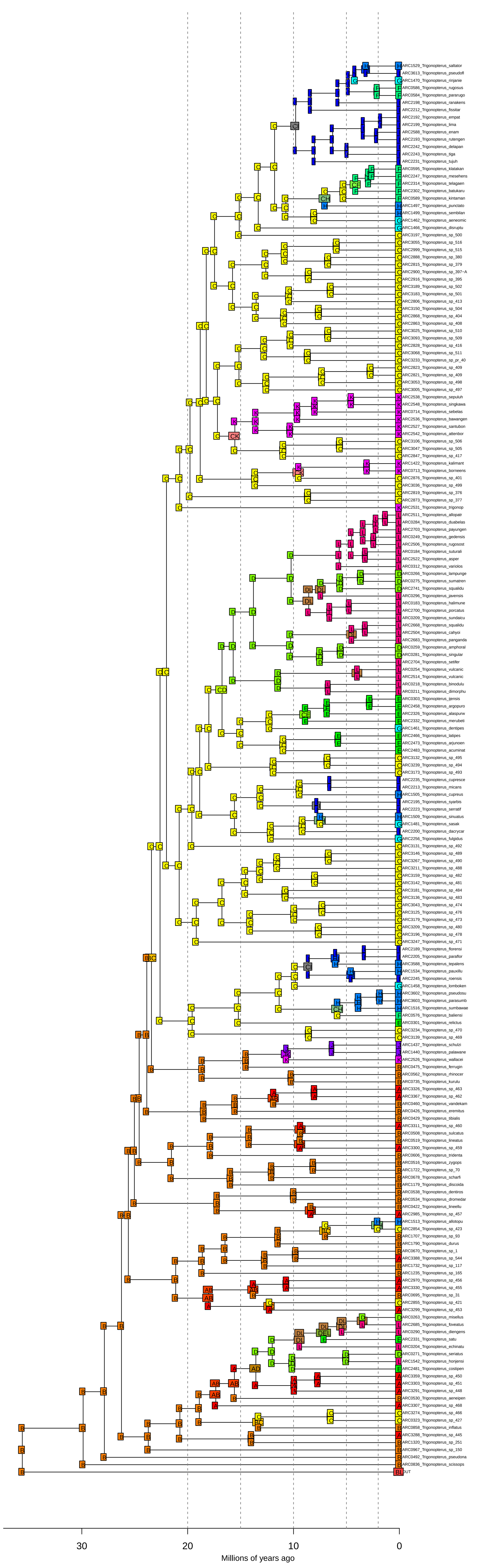

BioGeoBEARS DEC+x on Trigonopterus allopen time slice
ancstates: global optim, 3 areas max. d=0.0045; e=0.0191; x=−0.0441; j=0; LnL=−474.13

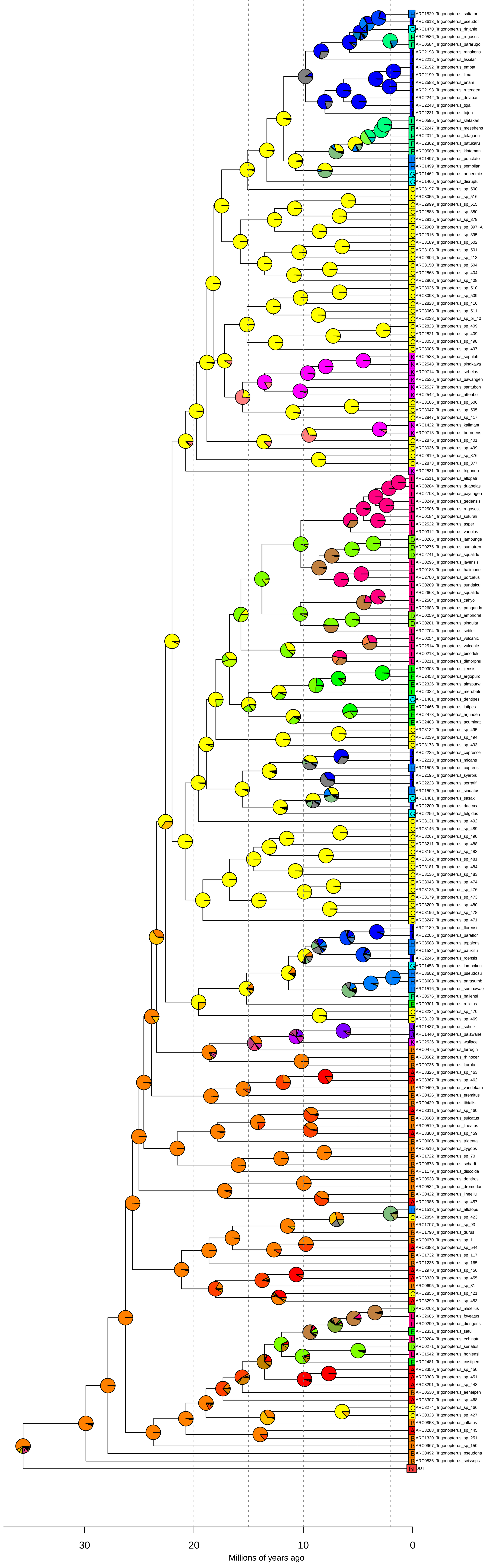

BioGeoBEARS DEC+x+j on Trigonopterus allopen time slice
ancstates: global optim, 3 areas max. d=0.0014; e=0.0107; x=−0.0029; j=0.0224; LnL=−429.83

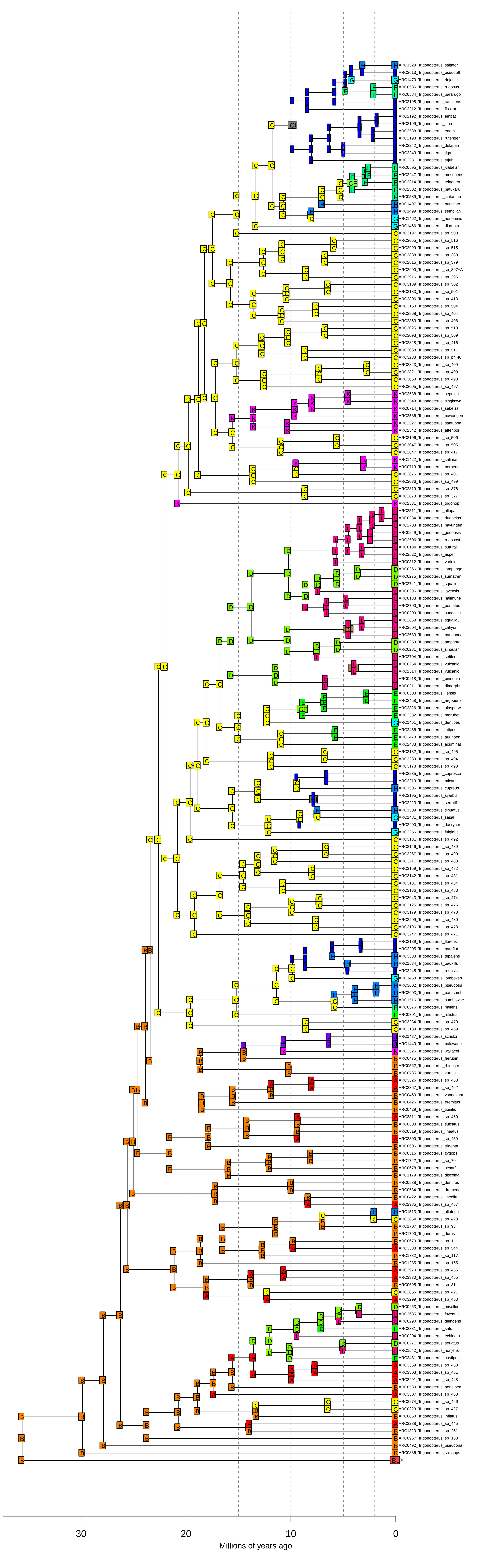

BioGeoBEARS DEC+x+j on Trigonopterus allopen time slice
ancstates: global optim, 3 areas max. d=0.0014; e=0.0107; x=−0.0029; j=0.0224; LnL=−429.83

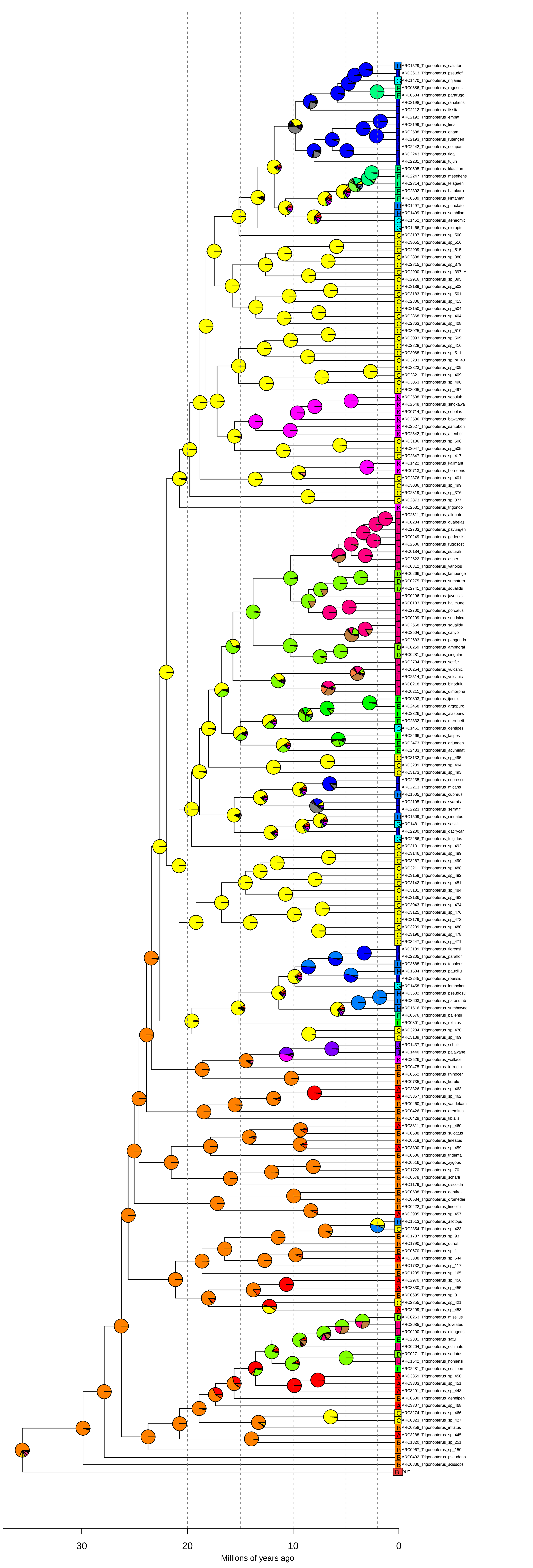

BioGeoBEARS DEC+x on Trigonopterus constraints time slice
ancstates: global optim, 3 areas max. d=0.0308; e=0.0177; x=−0.3367; j=0; LnL=−459.80

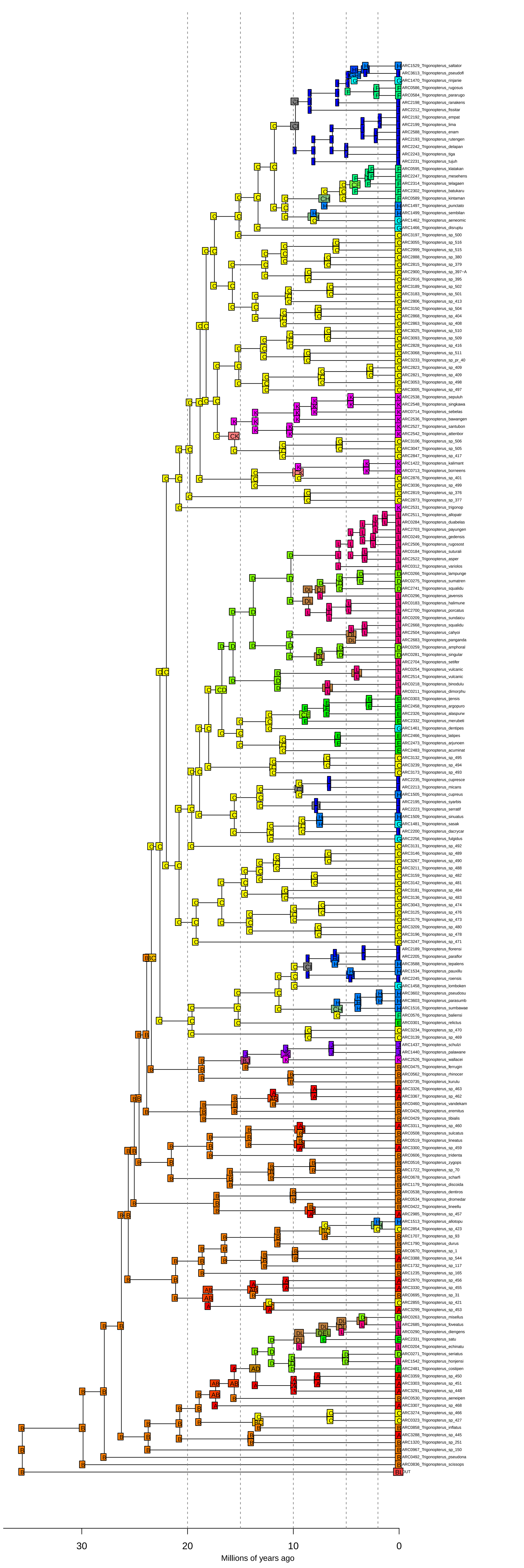

BioGeoBEARS DEC+x on Trigonopterus constraints time slice
ancstates: global optim, 3 areas max. d=0.0308; e=0.0177; x=−0.3367; j=0; LnL=−459.80

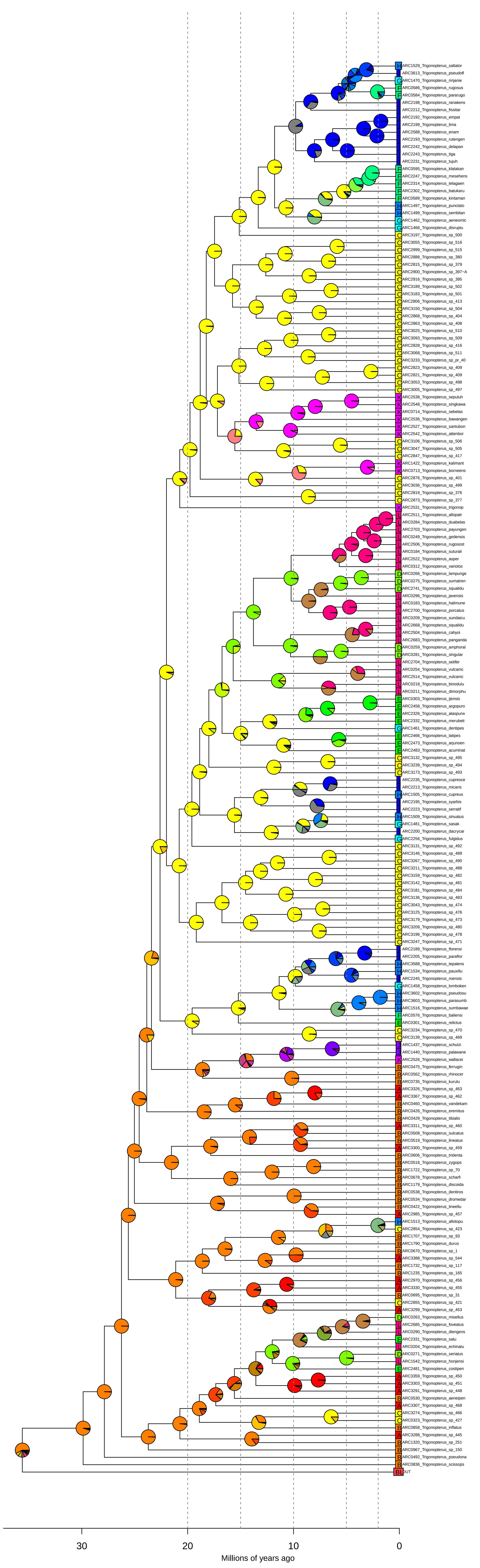

BioGeoBEARS DEC+J+x on Trigonopterus constraints time slice
ancstates: global optim, 3 areas max. d=0.0015; e=0.011; x=−0.0044; j=0.0214; LnL=−429.69

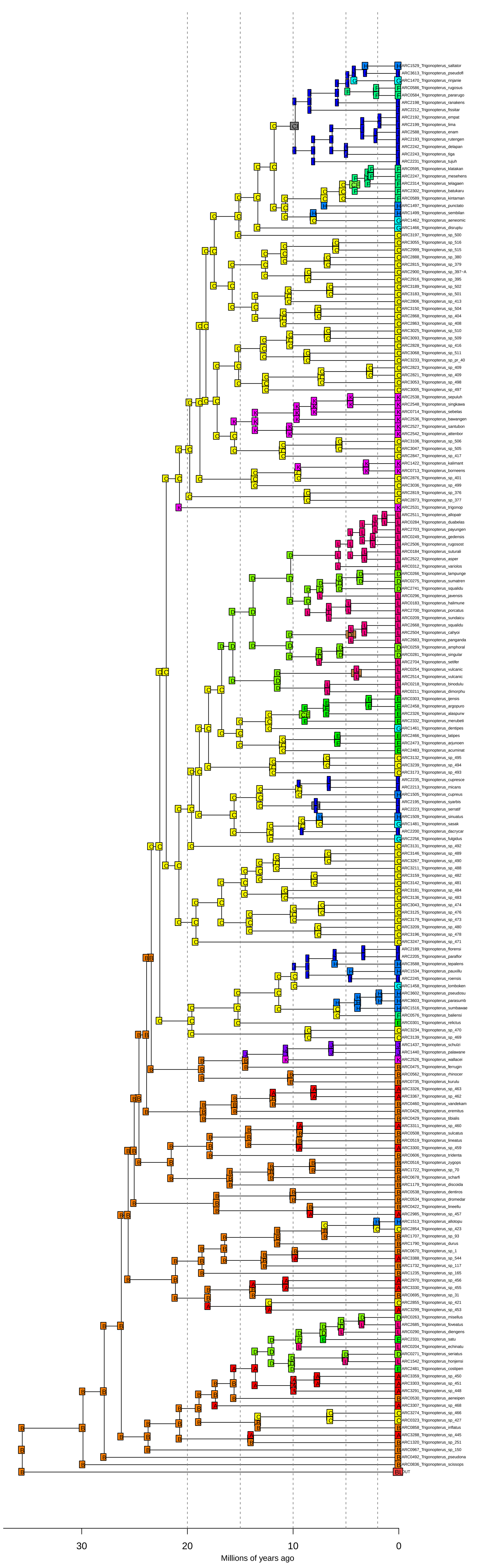

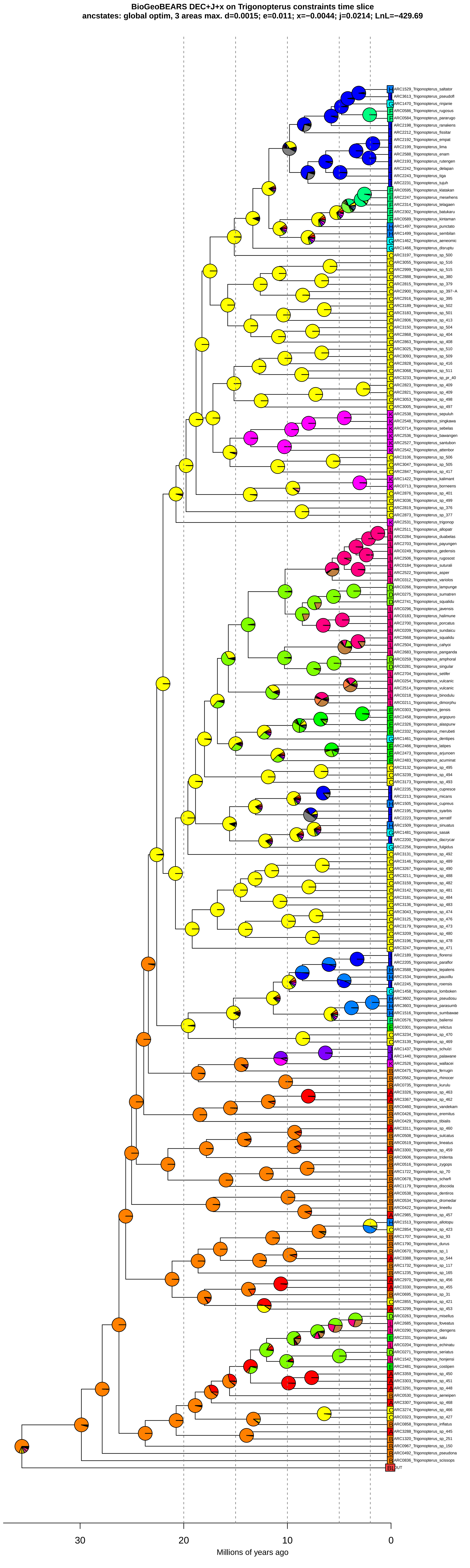
BioGeoBEARS DEC+J+x on Trigonopterus constraints time slice
ancstates: global optim, 3 areas max. d=0.0015; e=0.011; x=−0.0044; j=0.0214; LnL=−429.69
30
20
10
0
Millions of years ago

BioGeoBEARS DEC+x on Trigonopterus Lower Sunda Open time slice
ancstates: global optim, 3 areas max. d=0.0042; e=0.0183; x=−0.0372; j=0; LnL=−474.04

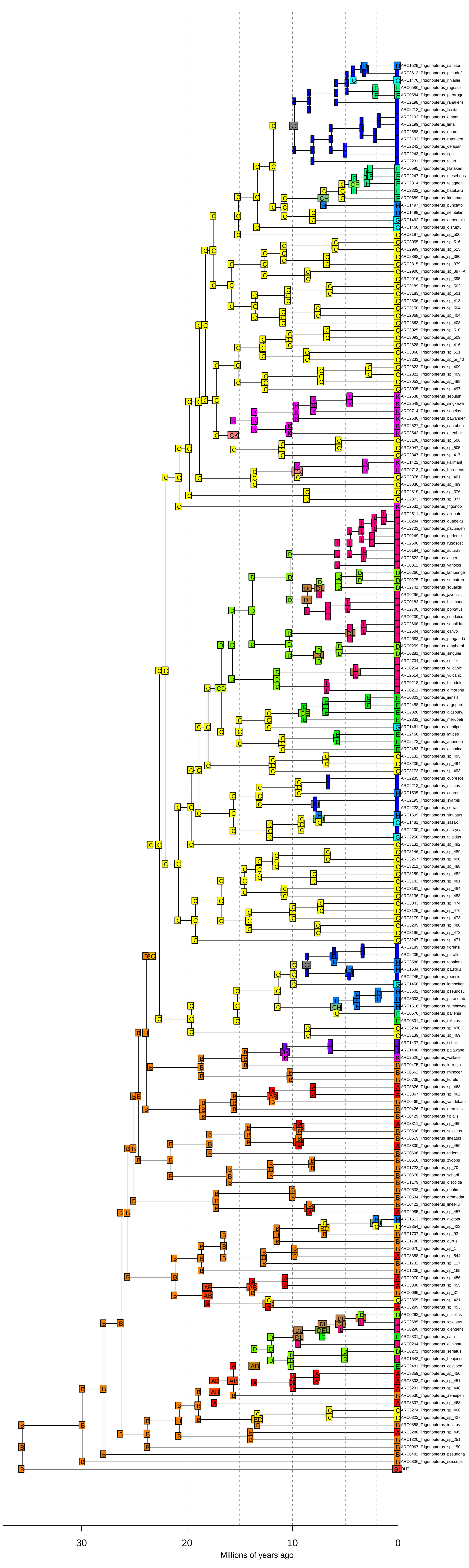

BioGeoBEARS DEC+x on Trigonopterus Lower Sunda Open time slice
ancstates: global optim, 3 areas max. d=0.0042; e=0.0183; x=−0.0372; j=0; LnL=−474.04

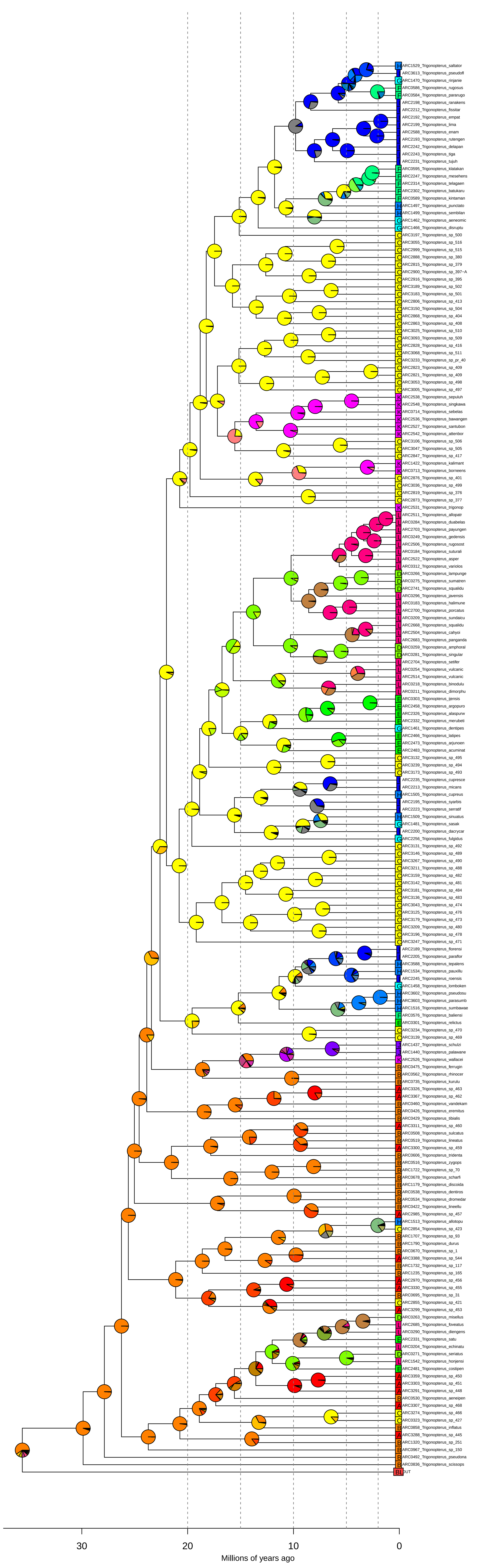

BioGeoBEARS DEC+J+x on Trigonopterus Lower Sunda Open time slice
ancstates: global optim, 3 areas max. d=0.0014; e=0.0109; x=−0.0031; j=0.0225; LnL=−429.79

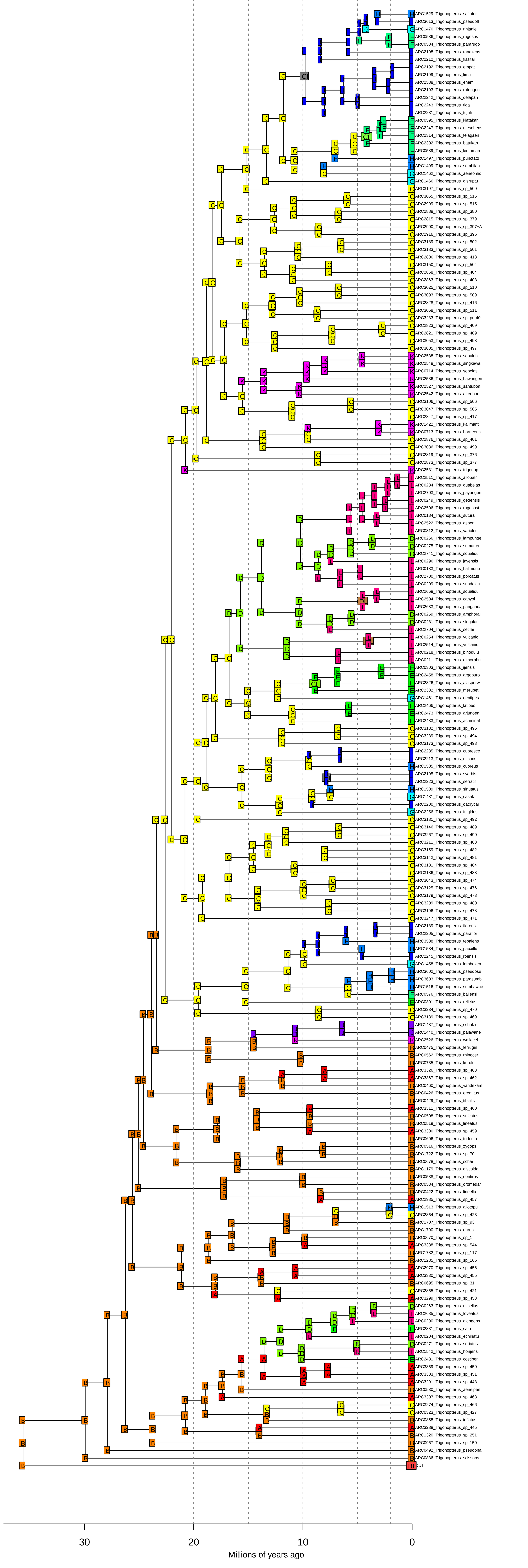

BioGeoBEARS DEC+J+x on Trigonopterus Lower Sunda Open time slice
ancstates: global optim, 3 areas max. d=0.0014; e=0.0109; x=−0.0031; j=0.0225; LnL=−429.79

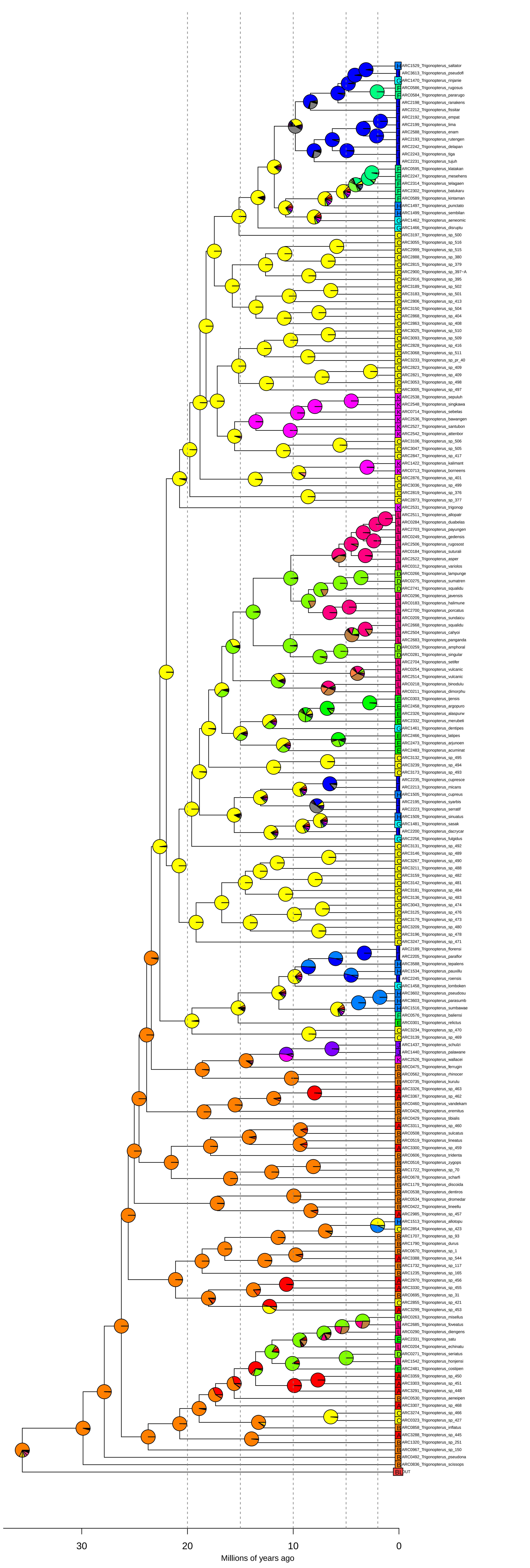

Supplement: Supplementary Information S2 [file srep18793-s2.pdf]
